# Transposons and satellite DNA: on the origin of the major satellite DNA family in the *Chenopodium* genome

**DOI:** 10.1186/s13100-020-00219-7

**Published:** 2020-06-26

**Authors:** Alexander Belyayev, Jiřina Josefiová, Michaela Jandová, Václav Mahelka, Karol Krak, Bohumil Mandák

**Affiliations:** 1 Czech Academy of Sciences, Institute of Botany, Zámek 1, CZ-252 43 Průhonice, Czech Republic; 2 Faculty of Environmental Sciences, Czech University of Life Sciences Prague, Kamýcká 129, 165 00 Praha, Suchdol Czech Republic

**Keywords:** CACTA transposons, Satellite DNA, Transposase, *Chenopodium*, Next generation sequencing, Oxford Nanopore sequencing

## Abstract

Extensive and complex links exist between transposable elements (TEs) and satellite DNA (satDNA), which are the two largest fractions of eukaryotic genome. These relationships have a crucial effect on genome structure, function and evolution. Here, we report a novel case of mutual relationships between TEs and satDNA. In the genomes of *Chenopodium* s. str. species, the deletion derivatives of *tnp2* conserved domain of the newly discovered CACTA-like TE *Jozin* are involved in generating monomers of the most abundant satDNA family of the *Chenopodium* satellitome. The analysis of the relative positions of satDNA and different TEs utilizing assembled Illumina reads revealed several associations between satDNA arrays and the transposases of putative CACTA-like elements when an ~ 40 bp fragment of *tnp2* served as the start monomer of the satDNA array. The high degree of identity of the consensus satDNA monomers of the investigated species and the *tnp2* fragment (from 82.1 to 94.9%) provides evidence of the genesis of CficCl-61-40 satDNA family monomers from analogous regions of their respective parental elements. The results were confirmed via molecular genetic methods and Oxford Nanopore sequencing. The discovered phenomenon leads to the continuous replenishment of species genomes with new identical satDNA monomers, which in turn may increase species satellitomes similarity.

## Introduction

Transposable elements (TEs) and satellite DNA (satDNA) are the major components of the eukaryotic genome repeatome, accounting for up to 90% of plant nuclear DNA [1]. Among the internal sources of genotypic change, TEs can be considered the most powerful due to their ability to move, insert at novel locations and thereby shape and induce the specialization of the landscapes of coding and non-coding DNA fractions [2–4]. In contrast, satDNA, which consists of long, late-replicating, noncoding arrays of tandemly arranged monomers and is predominantly concentrated in the heterochromatic regions of chromosomes [5, 6], is non-mobile, although recent studies suggest its involvement in various functions ranging from chromosome organization and pairing to cell metabolism and the modulation of gene functions [1, 7–10].

The relationships between these two large fractions are fascinating. Extensive and complex links between TEs and satDNAs exist in eukaryotic genome, creating a complex network of sequences that has a crucial effect on genome structure, function and evolution [10]. There is growing evidence of the involvement of TEs in generating a library of tandem repeats that can be dispersed throughout the genome and, in some cases, amplified into long arrays of new satDNAs [10–12]. Recently, the possibility of elucidating the details of this process by using next-generation sequencing (NGS) technology has arisen through the comparative analysis of entire species repeatomes.

Genome assemblies resulting from the alignment and merging of short DNA fragments to reconstruct the original sequence allow the determination of the interposition of genome elements and, to a certain extent, the possible links between them. For the analysis of the interrelationships between TEs and satDNA, we utilized the genomes of *Chenopodium* s. str. (also referred to as the *Chenopodium album* aggregate). Species of the *C. album* aggregate are distributed worldwide, with the highest species diversity in temperate areas [13]. The majority of the species in this diploid-polyploid complex are phenotypically exceptionally plastic [14] and are able to grow under a wide range of conditions [15]. Eight monophyletic lineages have been recognized within the Eurasian representatives of this group [16]. Five of these clades are represented by extant diploid species, whereas the remaining three were reconstructed based on the sequences found in polyploid taxa and are considered to have originated from extinct or still unknown species. All tetraploid taxa were found to be of allopolyploid origin arising as a result of hybridization between diploids from different clades. The allohexaploids exhibit a combined tetraploid and diploid genome (for the genomic composition of the analyzed taxa, see Table 1). The evolutionary history of this typical angiosperm group revealed by key molecular-phylogenetic markers can be briefly described as follows: the early differentiation of the *C. album* aggregate coincided with the beginning of the Miocene Climatic Optimum ~ 20 Mya. Clade H separated upon the transition between the Serravallian and Tortonian Ages, ~ 11 Mya. However, the main lineages were formed in the Pliocene. Subsequent speciation within the lineages and the appearance of the majority of polyploids took place in the Quaternary Period [16].

**Table 1:** Chenopodium species used for the study, their genome composition, ploidy, genome size and geographical origin

Species (accession number)	Genome composition [16]	Locality	Coordinates	Chr. No	Genome Size Mbp
*C. acerifolium* (316–1)	B + D	Russian Federation, Velsk	N 61.066704 E 42.095002	2n = 4x = 36	2570
*C. acuminatum* (429–3)	D	China, Xinjiang, Altaj, Burqin	N 47.815500 E 87.080028	2n = 2x = 18	960
*C. album* (291–1)	B + C + D	Czech Republic, Hrádek	N 48.781583 E 16.261528	2n = 6x = 54	3808
*C. bryoniifolium* (742–4)	A	Russian Federation, Primorski Krai, Nakhodka city district	N 42.88775 E 132.722361	2n = 2x = 18	2608
*C. ficifolium* (330–2)	B	Czech Republic, Slatina	N 50.226389 E 14.210528	2n = 2x = 18	1785
*C. iljinii* (433–9)	E	China, Xinjiang, Altaj, Hoboksar	N 46.541472 E 85.358083	2n = 2x = 18	1144
*C. jenissejense* (640)	B + E	Russian Federation, Verkhnekolymsky raion, Popovka river mouth	N 64.646833 E 151.640306	2n = 4x = 36	2935
*C. karoi* (460)	B + E	China, Xinjiang, Tumuxiukezhen	N 41.667306 E 79.693528	2n = 4x = 36	2929
*C. luteorubrum* (742–17)	A + C + D	Russian Federation, Primorski Krai, Nakhodka city district	N 42.88775 E 132.722361	2n = 6x = 54	3247
*C. novopokrovskyanum* (463–3)	C + D	China, Xinjiang, Tumuxiukezhen	N 41.667306 E 79.693528	2n = 4x = 36	1192
*C. opulifolium* (696–6)	B + C + F	Iran, Kurdistan, Marivan	N 35.498461 E 46.166946	2n = 6x = 54	4421
*C. pamiricum* (830–3)	E	Tajikistan, Gorno-Badakhshan autonomous region, Murghob district	N 37.821667 E 73.566667	2n = 2x = 18	1154
*C. sosnowskyi* (788)	A + G	Iran, west Azerbaijan, Siah Cheshmeh (Chaldoran)	N 39.065972 E 44.386170	2n = 4x = 36	2177
*C. striatiforme* (331–1)	C + D	Czech Republic, Mělník	N 50.349528 E 14.497444	2n = 4x = 36	2029
*C. strictum* (380–5)	C + D	Czech Republic, Prague	N 50.115964 E 14.433326	2n = 4x = 36	2022
*C. suecicum* (328–10)	B	Czech Republic, Švermov	N 50.176806 E 14.105472	2n = 2x = 18	1775
*C. vulvaria* (771–1)	H	Iran, Ardabil, Meshgin Shahr	N 38.405556 E 47.694722	2n = 2x = 18	924

The main characteristic of the *Chenopodium* species satellitome is the presence of a basic repeat unit of approximately 40 bp (CficCl-61-40 satDNA family), that was previously identified and described [17, 18]. The percentage of this satDNA family in the genome of *C. album* aggregate diploid species ranges from 0.25% (*C. pamiricum*) to 3.80% (*C. acuminatum*). It has been shown that this satDNA family is the most abundant and oldest component of the *Chenopodium* genome [18], but its origin is still unclear. In the present study, we aimed to infer the mutual relationships of the CficCl-61-40 satDNA family and TEs by analyzing the Illumina reads of 17 species of the *C. album* aggregate. We hypothesize that the origin of this family of repeats may be associated with the activity of one of the TEs.

## Results and discussion

### Association of TEs and *CficCl-61-40* satDNA family arrays in the genome of *C. acuminatum*

**Fig. 1:**
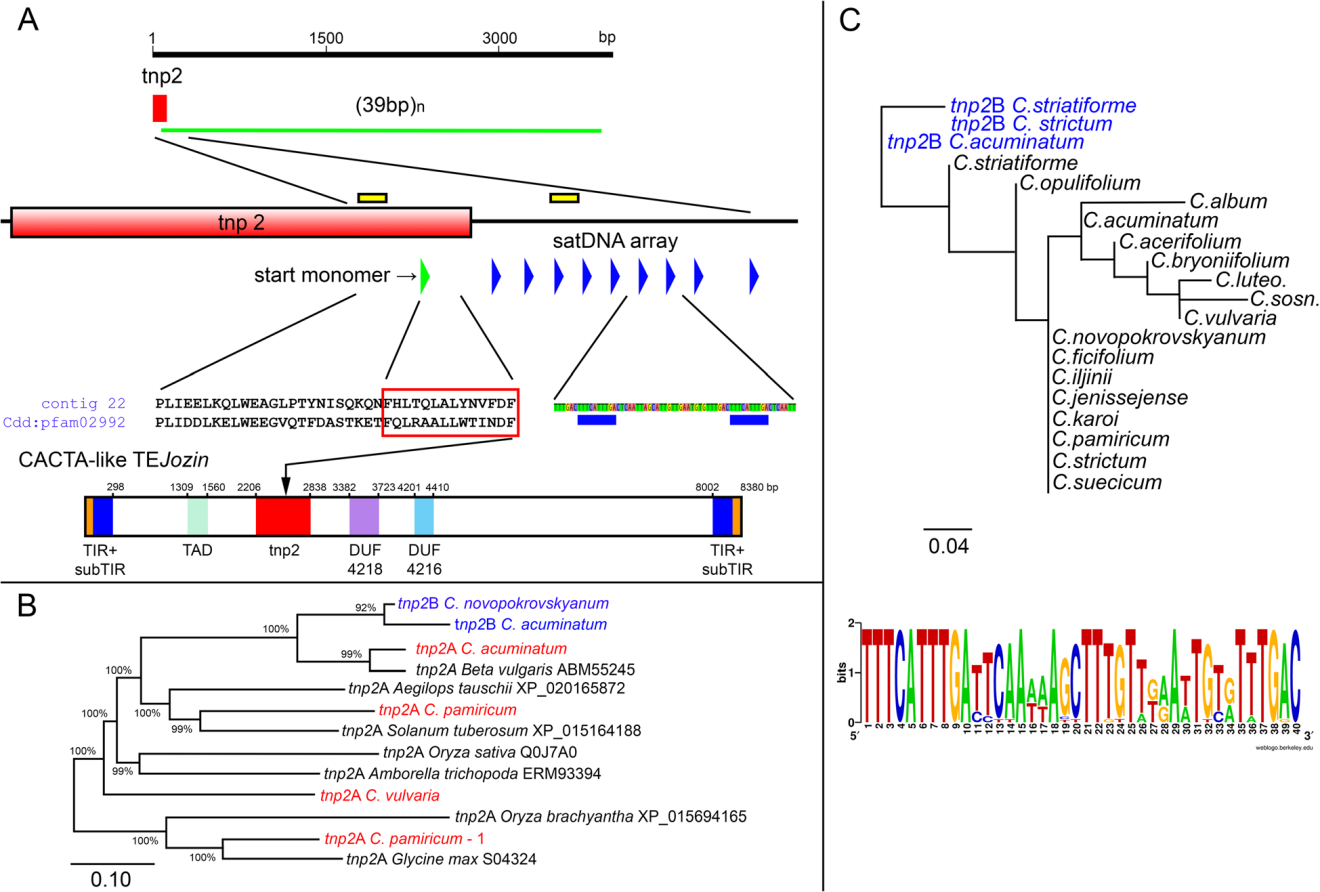
The *tnp2* transposase and the CficCl-61-40 satDNA family. **a** Schematic representation of contig 22 of the assembled *C. acuminatum* genome (the first 4000 bp) at different zoom levels. Red squares are the fragments of *tnp2*. The green line indicates the length of the CficCl-61-40 satDNA array. Blue triangles/squares are conserved motifs of the basic monomer. The green triangle is a similar conserved motif within *tnp2* (parental monomer). The red frame indicates the homologous protein sequence of the start monomer and the similar fragment from the other plant species (Cdd: pfam 02992). The positions of PCR primers used for validation of the physical existence of the association of *tnp2*B with CficCl-61-40 satDNA family arrays are shown with yellow rectangles (see also Fig. 2a). A diagram of the domain organization of the complete CACTA-like TE *Jozin* is shown at the bottom of **a** (see also Additional file 3). The 3′ position of the parent for the CficCl-61-40 satDNA array start monomer is shown with an arrow (for further explanation see the text). **b** Phylogenetic relationships of conserved protein domains of the *tnp2* transposase family*. Tnp2*A in the genomes of the species of the *C. album* aggregate is highlighted in red. *Tnp2*B in the genomes of the species of *C. album* aggregate is highlighted in blue. GenBank accession numbers follow the plant species name. **c** Phylogenetic relationships of CficCl-61-40 satDNA family monomers and corresponding fragments of *tnp2*B (the latter are highlighted in blue). A graphical representation of the conservation of CficCl-61-40 satDNA family monomers by sequence logo is shown at the bottom of **c** (Additional file 2.1)

The application of the Geneious Prime assembler [19] to the Illumina reads (genome coverage 0.4–0.9x) of seventeen diploid and polyploid *C. album* aggregate species (Table 1) resulted in the identification of CficCl-61-40 satDNA family arrays. The analysis of the relative positions of satDNAs and different TEs in the genome of *C. acuminatum* revealed cases of their colocalization with TEs (mainly with LTR retrotransposons), similar to the findings described by Heitkam et al. [20]. This is not surprising given that satDNA could be a target for TE insertions [21]. However, the comparative sequence analysis of CficCl-61-40 satDNA family arrays and retrotransposons revealed no significant similarities, and these cases could be regarded solely as insertions. In contrast to retrotransposons, several cases of interactions between the transposases (TPases) of DNA transposons (particularly those of putative CACTA-like elements) and CficCl-61-40 satDNA family arrays were revealed. Thus, in contig 22 of the *C. acuminatum* CficCl-61-40 satDNA family array of 94.4 monomers with a consensus sequence of 39 bp was attached to the incomplete *tnp2* TPase domain of 115 bp, and the start monomer of the array was simultaneously a *tnp2* fragment of 41 bp oriented in the same direction (Fig. 1a, Additional file 1). The similarity of the *tnp2* fragment and the consensus sequence of the adjacent array was very high, at 89.7%. Another case was found in contig 150 of the same species, in which a fragment of the *tnp2* TPase domain of 314 bp was attached to a CficCl-61-40 satDNA family array of 8.4 monomers. The similarity of the *tnp2* fragment and the consensus sequence of the adjacent array was again 89.7%. In contig 431, a similar fragment of *tnp2* of 293 bp and a CficCl-61-40 satDNA family array of 23.1 monomers were associated. The similarity of the *tnp2* fragment and the consensus sequence of the adjacent array was 89.9%. Finally, in contig 545, a fragment of *tnp2* of 389 bp was associated with a CficCl-61-40 satDNA family array of 15.2 monomers, with the highest similarity of corresponding fragments reaching 94.9%. Thus, in the genome of *C. acuminatum,* significant nucleotide similarities were found between monomers of the CficCl-61-40 satDNA family and the ~ 40 bp fragment of the *tnp2* domain, and it is possible to claim with a high probability that these monomers are derived from a similar region of their parent element, the ~ 40 bp fragment of *tnp2* of the putative CACTA-like transposon. Alternatively, but less likely, is a scenario in which minisatellites were captured by CACTA-like elements. Indeed, the capture of a single monomer or several monomers is possible, but this could not have led to the formation of a long associated array of the CficCl-61-40 satDNA family, as we observed in the genome of *C. acuminatum* (see below). It is also essential to note that among all studied species, the genome of *C. acuminatum* exhibits the highest contents of the CficCl-61-40 satDNA family (3.80% of the whole genome [18]).

A BLAST search for conserved domains identified the parental fragment as belonging to the TPase family member *tnp2* pfam02992, a member of the cl29371 superfamily analogous to other *tnp2* domains identified in several plants from different taxonomic groups [22] (Additional files 1, 2). Geneious Prime made it possible to detect the complete sequence of the *tnp2* domain in the assembled contigs of the *C. album* aggregate species genomes (see Material and Methods), which show high similarity to known sequences from GenBank (Fig. 1b, Additional file 2). The length of the *tnp2* domains detected in the explored species was approximately 630 bp. Surprisingly, within the complete *tnp2* domains (which are usually assembled in a single contig with DUF4216, DUF4218 and TPase-associated domains), we did not find the conserved TTTCATTTGA motif corresponding to the beginning of the *tnp2* fragment parent of the CficCl-61-40 satDNA family array. Here, it is essential to note that in all cases recorded in the genome of *C. acuminatum,* only incomplete *tnp2* domains of 115–389 bp could be recognized as parental to the CficCl-61-40 satDNA family array. Although these partial *tnp2* domains were assigned to the pfam02992 family, they differed from the complete domains. Phylogenetic analysis showed them to form a separate branch derived from the internal regions (approximately positions 63–357, Fig. 1a, Additional file 2) of complete domains (Fig. 1b). These sequences could be assumed to be part of a putative novel short CACTA-like element but probably represent modified deletion derivatives of *tnp2*. These CACTA-related components of the *Chenopodium* genomes will hereafter be referred to as *tnp2*A (complete domains) and *tnp2*B (deletion derivatives parental to CficCl-61-40 satDNA family arrays).

### The *tnp2B and CficCl-61-40* satDNA family *array associations in genomes of other species of the C. album aggregate*

Searches in the genomes of the other studied species using the conserved motif specific to *tnp2*B (see Material and Methods) revealed six additional cases of *tnp2*B and CficCl-61-40 satDNA family array links. The results are presented in Table 2 and Additional file 1. In the genomes of diploid *C. ficifolium* (B-genome), the tetraploids *C. acerifolium* (B- and D-genomes) and *C. novopokrovskyanum* (C- and D-genomes), and the hexaploid *C. luteorubrum* (A-, C- and D-genomes), fragments of *tnp2*B were identified, although without an association with CficCl-61-40 satDNA family arrays. In the other species, we did not find any *tnp2*B fragments, although domains of the *tnp2*A type were detectable, indicating the presence of CACTA transposons. Thus, we identified a complete putative CACTA element, which we refer to as *Jozin*, in the assembled genomes of *C. pamiricum* and *C. sosnowskyi*. The length of the element is approximately 8300 bp with similarity between the *tnp2*A conserved domains of 92.9%. The characteristics of the elements and their sequences are presented in Additional file 3 and Fig. 1a. Therefore, the distribution of *tnp2*B is uneven among the *C. album* aggregate species. All cases of *tnp2*B and CficCl-61-40 satDNA family array associations were connected with the D-genome (diploid *C. acuminatum* and polyploids harboring the D-genome as a haplome, including *C. album* s. str.*, C. striatiforme* and *C. strictum*), while most of the B-genome-containing species (Tables 1 and 2) possessed fragments of *tnp2*B, but no association with the CficCl-61-40 satDNA family arrays was found in silico.

**Table 2:** Presence and association of *tnp2*B and CficCl-61-40 satDNA family arrays (see also Additional file 1)

Species	Genomes	*tnp2B*	*tnp2B*+ array	Contig No	*tnp2B* lengths	Array copy-numb	Similarity *tnp2B-*array
*C. acerifolium*	B + D	yes	no	–	–	–	–
*C. album*	B + C + D	yes	yes	1990	579 bp	12.0	89.7%
*C. bryoniifolium*	A	no	no	–	–	–	–
*C. ficifolium*	B	yes	no	–	–	–	–
*C. iljinii*	E	no	no	–	–	–	–
*C. jenissejense*	B + E	no	no	–	–	–	–
*C. karoi*	B + E	no	no	–	–	–	–
*C. luteorubrum*	A + C + D	yes	no	–	–	–	–
*C. novopokrovskyanum*	C + D	yes	no	–	–	–	–
*C. opulifolium*	B + C + F	no	no	–	–	–	–
*C. pamiricum*	E	no	no	–	–	–	–
*C. sosnowskyi*	A + G	no	no	–	–	–	–
*C. striatiforme*	C + D	yes	yes	541	134 bp	54.2	89.8%
				28,391	371 bp	22.2	94.9%
*C. strictum*	C + D	yes	yes	700	371 bp	27.9	90.0%
				10,973	386 bp	17.4	94.9%
				11,346	341 bp	27.1	80.0%
*C. suecicum*	B	no	no	–	–	–	–
*C. vulvaria*	H	no	no	–	–	–	–

These data raise a key question: is the discovered mechanism of satDNA family monomer formation unique to D- and, probably, B-genome lineages, or has it arisen independently and repeatedly during the evolution of a fairly broad group of *Amaranthaceae* species? The latter possibility can be indirectly supported by the interspecific similarity of basic monomers: a higher similarity indicates a greater probability that this mechanism exists or existed previously in a certain genome. Thus, to assess the homology of CficCl-61-40 satDNA family monomers in all investigated species, tandem repeat finder (TRF) was used, and consensus monomers were identified. Related HOR units [18] were excluded from the analysis, and monomers were aligned according to the conserved TTTCATTTGA motif, which was present in all monomers and corresponds to the beginning of the *tnp2*B parental fragment (Additional file 2). The corresponding parental fragments of *tnp2*B were also included. The similarity between the consensus monomers and parental *tnp2*B fragments ranged from 82.1 to 94.9% (Fig. 1c). Such high similarity makes it possible to suggest that CficCl-61-40 satDNA family monomers are derived from similar regions of the parental ~ 40 bp fragment of *tnp2*B in all studied genomes and that this event took place at least once in their evolutionary history. Moreover, the similar conserved motif present in *Beta corolliflora* minisatellites of 40 bp (GenBank AJ288880.1) [23] extends the existence of the mechanism further back in time.

### Experimental validation of the computationally identified structures

**Fig. 2:**
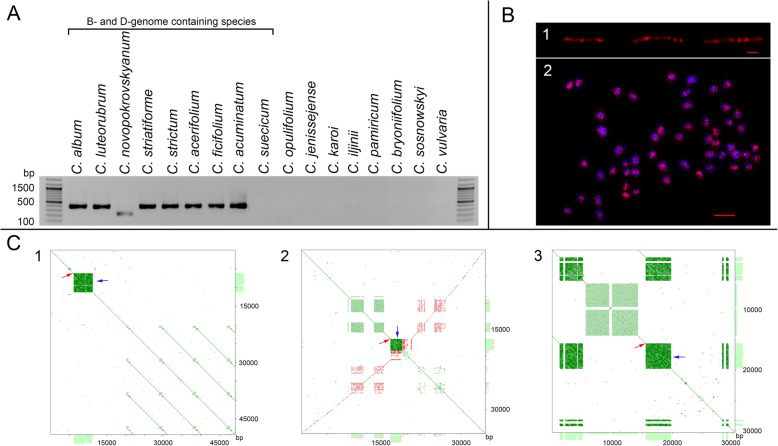
Experimental validation of the computationally identified structures. **a** PCR screening for the association of *tnp2*B with CficCl-61-40 satDNA family arrays. **b** (1) Fiber-FISH analysis of DNA strands of *C. acuminatum*. The CficCl-61-40 satDNA family probe (red signal) is associated with arrays (three examples). The bar represents 1 μm. (2) The distribution of CficCl-61-40 satDNA family sequences in the chromosomes of *C. album* s. str. CficCl-61-40 is labeled with Cy-3 (red signal); chromosomes are stained with DAPI (blue signal). The bar represents 5 μm. **c** Self-to-self comparisons of the three Oxford Nanopore ultralong reads from the *C. acuminatum* genome (Additional file 4) displayed as dot plots (YASS program output). Parallel lines indicate tandem repeats (the distance between the diagonals equals the lengths of the motifs). Histograms at the axes indicate the regions with tandem repeats. (1) Read #17 of 49,142 bp; (2) Read #131 of 34,531 bp; (3) Read # 313 of 30,368 bp. The positions of the *tnp2B* parental fragments are indicated with red arrows, and the associated CficCl-61-40 satDNA family arrays (squares) are indicated with blue arrows

The generation of consensus sequences by assembling reads into contigs is problematic for satellites due to their tandem structure [18]. Thus, we aimed to confirm the existence of the physical counterparts of the computer-generated associations in the genome of *C. acuminatum*. The physical existence of the association of *tnp2*B with CficCl-61-40 satDNA family arrays in the genome of *C. acuminatum* was supported by PCR screening, in which the forward, outward-facing primer corresponded to the conserved motif of *tnp2*B and the reverse primer to the CficCl-61-40 satDNA family array (Fig. 1a). The presence of a clear PCR band of the expected size (~ 400 bp) confirmed the existence of associations between *tnp2*B and CficCl-61-40 satDNA family arrays in the genome of *C. acuminatum* (Fig. 2a). The cloning results for the PCR-amplified DNA fragments showed 95.8–96.3% similarity with the assembled reference sequence, which indicates the accuracy of the assembly algorithm. The PCR screening of the other species revealed putative associations of *tnp2*B and CficCl-61-40 satDNA family arrays in the genomes of *C. acerifolium, C. ficifolium, C. luteorubrum* and *C. novopokrovskyanum.* In all of these species, the presence of the *tnp2*B fragment was also revealed based on the Illumina data. Moreover, we confirmed the association of *tnp*2B and CficCl-61-40 satDNA that was predicted in silico for *C. album* s. str.*, C. strictum* and *C. striatiforme* (Fig. 2a). The remainder of the analyzed species showed negative results regarding the presence of a *tnp*2B and CficCl-61-40 satDNA association according to both PCR analysis (Fig. 2a) and the in silico screening of the Illumina data.

Another important question is whether the monomers of the CficCl-61-40 satDNA family form fairly long arrays in the genome or could be an artifact of the assembly process. To address this issue, we applied the fiber-FISH (fluorescent in situ hybridization) technique, in which fluorescent probes of CficCl-61-40 satDNA family monomers were hybridized directly to isolated DNA strands to detect a possible row of signals. The fiber-FISH experiments revealed long chains of signals (Fig. 2b.1), thus confirming the array structure of the CficCl-61-40 satDNA family.

One additional piece of evidence for *tnp2B* and CficCl-61-40 satDNA family array associations and the existence of long CficCl-61-40 satDNA family arrays was provided by an alternative assembly-free approach for the analysis of ultralong Oxford Nanopore (ON) reads. In Fig. 2c, self-to-self comparisons of several ultralong ON reads from the *C. acuminatum* genome (Additional file 4) displayed as dot plots (YASS program output) are shown. Parallel lines indicate tandem repeats in which the distance between the diagonals is equal to the lengths of the motifs. This type of analysis confirmed both the *tnp2*B-satDNA association and the complicated structure of CficCl-61-40 satDNA family arrays [18]. ON sequencing also allowed the determination of the real length of the CficCl-61-40 satDNA family array that follows the *tnp2*B fragment. For ON read 17, the array consisted of ~ 125 tandemly arranged monomers (5.0 kb); for read 131, it was ~ 48 monomers (1.9 kb); and for read 313, it was ~ 105 monomers (4.2 kb) (Fig. 2c).

### Concluding remarks

Recently, we described the evolution of the CficCl-61-40 satDNA family in the genomes of several diploid *Chenopodium* species [18]. This family is a major component of the *Chenopodium* satellitome with a pan-chromosomal distribution (Fig. 2b.2). It has been hypothesized that this family of minisatellites could have arisen in the ancestral forms of *Chenopodioideae* and then been transmitted vertically during evolution, with subsequent divergence of both the nucleotide composition and length of the basic monomer. Definitely, this type of CficCl-61-40 satDNA family evolution exists in the *Chenopodium* genomes, and several tandem repeats have diverged significantly. For example, clone 12-13p from the genomes of *C. quinoa* (GenBank: HM641822.1) did not show similarity to any existing GenBank sequences at the time when it was discovered [24]. However, with the subsequent arrival of new data [18], similarities were found between clone 12-13p and the recently identified HOR unit from the *C. acuminatum* genome, which belongs to the CficCl-61-40 satDNA family (GenBank: MH257681.1 - MH257684.1). Thus, the 12-13p repeat could be attributed to a distantly diverged derivative of the CficCl-61-40 satDNA family. Now, it can be argued that along with vertical transmission, in at least some of the studied genomes, there is an ongoing process of CficCl-61-40 satDNA family monomer formation from the TPases of putative CACTA-like elements. This phenomenon is similar to that found in *Arabidopsis* by Kapitonov and Jurka [11], in which one of the *En/Spm*-like transposons (*Atenspm*) generates satellite arrays, and it is the internal region of the *En/Spm*-like transposon (as in our case) that is specifically involved in array formation. Therefore, the pool of CficCl-61-40 satDNA family sequences is continuously replenished. The specific mechanism of CficCl-61-40 satDNA family array formation from the fragment of *tnp2*B requires further investigation but is probably associated with CACTA-like transposon activity in the genome. TE proliferation is a dynamic process that may occur repeatedly over short evolutionary timescales. The active autonomous (intact) DNA elements in *trans* may influence non-autonomous (deletion derivatives) elements as long as they retain the *cis*-acting target sequences [25], and CACTA deletion derivatives may often generate regions of tandem repeated DNA such as the *Afa* and *TM-2* repeats in *Triticeae* [26]. Thus, there are two prerequisites for new CficCl-61-40 satDNA family monomer formation: (i) the presence of an active complete CACTA-like element and (ii) the presence of deletion derivatives of the *tnp2*B type. High similarity between parental *tnp2*B fragments and related satDNA monomers and their interposition in the genome may indicate that CACTA-like transposons have recently been active in the genomes of several *C. album* aggregate species. Thus, two pools of the CficCl-61-40 satDNA family could coexist: an ancestral pool and a newly formed pool.

What might be the consequences of the continuous generation of novel repeats of a certain type in the genome? The most salient point is that the continuous replenishment of genomes with new identical (because of *tnp2* domains high conservation) CficCl-61-40 satDNA family monomers may lead to an increase in satellitome similarity between species, which may in turn increase the possibility of interspecific hybridization [27]. The second important point is that in the genomes of several *Chenopodium* species, CACTA-like elements could be transpositionally active, with associated consequences [2, 28]. CACTA-like elements can cycle between an active and an inactive state [29]. At one extreme, an element may change frequently between states during plant development, whereas at the other extreme, the inactive state is stably inherited through many plant generations, and reactivation is not much more frequent than spontaneous mutation [2]. Thus, the question of which *Chenopodium* lineages still show activity of CACTA-like elements remains to be answered, which will significantly contribute to understanding *Chenopodium* genome evolution.

## Materials and methods

### Plant material, DNA extraction, library preparation and Illumina sequencing

For the preparation of the DNA libraries, plants of the following species were used: *C. acerifolium C. acuminatum, C. album* s. str.*, C. bryoniifolium, C. ficifolium, C. iljinii, C. jenissejense, C. karoi, C. luteorubrum, C. novopokrovskyanum, C. opulifolium, C. pamiricum, C. sosnowskyi, C. striatiforme, C. strictum, C. suecicum,* and *C. vulvaria* (Table 1). DNA was extracted from fresh leaves using the DNeasy Plant Mini Kit (Qiagen) according to the manufacturer’s instructions. For in situ hybridization experiments, the tips of the young, fine roots of *C. album* s. str. Were collected and fixed as described previously [15], and then stored until use. For all analyzed accessions, the DNA ploidy level was checked by flow cytometry as described previously [30]. One individual per species was used for library preparation and NGS. The details of library preparation and Illumina sequencing were as described in Belyayev et al. [18]. The Illumina data have been deposited in the NCBI Sequence Read Archive as BioProject PRJNA634444.

### Genome assembly, the search for repeatome elements and data processing

For the processing of Illumina NGS data and the identification of the colocalization of TEs and CficCl-61-40 satDNA family arrays in the genomes of all investigated species, Geneious Prime software version 2019.2.1 (https://www.geneious.com) was used [19]. The advantage of this assembler is that it produces large contigs. De novo assembly was carried out with medium-low sensitivity, which is the best option for large numbers (e.g., 100,000 or more) of Illumina sequencing reads. The main satellite was identified using a short conserved motif as a query. This motif, generated on the basis of our previous research [18], was TTTCATTTGA. In contigs with CficCl-61-40 satDNA family arrays, a search for TEs was carried out using BLAST searches for conserved domains (https://www.ncbi.nlm.nih.gov/Structure/cdd/wrpsb.cgi) [31]. TRF (https://tandem.bu.edu/trf/trf.html) analysis [32] allowed the determination of the consensus monomers of the CficCl-61-40 satDNA family. The described algorithms were applied to the genomes of each species separately.

Genomes were scanned for the presence of the *tnp2*A and *tnp2*B TPases in individual contigs with the aid of the conserved motifs. The *tnp2*A TATAACTTGCCTCCTT motif was first detected in the genomes of *C. acuminatum* and *C. vulvaria*. The position of the motif covered the region from 159 to 175 bp of the ~ 630 bp *tnp2* domain. For *tnp2*B, the conserved GGCTGGGTTACC motif was detected 20 bp upstream from the beginning of the *tnp2*B fragment parental to the CficCl-61-40 satDNA family array. The latter motif was missing in the full domains. Scanning was performed by using the “search for motifs” command of Geneious software with a one-nucleotide maximum mismatch for *tnp2*A and a two-nucleotide maximum mismatch for *tnp2*B.

For the reconstruction of phylogenetic relationships among the analyzed monomers and TPases, multiple alignments were performed with ClustalW [33]. The phylogenetic relationships among the sequences were then reconstructed from the pairwise distance matrix [34]. The distance matrix thus obtained could be used to construct a phylogenetic tree via the minimum evolution method. The construction of the phylogenetic tree was performed in the MEGA program (Fig. 1b, c) [35]. The sequence logo was produced using the publicly available online tool: https://weblogo.berkeley.edu/logo.cgi [36].

### PCR screening for the association of tnp2B with *CficCl-61-40* satDNA family arrays

Primer pairs were designed for the PCR screening of the association of tnp2B with CficCl-61-40 satDNA family arrays, in which the 5′ primer was an outward-facing *tnp2*B-specific primer based on the conserved GGCTGGGTTACC motif, and the 3′ primer corresponded to the beginning of the CficCl-61-40 satDNA family array (contig 22 of *C. acuminatum*) (Fig. 1a). If an association of *tnp2*B with CficCl-61-40 satDNA family arrays physically exists in the genome, we would expect to obtain a combined fragment of approximately 400 bp consisting of sequences from both the *tnp2*B sequence and the CficCl-61-40 satDNA family array. If such an association does not exist and the suggested association is an artifact of the assembly process, no PCR product will be observed. The oligonucleotide sequences of the PCR primers were as follows: 5′ *tnp2*B-specific primer, GGCTGGGTTACCGACTTACA; 3′ CficCl-61-40 satDNA array-specific primer, TCAAACATGTACATCCAGCCA. As a template, we used the total DNA of all investigated species (Fig. 2a). PCR was performed in 25 μl reactions containing 1x TopBio Plain PP Master Mix (TopBio), each primer at 0.2 mM and 5 ng of genomic DNA. The cycling conditions were as follows: 5 min at 95 °C, followed by 35 cycles of 95 °C for 30 s, the sequence-specific annealing temperature (55 °C) for 30 s and 72 °C for 1.5 min, and a final extension at 72 °C for 15 min. The PCR results were verified in a 1% agarose gel. The PCR products of clusters were excised from the gels, cloned and sequenced at Eurofins Genomics (Konstanz, Germany) according to standard protocols.

### FISH procedure

To detect the chromosomal distribution of the CficCl-61-40 satDNA family in the chromosomes of *C. album* s. str., FISH was performed. Root tips were pretreated in 0.002 M 8-hydroxyquinolin for 3 h in the dark and fixed in 3:1 (v/v) 100% ethanol:acetic acid. The fixed root meristems were thoroughly washed in water and enzyme buffer (10 mM citrate buffer at pH 4.6) and partially digested in 0,3% (w/v) cytohelicase, pectolyase and cellulase (Sigma, St. Louis, MS, USA) at 37 °C for 3 h, followed by several washes in water. The material in a water drop was carefully transferred to a grease-free microscope slide, and the cells were spread as previously described [18]. Fiber-FISH slides were prepared from the total DNA of *C. acuminatum* according to the technique described by Schwarzacher and Heslop-Harrison [37].

FISH experiments were performed with the CficCl-61-40 X-1 clone [18] as a probe, which was labeled with Cy3 (Amersham, Amersham, UK) according to a standard oligo labeling protocol. FISH was performed in a ThermoBrite programmable temperature-controlled slide processing system at 63 °C for 3 h. The slides were stained with DAPI, mounted in antifade mountant (Vector Laboratories, Peterborough, UK) and examined and photographed on a Zeiss Axio Imager.Z2 microscope system.

### Oxford Nanopore sequencing and ultralong read analysis for the association of tnp2B and the *CficCl-61-40* satDNA family

For Oxford Nanopore sequencing, the DNA of *C. acuminatum* was used. The DNA was fragmented by pipetting. The sequencing libraries were prepared from 1 μg of the partially fragmented DNA using an SQK-LSK109 Ligation Sequencing Kit (Oxford Nanopore Technologies) following the manufacturer’s protocol. The DNA was treated with 2 μl of NEBNext FFPE DNA Repair Mix and 3 μl of NEBNext Ultra II End-prep enzyme mix in a 60 μl volume that also included 3.5 μl of FFPE and 3.5 μl of End-prep reaction buffers (New England Biolabs). The reaction was performed at 20 °C for 5 min and 65 °C for 5 min, followed by purification using a 1x volume of AMPure XP beads (Beckman Coulter). Subsequent steps, including adapter ligation using NEBNext Quick T4 DNA Ligase and library preparation for sequencing, were performed according to the provided protocols. The whole library was loaded into the FLO-MIN106 R9.4 flow cell and sequenced for 20 h.

A search for *tnp2B* was performed with the conserved GGCTGGGTTACC motif among the longest (> 30 kbp) ON reads of the *C. acuminatum* genome. For the analysis of selected ultralong ON reads, the YASS genomic similarity tool was used, which enables searches of tandem repeat organization (http://bioinfo.lifl.fr/yass/yass.php) [18, 38].

## Supplementary information

**Additional file 1: **Contig showing the association of *tnp2*B and CficCl-61-40 satDNA family arrays.

**Additional file 2: **1. Consensus monomers of the CficCl-61-40 satDNA family and corresponding fragments of *tnp2* for species of the *C. album* aggregate. 2. TPase domains of putative CACTA-like transposons detected in the genomes of the species of the *C. album* aggregate in comparison with similar domains of other species.

**Additional file 3: **Characteristics of the putative CACTA element *Jozin* from the genomes of *C. pamiricum* and *C. sosnowskyi.*

**Additional file 4: **Three ON reads from the *C. acuminatum* genome with association of *tnp2*B and CficCl-61-40 satDNA family arrays.

## Data Availability

Please contact author for data requests for the datasets used and/or analyzed during the current study including the original fastq sequencing files. All annotations files and generated output data sets corresponding to number of contigs and/or reads mapping putative CACTA-like element *Jozin* and CficCl-61-40 satDNA family arrays are included in this published article (and its supplementary information files).

## Abbreviations

DUF: Domain of unknown function
FISH: Fluorescent in situ hybridization
HOR: High order repeats
NGS: Next-generation sequencing
ON: Oxford Nanopore sequencing
satDNA: Satellite DNA
TAD: Transposase associated domain
TEs: Transposable elements
TPase: Transposase
TRF: Tandem repeats finder.
