# Supplementary material for: Transposons and satellite DNA: on the origin of the major satellite DNA family in the Chenopodium genome
Source: Mob DNA. 2020 Jun 26;11:20. doi: 10.1186/s13100-020-00219-7 (PMC7320549; doi:10.1186/s13100-020-00219-7)

**Additional file 1.** Contig with an association of tnp2B and CficCl-61-40 satDNA family arrays

Conserved motif of *tnp2*B highlighted blue. Beginning of parental fragment of *tnp2*B highlighted green. Conservative motif of CficCl-61-40 satDNA family monomer highlighted yellow (beginning of arrays).

*C. acuminatum*

> contig 22

GACCTCTTATTGAAGAGTTAAAGCAATTATGGGAGGCTGGGTTACCGACTTACAACATCTCGCAAAAACAAAATTTTCATTTGACTCAATTAGCTTTGTATAATGTGTTTGACTTTCATTTGACTCAATTAGCTTTGTTAATTACATTTAACTTTCATTTAATTCAACAAGCTTTGTTGAATGTGTTTGACTTTTATTTGACTCAATTACCTTTGTTGAATGCATTTTACTTTCATTTGATTCAAAAAGCTTCGTTTGAATGCGTTTGAATTTCATTTGACTCAATTAGCTTAGTTGAATGCATTTGACTTTCATTTGATTCAAAAAGCTTTGTATGAATATGTTTTACTTTCATTAGACTCAATTATCTTTCTTGAATGCATTTGTCTTTCATTTGATTCAAAAACCATTGTTTGAATGTGTTTGACTTTCATTTGACTCAATTAGCTTTGTTGAATGCATTTTACTTTCATTTGATACAAAAAGCTTTCTTTCAATGTGTTTGACTTTCATTTTACTCAATTAGCTTTGTTGAATGCATTTGACTTTAGTTTGATTCAAAAAGCATTGTTTGAATGTGTTTGACTTTCATTTGACTCAATTAGCTTTGTAGACTGCATTTGACTTTCATTTAACTCAATTAGCTTTGTATGAATGTGTTTGACATTCATTTGACTCAATTAGCTTTGTTAATTGCATTTCACTTTCATTTAATTCAAAAAGCTTTGTTTGAATGTGTTTCACTTTCATTTGATTGAAAAAGCTTTGTTTGAATGTGTTTGACTTTCATTTGACTCAATTACCTTTGTTGAATGCATTTGACTTTCATTTGATTCAAAAAGTATTGTTTGAATGTGTTTGACTTTCAATTGACTCAATTAGCTTTGTTGAATGCATTAGACTTTCATTTGATATAAAACGCTTTGTTTGAATGTGTTTTAATTTCATTTGACTCAATTATTTTTGTTGAACGCATTTGACTTTCATTTGTTTCAAAAAGCTTTGTATGTGTGTGTTTGACTTTAATTTGACACAATTATCTTTATTGAATGCATTTGACTTTTATTTGATTCAAAACGCTTTGTTTGAAGGTGCTTGACTTTCATTTGAGTCAAAAATCATTGTTGAATGCATTTGACTTTTATTTGATTCAAAAAGCTTTGATTGAATGTGTTGGACTTTCATTTGACTCAATTAGCTTTGTTAGAATGTGTATGACTTTCATATTACTTAATTATGTTTGTTGAATGCATTTGACTTTCATTTGATTCAAAAAGCTTTGTTTGAAGGTGTTGACTTTCATTTGAGTTATAAAGCATTGTTGAATGCACTTGACTTTCGTTTAATATAAAAAGCTTTGTTTGTATGTGTTTGACTTTCATTTGAGTCAATTAGCTTTGTTCAATGCATTTGACTTTCATTTGATTCAAAAAGCTTTGTTTGAATGTGTTTGACTTTCATTTGACTCAATTATCCTTTTTGAATGCAATTGACTTTCAATTGATTCAAAAAGCTTTGTATGAATGTGTTTGACTTTCATTTGACTCAATTAGCTTCGTTGAATGCATTTGACTTTCATTTGATTTAAAAAGCTTTGTTTGATTGTGTTTGACTTTCATTTGGTTCAAAAAGCTTTGTTGAATGTGTTTGATTTTCATTTTACTCAATTAGCTTTGTTGAATGCATTTGACTTTCATTTGATTTTAAAACCTTTGTTTAAATGTGTTTGACTTTCATTTGGTTCAACAAGCTTTGTTGAATGAGTTTGACTTTCATTTTACTCAATTAGATTTGTTGAATGCATTTGACTTTCATTTGATTAAAAAAGCATTGTTTGAATGTGTTAGACTTTATTTAACTCAATAAACTTTGTTGAATGCATTTGACTTTCATTTGATTTTAAAAGCTTTGTTTGAATGTGTTTTGACTTTCATTTGACTCAATTAGCTTTGTTAATTGCATTTCACTTTCATTTAATTCAAAAAGCTTTGTTTGAATGTGTTTTACTTTTATTTGACTCAATTAGCTTTGTTGAATGCATTTTACTTTCATTTGATTAAAAAAGCTTTGTATGAATGTGTTTGACTTTCATTTGACTCAATTATCTTCGTTGAATGCATTTGACTTTCACTTGATTTTAAAAGCTCTGTTTGAATGTGTTTGACTTTCATTTGGTTCAAAAAGCTTTGTTGAATGAGTTTGTCTTTCATTTTACTCAATTTAGCTTTGTTGAATGCATTTGACTTTCATTTGATTAAAAAAGCATTGTTTGAATGTGTTTGACTTTCATTTGACTTAATTAACTATGATGAATGCATTTATCTTTGATTTGATTCAAAAAGCTTTGTTTGAAAGTGTTTGACTTTCATTTGACTCAATTACCTTTATTGAATGCATTTGACTTTCATTTGATTCCAAAAGCTTTGTATGAATGTGTTTGACTTTCATTTGACTCAATTATCCTTGTTGAATACATTTGACTTTCATTTGATTTAAAAAGCTTTGTTTGAACGTGTTTGACTTTTATTTGAGTTAAAAATCTTTGTTGAATGCATTTGACTTTATTTGATTCAAGAACCTTTGTTTGAATGAGTTTGACTTTCATTTGACTTAATTATCTTTGTTGAATGCATTTGACTTTCATTTGGTTCAAAAAGCGTTGTTTAATGTGTTTGACTTTCATTTGACTCAATTAGCTTGGTTGAATGCATTTGAGTTTTATTTGATTCAAAAAGCTTTGTATGAATGTGTTTGACTTTTATTTGACCCAATTACCTTTGTTGAATGCATTTGTCTTTCATTTGATTCAATATATCTCTATTTTGTTGCTTTTGAATACAAGTTGTTTTACTAGTTGTTGCTAGATTTCCTCAAATTTTGTCTTGTTTTCATATGTCAACAAATTCTATCAACTAATTTTTTATTTGTATCGATTTAGAAACAAATTGAGGCAACAAATTTTTGAATTGTTCCTATTAGAAATTAGCCGTCTTACTAGTTGTTGCATTTTACATTCATTTGATTCAAAAAGCTTTGTTGAAAGTATTTGACTTTCATTTGACTCAATAAGCTTTGTTGAATGCATTTGACTTTCATTTGATTCAAAGAGCTTTGTTTGAATGTGTTTGACTTTCATTTGAGTCAAAAAGCTTTGTTGAATGCATTTGACTTTCATTAGATTCAAAAAGCTTTGTTTGAATGTGTTTGACTTTCATTTGAGTTAAAAAGATATGTTGAATGCATTAGACTTCCATTTGATTCAAAAAGCTTGGTTTAAATGTGTTTGACTTTCATTTGACTCAATTATCTTTGTTGAATGCATTTGACTTTCATTTGATTCAAAAAGCTTTGTTTGAATGTGTTTGACTTTCATTTGACTCAATTGTCTTTGTTGAATGCATTTGACTTTCATTTGATTTAGAAAGCGTTGTTGAATGTGTTTGACTATCATTTGACTCAATTAGCTTTGTTGAATGCATTTAAGTTTTATTTGATTCAAAAAGCTTTGTATCAATGTGTTTGACTTTCATTTGACCCAATTACCTTTGTTGAATGCATTTGACTTTCATTTGATTCAAAAAGCTTTGTTTGAATGTGTTTGACTTTCATTTGACTCAATTGTCTTTGTTGAATGCATTTGACTTTCATTTGATTTAAAAAGCGTTGTTGAATGTGTTTGACTTTCATTTGACTCAATTAGCTTTGTTGAATGTATTTAAGTTTTATTTGATTCAAAAAGCTTTGTATCAATGTGTTTGACTTTCATTTGACCCAATTACCTTTGTTGAATGCATTTGACTTTCATTTGATTCAATATATCTCTTTTTTGTTGCTATTGAAAACAAGTTGTCTTACTAGTTGTTGCTAAATTTCATCAATTTTGTCTCGTTTCCATATATCAACAAATTCTGACAACTAATTTTTTATTTGTAGCGATTTAGAATCAAATTGAGGCACTAAATTTTTGAGTTGTTCCTATTAGAAATTAGCCGTCTCGTTGTTGTTGCTAGATGACAACAGATTTTTTATTTGTTGTCATTTAGCAATGCCATTTTACAAAACGTTTGGCAACAAAATTTCTAGTTGTTGCTATTAGGGACACTTGTTTTACTATTTCTTGCTAAAATGACATCACATTTTAATTTGTTGCCATTAGAAGAAAGTTGTGTTATAAATTGTTGCTAATTTATTAAGAGTCATTGAAGTAATTTACATAATATGTTTTAATAAATAAAAACGAAAACAAGTCATCTTGTTCTATACAAAAGAAATATTAGAAGGAATTAATAATCGTTCAATCACTTTTAGCAATAAAACGAAGGCGATAAAATAATATATATTATTTTATAAGTTCATTAGAAAGAGCATGTATAAAATGGGAATGCTTCTTACTATTATGGCCGAATTTGCTTGATTTTGGTTCTACAAGACCTTAAAACCTTATAACATGCTATGTTTTTGTGATTTCTTTTCTTAGGAAGTTATAAATACATATGTTTGACATGAATCAAGCCTTTTTAAGTTCATTAGAAAGAGCATGTAGAAAATGGGAACGCTTCTTCCCATTATGGCCGAATTCGCTTGATTTCTGTTCTAATAGACCTTAAAACCTTATACCTTGCTTTGTCTTAGTGATTTCTATGCTTAGCCAGTTACTAATACATATATTTGACATCATTCAAGCCTTTATAATTTCATTAGAAAGATGAAAGCAGGAAATGGGAACGCTTTTTATCATTTAGGCCGAATTTGGTTGATTTTGGTTCTACAAGACCTTAAAACCTTATAACATCCTTGTTTTTGTGATTTCTTTTTTTAGGAAGTTATAAATATATATATTTGACATGAATCAAGCCTTTTCAAGTTCATTAGAAAAAGCATGTAAAATATGGGTGCTTCTTCCTATTTTGCTCGAATTTGTTTGATTTCGGTTCTACCAGTCCTTAAAATCTTATAACTTGCTTTGTTTTTGTTATTTCTATGATTAGGAATTATAAATACATATATTTAACATTATTCAAGTTTTTTGTAGTTCATTAGAAAGAGCATGTAGAAAATGAGAATGCTTCTTTCTATTTTGCCTGAATTTGCTTGATTTTGGTTCTACAAAACCTTAAAACCTTATAATTTGCTTTGGTTATTGTCATTTCTTTGGTTAGGAAGTTATAAATACATATATTTGACATGAATCAAGCCTTTTCAAGTTCATTAGAAAGAGCATGTGGAAAATGGGTACGCTTTTTCCCATTTTGCCCAAATTTGCTTGATTTAGGCTCTACAACACCTTAAAACCTTATAACTTGCTTTTTTTTTTGTGATTTATTTGCTTAGAAAATTATAAATACATATATTTTATATGATTCAAGAATTTATAAATTCATTAGAAAGAGCATGTAGAAAATGGAAAGGCTTCTTACTATTTTGCCCGAATTTGCATGATTTTTGTTCTACTAGACCTTAAAACCTTATATCTTGCTTTGTTTTTGTGATTTATATTGTTAGGAAGTTATCAAGTTTATTAAAAAGAGCATGTAGAAAAAGGGAATGCTTCTTACTATTTTGCCCGAATTTGATTGATTTCAGTTCTACAAGACCTTAAAACCTTATAAATTGCTTAGTTTTTGCGATTTCTATGCTTATGCATTTATAAATACATATATTTCACATGATTCAAACAATTATAAGTTCATAAGAAAGAGCATGTAGAATTTGGGAATGCTTCTTACAATTTTACCCAAATTTGATTGATTTTGGTTCTACAAGACCTTAAAACCTCATAACTTGATTTGTATTTGTTATTTATTTTTTAATGGAGTTATAAATACATATATTTGACATGATCCAAGCATTTATAAGCTCATTAGAAAGAGCATGCAGAAAATGGGAACGTTTCTTTCCATTTTGCCCGAATTTGCTTGATTTTGGTTCTACAAGACCTTAAAACCATATAACTTGCTTTTTCTTTGTTATTTATTTGCTTAGGCAGTTATAAATATGTATATTTGACATGATTCAAGAATTTATAATTTCATTAGAAAGAGCATGTAGAAAATGGGAATGCTTCTTACTATTTTGCACGAATTTGCTTGATTTTGGTTCTACAAGACCTTATAACCTTATGAATTGATTTCTTTTTGTGATTTTTTTTGTGAGGCAGTTATAACTACATATATTTGACATTACTCAAGCCTTTTCAAATTCATTAGAAAGAGCATGTAAAAAATGGGAATGCTTCTTCCCATTTTGGCCGTATTTGCTTAATTTTGGCTCTACAAGACCTTAAAACCTTATGACATGCGTTATTTTTGTGATTTATTTTCTTAGGAAGTTGTAAATACATATATTTTACATGAATCAAGCCTTTTCAAGTTCATTAGAAAGAGCATGTAGAAAATGGGAACGCTTCTTCTCATTTTGCCCGAATTTGCTTGATTTCGGTTCTACCAGACCTTAAAACCTTATAACTTGCTTGGTCTTAGTGATTTCTATGCTTAGTCAGTTACAAATACATATATTTGACATTATTCAAGCCTTTATCAGTTCATTAGAAAGAGCATGTAGAAAATGGGAACGCTTCTTCCCATTTGGACCGAATTTGCTTGATTTTGGTTCTACAAGACCTTAAAACCTTATAAAATGCTTTGTTTTTGTGATTTCTTTTCTTAGGAAGTTATAAATACATATATTTGACATGAATCAAGCCTTTTCAAGTTCATTAGAAAGAGCATGTAGAAATTGGGAACGCTTCTTCCCATTATGCCCGAATTTGCTTGATTTCGGTTCTACTAGACCTTAAAACCTTATACCTTGCTTTGTCTTAGTGATTTCTATGCTTAGGCAGTTACTAATACATATATTTGACATAATTCAAGCCTTTATAAGTTCATTAGAAAGAGCATGTAGAAAATGGGAACGCTTTTTACCATTTGGGCCGAATTTGGTTGATTTTGGTTCTACAAGACTTTAAAACCTTATAACATCTTTTGTTTTTGTGATTTCTTTTGTTAGGAAATTATAAATACATATATTTGACATGAATAAAGCCTTTTCAAGTTCATTAGAAAGAGCATGTAAAAAATGGGTGCTTTTTCCTATTTTGCTCGAATTTGCTTGGTTTCGGTTCTACCAGTCCTTAAAACCTTATAACTTGCTTTATTTTTGTTATATCTGTGATTAGGAATTATAAATACATATATTTAACATTATTCAAGTTTTTTGTAGTTCATTAGAAAGAGCATGTAGAAAATGAGAATGCTTCTTTCTATTTTGCCTGAATTTGCTTGATTTTGGTTCTACAAAACCTTAAAACCTTATAACTTGCTTTGGTTATTGTCATTTCTTTGGTTAGGCAGTTATAAATACATGTATTTGACATGAATCAAGCCTTTTAAAGGTCATTAGAAAGAGCATGTGGAAAATGGGTACGTTTTTTCCCATTTTGCCCAAATTTGCTTGATTTAGGCTCCACAACACCTTAAAACCTTATAACATGTTTTTTTTTGTGATTTACTTGCTTAGAAAATAATAAATGCATATATTTTATATGATTCAAGAATTTATAAGTTCATTAGAAAGAGCATGTAGAAAATGGAAAGGCTTCTTTATTATTTTGCCCGAATTTGCTTGATTTTGGTTCTACAAGACCTTAAAACCTTATAACTTGCTTTTTTTTGTGATTTATTTTGTTAGGAATTATAAATTCATATATTTGACATGAATCAAGCCTTTTCAAGTTTATTAAAAAGAGCATGTAGAAAAAGGGAATGCTTCTTACTAGTTTGTCCGAATTTGCTTGATTTCAGTTCTACAAGACCTTAAAACCTTATAACTTGCTTTCTTTTTGTGATTTCTATGCTTATGCATTTATAAATACATATATTTCACATGATTCAAACAATTATAAGTTCATTAGAAAGAGCATGTAGAATTTGGGAATGCTTCTTACTATTTTGCCCGAATTTGCTTGATTTTGGTTCTACAAGACCTTAAAACCTTAATACATGCTTTGTTTTTGTGATTTATTTTCTTACACAGTTATGAATACATATATTTTAAGTGAATCAATCCTTTTCAAGTTCATTAGGATGAACGTATAGAAAATGGGAACGCTTCTTCTCATTTTCCCTGAATTTGCTTGATTCGGATTCTACCAGACCTTAAAACCTTATAAATTGTTTTATCTTTGTTATTTATTTGCTTAGGCAGTTACAAATACATATATCTTATATGATTCAAGCCTTTATAATTTCATTAGAAGGAGCATGTAGAAAACGGGAACGCTTCTTCCCATTTTGGCCGAATTTGCTTGATTTTGGTTCTACAACACCTTTAAACCCTATAACATACTTTTTTTGTGATTCTTTTCTTAGGAAGTTATAAATACATATATTAGACATGAATCAAACCTTTTCAAGTTCATTAGAAAGAGTGTGTTGAAAATAGGAACGCTTCTCATTTTGCCTTAATTTGCTTGATTTCGGTTCTACCAGACCTTAAAACCTTATAACTTGCTTTTTTTTTGTGATTTCTATGCTTAGGAATTTATAAAAAAATATATTTGACATGACTCAAGCATTTATAAGTTTTTTAGAAAGAGCATGTAGAAAATGGGTACGCTTCTTACCATTTTTCCCGAAAATGCTTGATTTTGGTTCTACAAGACCTTAATACCTTATAACATCCTTTTTTTTGTGATTCATTTTGTTATGAAGTTATAAATACATATATTTGACATGATTCAAGCCTTTTCATGTTCATTAGAAAGAGCATGTCGAAAATGGGAATGCTTCTTCCCATGTTGCTCGAATTTGCTTGATTCGGTTCTACAAGACCATAAAACCTTATAACTTGCTTTTTTTTATTTCTATTCTTAGGCAGTTATAAATTCATATATTTAACATGATTCAAGCATTTATATGTTCATTAGAAAGTGTATGTAGAAAATGGGAATGCTTCTTACTATTTTGCCCGAATATGCTTGATTTTGGTACTACAAGACCTTAAATTCTTATAACATCCTTTTTTTGTGATTTCTTTTTTTAGGAAGTTATGAATACATATATTTGACATAAATAAAGCCTTTTCAAGTTCATTAGAAAGAGCATGTAGAAAATGGGAACGCTTCTTCTCATTTTGCTCGAATTCGGTTGATTTCGGTTCTACCGGACCTTAAAACGTTATAACTTACTTTGTCTTAGTGCTTTCTATGCTTAGGGAGTTACTAATGATATATATTTAACATGATTCAAGCCTTTATAAGTTCATTAGAAAGAGCATGTAGAAAATAGGAACGCTTCTTCCCATTTGGGCCGAATTTGGTTAATTTTGGTTCTACAAGACCTTTAAACCTTATAAAATCCATTGTTTTTGAGATTTATTTTGTTGGGAAGTTATAAATACATATATTTGACATGACTCAAGCCTATTAAAGTTCATTAGTAAGTGCATGTAGAAAATGAGAACGCTTCTTCTCATTTTGCCTTAATTTGCTTGATTTCGGTTCTACCAGACCTTAAAAACTTATAACTTGCTCTGTTTTTGTGATTTCTATGCTTAGGAATTTACAAAAAAATATATTTGACAAGACTCAAGCATTTATAAGTTTATTAAAAAGAGCATGTAGAAAATGGGTACGCTTCTTAGCATTTTGCCCGAAAATGCTTGATTTTGGTTCTACAAGACCTTAAAACCTTATAACATCTTTTTTTTTGTGATTCATTTTGTTATGAAGTTATAAATACGTATATTTGACATGACTCAGGCCTTTTCATGTTCATTAGAAAGAGCATGTCGAAAATGGGAATGCTTCTTCCCATGTTGCTCGAATTTGCTTGATTCGGTTCTACAAGACCATAAAACCTTATAACTTGCTTTTTTTTTATTTCTATTCTTAGGCAGTTATAAATCCATATATTTGACATGATTCAAGCATTTATAAGTTCATTAGAAATAGTATGTAGAAAATGGGAATGCTTCTTACTATTTTGCCCGAATATGCTTGATTTTGGTACTACAAGACCTTAAAACCTTATAACATCCTTTTTTGTGATTTATTTTTTTAGGAAGTTATGAATACATATATTTGACATAAATCAAGCCTTTTCAAGTTCATTAGAAAGAGCATGTAGAAAATGGGAACGCTTCTTCTCATTTTGCCCGAATTCGCTTGATTTCGGTTCTACCGGACCTTAAAACCTTATAACTTGCTTTGTCTTAGTGATTTCTATGCTAAGGGAGTTACTAATACATATATGTAACATGATTCAAGCCTTTATAAGTTCATTAGAAAGAGCATGTAGAAAATGGGAACGCTTCTTCCCATTTGGGCCGAATTTGGTTGATTTTGGTTCTACAAGACCTTTAAACCTTATAAAATCCATTGTTTTTGTGATTTCTTTTTTTAGGAAGTTATAAATACATATATTTGACATGACTCAAGCCTTTTCAAGTTCATTAGAACGTACATGTTGAAAATGAGAACGCTTCTTCTCATTTTGCCTGAATTTGCTTGATTTCGGTTCTATCAGACCTTAAAACCTTATTAATTGCTTTCTCTTTGTTATTTATTTTCTTAGGAAGTTACAAATACATATATTTGACATGATTCAAGCCTTTATAAGTTCATTAGAAGGAGCATGTAGAAAATAGGAACGCTTCTTCCCATTTTGGCCGAATTTGCTTGATTTTGGTTCTACAAGACCTTAAAACTTTATAACATGCATTTTTTGTGATTTCTTTTCTTAGGAATTTATAAATACATATATTAGACATGAATCAAGCCTTTTCAAGTTCATTAGAAATAGCGTGTAGAAAATGGGAACGCTTCTTCTCATATGATGTTAATTGGTAACAATAATTTTCATTTTGATGACCTTAAATAGTTAAATGACCTAAAAGAATGTCATTCTTCTAAGTGCTTTACTTAG

| Indices | Period Size | Copy Number | Consensus Size | Percent Matches | Percent Indels | Score | A | C | G | T | Entropy (0-2) |
| --- | --- | --- | --- | --- | --- | --- | --- | --- | --- | --- | --- |
| 75--3816 | 39 | 94.4 | 39 | 74 | 7 | 2616 | 25 | 12 | 15 | 46 | 1.81 |

Consensus pattern (39 bp):

TTTCATTTGATTCAATAAGCTTTGTTGAATGTGTTTGAC


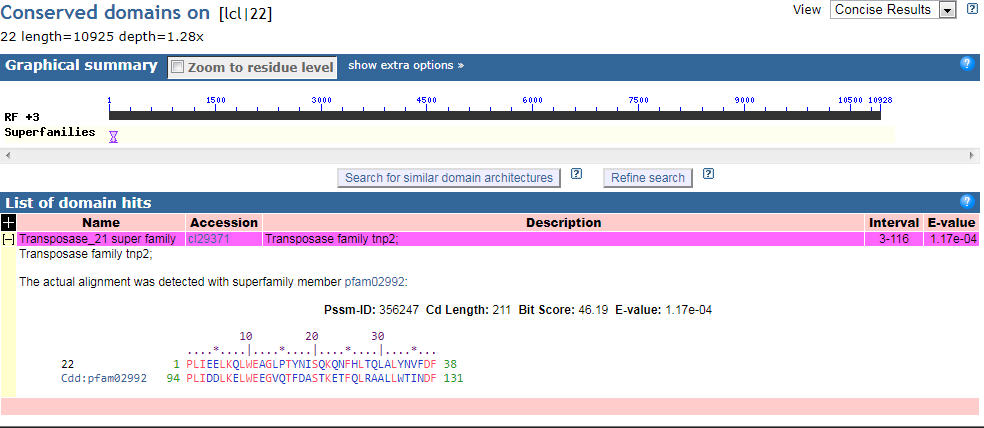


>contig 150

ATTTCAGGTTAACATAACCTTATTTAAACCCAAATAAAAATTCAGGTCAAATAAGGTCATTTTCAGGTTGACCCGAACCTGAAAAAATCAGGTTCAGGTTGAGGTTTTCAACCCGAAATGTTAAACGGATCAGGTTCAGGTCAGGGGGTAGCTGACCCGAAATCAGCTGACCCGAACACGGACCGGACCCGACCCGACCTGATCCGATTGCAAGCCCTACCGAGGATGCATTTAACCCATATAGAAACATGGTATTAGATGCCTTTGGACTAGAAGGTGGTTTAGAAAATATTGAAGAAGAGCCACATGTTAGTTACAAGAAGTTCTTTGACATGTTGAAGGCAGCAGAAGAGCCATTGTATGGTGGCTGCAAGTTGTCTGTATTGTCTGCAGCTGCAAGGATGGAAAACATCAAGTCCGAGTATAATATTCCGCATAAAGCTATAGATGGTGTTGCTTCCATAATGAAAGATAGTTTTTACAAGACTAAGACATTGCTTAAAGGGCCAGAACTTCCTCACCAAAAGATTCATGTATGTCCAAATGGAT?????????????????????????AGATCTCAAAGAATGTCTATATTGCAAAGGAAGTCGTTATAAGACACTTAGAGAAAGTGGCAACAATTCTCCTCATAGTGCTCTTATTTACTTTCCTGTAGGCCCGAGATTACAAAGATTATATGCAACAAGATCTACTGCGGAGCAGATGAGGTGGCATAAAGATAATCCTCGAGTTCATGGCCTTATGTCTCATCCAAGTGATGAAGAGGCGTGGAAACACCTAGATGAAGAGTACCCTTCTTTTGCTGCGGAGCCTAGTGTAATACCCGAATATTTTATGATTTTATATATAATTTATTTTTATTTTTATTTATTAAATAACTTTCCTAATCTCATATTTTTATTTTAGAGTTCGGAAAGGTTTAAAACCCTATTTTAATGATATTAGGACTCTTATTTAATTTATATAAACTATCTTAAAACCCTAATTATATATTTTATAATGGCCGTTCGTGCGCCTGTTTGTGGCTCTTTGTTTGCTTTTGATCAATTTTCACCTACCCTTGATACTTACATCATCAACCAATTTATAGCTTTGATCTTATTTCCACCCATTGACTATATTATACGTATGCAATTGTGAACCAAATTTCAAGAGACCAATCTCATTAAAGCCTTTCACGCATATGCTATGAATGTAATACCGTCGTCTTGCTTTCTATTTGGTTTGATGAGCAGCATGTCGCTTATTGCTATTTTTCTATCATGAAATTTGGTTTATCGTCTCCCTAGGTACTCACTATATCAATTCTTTGGAGTCGTATGCTTAATTTATATACTTTTGTCTCGTAATCACACGAATCTTATTTCTTGATTTTGTTTGATTATAAATTGTTAGTATTGATTGTACTTCGTATTGTTATACTACGTATATAAATTTTATTATTTGTGAATTATATATAATCATGATTTATATGTATATGGTTAGTGTGTGGTATTGTATGATTTAAATATCGCATTGATTGATTGTGTTATTTCTAGGGTTTTAATGTTGTCGTTTTAAGGAGTATTGTTCTTGAGGTTTTAGGGCTACTAATATTATATTGGGTTAAATTAATTAATTTTGGGTACTAGTATTAGCCATATAAGAGTTGAGATATTTTATGAATTGGGAACTTGTGCCAATATTGAGATATTATTTATTATCATGTGAAAGATTATTTGATCAAGTATGAGATCAAAAGGTTGAGAAATTATTATTTTGTTCTTGGTTTGAGGCTCGGCGTTATAGACCTAGTCCTTGGCATGTTGGAAATGAGATAATGATCATGAGTTAAGAATGAGGTACAATCTTTATATTATTTATTTTTCAATGGTCCCCTACAGTGGGAATTGGCCCGTATGTGGTCTGGTTCAAGGTGTTTTTCTAGTAATTGGGAGTATTCAAGTTTTTATACTTGTTGGTTTGTTATTCGAATTACCCATGTCCAAGGCTAGGTATAGAACAATGAGCATGGCACACTTATGGGTTATATGAGTTGATGATTGAGTAAAGGTTCGTATTATTATTCGAGGTTGAGTTGACCAATGGTTACTTTTCTCAGAGTACAATGTGTAATGTAGTTTGAACTACATTGTTTAATTGAGCTAGTAATGTTGATTATTTTGCTATACTATGTATTCTGATATGTGGTGATTACTCAGCTTTTTGCTGACGTGTGTGTATTTTTGTTGTGCTACTTGTGTTTGCGGCCATGTCTTTTTCTTATGGTGGCCCTGCGACGATCTATTTTGGAATTTGTCCTTAATGGTGAGCAGTCAAGATTGTAACAGGTTGATCAAGTGATGATCGTAGCTACAGGAGCTCAAGTGTATCGATTAGTTTGTTATAACGAGTTTATAAAGTTGTACAAGTAGCTCACCTAGATTATTGGGTTGTAATTATTGGGATTTTGTAAGCCTTAGCACTTGACAAGTTGTCGAGTGTGGCGGTAATACCTCCGATTTAGGTGTATGCTTCCGCAATTTTATTTAAAGGTTGTCTATTTTTCCTTTATTTCTTATTTATTATTAATTAGTAAATCGGGGGTGTTACACCTAGAAATGTCCGACTAGGTCTTTGTACYGACGGATTTTCACCATTTGGAAAGACAGGAAGGCAATATTCTTGTTGGCCCGTCATTTTAACTCCTTATAATCTTCCACCTGAATTGTGCATGAAGAAACCTTTTATGTTTCTAAGTTTGATAATTCCAGGTCCGAAGAATCCTAAAGGGAATCTCGATGTGTACTTGCAACCTCTTATTGAAGAGTTAAAGCAATTATGGGAGGCTGGGTTACCGACTTACAACATCTCGCAAAAACAAAATTTTCATTTGACTCAATTAGCTTTGTATAATGTGTTTGACTTTCATTTGACTCAATTAGCTTTGTTAATTACATTTAACTTTCATTTAATTCAACAAGCTTTGTTGAATGTGTTTGACTTTTATTTGACTCAAKTACCTTTGTTGAATGCATTTTACTTTCATTTGATTCAAAAAGCTTCGTTTGAATGCGTTTGAATTTCATTTRACTCAATTAGCTTAGTTGAATGCACTTGACTTTCATTTGATTCAAAAAGCTTTGTATGAATATGTTTTACTTTCATTAGACTAAATTATCTTTCTTGAATGCATTTGTCTTTCATTTGATTCAAAATCCATCCTACGAAATCAAGATGGATTATGGGAGTGTGTGACTTGAACTATTGATTAGGACGTGCAGGTATATGTCCGTTTCTGTCACATTAAAATTCATACTCAAATGTGTCTTTTGTCCAACCGCCATATAAGTAAGTCCTATAAAGAGGATAGGTT

| Indices | Period Size | Copy Number | Consensus Size | Percent Matches | Percent Indels | Score | A | C | G | T | Entropy (0-2) |
| --- | --- | --- | --- | --- | --- | --- | --- | --- | --- | --- | --- |
| 2862--3190 | 39 | 8.4 | 39 | 75 | 2 | 284 | 26 | 13 | 12 | 46 | 1.79 |

Consensus pattern (39 bp):

TTTCATTTGACTCAATTAGCTTTGTTGAATGCATTTGAC


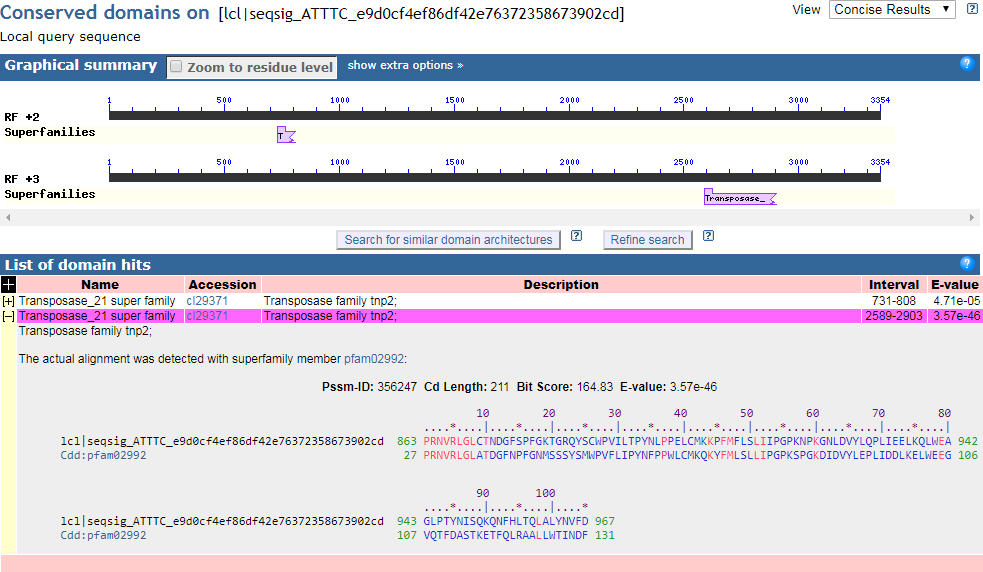


>contig 431

GATTATGTTTTAAAATTTGTTCCTATGTTCTAAATTATTTTTTTCAGCTTATGTTCTAAATTTTTTTTTCTATGTTCTAAATTATTTTTCAGCTTATGTTCTAAAGTTTTTCCTATATTCTAAATTTTTTCTACTTATGTTCCAAATGATTTATAATGTTCTAAATTGTTTTTAGATTATGTTCTAAATTTGTTCYTATGTTCGAAATTATTTTTTCAGCTTATGTTCTAAATTTTTTCTATGTTCTAAATTATTTTTTTTTNCAGATTATGTTATTAATTTTTACCTATGCTACGATTATTTTTTTCAACTTATGTTCTAAATTATTTTTTCTTATGTTCTAACTTTTTTGGTTGTTTATGTTCTACATTTTTGCTATCTTTCAAATTATTTTTTTTTATGTTCTAAAATTTTCTGCTTATGTTCTAAACTTTTTCTACTTATGTTCTAAATTTTTTTTTTCTATGTTCTAAATTTTTTTTCTATATTCTAAATACGAAATTCTCAAAATTACAGAAATGCCATTACTAAAGGATCATTTTAGATGGTTAGATCAAAAYTAATRAAGGGCTGAGATTGGTTCTTATATAAGWTTGGGTTCTTATATARAGGAGGGTTCTTACACGAGCCTTCTTCTATATAAATATATATATACTTTAATTGTAGTAGGAAAACCCTKCTCTAGTGGATAATTTTATATATGTATATATATACCAATTCTAAGAAGAGATTGAAGTGGCTGCAGGTTTTACAAATAACTTGCAATGCAAGATACCMTTTCCACCAAATTCCACGCTTTGACATGATTCATGAATTTATRATTTCRTTAGAAAGAAMATGTAGAAAATGGGAATGCWTCTTACTATTTTGCCCGAATTTGCTTGATATTGGTTCTAAAAGACCTTAAAAGCTTAATACATGCTTTGTTTTTGTGATTTATTTTCTTACGCAGTTATGAATACATATATTTGACATGAATCAATCCTTTTAAAGTTCATTAGAAAGAGGGTATAGAAAATGGGAACACTTCTTCTCATTTTGCCTGAATTTGCTTGATTTCGATTCTACCAGACCTTAAAACCTTATAAATTGCTTTCTCTTTATAATTTTTTTGATTAGTCAGTTACAAATACATATATCTAATATGATTCAAATAATTATAATTTAATTAGAAGGAGCATGTAGAAAACGGGAACGCTTCTTCCCATTTTGGCCGAATTTGCTTGATTTTGGTTCTACAACACCTTAAAACCTTATAAAATGCTTTTTTTGTTATTTTGTTTCTTAGGAAGTTATAAATACATATATTCGACATGAATCAAGCCTTTTCAAGTTCATTAGAAAGAGCATGTAGAAATTGGGAACGCTTCTTCCCATTATGCCTGAATTTGCTTGATTTCGGTTCTACTAGACCTTAAAACCTTATACCTTGCTTTGTCTTAGTGACTTCTATTCTTAGGCAGTTACTAATTAATATATTTGATATAATTCAAGCCTTTATAAGTTCATTAGAAAGATCATGTAGAAAATGGGAACGCTTTTTACCATTTGGGCCGAATTTGCTTGCAAGACCTTAAAACCTTATGACATGCTTTATTTTTGTGATTTATTTTCTTAGGAAGTTGTAAATACATATATTTGTCATGAATCAAGCCTTTTTAAGTTCATTAGAAAGAGCATGTAGAAAATGGGAACACTTTTTCCTATTAGGCCCGAATTTGATTGATTTTGGTTCTACTACACCTTAAAACCTTATAACTTGCTTCGTTTTTGTGATTTATAAGCTTAGAAAATTATAAATACATATAGTTGATATGATTCAAGAATTTATAAGTTCATTAAAAAGAGCATGTAGAAAATGGAAAGGCTTCTAACTATTTTGCCCGAATATGCTTGATTTTGGTTCTACAAGACCTTAAAACCTTATAACTTTTTTTGTTTTTGTGATTTATTTAGTTAGGAATTATAAATTCATATATTTCACATGAATCAAGCCTTTTCAAGTTTATTAAAAACAGCATGTAGAAAAAGGGAATGCTTCTTACTAGTTTGCCCGAATTTGCTTGATTTCAGTTCTACAAGACCTTAAAACCTTWTAACTTGCTTTGTTTTTGTGATTTCTATTCTTKTGCATTTATAAATACATATATTTTACATGATTCAAACAATTATAAGTTCATTAGAACGAGCATGTAGAATTTGGGAATGTTTCTTACTATTTTGCCCGAATTTGTTTGATTTTGGTTCTACAAGACCTTAAAATCTCATAACTTGCTTTNTTTTTGAAATTTTTTTTCTTATGGTGTTTTAAATACATATATTTGACATGATACAAGAATTTATAAATTCATTAGAAAGTGCATGTAGAAAATGGGAACGTTTATTCCCATTTTGCMCGAATTTGCTTGATTTTGGTTCTACAAGACCTTAAAACCTTATAACTTGATTTTGCTTTGTTATGTATTTTCTTAGGCAGTTAAAAATAGGTATATTTGACATGATTCAAGAATTTATGATTTCATTGGAAAGAACATGTAGAAAATGGGAATGCTTCTTACTATATTGCCCGAATTTGCTTGATATTGTTTCTACAGGACCTTAAAACCTTAATACATGCTTTGTTTTTGTGTTTTATTTTCTTAYGCAGTTATGAATACATATATTTGACATGAATCAATCCTTTTCAAGTTCATTAGAAAGAGTGCATAGAAAATGGGAACGCTTCTTCTCATTTTGCCTGAATTTGCTTGATTTCGATTCTACCAGACCTTAAAACCTTCTAAATTGCTTTCTCTTTGTTATTTATTTGCTTAGGCAGTTACAAATACGTATATCTAATTTCATTAGAAGGAGCATGTAGAAAACGGGAACGCTTATTCCCATTTTGGCCGAATTTGCTTGATTTTGGTTCTACAACACCTAAAAACCTTATAACATGCTTTTTTGTGATTTCTTTTCTTAGGAAGTTATAAATACATATATTAGACATGAATCAAGCCTTTTCAAGTTCGTTAGATAGAGCGTGTAGAAAATAGGAACGCTTCTTCTCATTTTRCCTGAATTTGCTTGATTTCGGTTCTACCAGACCTTAAAACCTTATAACTTGTTTTATTTTTGTGATTTCTATTCTTAGGCAGTTACAAATAAATATATTCGACATGACTCAAGCATTTATAAGTTCATTAGAAAGAGCATGTAGAAAATGGGAACGCTTTTTACCATTTTACCCGAAAATGCTTGATTTTGGTTCTACAAGACCTTAAAACCTTATAACATCCTTTTTTTTGTGATTTATTTTGTTATGAAGTTATAAATACATATATTTGACATGACTCAAGCCTTTTCATGTTCATTAGAAAGAGCATGTCGAAAATGGGAATTCTTCTTCCCATGTTGCTCGAATTTTCTTAATTTCGGTTCTACAAAACCTTAAAACCTTATAACTTGCTTTTTTTTNAATTTCTATGGTTAGGCAGTTATAAATACAGATATTTGAAATGATTCAAGCATTTATAATTTCATTAGAAAGAGTATGTAGAAAATGGGAACGTTTCTTACTATTTTGCCCGAATATGCCTGATTTTGGTACTACAAGACCTTAAAACCTTATAACATCCTTTTTTGTGATTTCTTTTTTTATGAAGTTATAAATACATATATTTGACATAAATCAAGCCTTTTCAAGTTCATTAGAAAGAGCATGTAGAAAATGGGAACGCTTCTTCTCATTTTGCCCGAATTTGCTTGATTTCAGTTCTACCAGACCTTAAAACCTTATAACTTGCTTTGTCTTAGTGATTTCTATGCTTAGGGAGTTACTAATACATATATGTGACATGATTCAAGCCTTTAAAAGTTCATTAGAAAGAGCATGTAGAAAATGGGAATGCTTCTTCCCATTTGGTCCGAATTTGGTTGATTTTGGTTCTACAAGACCTTTAAACCTTATGAAATCCTTTGTTTTTGTGATTTATTTTGTTAGGAAGTTATAAAAATACATATATTTGACAGGACTCAAGCCTTTTCAAGTTCATTAGAAAGTGCATGTAGAAAATGAGAACGCTTCTTCTCATTTTGCCTGAATTTGCTTGATTTCGGTTCTATCAGACCTTAAAACCTTATAAATTGCTTTCTCTTTGTTATTTATTTTCWTAGGAAGTTACAAATACATATATTTGACATGATTCAAGCCTTTGTAAGTTCATTAGAAGGAGCATGTAGAAAATTGGAACGTTTCTTCCCATTTTGGCCGAAKTTGCTTGATTTTGGTTCYACAAGACCTTAAAACCTTGTAACATGATTTTTTTGTGATTTATTTTCTTAGGAATTTATAAATACATATATTAGACATTAATCAAGTCTTTTCAAGTTCATTAGAAATAGCGTGTAGAAAATGGGAACGCTTCTTCTCTTATGATGTTAATTGGTAATAATTTTCATTCTGATGACCTTAAATAGTTAAATGACCTAAAAGAATGTCATTTTTCTAAGTGCTTTACTTACTTTGTCTATGCTTTTTGTATCATTTATTTTTTAGTGAAACTGCTATATAATGAGGGAGGATCGAGCATGGATGTATAAACGGTTAGAAGGAAAATTCCTTAGTCCAACCTTTGCTGAAAAGGTCAATGAATTTATTACATTTGCTACCACACAAGAGAATGTCGTGATAGATGGTGTGATGAAATGTCCATGTGCACAGTGTCGAAATATTCCTTATCAAGATCTTGATACCATTATTGAGCATCTCTATAGGCATGGTTTTTTGCCTAACTATTTCCAATGGGTTTTTCATGGTGAACTGCACTTCCAAAGAAAATCCCAAAGTAGCAGTTCCATATCCACCGAGGATGCATTGAACCCATATAGAAACATGGTATTAGATGCCTTTGGACTAGAAGGTGGTTTAGAAAATATTGAAGAAGAGCCACATGCTAGTTACAAGAAGTTCTTTGATATGTTGAAGGCAGCGGAAGAGCCATTGTATGATGGCTGCAAGTTGTCTGTATTGTCTGCAGCTGCAAAGATGGCAAACATCAAGTCTTTTGCTGCGGAGCCTAGAAATGTCTGAATAGGTCTTTGTACCGACGGATTTTCACCGTTTGGAAAGACAGGAAGGCAATATTCTTGTTGGCCCGTCATTTTAACTCCTTATAATCTTCCACCTGAATTGTGCATGAAGAAACCTTTTATGTTTCTAAGTTTGATAATTCCAAGTCCGAAGAATCCTAAAGGGAATCTCGATGTGTACTTGCGACCTCTTATTGAAGAGTTAAAGCAATTATGGGAGGCTGGGTTACCGACTTACAACATCTCGCAAAAAACAAAATTTTCATTTGACTCAATTAGCTTTGTATAATGTGTTTGACTTTCATTTGACTCAATTAGCTTTGTTAATTACATTTAACTTTCATTTAATTCGACAAGCTTTGTTGAATGTGTTTGACTTTTATTTGACTCAAGTACCTTTGTTGAATGCATTTTACTTTCATTTGATACAAAAACTTTCTTTCAATGTGTTTGACTTTCATTTCACTTAGTTAGATTTGTTGAATGCATTTGACTTTAGTTATATTCAAAAAGCATTGTTTGAATGTGTTTGGCTTTCATTTTACACAATTATCTTTGTAGACTGCATTTGACATTCATTTAACTCAATTAGCTTGTATGAATGTGTTTGACATTCATTTGACTCAATTAGCTTTGTTAATTGCACTTCACTTTCATTTAATTCAAAAAGCTTTGTTTGAATGTGTTTCACTTTCATTTGATTGAAAAAGCTTTGTTTGAATGTGATTGACTTTCATTTGACTCAATTACCTTTGTTGAATGCATTTGACTTTCATTTGATTCAAAAAGTATTGTTTGAATGTGTTTGACATTCAATTGACTCATTTAGTTTTA????????????????????????????????????TTGTTTGAATGTGTTCTAATTTTATTTGAGTCAAAAAGGTTTGTTGAATGCATTTGACTTTCATGTGATTCAAAAAGCTTTGTTTGAATGTGTTTAACTTTCATTTAACTCAATTATCTTTGTTGAATGTATTTGACTTTCTCTTGATTCAAAAAGCTTTGTAACAATATGATAAAATTTCGTTTGACTCAATTAGCTTTGTTAATTGCATTTCATTTTCATTTAATTCAAAACGATTTGTTTGAATGTATTTTACGTTTATTTGACTCAATTAACTTTGTTGAATTCATTTTACTTTCATTTGATTCAAAAAGCTTTTTTAGAATGTGTTTGACTTAATTAGCTTTGTTGAATGCATTTGACTTTCATTTGTTTCAAAAAGCTTTGTTTGAATGTGTTTAACTTTCATTTGAGTTAAAAGCATTGTTGAATGCACTTGACTTTCGTTTCATTTAAAAAGCTTTGTTTTAGTGTGTTTGACATTCATGAAAGGAATGGTGTCCTTC

| Indices | Period Size | Copy Number | Consensus Size | Percent Matches | Percent Indels | Score | A | C | G | T | Entropy (0-2) |
| --- | --- | --- | --- | --- | --- | --- | --- | --- | --- | --- | --- |
| 5331--6241 | 39 | 23.1 | 39 | 72 | 9 | 508 | 26 | 12 | 13 | 47 | 1.79 |

Consensus pattern (39 bp):

TTTCATTTGACTCAATTAGCTTTGTTGAATGTGTTTGAC


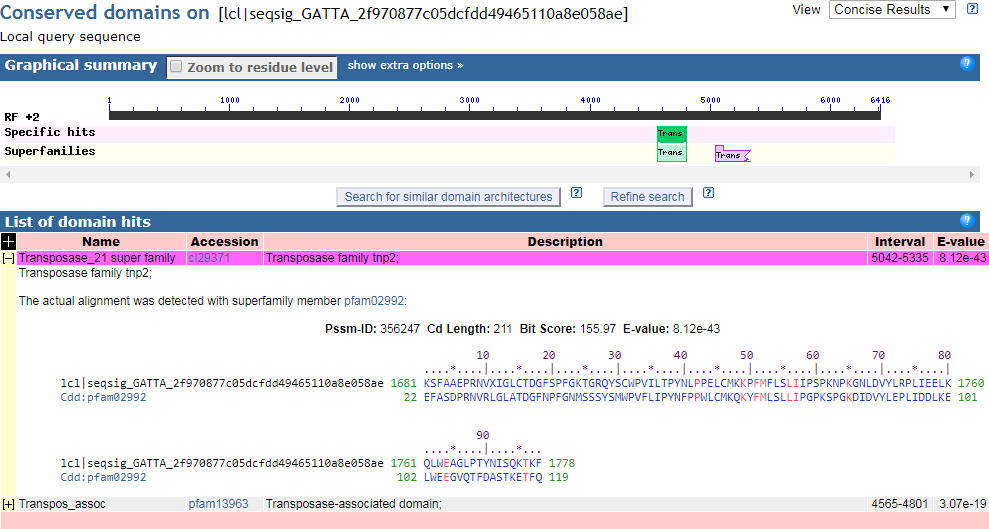


>contig 545

AAATAACTCGGATATTCTTTTGGATTAACTTCACTTCAACTTAGCTAGGAAATAGGCTTACTAATACTTTCAGCTTAGTCCTTAGTGCCATAGTATTAAAATTGTGTTAGCAGGTTTTCTATGTTTGCTTGATACAAGATTCAGCTAGTGCCATATATTAAAAACAGTGTTAGCAGGTTTTCTATCTTTGCTGGATATATGATATATTATGAACCCATATAGAAACATACTTTTAGATACCTTTGGACTAGAAGGTGGTTTAGAAAATATTGAAGAAGAGCCACATGCTAGTTACAAGAAGTTCTTTGACATGTTGAAGGCAGCGGAAGAGCATTGTATGATGGCTGCAAGTTGTTTGTATTGTCTGCAGCTGCAAGGATGGCAAACATCAAGTCCGAGTATAATATTCCACATAAAGCTATAGATGGTGTTGCTTCCTTAATGAAAGATAGTTTTTACAAGACTAAGAAATTGCTTGAAGGGCCAGAACTTCCGCACCAAAAGATTCATGTATGTCCAAATGGATGTATGCTATTTTGGAAGGAACACAAAGATCTCAAAGAATGTCTATATTGCAAAGGAAGTCGTTATAAGACACTTAGAGAAAGTGGCAACAATTCTCCTCATAGTGCTCTTATTTACTTTCCTGTAGGCCCGAGATTACAAAGATTATATGCAACAAGATCTACTGCGGAGCAGATGAGGTGGCATAAAGATAATCCTCGAGTTCATGGCCTTATGTCTCATCCAAGTGATGAAGAGGCGTGGAAACACCTAGATGAAGAGTACCCTTCTTTTGCTGCGGAGCCTAGAAATGTCCGACTAGGTCTTTGTACCGACGGATTTTCACCATTTGGAAAGACAGGAAGGCAATATTCTTGTTGGCCCGTCATTTTAACTCCTTATAATCTTCCACCTGAATTGTGCATGAAGAAACCTTTTATGTTTCTAAGTTTGATAATTCCAGGTCCGAAGAATCCTAAAGGGAATCTCGATGTGTACTTGCGACCTCTTATTGAAGAGTTAAAGCAATTATGGGAGGGCTGGGTTACCACTTACAACATCTCGCAAAAACAAAATTTTCATTTGACTCAATTAGCTTTGTATAATGTGTTTGACTTTCATTTGACTCAATTAGCTTTGTTAATTACATTTAACTTTCATTTAATTCAACAAGCTTTGTTGAATGTGTTTGACTTTTATTTGACTCAATTACCTTTGTTGAATGCATTTTACTTTCATTTGATTCAAAAAGCTTCGTTTGAATGCGTTTGAATTTCATTTGACTCAATTAGCTTAGTTGAATGCATTTGACTTTCATTTGATTCAAAAAGCTTTGTATGAATATGTTTTACTTTCATTAGACTCAATTATCTTTCTTGAATGCATTTGTCTTTCATTTGATTCAAAAACCATGTTTGAATGTGTTTGACTTTCATTTGACTCAATTAGCTTTGTTGAATGCATTTTACTTTCATTTGATACAAAAAGCTTTCTTTCAATGTGTTTGACTTTCATTTTACTCAATTAGCTTTGTTGAATGCATTTGACTTTAGTTTGATTCAAAAAGCATTGTTTGAATGTGTTTGACTTTCATTTGACTCAATTAGCTTTGTAGACTGCATTTGACTTTCATTTAACTCAATTAGCTTTGTATGAATGTGTTTGACATTCATTGACGTGGGGAAAACTAGGTCGTTGGCTAATTCTCCCTAAGCCAAACAAAATTTATAAACACTCAACAAACCTAAATAAAACAAGTATAGAGGTAAGTCAAGGGTCGAACCCAAAGGAACGGTAATGTCTAAATGCT

| Indices | Period Size | Copy Number | Consensus Size | Percent Matches | Percent Indels | Score | A | C | G | T | Entropy (0-2) |
| --- | --- | --- | --- | --- | --- | --- | --- | --- | --- | --- | --- |
| 1081--1677 | 39 | 15.2 | 39 | 76 | 5 | 549 | 25 | 13 | 13 | 46 | 1.81 |

Consensus pattern (39 bp):

TTTCATTTGACTCAATTAGCTTTGTTGAATGTGTTTGAC


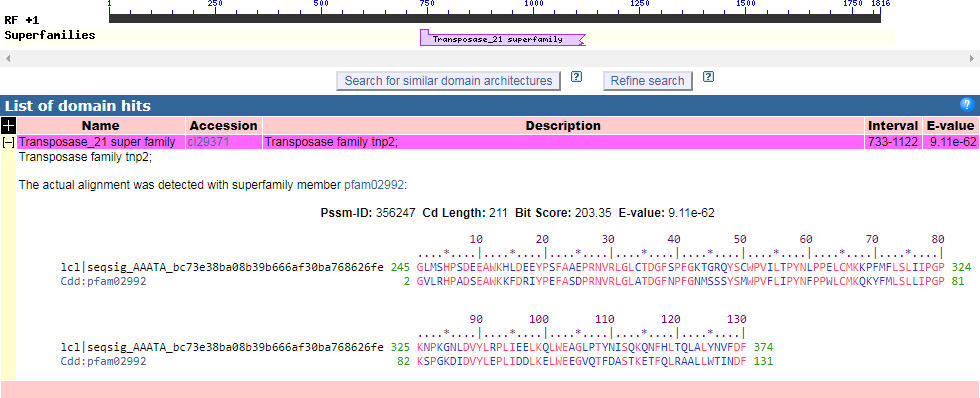


*C. striatiforme*

>contig 541

ATCTTATATACTACAAAAATAAAACCCAAAACAAAGGTTTTTGAATCAATTGAAAGTCAAATGCATTCAACAAAGCTTTTTTACTCAAATGAAAGTCAAACACATTCAAACAAATCTTTTTGAATCNAAATGAAAGTCAAATGCATTAAACAAAGCTAATTGAGTCAAATGAAAGTCAAACCCATTCAAACAAAGCTTTGAATCAAAGAAATCAAATGAATTCAACAAAGCTAATTGAGTCAAATAAAAGTCAAACACTTTCATCAAAGCTTTTAGAACAAATTAAGTCAATATATTCAAACAAAGCTTTTTGAATCAAATGAAAGTCAATTGCATTCCACAATGCTAATTGAGTCAAATGAAGGTCAAACACATTCAACGAAGCTAATTGAGTCAAATGAAAGTCAAACACATTCAAACAAAGCTTTTTGAATCAAATGAAAGTCAAATGCATTCAACAAAGCAATTGAGTCAAATGAAAGTCAAACACATTCAAACAAAGCTTTTGATCAAATGAAAGTCAAATGCATTCAACAAAGCTAATTGAGTCAAATGAAAGTCAAACACTTTCAACAAAGCGTTTAGAATCAAATGAAAGTCAAATACATTCAATCAAAGCTTTTTGAATCAAATGAAAGTCAATTGCATTCCACAATGCTAATTGAGTCAAATGAAAGTCAAACACATTCAACAAAGCTAATTGAGTCAAAAGAAAGTCAAACACATTCAAACAAAGCTTTTTGAATCAAATGAAAGTCAAATGCATTCAACAAAGCTAATTGAGTCAAATGAAAGTCAAACACTTTCAACAAAGCTAATTGAGTCAAATGAAAGTCAAACACATTCAAACAAAGCTTTTTGATTCAAATGAAAGTCAAATGCATTCAACAAAGCTAATTGAGTCAAATGAAAGTCAAACACTTTCAACAAAGCTTTTAGAATCAAATGAAAGTCAAATACATTCAAACAAAGCTTTTTGAATCAAATGAAAGTCAATTGCATTCCACAATGCTAATTGAGTCAAATGAAAGTCAAACACATTCAACAAAGCTAATTGAGTCAAAGAAAGTCAAACACATTCAAACAAAGCTTTTTGAATCAAATGAAAGTCAAATGCATTCAACAAAGCTAATTGAGTCAAATGAAAGTCAAACACTTCAACAAAGCTAATTGAGTCAAATGAAAGTCAAACACATTCAAACAAAGCTTTTTGAATCAAATGAAATCAAATGCATTCAACAAAGCTTTTTGAATCAAATGAAAATCAAATGAATTCAACAAAGCTAATTGAGTCAAATGAAAGTCAAACACATTCAAACAAAATTTTTTTTTTTTTTCTATTTGGCAGTTATAAATACATATATTTGACATGATTCAAGCATTTATAAGTTCATTAGAAAGAGTATGTAGAAAATGGGAATGCTTCTTACTATTTTTCCCGAATATGCTTGATTTTTGTACTACAAGACCTTAAAACCTTATAACATCCTTTTTTTGTGATTTATTTTCTTAGGAAGTTATAAATACATAAATTTGACATAAATCAAGCCTTTTCAAGTTCATTAGAAAGAACATGTAGAAAATGGGAACGCTTCTTCCCATTTGCGCCGAATTTGGTTGATTTTGGTTCTACAAGACCTTAAAACCTATAACATCCTTTGTTTTTGTGATTTCTTTATTAGGAAGTTATAAATACATATATTTGACATGACCAAGCCTTTTCAAGTATTAGAAAGTGCATGTAGAAAATGAGAACGCTTCTTCTCATTTTGCCTGAATTTGCTTGATTTCGGTTCTATCATACCTTATAACCTTATAAATTGCTTTCTCTTTGTTATTTATTTTCTTAGGCAGTTACAAATACATATATTTGACATGATTCAAGCCTTTATAAGTTCATTAGAAGGAGCATGTAGAAAATGGGAACGCTTCTTCCCATTTTGGCCGAATTTGCTTGATTTTGGTTCTACAAGACCTTAAAACCTTATAACATGCTTTTTTTTGTGATTTCTTTTCTTAGGAATTTATAAATACATATATTAGACATGAATCAAGCCTTTTCAAGTTCATTAGAAAGAGCGTGTAGAAAATGGGAACGCTTCTTCTCATATGATGTTAATTGGTAACAATAATTTTCATTTTGATGACCTTAAATAGTTAAATGACCTAAAAGAATGTCATTCTTCTAAGTGCTTTACTTACTTTGTCTATGCTTTTTGTATCATTTATTTTTTAGTGAAACTGCTATATAATGAGGGAGGATCGAGCATGGATGTATAAACGGTTAGAAGGAAAATTCCTTAGTCCAACCTTTGCTGAAAAGGTCAATGAATTTATTACATTTGCTACCACACAAGACAATGTCGTGATAGATGGTGTGATGAAATGTCCATGTGCACAGTGTCGAAATATTCCTTATCAAGATCTTGATACCATTAAGGAGCATCTCTATAGGCATGGTTTTTTGCCTAACTATTTCCAATGGGTTTTTCATGGTGAACTGCACTTCCAAAGAGAATCCCAAAGTAGCAGCTCCATATCCACCGAGGATGCATTGAACCCATATAGAAACATGGTATTAGATGCCTTTGGACTAGAAGGTGGTTTAGAAAATATTGAAGAAGAGCCACATGCTAGTTACAAGAAGTTCTTTGACATGTTGAAGGCAGCGGAAGAGCCATTGTATGATGGCTGCAAGTTGTCTGTATTGTCTGCTGCTGCAAGGATGGCAAACATCAAGTGCGAGTATAATATTCCACATAAAGCTATAGATGGTGTTGCTTCCTTAATGAAAGACATGTGCCCAGATGAAAACAAGATGACAAATAGTTTTTACAAGACTAAGAAATTGCTTGAAGGGCTAGAACTTCGCACCAAAAGATTCATGTATGTCCAAATGGATGTATGTTATTTTGGAAGGAACACAAAGATCTCAAAGAATGTCTATATTGCAAAGGAAGTCGTTATAAGACACTTAGAGAAAGTGGCAACAATTCTCCTCATAGTGCTCTTATTTACTTTCCTATAGGCCCGAGATTACAAAGATTATATGCAACAAGATCTACTGCGGAGCAGATGAGGTGGCATAAAGATAATCCTCGAGTTCATGGCCTTATGTCTCATCCAAGTGATGGAGAGGCGTGGAAACACCTAGATGAAGAGTACCCTTCTTTTGCTGCGGAGCCTAGAAATGTCCGACTAGGTCTTTGTACCGACGGATTTTCACCATTTGGAAAGACAGGAAGGCAATATTCTTGTTGCCCGTCATTTTAACTCCTTATAATCTTCCACCTGAATTGTGCATGAAGAAACCTTTTATGTTTCTAAGTTTGATAATTCCAGGTCGAAGAATCTTAAAGGGAATCTCGATGTGTACTTGCAACCTCTTATTGAAGAGTTAAAGCAATTATGGGAGGCTGGGTTACCGACTTACGACATCTCGCAAAAACAAAATTTTCATTTGACCCAATTAGCTTTGTATAATGTGTATGACTTTCATTTGACTCTATTAGCTTTGTTAATTGCATTTAACTTTCATTTAATTCAACAAGCTTTGTTTGAATGTGTTTGACTTTAATTTGACTCAATTAGCTTTGTTGAATGCATTTTACTTTCATTTGATTCAAAAAGCTTCGTTTGAATGTGTTTGAATTTCATTTGACTCAATTAGCTTGTTGAATGCATTTGACTTTCATTTGATTCAAAAAGTTTGTTGAATGTGTTTACTTTCATTGACTCAATTTCTTTTTGAGCATTTGCTTTCATTTGATTTAAAAAGCTTTGGTTGAATGTGTTTGACTTTCATTTGAGTCAATTATCTATGTTTAATACATTTGACTTTCATTTGATTCAAAAAGCTTTATTTGAATGTGTTTGACTTTCATTTGACTCAATTAGCTTTGATGAATGCATTTGACTTTAATTTAATTCAAAAAGCTTTGTTTGAAAGTGTTTGACTTTCATGTGACTCAATTAGCTTTGTTGAATGCATTTGAGTTTTATTTGACTCAAAATGCTTTGTATGAATGCATTTGACTTTCATTTGATTCAATATATCTCTTTTTTACAGCTATTGAAAACAAGTTGTCTTACTTGTTGTTGCTAGATTTCATTTGACTCAATTATCTTTGTTGAATTCGTTTTACTTTCATTTGATTCAAAAAGCATTGTTTGAATGTGTTTGACTTTCATTTGACTCAAAAATCTTTATTAGATTTTGTTTGACTTACATTTGACCCAACTAGCTTTATTGAATGTGTTTGACTTTCATTTGACTCAATTAGCTTCGTTGAATGCATTTGATTTCATTTGATTCAAAAAGCTATTAGAATGTGTTTGATTTTCATTTGACCAGTTAGCTTTGTGAATGTGTTTGACTTTCATTTAACTCAATTAGCTTTGTTGAATGTGTTTAACTTTCATTTGATTCTAAAAGCTTTGTGGAAAGTGTTTTACTTTCAATTTACTCAATTAGCTTTGTGGAATTCATGTGACTTACATTTTATTCCAAAAGCTTTGTTAGAATGTGTTTGACTTTCATTTGACTATATTATCTTTGTTGAATGCAGTTGACTTTTATTATTTGACTCAAAAAGCTTTGTTTGAATGTGTTTAGCTTTAATTTGACTCAATACGCTTTATTGCATGCATTTGACTTTCATTTGATTCAAAAAGCTTTGTTGAATGTGTTTATTTCATTTGTCAATTAGCTTTGTTAATTGCATTTCACTTTCATTTAATTAAAAAGCTTTTTGAATTTTTGACTTTCATTTGACTCAATTAGCTTTGTAGAAAGCATTTTACTTTCATTTGATTCAAAAAGCTTTGTTTGAATATGTTTCACTTTCGTTAGACTCAATTATCTTTGTTGAATGCATTTGATTTTCATTTGATTTAAAAAGCTTTGTTTGAATGTGTTTGAATTTTATTTACTCAATTATTTGTTGAATGCATTTGACTTTATTGATTCAAAAGTTTGTTTGAATGTGTTTACTTTCTTTACTCAATTAGCTTGTGAATGCATTTGATTTTCATTTGACTCAATTAGCTTTTATGAATATGTTTGACTTTCATTTACTCAATTAGCTTTGAATTGCATTTCACTTTCATTTAATTTAAAAAGCTTTGCTTAATGTGTTTGATTTTCATTTGATTCAAAAAGCTTTGTTGAATGAATTTGACTTCATTTGATTTAAAAAGCATTATTTGAATATGTTTGAATTTCATTTGACTCAATTAGCTTTGTTAAATGCATTTTACTTTATTTGATTCAAAAAACTTTTTTTGAATGTGTTTGACTTTCATTTGACTAAATTAGCTTTTGACTTTCAGTTGATTTAAAAAGCTTTGTTTGAATGTGTTTGGCTTTCATTTGATTAATATAGCTTTGTTAAATGCATTTGACTTTCATTTGCTTCAAAAAGCTTTGTTTAATTCAGTTTACCTTCATTTGCTTTAAAAAGGTTTTGTTAATGTGTTTTACTTTCATTTTACTCAAAAAGCTTTGTTGAATGTATTTGGCTTTCATTTTCTTCAAAAAGTGTTGTTTTAATTTGTTTGCCTTTCATTTTATTCAAAT

| Indices | Period Size | Copy Number | Consensus Size | Percent Matches | Percent Indels | Score | A | C | G | T | Entropy (0-2) |
| --- | --- | --- | --- | --- | --- | --- | --- | --- | --- | --- | --- |
| 3455--5529 | 39 | 54.2 | 39 | 72 | 12 | 1205 | 25 | 12 | 14 | 47 | 1.79 |
| 5320--5577 | 39 | 6.6 | 39 | 77 | 10 | 195 | 24 | 11 | 13 | 50 | 1.75 |

Consensus pattern (39 bp):

TTTCATTTGACTCAATTAGCTTTGTTGAATGTGTTTGAC

Consensus pattern (39 bp):

TTTGACTTTCATTTTCTTCAAAAAGCTTTGTTTAATGTG


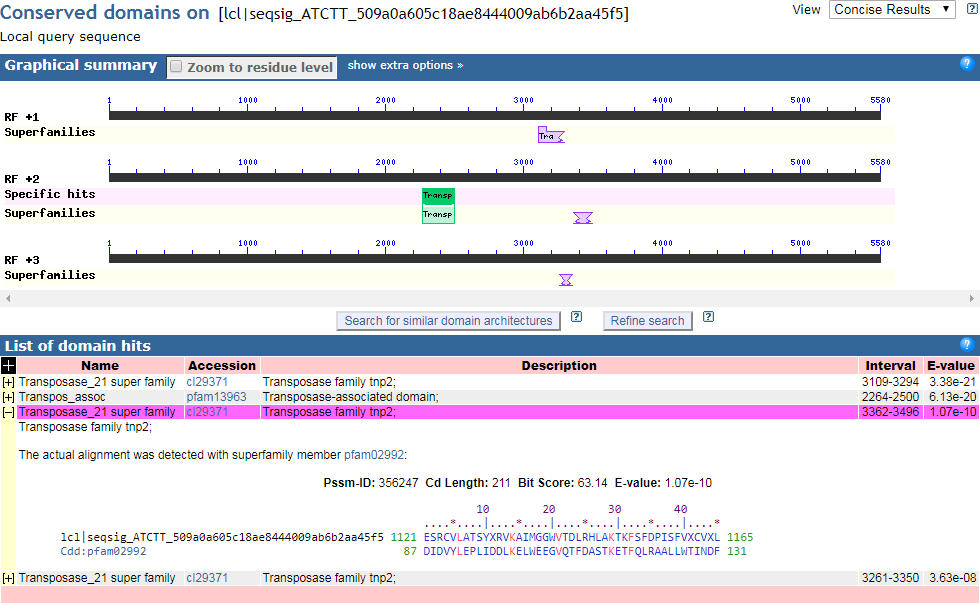


>contig 28391 (partial)

AAGAAATTGCTTGAAGGGCTAGAACTTCTGCACCAAAAGATTCATGTATGTCCAAATGGATGTATGTTATTTTGGAAGGAACACAATTCTCCTCATAGTGCTCTTATTTACTTTCCTATAGGCCCAAGATTACAAAGATTATATGCAACAAGATCTACTGCGGAGCAGATGAGGTGGCATAAAGATAATCCTCRAGTTCATGGCCTTATGTCTCATCCAAGTGATGGARAGGCGTGGAAACACCTAGATGAARAKTACCCTTCATTTGCTGCGGAGSMTAGAAATGTCCGACTAGGTCTTTGTACCGACAGATTTTCACCATTTGGAAAGACAGGAAGGCAATATTCTTSTTGGCCTGTCATTTTAACTCCTTATAATCTTCCACCTGAATTGTGCATGAAGAAATCTTTTATGTTTCTAAGTTTGATAATTCCAGGTCCGAAGAATCCTAAAGGGAATCTCGATGTGTACTTGCAACCTCTTATTGAAGAGTTAAAGCAATTAWGGGAGGCTGGGTTACCGACTTAAGACATCTCGCMAAAAYAAAATTTTCATTTGACTCAATTAGCTTTGTATAATGTGTTTGACTTTCATTTGACTCAATTAGCTTTGTTAATTGCATTTAACTTTCATTTAATTCAACAAGCTTTGTTWGAATGTGTTTGACTTTAATTTGACTCAATTAGCTTTGTTGAATGCATTTTACTTTCATTTGATTCAAAAWGCTTCGTTTGAATGTGTTTGAMTTTCATTTGACTCAATTAKCYTWGTWGAAYGCATTTGACTTTCATTTGATWCAAAAARCTTTSTWTNGAATGTGTTTGACTTTCATTTRRCTCAATTATCTTGGTTGAATGCATTTGTCTTTCATTGGATTCAAAAAGCTTTTTTTGAATGTGTTTGACTGTCGTTTGAATCAGTTAGGTTTGTTGAATGCATTTGACTTTCATTTTACTGTATTTGCTTTGTATGAATTTTGTTTACTTTCATTTGATTAAAAAAAGCTTTGTWTGAATGTGTTTGAATTTCATTTGACTCAATTAKCTTTGTTGAATGCATTTGGCTTTCATTTAATGTAAAAAGCTTTGTTTGAATGTGTTAGATTTTATTTGAGTCGAATTGCTTTTTTGAATGCATTTGACTTTCATTTGGTTCAAAAAGCTTTTTTTGTACGGGTTTTACTTTCATTTGACTCAATTAGCTTTGTACAATGCATTTGACWTTTATTTGACTCAATTAGCTTTTTATGAATGTGTTAGACTTTCATTTAACTCAATTAGCTTTGTTAATTGCATTTCACTTTGAATTAATTCAAAAAGCTTTGTTTGAATGGATTTGACTTTCATTTGACTCAATTAACTTTGTTGAATGCATTTGACTTTCATTTGATTTAAAAACCTTTGTTTGAATTTGTTTTACTTTCATTTTATTTAACTGTCTTTTTTGTATTTATTTTACTTTTATTTT

| Indices | Period Size | Copy Number | Consensus Size | Percent Matches | Percent Indels | Score | A | C | G | T | Entropy (0-2) |
| --- | --- | --- | --- | --- | --- | --- | --- | --- | --- | --- | --- |
| 550--1427 | 39 | 22.2 | 39 | 71 | 7 | 540 | 24 | 11 | 14 | 47 | 1.78 |

Consensus pattern (39 bp):

TTTCATTTGACTCAATTAGCTTTGTTGAATGTGTTTGAC


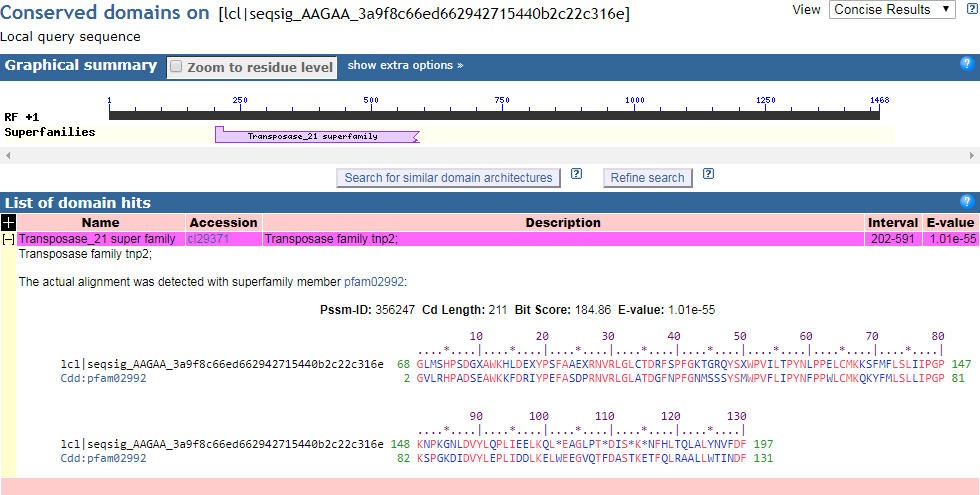


*C. strictum*

>contig 700

TACGATACATTTTGTGCACAGAATCGGGGTTTTTTTATTTATTTATTTTTTATGTGAAGAGAATCCGTTATAAAATGATAATAAAATGTGCAAAAGTTTCACTAAAACGCACAAAAGTTACCCTGACATATATATAAGAGTTATTTATGCTTCATTGATTAATTTGACAAAAGTGACACTAAATTTGACAAAAGTGTACCCGATGTACAGTAAATTACTGAAAACCGGGTGTATTCAAAAGTTTTCCAAAATAATAAATGTTACTTGTATATATAGATGATTAGAGTTGATGACTATCATGGTGACAGTTTGCATCCGCAATATAAATAGCTAATTTCATCGAACACTATAATATAAATGTTCTTTTTCGTTCAAAATTAGTGTCACAAGACTCACAACTCACATTATTCCAATCTCTTTTAATAATTTTACATTAAAAATAAAACCCTTAATAATAGTTGCCGCTTTTCCTTGCTGCGGATGCGCCTGGAGATGAGGGACCCATCTCTGGCAGTATAGGTTTCAACGTCACCGTCGTCACCTGAGTAATGGTCCGACTGGACCAATACTTCTTCCCGGGACCGATTGGTCTCTGGTGGCTGTCTGGTCTCCGGTGGCCGATTGGTTTCCGGAGGCCGATTGGTTACAGAAATTGGTACAGTTTCCAAAGAGGCCATCTTAGAGGTACTCTCCCCACAGACGGTGCCAAATGTTTCGAGGTTTTTTGAGTCCCTTGGGAGAGTTACAACACTAAGACTGGATGCTCGCTTGGATACCTGTACCTGCAAAACATCGGAGCACCGAATGGTCGGTGGTCCGCCTCCAAAGCTTAAGTTAGTCTAAGTCCGAAGAATAAGGTGCGGAATGTAAAGGGTTATCTGCTCGGATTCCGAGCTCGGGTGTACTGTTCTGTTGAGAGTGACAGTTCTAACAATGATTAGTAATCACTGAGTATATCTCACGTAAAAAGTGTCAAGATGTTTTACGCGTTGCGTAAATATGTATGACGTAGCCATAAATGAGGAGGTCATTTCCTATTTATAGGCTTCTCGGAAACCGTGCACTCCCTAGGGTTTCGTGCCCTAGGGAATATTCAAGCAGCCTTTGCAGACCTCGGGATTTGGCCTAAAGCGCCAATCCCAGGTCCTTTTATCGTCTGCCACGTGTCGTTCCCTGAGAGGTCCAAGGTGGCGCCCCTTTCAGTGACACGTGGCGGACTCTGATTGGGCCAACTGGCCTAGGGTATTTTTACCCGAATCAACTTTCATTTGATTCAAAAAGCTTTATTTGAAGGTGTTTGACTTTCGTTTGACTCAATTAGCTTCGTTAAATGTGTTTGACTTTCATTTGAATCAATTAGCATTGTAGAATGCAATTGACTTTCATTTGATTCAAAAGCTTTGTTGAAGTATTGACTTTCATTGATTCTAAAAGCTTTGTGAATGTGTTTGACTTTCATTTGACTCAATTAGCTTTGTTGAATCATTTGATTTCATTTGATTCNAAAAGCTTTGTTGAATGTGTTTGACTTTCATTTGATTATAAAAGCTTTGTTGAAAGGTTTGACTTTCATTTGACTCAATTAGCTTTGTGGAATGCAATTGACTTTCTTTTGATTCAAAAATCTTTGTATTAAAGTATTTGACTTTCATTTAATTTTAAAAGCTTCTTTAAAAGTGTTGGACTTTGATTTGACTCAATTAGCTTTGTGGAATGCATTTGACTTTCATTTGATTCAATAAGCTTTGTTTGAATGTGATTGACTTTCATTTGAGTCAAAAAGCATTGTTGAATGCATTTGACTTTCATTTGATTCAAAAATCTTTGTTTGAATGTTTTTGACTTTCATTTGACTCAATTATCTTTGTTGAATGCATTTCATTTTCATTTGGTTCTAAAACCTTTGTTTGAATGTGTTTGATTTTCATTTGACACAATTATCATTGTTTGAATGTGTTTGACTTTCATTAGATTCAAAAAGCTTTGTTTGAATGTGTTTGAAATTCATTTGATTCAAAATACATTGTATTAATGTGTTTGACTTTATTTACACAATTATCTTTGTTGAATGCATTTGAGTTTCATTTAATCAAAAAGCATTGTTTGAATGTGTTTGACTTTGTTTGACTCAATTAGCTTTGTTGAATGCAATTTACATTCATTTGATTTAAAAAGCTTTGTTTGAATGTGTTTGACTTCAATTTCACTCAATTATCTTTGTTGAATGCATTTGACTTTCATTTGATTCAATAAGCTTTGTTTGAATGTGTTTGACTTTCATTTGACTCAACTATCTTTGTTGAATGCATTTGACTTTCATTTGATTCAAAAATCTTTGTTTGAATGTGTTTGACTTTCATTTGACTCAATTAGCATTGCTGCATGCATTTGACTTTCACATGATTCAAAAAGCTTTATTGTTTGAATGTGTTTCACTTTCATTTGACTCAATTATCTTTATTGAATGCAATTAAGTTTCATTTTATTCAAAAAGCTTTGTTTGATGTTTTTGACTTTCATTTGAGTCAAAAAACTTTATTGAATGCATTTGACTTTTATTTCGTTCAAAAAGCATTGTTTGAATGTGTTTGACATTCATTTGATTCAAAAAGCTTTGTGTGAATAGGTTTGACTTTCATTTTATTCAAAAAGCTTTGTTTGAATGTTTTTTACTTTCATTTGACTCAAATCTTTGTTGAATGCATTTGATTTCATTTGATTCAAAAAGCTTTGTTTGAATGTGTTTGACTTTCAATTGACTCAATTGTCTTTGTTGAATGTATTTGACTTTCATTTGATTCAAAAAGCGTTGTTGCTATTAGAAATTAGCCGTCTCATTATTACTGCTAGATGATAACATATTTTTTTATTTGTTGTCATTTAGCAATGCCATTTTACAAAACGTTTGGCAACAAAATTTCTAGTTGTTGCTAATTGGGACACTTGTTTTACTATTTGTTGCTAAAATGACATCGCACTTTATTTTGTTGCCATTAGAAATAAGTTGTGTTATAAATTGTTGCTAATTTATTAAGAGTAATTGAAGTAATTTAAATAATATGTTGTAATAAATAAAACGAAAACAAGTCATCTTGTTCTATACAAAAGAAATATTAGAAGGAATTAATAATCGTTCAATCACTTTTAGCAATAAAATGAAGGCGATAAAATAATATATATTATTTTATAAGTTCATTAGAAAGAGCATGTAGAAAACGGGAATACTTCTTACTCTTTTGCCCGAATTTGCTTGATTTTAGTTCTACAAGACCCTAAAACCTTATGAATTGCTTTGTTTTTGTGATTTCTTTTGTGAGGCAGTTATGAATAAATATTTTTGACATGACTCAAGCCTTTTCAAGTTCATTAGAAAGAGCATGTAGAAAATGGGAATGCTTCTTCTCATTTTGCCCGAATTTGCTTGATTTCGGTTCTACCAGACCTTAAAACCTTATAATTTGCTTTGTCTTAGTGATTTCTATGCTTAGGCATTACAAATACATATATTGACATGATTCAAGCCTTTTAAGTTCATTACAAAGAGCATGTAAAAAATGGGAACGTTTCTTCCCATTTGGGCCGAATTTGCTTGATTTTGGTTCTACAAGACCTTAAAACCTTATAACATGCTTTGTTTTTGTGATTTCTTTTCTTAGAAAGTTATAAATACATATATTTGACATGAATCAAGCCTTTTCAAGTTCATTAGAAAAACCACGTAGAAAATGGGAATGCTTTTTCCCATTTCCCCGAATTAGCTTGATTTCGGTTCTACTAGACCTCAAACCTTATAACTTGCTTTGTCTTAGTGATTTCTATGCTTAGGCAGTTACTAATACATATATTTGACATGATTCAAGCCTTTATAAGTTCATTAGAAAGAGCATGTAGAAAATGTGAACGCTTTTTACCAGTTGGGCCGAATTTGGTTGATTTTGGTTCTATAAGACCTTAAAACCTTATAACATGCTTTGTTTTTGTGATTAGTTTTCTTAGGAAGTTCTTCTTACTATTTTGCCCGAATTTGCTTGATTTTGGTTCTACAAGACCTTAAAATCTTATAACATGATTTTTTATGATTTCTTTTCATAGGAAGCTATAAATAAATATATTAGAATGAATCAAGCCTTTTCAAGTTATTAGAAAGAGCGTGTAGAAAATAGGAACGCTTCTTCTCATTTTGCCTGAATTTGCTTGATTTCGGTTCTACCAGACCTTAAAACTTATAACTTGCTTTGTTTTTGTGATTTCTATGCTTAGGCAGTTACAAATAAATATATTTGACATGACTAAGCATTTATAAGTTCATTAGAAAGAGCATGTAGAAAATGGGAACGCTTCTTACCATTTTGCCCGAAAATGCTTGATTTTGTTTCAGAAGACCTTAAAACCTTAAAACATCCTTTTTTTTATGATTTATTCTGTTATGAAGTTATAAATACATATTTTGACATGACTCAAGCCTTTTCATGTTCATTAGAAAGAGCATGTTAAAATGGGAATGCTTTTTCCCATTTTGCTCGAATTTCCTTGATTTCGGTTCTACAAGACCTTTGAACCTTATAACTTGCTTTTTTTTGTGATTTCTATGCTTAGGCATTTATAAATACATATATTTGACATGATTCAAGCATTTATAAGTTCATTAGAAACAGATGTAGAAAATGGGAATGCTTCTTTCTATTTTGCCCGAATATGCTTGATTTTGGTTCTACAAGCCTCAAAACCTTATAACATCCTTTTTTTGTGATTTCTTTAGTTAGGAAGTTATAAATACATATATTTGACATGAATCAAGCCTTTTCAAGTTCATTAGGAAGAGCATGTAGAAAATGGGAACGCTTCTTCTCATTTTGCCCGAATTTGCTTGATTTCGGTTCTACCTGACCTTAAAACCTTATAACTTGCTTTGTCTTAGTGATTTCTATGCTTAGGAAGTTACTAATATATATATGTGACATGATTCAAGCCTTTATAAATTCATTAGAAAGAGCATGTTGAAAACGGGAACGCTTCTTCCTATTTTGGCCGAATTTGGTTGATTTTGGTTCCACCAGACCTTAAAACCTTATAACATCCTTTTTTTGTGATTTCTTTTGTTAAGAAGTTATAAATACATATATTTGTCATGACTCAAGCCTTTTCAAGTTCATTAGAAAGTGCATGTAGAAAATGAGAACGTTCTTCTCATTTTGCCTGAATTTGCTTGATTTCGGTTCTACCAGACCTTATAACCTTATAAATTGCTTTCTCTTTGTTATTTATTTCTTAGGCAGTTACAAATACATATATTTGACATGATTCAATCCTTTATAAGTTCATTAGAAGGAGCATGTAGAAAATGGGTACGCTTCTTCCCATTTTGGCCGCATTTGCTTGATTTTGGTTCTACAAGACCTTAAAACCTTATAACATGCTTTTTTTTGTGATTTCTTTCTTAGGAATTTATAAATACATATATTACATGAATCAAGCCTTTTCAAGTTCATTAGAAAGAGCGTGTAGAAAATGGGAAGCTTCTTCTCATATGATGTTAATTGGTAACAATAATTTTCATTTTGATGATCTTAAATAGTTAAATGACCTAAAATAATGTCATTTTTCTAAGTGCTTTACTTACTTTGTCTATGCTTTTTGTATCATTTATTTTTTAGTGAAACTGCTATATAATGAGGGAGGATCGAGTATGGATGTTAAACGGTTAGAAGGAAAATTCCTTAGTCCAACCTTTGCGGAAAAGGTCAATGAATTTATTACATTTGCTACCACACAAGACAATGTCGTGATAGATGGTGTGATGAAATGTCCATGTGCACAGTGTCGAAATATTCCTTATCAAGATCTTGATACCATTAAGGAGCATCTCTATAGGCATGGTTTTTTGCCTAACTATTTCCAATGGGTTTTTCATGGTGAACTGCACTTCCAAAGAGAATCCCAAAGTAGCAGCTCCATATCCACCAAGGATGCATTGAACCCATATAGAAACTTGGTATTAGATGCCTTTGGACTAGAAGGTGGTTTAGAAAATATTGAAGAAGAGCCACATGCTAGTTACAAGAAGTTCTTTGACATGTTGAAGGCAGCGGAAGAGCCATTGTATGACGGCTGCAAGTTGTCTGTATTGTCTGCAGCTGCAAAGATGGTAAACATCAAGTGCGAGTATAATATTCCACATAAAGCTATATCCTTAATGAAAGACATGTGCCCAGATGAAAACAAGATGACAGATAATTTTTACAAGACTAAGAAATTGCTTGAAGGGCTAGAACTTCCGCACCAAAAGATTCATGTATGTCCAAATGGATGTATGTTATTTTGGAAGGAACACAAAGATCTCAAAGAATGTCTATATTGCAAAGGAAGTCGTTGGCAACAATTCTCCTCATAGTTCTCTTATTTACTTTCCTACAGGCTCGCGATTACAAAAATTATATGCAACAAGATCTACTGCAGAGCAGATGAGGTGGCATAAAGATAATCCTCGAGTTCATGGCCTTATGTCTCATCCAAGTGATGGAGAGGCGTGGAAACACCTAGATAAAGAGTACCCTTCTTTTGCTGCGGAGCCTAGAAATGTCCGACTAGGTCTTTGTACCGACGGATTTTCACCATTTGGAAAGACAGGAAAGCAATATTCTTGTTCGCCCTTCATTTTAACTCCTTATAATCTTCCACCTGAGTTGTGCATGTTTCTAAGTTTGATAATTCCAGGTCCGAAGAATCCTAAAGGGAATCTCGATGTGTACTTGCGACCTCTTATTGAAGAGTTAAAGCAATTATGGGAGGCTGGGTTACCGACTTACGACATCTCGCAAAAACAAAATTTTCATTTGACTCAATTATCTTTGTATAATGTATTTGACATTCATTTGACTCAATTAGCTTTGTTAATTGCATTTAACTTTCATTTACATCAACAAGCTTTGTTTGAATATGTTTGACTTTTATTTGACTCAATTAGCTTTGTTGAATGCATTTTACTTTAATTTGATTCAAAAAGCTTCGTTTGAATGTGTTTGAATTATTTGACTCAATTAGCTTATTGAATGCATTTGACTTTCATTTGATTCAAAAAGCTTTGTATGAATGTGTTTTACTTTCATTTGACTCAATTATCTTTCTTGAATGCATTTGTCTTTCATTTGATTCAAAAAGCATTGTTTGAATGTGTTTGACTTTCATTTGACTCAATTAGCTTTGTTGAATGCATTTTACTTTCATTTGATACAAAAAGCTTTGTTTGAATGTGTTTGCTTTCATTTGACTCAATTAGCTTTGTTGAATGCATTTGACTTTAATTTGATTCAAAAAGCATTGTTTGAATGTGTTTGACTTTCATTTGACTCAATCAGCATTGTAGAATGCATTTGACTTTTATTTGACTTAATTAGCTATGTATGAATGTGTTTGACTTTCATTTCACTCAATTAGCTTTGTTAATTGCATTTCACTTTTATTTAATTCAAAACGCTTTGTTTGAATGAGTTTCACTTTCATTTGATCAAAAAGCTTTGTTTGAATATGTTTGACTTTCATTTGACTCAATTAGCTTTGTTGAATGCTTTGACTTTCATTTTATTCAAAAAGCTTTGTTTGAATGTGTTTGACTTTCATTTGATTCAATTAGCTTTGTTGAATTCATTTGGCTTTCATTTGATTCAAAATGTATTGTTTGAATGTGTTTGACTTTCATTTAATTCAAAATTCTTGGTTTGAATGTGTTTGACTTTCATTTGATTTAATTAGATTTGTTTAATGCATTTGAATTTCATTTGATTCAAAAAGCATTGTTTAAATGTGTTTGACTTTCTTATGATTCAATTAGCTTTATTGAATGCATTTGACTTTCATTTGATTCAAAAAGCTTTGTTTGAATGTTTTCGACTTTCATTTGCTTTAATTAGCTTGT

| Indices | Period Size | Copy Number | Consensus Size | Percent Matches | Percent Indels | Score | A | C | G | T | Entropy (0-2) |
| --- | --- | --- | --- | --- | --- | --- | --- | --- | --- | --- | --- |
| 6828--7910 | 39 | 27.5 | 40 | 76 | 7 | 916 | 25 | 12 | 14 | 47 | 1.78 |

Consensus pattern (39 bp):

TTTCATTTGATTCAATTAGCTTTGTTTGAATGTATTTGAC


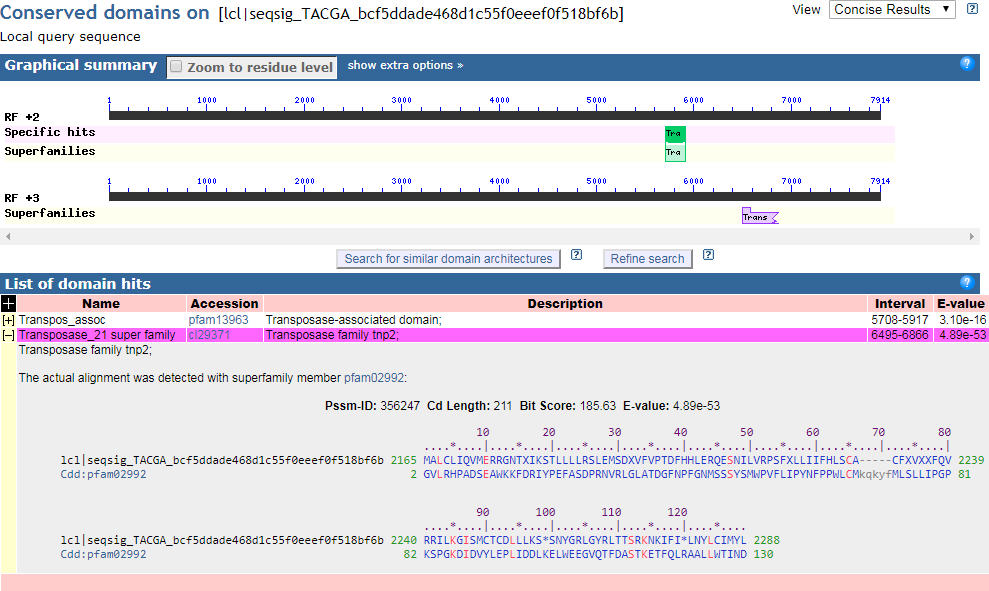


>contig 10973

TCTCTAGTTTTTGATTACTCGATTCTCATAACAAAACTTTATACCATCTATAGATCCTATATAAAAAGTTTAGATGTATATAATACTAGTAGATAACAATAGATGTTAGAGATCCAATGACAACACCAACACCGCCAGGTCTGCCACCAACACACTTTGGCCAAATTAACCAAGATAAAATCTAACACCTGAACCATCAAAACAAATGATATATTCTGCAATGAAATGCTGAGGTAGTTGAAATATATAAAGCCTAGCAAACTTACTTCTTTATTCGATCTTTTAGCTCCGCAATCTTTGGCAGCGGTGTTTTATAATCTATTCCAAACTCCATAGTATCTGCCTGATCTGGACTTCTGTAATAGTTGCCAATGGCCTCGGTTTCGACGCTCTAAGCTCGAATTTCCGACGTCTAAATAAATATTACTCGGTTGACTCATTAATTCCTTCCGAATTAATTCGTCGATTCGGTTTGTTGAATGCTTGGGTGTGACCCGAAGGGTTCAGTCAAGATTAAGCTGTAACATTAATTATCCATTAATATTACTGATCGGAAGCGGCCTCCACCCAGGCATTCCGATCACTTGACCTTACTGAATATATTAGCTTTCTAATAATTCTGAACCCGCTAACGGAATAGTACACTTGTTCGAAGGACACCATTCCTTTCAATAGNTCATTAGAAGGAGCATGTAGAAAATAGGAACGCTTCTTCCCATTTTTGCCGAATTTGCTTGATTTTGGTGTGATGAAATGTCCATGTCCACAGTGTCGAAATATTCCTTATCAAGATCTTGATACCATTAAGGAGCATCTTTATAGGCATGGTTTTTTGCCTAACTATTTCCAATGGGTTTTTCATGGTGAACTGCACTTCCAAAGAGAATCCCAAAGTAGCAGCTCCATATCCACCGAGGATGCATTGAACCCATATAGAAACATGGTATTAGATGCCTTTGGACTAGAAGGTGGTTTAAAAAGTATTGAAGAAGAGCCACATGCTAGTTACAAGAAGTTCTTTGACATGTTGAAGGCAGCGGAAGAGCCATTGTATGATGGCTGCAAGTTGTCTGTATTGTCTATTGCTGCAAGGATGGCAAACATCAAGTGCGAGTATAATATTCCACATAAAGCTATAGATGGTGTTGCTTCCTTAATGAAAGACATGTGCCCAGATGAAATCAAGATGACAAATAGTTTTTACAAGACTAAGAAATTGCTTGAAGGGCTAGAACTTCCTCACCAAAAGATTCATGTATGTCCAAATGGATGTATGTTATTTTGGAAAGAATACAAAGATCTCAAAGAATGTCTATATTGCAAAGGAAGTCGTTATAAGACACTTAGAGAAAGTGGCAACAATTCTCCTCATAGTGCTGTTATTACTTTCCTTAGGCCCGAGATTACAAAGATTATATGCAACAAGATCTACTGCGGAGCAGATGAGGTGGCATAAAGATAATCCTCGAGTTCATGGCCTTATGTCTCATCCAAGTGATGGAGAGGCGTGGAAACACCTAGATGAATAGTACCATTCTTTTTCTGCGGAGCTTAGAAATGTCCGACTAGGTCTTTGTACCGACGGATTTTCACCATTTGGAAAGACAGGAAGGCAATATTCTTGTTGGCCCGTCATTTTAACTCCTTATAATCTTCCACCTGAATTGTGCATGAAGAAACCTTTTATGTTTCTAAGCTTAATAATTCCAGGTCCGAAGAATCTTAAAGGGAATCTCGATGTGTACTTGCAACCTCTTATTGAAGAGTTAAAGCAATTATGGGAGGCTGGGTTACCGACTTAAGACATCTCGCAAAAAAAATATTTTCATTTGACTCAATTAGCTTTGTATAATGTGTTTGACTCTCATTTGACTCAATTAGCTTTGTTAATTGCATTTAACTTTCATTTAATTCAACAAGCTTTGTTTAAATATGTTTGACTTTTATTTGACTCAATTAGCTTTGTTGAATGCATTTTACTTTCATTTGATTAAAAAAGCTTCATTTCAATGTGTTTGAATTTCATTTGACTCAATTAGCTTAGTTGAATGCATTTGACATTCATTTGATTCAAAAAGCTTTGTATGAATGTGTTTGACTTTCATTAGACTCAATTATCTTTATTGAATGCAGTCATTTGATTCAAAAAGCTTTGTTTCAATGTGTTTGAATTACATTTGACTCAATTATCTTTGTTGAATGTGTTTGACATTCATTTGATTCAAAAAGCTTTGTTTGAATGTGTTTGACTCTCATTTGACTCAATAGCTTTGTTGAATGTATTTGATTTCATTTGATTCAAAAAGCATTGTTTAATGTGTTTGACTTTCATTTGACTCAATTAGCTTTATAGATTGCATTTGACTTTCATTTTACTCAATTAGCCTTGTATGAATGTGTTTGACTTTTATTTGACTCAATTAGCTTTCTAATTGCATTTCACTTTCATTTAATTCAAAAAGCTATGTTTGAATGTGTTTGACTTTCATTTGATTCAAAAAGCATTTTTTCAATGTG

| Indices | Period Size | Copy Number | Consensus Size | Percent Matches | Percent Indels | Score | A | C | G | T | Entropy (0-2) |
| --- | --- | --- | --- | --- | --- | --- | --- | --- | --- | --- | --- |
| 1824--2498 | 39 | 17.4 | 39 | 74 | 8 | 545 | 26 | 13 | 13 | 46 | 1.80 |

Consensus pattern (39 bp):

TTTCATTTGACTCAATTAGCTTTGTTGAATGTGTTTGAC


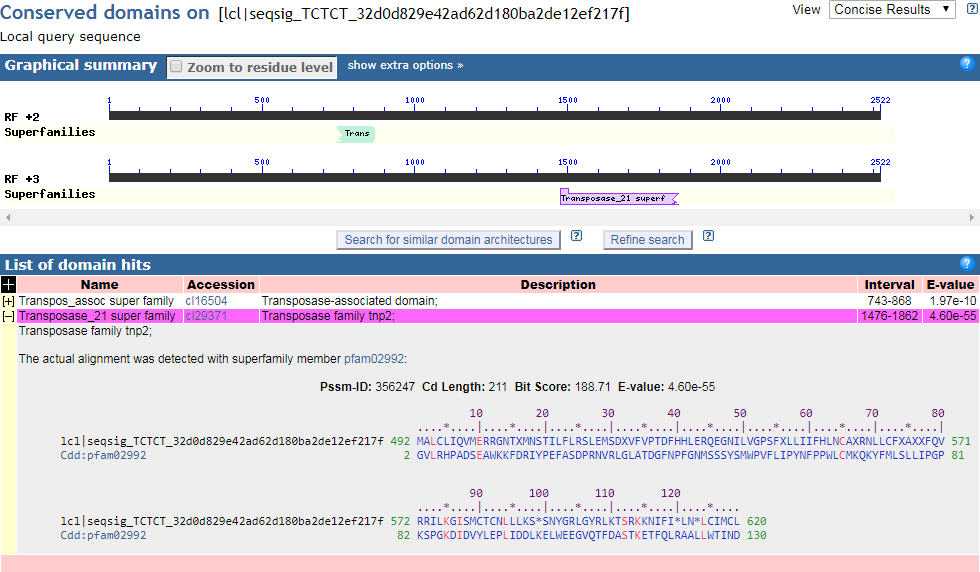


>contig 11346

GGCTAGAACTTCCGCACTAAAAGATTCATGTATGTCCAAATGGATGTATGTTCTTTTGGAAGGAACACAAAGATCTCAAAGAATGTCTATATTGCAAAGGAAGTCGTTATAAGACACTTAGAGAAAGTGGCAACAATTCTCCTCATAGTGCTCTTATTTACTTCCCAATAGGCCCGAGATTACAAAGATTATATGCAACAAGATCTACTGCGGAGCAGATGAGGTGGCATAAAGATAATCCTCGAGTTCATGGCCTTATGTCTCATCCAAGTAATGGAGAGGCGTGGAAACACCTAGATGAAGAGTACCCTTCTTTTGCTGCCGAGCCTAGAAATGTCTGAGACGGATTTTCACCATTTGGAAAGACAGGAAGGCAATATTCTTGTTGGCCGGTCATTTTAACTCCTTATAATCTTCCACCTGTATTGTGCATGAAGAAACCTTTTATGTTTCTAAGTCTGATAATTCCAGGTCCGAAGAATCCTAAAGGGAATCTCGATGTGTACTTGCAACCTCTTATTGAAGAGTTAAAGCAATTATGGGAGGCTGGGTTACCGACTTACGACATCTCGCAAAAACAAAATTTTCATTTGACTCATTACTTTGTATAATGTGTTTGACTTTCATTTGACTCAATTAGCTTTGTAATTGCATTTAACTTTCATTTAATTCAAAAGCTTTATTTAAATGTGTTTGACTTTCATTTGACTCAATAGCTTTGTTGATGCATTTTACTTTCATTTGATTCAAAAAGCTTTGTTTGAGTGTGTTTGACTTTCATTTGAGTCAATTAGGTTTGTTCAATGCATATGACTTTCATTTGAACCGAAAAGCTTTGTTTGAATGTGTTGGCTTTCATTTGACTCAATTATCTTTTTTGAATGCAATTGACTTTCATTTGATTCAAAAAGCTTTGTATGTGTGTTTNACTTTCATTTGACTTAATTATCTTTATTGAATGCATTTGACATTTATTTGATTAAAAAAGCTTTATTTGAATGTGTTTGATTTTCATTTGAGTCTAAAAGCTTTATTGAATGCATTTGACTTTAATTTGATTCAAAAAGCTTTGTTTGAACGTGTTTGACTTTCATTTGACTAAATTATCTTTGTTGAATGCATTTGACTTTCATTAAATTAAAAAAGCTTTGTTTGAATGTGTTTGACTTTCATTTGACTCAATTATCTTTGTTGTATGCATTTTACTTTTAATTCGACTTTCATTTGCTTAAAAAAGCTTTGTTTTAATGTGTTTACTTTCATTTGATTCAAATAGCTTTGTTTAATGCATTTGATTATCATTCGCTTTAAAAAGCTTCGTTTAAATGTGTTTGACTTTCATTTGATTTAAAAACCTTTTTTTGAATGTATTTGACTTTCAGTTGATTCCAATAGCTTTGTTGAATACAATAGACTTTCATTTGCATCAAAAAGCTTTGTTTGAATGTGTTTAACATTAATTTGATATAAAACCTATGTTTGAATGTGTTTGACTTTCATTTGATTCAAATAGCTTTATAGAATACATTAGACTTTCATTTGATTCAAAAAGTCTTGTTTGAGTGTGTTTGACTTTCATTTGATTCAAGAAGCTTTGTTTGAATGTATTTGACTTTCATTTGATTCAAAAAGATTTGTTTGAATGTATTTGACCTTCATTTGATTCAAATAGCTTTCTGGATTGCATTTACCTTTTATTTC

| Indices | Period Size | Copy Number | Consensus Size | Percent Matches | Percent Indels | Score | A | C | G | T | Entropy (0-2) |
| --- | --- | --- | --- | --- | --- | --- | --- | --- | --- | --- | --- |
| 602--1676 | 39 | 27.1 | 40 | 73 | 8 | 802 | 26 | 11 | 14 | 46 | 1.79 |

Consensus pattern (40 bp):

TTTCATTTGATTCAAAAAGCTTTGTTTGAATGTATTTGAC


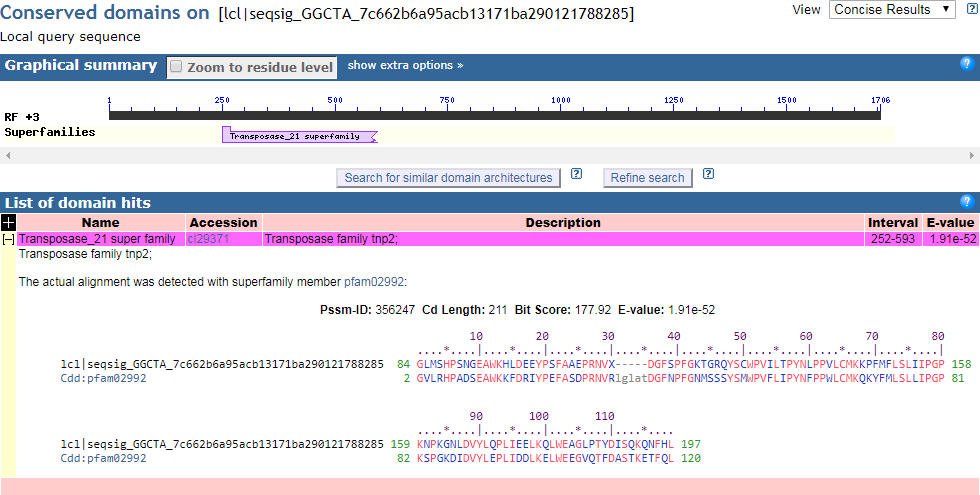


*C. album*

>contig 1990

GAATAATGCTACCTTTCACTTTAGACTTCGACCTAGCTTAGGCGTTAATTAGAAATACTTGAACATAACCGCTCCTAGTGGTTCGACCCTTGACTTACCTCATCTTGTATTTAGGTAGTTGAGTGATTATAAATTTGTTTGACCGTAGGGAGGATTAGTTCACGACATAGTTTTCCCCACGTCAAAACCTTATAACTTGCTTTGTTTTTATGAAGTTACAAATAAATATATTTGACATGACTCAAGCATTTATAATTTCATTAAAAAGAGCATGTAGAAAATGGGAACGCTTCTTACCATTTTGCCCGAAAATGCTTGATTTTGGTTCTACAAGACCTTAAAACCTTATAACCTTCTTTTTTTGTAATTTATTTTGTTATGAATTTATAAATACATATATTTGACATGCCTTAAGCCTTTTCATGTTCATTAGGAAGAGCATGTTGAAAATGGGAATGCTTTTTCCCATTTTGCTCGAATTTGTTTGATTTCGGTTCAACAAGACCTTAAAACCCTATAACTTGCTTTTTTTGTGATTTCTATGCTTAGGGAGTTATAAATACATATATTTGACATGATTCAAGCATTTATAAGTTGATTTCAAAGAGCATGTAGAAAATGGGAACTTCTTAATTTTGCCCGAAAATGCTTGATTTTGGTTCTAAAGACCTTAAAACCTTATAACATCCTTTTTCTTTTATGATATATTCTGTTATGAAGTTATAAATACATATGTTTGACATGACTCAAGCCTTTTCATGTTCATTAGAAAGAGCATGTTGAAAATGTCAATGCTTTTTCCCATTTTGCTCGAATTTCTTGATTTCGGTTCTACAAGACCTTAAAACCTTATAACTTGATTTTTTTGTGATTTCTATGCTTAGAGTTATAATACATATATTTGACATGATTCAAGATTTATAAGTTCATTAGAAATAGCATGTAGAAAATGGGAGTGCTGCTAACTATTTTGCCTGAATATGCTTGATTTTGGTTCTACAAGACCTTAAAACCTTTTAACATCCTTTTTTGTGATTTATTTAGTTAGGAAGTTATAAATACATATATTTGACATGAATCAAGCCTTTTAAAGTTCATTAGAAAGAGCATGTAGAAAATGAGTACGCTTCTTCTCATTTTGCCCGAATTTGCTTGATTTCGGTTCTACCAGACCTTAAAACCTTATAACATCCATTTTTTTTTATGATTTATTCTGTTATGAAGTTATAAATACATATGTTTGACATGACTCAAGCCTTTTCATGTTCATTAGAAAGAGCATGTTTAAAATGGGAATGCCTTTTCCCATTTTGCTCGAATTGCCTTGATTTCGGTTCTACAAGACCTTAAAACCTTATAACTTGCTTTTTTTTTTGTGATTTCTATTATTAGGCAGTTATAAATACATATATTTGACATGATTCAAGCATTTATATGTTCATTAGAAAGAGCATGTAGAAAATGGGAACGCTTCTTCCCATTTTGGCCGAATTTGCTTGATTTTGGCTCTACAAGACCATAAAACCTTATAACATGCTTTATTTTTGTGATTTATTTTATTAGGAAGTGATAAATACCTATATGTGACATGAATCAAGCCTTTTCAAGTTCATTAGAAAGAGCATGTAGAAAATGGGAACGCTTCTGCTCATTTTGCCCGAATTTGCTTGATTTCGATTCTACCAGACCTTAAAACCTTATAGCTTGCTTTTTTTTTTTGTGATTTCTATGCTTAGGCAGTTATTAACTACATATATTTGACATTATTCAAGCATTTACACGTTATTAGAAAGAACATGTAGAAAATGGGAATGCTTCTTACTATTTTGCGTGAATTTGCTTGATATTGGTTCTACAAGACCTTAAAACCTTAGTACATGCTTTGTTTTTGTGATTTATTTTCATAGGCAGTTATAAATACATATATTTGACATGAATCAATCCTTTTCAAGTTCATTAGAAAAGCGTGTAGAAAATGGGAACGCTTCTTCCATATGATGTTAATTGGTAAAAATAATTTTCATTTTGATGACCTTAAATAGTTAAATGACCTAAAAGAATGTCATTATTCTAAGTCTTACTTACTTTGTCTATGTTTTTGTATCATTTATTTTTTAGTGAAACTGCTATATAATGAGGGAGGATCGAGCATGGATGTATAAACGGTTAGAAGGAAAATTCCTGAGTCCAACCTTTCTGAAAAGATCAATGAATTTATTACATTTGCTACCACACAAGACAATGTCGTGATAGATGGTGTGATGAAATGTCCATGTGCATAGTGTCAAAATATTCCTTATCAAGATCTATACCATTAAGGAGCATCTCTATAGGCATGGTTTTTTGCCTAACTATTTCCAATGGGTTTTTCATGGTGAACTGCACTTCCAAAGAGAATCCCAAAGTAGCAGCTCCATATCCACCGAGGATGCATTGAACCCATATAGAAACATGGTATTAGATGCCTTTGGACTAGAAGGTGGTTTAGAAAATATTGAAGAAGAGCCACATGCTAGTTACAAGAAGTTCTTTGACATGTTGAAGGCAGCAGAGAGCCATTGTATGATGGCTGCAAGTTGTCTGTATTGTCTGCTGCTGCAAGGATGGCAAACATCAAGTGCGAGTATAATATTCCACATAAAGCTATAGATGGTGTTGCTTCCTTAATGAAAGACATGTGCCCAGATGAAAACAAGATGACAGATANTTTTTAGAAGATTAAGAAATTGCTTGAAGGGCCAGAACTTCCGCACCAAAAGATTCATGTATGTCCAAATGGATGTATATTATTTTGGAAGGAACACAAAGATCTCAAAGAATGTCTATATTGCAAAGGAATTCGTTATAAGAAACTTAGAGAAAGTGGCAACAATTCTCCTCATAGTGCTCTTATTTACTTTCCTATAGGCCCGAGATTACAAAGATTATATGCAACAAGATCTACTGCGAAGCAGATGAGGTGGCATAAAGATAATCCTCGAGTTCATGGCCTTATGTCTCATCCAAGTGATGGAAAGGCGTGGAAACACCAGATGAAAATTACCCTTCTTTTGCTGCGGAGCATAAAAATGTCCGACTAGGTCTTTGTACCGACGGATTTTCACCATTTGGAAAGACAGGAAGGCAATATTCTTGTTGGCCCGTCATTTTAATCCTTATAATCTTCAACCTGAATTGTGCATGAAGAAACCTTTTATGTTTCTAAGTTTGATAATTCCAGGTCCGAAGAATCCTAAAGGGAATCTCGATGTGTACTTGCGACCTCTTATTGAAGAGTTAAAGCAATTATGGGAGGCTGGGTTACCGACTTACGACATCTCGCAAAAACAAAATTTTCATTTGACTCAGTTAGCTTTGTATAATGTATTTGACATTCATTTGACTCAATTAGCTTTGTTAATTGCATTTAACTTTCATTTACATCAACAAGCTTTGTTTGAATATGTTTGACTTTTATTTGACTCAATTAGCTTTTTGAATGCATTTTACTTTATTTGATTCAAAAAGCTTCGTTTGAATGTGTTTGAATTTCATTTGACTCAATTAGCTTAATTGAATGCATTTGACTTTCATTTGATTCAAAAAGCTTTGTATGAATGTGTTTTACTTTCATTTGACTCAATTATCTTTTTTGAATGCATTTGTCTTTCATTTGATTAAAAAAGCATTGTTTGAATGTGTTGGACATTCATTTGACTCAATTAGCTCTGTTGAATGCATTTTACTTTCATTTGATACAAAAAGCTTTGTTTGAATGTGTTTGACTTTCATTTGACTCAATTAGCTGTGTTGAATGCATTTGACGTTAGTTTGATTCAAAAAGCATTGTTTGAATG

| Indices | Period Size | Copy Number | Consensus Size | Percent Matches | Percent Indels | Score | A | C | G | T | Entropy (0-2) |
| --- | --- | --- | --- | --- | --- | --- | --- | --- | --- | --- | --- |
| 3334--3804 | 39 | 12.0 | 39 | 75 | 5 | 414 | 26 | 12 | 14 | 46 | 1.80 |

Consensus pattern (39 bp):

TTTCATTTGACTCAATTAGCTTTGTTGAATGCATTTGAC


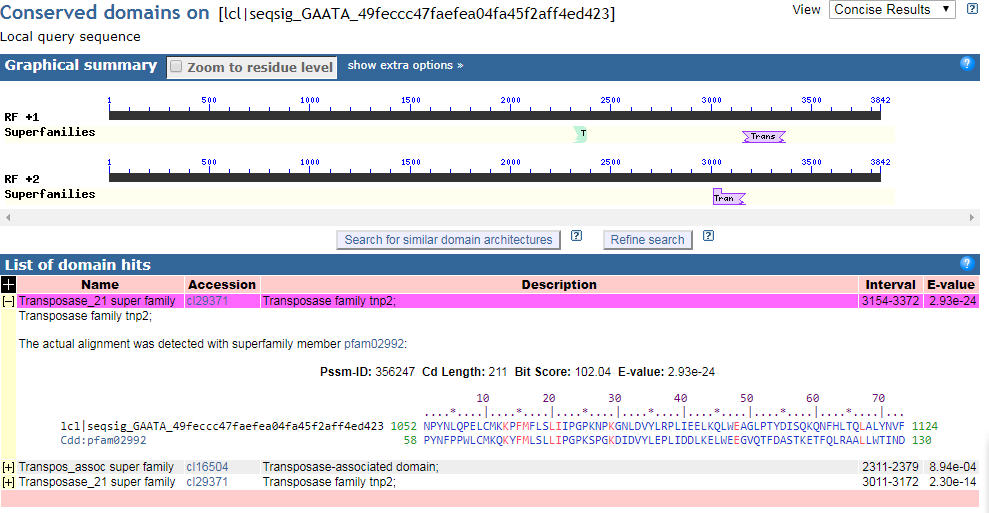

Supplement: Supplementary file 1 — Additional file 1: Contig showing the association of tnp2B and CficCl-61-40 satDNA family arrays. [file 13100_2020_219_MOESM1_ESM.docx]
