# Supplementary material for: Transposons and satellite DNA: on the origin of the major satellite DNA family in the Chenopodium genome
Source: Mob DNA. 2020 Jun 26;11:20. doi: 10.1186/s13100-020-00219-7 (PMC7320549; doi:10.1186/s13100-020-00219-7)
Supplement: Supplementary file 2 — Additional file 2: 1. Consensus monomers of the CficCl-61-40 satDNA family and corresponding fragments of tnp2 for species of the C. album aggregate. 2. TPase domains of putative CACTA-like transposons detected in the genomes of the species of the C. album aggregate in comparison with similar domains of other species. [file 13100_2020_219_MOESM2_ESM.docx]

**Additional file 2**

1. Consensus monomers of the CficCl-61-40 satDNA family and corresponding fragments of *tnp2* for species of the *C. album* aggregate.

....|....| ....|....| ....|....| ....|....|

5 15 25 35

********** * * * * *** * ** ** * ****

C. acerifolium TTTCATTTGA TTCAAT-AAC TTTGTT-GAA TGTATTTGAC

C. acuminatum TTTCATTTGA TTCAAAAAGC TTTGTT-GAA TGTATTTGAC

C. album TTTCATTTGA TTCTAAAAGC TTTTTT-GAA TGTATTTGGC

C. bryoniifolium TTTCATTTGA TTCAATAAGC TTTGTT-GAA TGCATTTGAC

C. ficifolium TTTCATTTGA TTCAAAAAGC TTTGTTTGAA TGTGTTTGAC

C. iljinii TTTCATTTGA TTCAAAAAGC TTTGTTTGAA TGTGTTTGAC

C. jenissejense TTTCATTTGA TTCAAAAAGC TTTGTTTGAA TGTGTTTGAC

C. karoi TTTCATTTGA TTCAAAAAGC TTTGTTTGAA TGTGTTTGAC

C. luteorubrum TTTCATTTGA TTCAAATAGC TTTGTT-GAA TGCATTTGAC

C. novopokrovskyanum TTTCATTTGA TTCAAAAAGC TTTGTT-GAA TGTGTTTGAC

C. opulifolium TTTCATTTGA TTCAATAAGC TTTGTT-GAA TGTGTTTGAC

C. pamiricum TTTCATTTGA TTCAAAAAGC TTTGTTTGAA TGTGTTTGAC

C. sosnowskyi TTTCATTTGA TTTAATTAGC TTTGTT-GAA TTCATTTGAC

C. striatiforme TTTCATTTGA CTCAATTAGC TTTGTT-GAA TGTGTTTGAC

C. strictum TTTCATTTGA TTCAAAAAGC TTTGTTTGAA TGTGTTTGAC

C. suecicum TTTCATTTGA TTCAAAAAGC TTTGTTTGAA TGTGTTTGAC

C. vulvaria TTTCATTTGA TTCAATTAGC TTTGTT-GAA TGCATTTGAC

tnp2 C. acuminatum TTTCATTTGA CTCAATTATC TTTGTA-TAA TGTATTTGAC

tnp2 C. stratiforme TTTCATTTGA CCCAATTAGC TTTGTA-TAA TGTGTATGAC

tnp2 C. strictum TTTCATTTGA CCCAATTAGC TTTGTA-TAA TGTGTATGAC

1. TPase domains of putative CACTA-like transposons detected in the genomes of the species of the *C. album* aggregate in comparison with similar domains of other species

CLUSTAL multiple sequence alignment by MUSCLE (3.8)

tnp2A_O. brachyantha DGMLRNPADSMQWRNIDRIYP-QFAEDSRNMRVCLCTDGMNQFGDMSSRHSTWPVLIANY

tnp2A_pamiricum1 --MLKHPADSPQWKNIDLKFE-KFGDEVRNLRLGLCTDGMNPFGTLSTQHSTWPVLLVIY

tnp2A_soybean DGMVRHPADCSQWKKIDSLYP-NFGKEARNLRLGLASDGMNPYGNLSTQHSSWPVLLVIY

tnp2A_jap-rice DNELSHPADGEAWKDFDRKYE-WFANDARNVRLGLATDGFNPFGKMSSSYSMWPVFLIPY

tnp2A_vulvaria DHLLHHPVDGQAWKEFDSLYT-KFADDPRNVRLGLATDGFSPFNSMSIVHSTWPVMLINY

tnp2A_pamiricum DKMLRHPSDGEAWKKFDEKYT-EFAADPRSVRLGLASDGFNPYRLMNTNYSTWPVVLIPY

tnp2A_potato DGLMRHPRDGEAWKTFDRTHS-GFASDPRNVRLGLASDGFNPFGTMSTTYSIWPVFLIPY

tnp2_aegilops DGIMRHPADSLAWKHFDNIYSKGFSSDARNVRLGLASDGFNPYGIMNVSYSCWPVILIPY

tnp2A_Amborella DGVLRHPADAEEWKQFDRLHP-SFAVEPRNVRLGLATDGFNPFGNMSNSYSLWPVICVPY

tnp2A_acuminatum -GTMAHPSDSEAWKHLDSCFP-DFASEPRNVRLGLCTDGFAPHGQFGGQYSCWPVILTPY

tnp2B_novopokr -GLMSHPSDGEAWKHLDKEYP-SFAAEPRNVRLGLCTDGXSPFGKTGXXYSCWPVILTPY

tnp2B_acuminatum ----------------------SFAAEPRNVXIGLCTDGFSPFGKTGRQYSCWPVILTPY

*. : *.: : *.:** . . :* ***. *

tnp2A_O. brachyantha NFPPWLCFKRKYIMLCLLIQGPRQPGNDIVVFLEPVGDDLEILWNEGVQTSDSYGREQFN

tnp2A_pamiricum1 NLPPWLCMKRKYVMLSLLITGPRQPGNDIDVYLAPLIDDLRKMWDEGVSVFDADQNEMFT

tnp2A_soybean NFPPWLCMKRKYMMLSMMISGPRQPGNDIDVYLSPLIEDLRKLWDEGVLVFDGFRKETFQ

tnp2A_jap-rice NFPPWQCMEQSNFMMCLLILGPTCPGKDMDLFLQPLVEELLNLW-SGVPTLDALTGKEFD

tnp2A_vulvaria NLPPWMIMKPEYLMLALLVPGPSSPGNDIDIYLQPLIKDLKDLWEFGLETYDASSNQRFD

tnp2A_pamiricum NLPPWLCMKSSSFILSIIIPGKFGPGMDIDVYLQPLIHELKLLW-VGVGAFDSYSGSNFK

tnp2A_potato NLPPWMCMKHTSFILSMIIPGKQMPGNNIDVYLQPLVKELCELWNDGVETFDSSLNETFR

tnp2_aegilops NLPPWLCLKQPYWFMSMIIPGKKSPGNNIDVYLQPLIDELKDLWYVGADTYDATTKKNFQ

tnp2A_Amborella NLPPWKCMSSESLLLTLLIPGPSSPGKDIDVFMRPLIDELKQLWETGVETRDAYNGTVFS

tnp2A_acuminatum NLPPSMCMKRQFMFLSLLVPGPKNPKGNLDVYMQPLIDELKQLWEVGANTFDISRKQNFN

tnp2B_novopokrov XLPPELCMKKPFMFLSLIIPGPKNPKGNLDVYL-PLIEELKQL--EVLPTYDISQKQNFQ

tnp2B_acuminatum NLPPELCMKKPFMFLSLIIPSPKNPKGNLDVYLRPLIEELKQLWEAGLPTYNISQKTKFS

:** :. :: ::: . * :: ::: *: :* : . : *

tnp2A_O. brachyantha LRVLLFGTINVWPALGNLSGQSIKRMNAGINCKKNTRSLCLKHSRKMV-YLGHRRWLPIR

tnp2A_pamiricum1 LRAALMWTMNDFPAYGNLCGYKNKGHKACPICVDDTPNVYLDHYGKDV-YVRTRRFLRRD

tnp2A_soybean MRAMLFCTINDFPAYGNLSGYSVKGHLACPICEEDTSYIQLKHGRKTV-YTRHRVFLKAH

tnp2A_jap-rice LHAAIIWCIHDYPALSTLSGRVTRGYYACVCC--DKNPCYKRLRNKIC-YIGHRRFLPVD

tnp2A_vulvaria MHVALMTTVSDFPAYAMLSGWSTKGYLACPECHYETDSERLPCSGKNV-YRANRRFLDIS

tnp2A_pamiricum MRAALHSTINDFPAYAMLSGWSTKGYKACPSCTHST--YSDRFGGKII-YPGYRKWLPID

tnp2A_potato MHAALMWTISDFPGLGILSGWNTHTGFACPTCNFDTEPCRLVHSKKWC-FMGHRRFLRRN

tnp2_aegilops MHAALMWTINDFPAYAMLSGWSTKGKLACPYCHMHTDHLWLKYGRKYC-YMGHRRFLSRD

tnp2A_Amborella MRAAVLWTINDFPAYALMSGWSTKGYMACPTCNEHTPSIGLN--SKIG-YVGHRRFLEMS

tnp2A_acuminatum LRAAILWTVSDFPAYGMLSGWTTAGKKACPYCMEKSKAFWLEHGGKVSWFDCHRQFLPTD

tnp2B_novopokr LKA-LLWTISDFPAYGMLSGWTTSGRLACPYCMENTKAFTLKNGGKQSWFDCHRQFLP-D

tnp2B_acuminatum ------------------------------------------------------------

tnp2A_O. brachyantha HRYRRMKRSF-NGKNELLPAPKTLTGKEVYDMVQ

tnp2A_pamiricum1 HPYRRQRKAF-NGKVEEDAAPRPLSGKEVYARVK

tnp2A_soybean HPYRRLKKAF-NGSQEHEIRRTPLTGEQV-----

tnp2A_jap-rice HIWRRKKD-F-NGQTEERAQPEEFTQDELMQQLA

tnp2A_vulvaria HPWRHDKRNF-DGKLEERYQPIPLNGIDIENML-

tnp2A_pamiricum HPYRSQANLF-DGKEEYGIAPIRTSGAEVLKQ--

tnp2A_potato HRFRFNRVRF-NGSTEERNPPIKLSGSDILRQIA

tnp2_aegilops HKWRRNKSCF-NNETENRDAPVPLSGNDVVQQHA

tnp2A_Amborella DPRRRSKKV--HGKTEKRAPPPVMTGDDILTQLD

tnp2A_acuminatum HPFRNSKTAFCKNKVEKGAPPHIMTGEELWECVK

tnp2B_novopokr HNFRKKSAFF-KDRVENDSPPR-MSGEDINRV--

tnp2B_acuminatum ----------------------------------
