# Supplementary material for: Transposons and satellite DNA: on the origin of the major satellite DNA family in the Chenopodium genome
Source: Mob DNA. 2020 Jun 26;11:20. doi: 10.1186/s13100-020-00219-7 (PMC7320549; doi:10.1186/s13100-020-00219-7)

**Additional file 3**

Characteristics of the putative CACTA element *Jozin* from the genome of *C. pamiricum*

**Characteristics:**

Length: 8380 bp

TIRs: 28 bp cactagtagaaaaaacgtcatttgtaac (highlighted yellow)

Sub TIRs: 5’ 270 bp, 3’ 350 bp

ORF: position 1303-4599; 3297 bp | 1098 aa

Conserved domains (highlighted red):

Transposase associated: position 1309-1560 bp, 251 bp;

Transposase tnp2: position 2206-2838 bp, 632 bp;

DUF 4218: 3382-3723 bp, 341 bp;

DUF 4216: 4201-4410 bp, 209 bp

Sequence:

CACTAGTAGAAAAAACGTCATTTGTAACCCCCTATTTGTAACTAACAATACTTTTTTAGTTACAATTAATATATTTGTAACAAACAATTAAAAAATCCGTTACAAATAATTAGTAACAACTTGTATGACTCATGTAATCGTAACTAACTAAATTTGTTGGTTACAAGTAATTGTTATTGTAACTAACAATTTAATTGACTGTTACGACTACTATACTTATATTTTCTGTAAATATATATTTGTAACTATCAACACATAGTTGGCTACAAATACTAATTTCATTTTATTTTAATTTATTTTTTCCAATAAAGAAATAGAAAAAACTAAGCCCCATCAAAATAAAAAAACCCCTTGACCTCCCCTATTACCCCCAACCTTAGTCTAAAATTCCCCCCAAAATAAAAAAAACTCCCACCAAAATTCCCCCCAATTAACCCAGAAAACTTGAAGACCACGACACCCAAATTGGGCTTGAAGACTGCCATCAACCTGCACTTGAAGACCGCCGAAGTTCAACCGGAGTTTTGCCTTCCACAATCGCTGGAGTTCCGCTACAGTTCTACAATCGCTGGAGTTCCGCTACAGTTCTACAGTCGCTGGAGTTGAAGAGCGCTGGAGTTCCGTCGGCTAGTACGACGTTTAGCCACCGTCACGCAGCTTCAATTCAACACTTATTAGGGTATGTTCGAGAATTATTTGGGGTTTTTTTTACATGCAAATTGGGATCTTTTACTTCTTTGGCTGTTGTACTCTAGAAATCCTAGGGCTATGCTTATTTATTTTTGCAAGTTCTTATTTGTTTTTTACATTGAATTCAAATTTCTGTTTCAGTTTTTTGTCTCAATTTTCTATCTATATTGCATTTAAATACCTATTTCAATTTTGTGTCTCAATATTTTGTTGATAGTTTCTGTTTATATTGTATTTAGCTATTGTATTTGAATTATATTTCGGGAGTCTCAATTATTTTTTGGGTTAAGATCTTTGAGGTGTAGTTGTGTAATTTAATTTAATATAATTTAATTTTATAGGGTTAATCTCTTGATTTTTGGGTTATGTAATGTTTTTGAGTGCTTTGATGAGCTTGGTTTTAAGTGTTATATGTGTTATCTCACTGTGAGGTGGATTTTGTCGATCTTGGGTTTCTGACATTTCAGATATAAAGTCCATCTGCCTTAATTAGTGTGTGTTATATCATAGTGTATACTTAGTCGTTCAAATTCTAGTTATGAATCACGTCATAACTGGGTAGATACTTTGCTTTTTTTGGTGTTGAATATAACATGTTATTACATTTTAGAAATGAATCGTGAATGGATGTATGATGCAATCCGAACTTCACCTGCATACTTGGAAGGAGTTAATGAGTTTTGTAAAATTGCTGTGCAACATCAGTGGAGTGTTGTTGTTGAACAAAGACTTACTAGGGCAAGACCTATCTTCTGCCCTTGTTGTCATTGCAAGAACGTGAAGAGGTGGGATGACATTAAGAAAATAGAGGAACATTTGATTATTCATGGGTTCATGTCAGACTACACAGTTTGGTACTGGCACGGTGAAAAACTTACTCCTATGGACAGTTCATCTTCTATTAATAATGATATCGAACTTGATAGGGAGTCGGGTGGTAGTGATAATAATGATGGGTTTGATCATGAAAACAATGTTGATGAAGATAACATCAATGATATGATGGAAGGGTTGGAGGATCGTGTGAATGAAAATTCTCGTACAATTGAGGAGGTGTCAAAGGCTGCAGAGACACCACTATATCCTGGTTGTACAAAGTATTCCAAGCTTTCGGGGGTGTTAAAATTATTCAACTTGAAAGCCAAGAGCGGTTGGACTGACACGAGTTTCACTTTGTTGTTAGAAACTCTTTCGGATATGTTCCCCGAGGGGAATGACATTCCTAAGTCCACCTATTATGCGAAGAAATTGATGTGTCCCATGGGTTTGGAGTACACCAAGATCCATGCATGTCCTAATGACTGTGTGCTTTTTCGAAAGGAGAATGAAAACTTAGATGCTTGTCCACAGTGCGGTGAGTCTCGATACAAGCGGGAGGGTTTAAACTTGGACCGTAAGAAGTGGCCGCCAGCTAAGGTCGTATGGTATCTTCCAATAATTCCAAGGTTTAAGCGTCTGTTTTCAATCAAGCAAGATGCTAAAAACTTGGTGTGGCATGACAATGAGAGGAAGAAAGATGGGTTAATTAGACACCCGGCTGATTCGTTGCAATGGAAACACATTGATGAGACATTTCCAGAGTTCGGTAATGAGCCTAGGAACTTGAGGCTTGCGTTGAGTACAGACGGGATGAACCCTTATGGCACTTTAAGTAGCAAACATAGCACATGGCCAGTTCTTTTGTCGATATACAACTTACCTCCTTGGTTGTGCCAGAAGCGAAAGTACATCATGCTGTCACTGTTAATATCAGGTCCTAAACAACCTGGTCATGATATAGATGTGTACTTAGAGCCACTCATAGATGATTTGAAGCTGTTGTGGGATGAAGGTGTTGTGACATTCGATGCTCATACCGGGACGGATTTCAGACTAAGAGCCATGATTTTCTGTACCATTAATGATTTTCCGGCGTATGGAAATTTGTCGGGGTACAAAAACAAAGGACAGAAAGCATGCCCAGTATGTGAAGATGATATGCCAGTTACATACCTGAAACATTGGGGTAAGAATGTGTATTTGCATACTCGAAGGTCACTTCGTCGAGACCATCCATATCGAAAGATGAAGAAACAATTCAACGGGTTTACTGAGAAAGGAGTGTGTCGTAAGCCATTGACAGGGACCCAGGTGTATGAAAGAATTAAACATATCAACACAGTCTACGGAAAGCTTCACACACCAAAACCAAAGAAGGGTGTTCTATGGAAAAAAGTGTCTAAGTTATGGGAACTTCCTTATTGGAAGCACTTAAGAGTTAGACATTGTCTGGATGTCATGCATATTGAAAAAAATGTTTGTGATTCATTGATTGGGACATTGTTGAATATTAAGGGGAAGACCAAGGATGGTGCTACTGTGCGTAGAGACATGAAGGAAGCCAATGTTCGACCAAAGTTATGGCCCGAGGATAATGAAAGCAAGAAGAAATCATTCTTGCCTCATGCGTGCTACACATTGTCTAGGGAAGAGAAGAGGATTTTTTGTGAATGTTTGAAAGGAATCAAGGTACCTACGGGGTATTCGTCGAATGTGAGCCGTTTTGTTTCGGTAACCGATAAGAAAGTAACTGGTATGAAATGTCATGATTGTCATGTCATGATGCAAGTATTCTTACCAATTGCTATTCGGGGGCTTTTGCCAAAACATGTTAGGTATGCCGTTGTGAAGCTTTGTGACTTTTTCAGTGACATATGTAGTAAGGCAATCGATCCCAAAAGGCTTGATGAAATGGAAGCTGATGTTATTGAAACCTTATGCAAGTTCGAGATGTATTTTCCACCCTCATTCTTTGATATAATGGTACATGTAGTTATCCACTTACCTCGAGAGATTAAAGAATGCGGTCCTGTGTTCATGCATTATATGTACCCTTATGAAAGGCACATGGGAAGTTTGCAAGATAAAGTTAAAAATAGAGCAAGTCCCGAGGGTAGTATCATTCAAGCTACCGTGGCAGAGGAAGCTGGGAACTTTTGTGCCGTGTTCTTGGCTAGGGCTAAAGAAATTGGTGTTCCCATATCTCGACATGAGGGAAGGCTGCAAGGGAAGGGTACAGTTGGTCGAACATTGGTCAGGCCTCCGATTGACAGATTTCAAAAGGCCCATCGATATGTTTTGCAACAGCTCTCTGAAGTTCATCCATACATAGAAAAGCATCAACATGAATTGCAACGTCAAAATCCAAGAATAACTGCTTATTCATTGATGCAAGAGCATAATCGCCGGTTTGTTGAGTGGTTTCAAGTCCAAGTGAAGCTTCAATTGAGTCAAAATGAAAATGTACCAGAAATGATCAAGTGGCTAGCACGAGGTCCCCAACCACTTGTTTATACTTATGAGAGCTATGACATCAATGGATATTCTTTCTCTACTTATCTTAAGGATAAAAAGAGTGTTCAGCAAAATAGTGGCGTTGTTGTAGTTGCATCTTCAACAGAATACTCAAGTGCTAAAGATACAAGGCCAACCAATGCGACACAAGCATATTATGGGATTATTCAAGAGATTTGGGAGCTAGACTATGTTGATTTCACCATTCCTCTTTTTCGATGCAAGTGGGCTGACAATCGTCGTGGACATAAAAGGGATGTTCTGTTTGGTTATACTTTGGTAGATTCAAGTCGTTATATAGAAGGTGAGGAGCCTTTTATCTTAGCAAAACAAGCTAAACAGATTTTTTACATCAAAGACAATATGGATCCAATGTGGCGTGTTGTGGTTCAAGGAAAAAGACGAATTGTTGGTGTTGAAGATGTCGTAGATGAAGAAGAGTATGACAGATTGGATGACACCCCACCTTTATCCATGGGTGTCCAACCTCTTCAAGATGGAGAAGATATAGTTGATGATGAGGACTATGATCGTGACGAAGGGGTGGAGGTTGATTTAGTTACGACACCACTATAGGGTTTATGTTAGTATTATTTTGCTATGTTAGCTTAGAAATTCTGTAATATTAGCAATGAACTTGGTTTCTCTCTCTTTTTTTTTGTTTTTTTTTCTCCAGATATTTTAAATTGATCATAGTAATTTGTGCAATCTATTGCAATTAGTTTCTGTCCTAATTTTCATCATTTGCACCATACCTAATTATATTGTTGTACATAATTAGTGGGAATACCTTTGCTGAGAATGCGTGCTTCTGCGAGCTTATACATTTCATCTTACCCTTTTGTAATTGTATGCATGCAGTGTGTAAGACAACTATCCCCGAGAAAGCAGATTTTATGCGGAACTTCCTTGAGATCTCTCTAGCAGCTACAGATCTTAGTGCTGTATCCTGGTGACCTGGTAAGTAGAGGATAGTCAAATCATTGTCACTTAAGTATATATACACAGGCATAAATTAGCAACATTTCAATATTTATCTGCTTATGATTTTCAACACCCCGTGCCTGAGGGAGAAATGCTATCTTGATCTGTATGTTAAGCTAGCTGTATGTTAAGACTTAAAACTGAGATAGTATAGAGACACAAATGTACCAGTGTTAAAGGATTTTTATGCCAGAAAACCTAACATACACCAAAGTTATGATATCAGGATTCGCGCTCTGCAGCCTTCTTCTTGTGACGAATCTAATATTTTCAGCAATGATACATGAATCAAATAATTTATCAAATTTTAATATATGAAAAGTTTCTGTTGGTCTATGTTTTTAATATATGAAAAGCGGTGTTATATTTATTCAACCCAAGTTATGTCTATAATGTTTAATTTTGTAAGCCATTCCTGTACTTAAGACTCTTGGTCTATATTCATTTTTCTTTCTTTAGCTTTATCAGAAATACACAAGGTTTATATTCTGTACTTTCTTCAGCTTTATCACTATCAGATGAACTATTATCATTTTTCTTGTCACTGGAAAGACTTGTCTGCTGCCTGTTGGGGTGCCTTAATTTGAGTGATAGAAGACGGGATACTTCATGTAGGCCGCCCCATTTTTCAAGGGCACGGGCAATGTCATACCGTCCTGCAAAAATAAAATTCTTCTCAGTTTTTAATATCTTACAGGAAACTAAACCAAAAGCCTACCAAAAAGGCAATAAGTGATTTTTTTTCTTTTGGAAACAAAGCTGTCCAAATATATACAGGTTGGGAAGTAATTAAGCAATATTAGTTTGACCTGCGCGTTCAAATGCTTTTCTGCTGGGCATATATGATGGATCCATCCCCCAGTACCTTAGCAACTCCAACCCACCATCATTCCCTTCAAGTCCCGTTATCTTACCTATTATTTAATAAGCCTAGAAGTATAATCTGTTTTTACATTCACGTAAATCATAGAAGAAATTTCTATGAAATACATTTGTTATCTGAAACAATAGGTTGTCATGTAAAAGGCTGTGGTGTATAGCTGAAAGCATAAGCTTCTAAACCAGTTGATATATGCTGCTGACTTGTGAATCATATTCTTGTAATTTTTGGTAGATTGTCCAATTTTCCATTCCAGTTGCTCCAAAGGTATTTTCCTACAGTTTAGAAGTTCAGATACTGCAGTTCAGGATCTTGCTCACCATTTTCAATATGTTGTGTTTTCTGGTTTCATAGCAGACCAAGAATTGCCATGGAGGAAAGTGGAGATGACTACAGCCAAGAACGTCAAGATATGGAAGAGCTTCAACATGATGTTGCTACATCACAACCTAAGCCTAAGAAAAAAGGAAGAGGTCCATCAAAAGGGGTCCAAACAACAACTCCTATCTTCCTTGAATTTGATGAATTTGGTTTACCTATGGGGAAATGGGAATCTGAATATGGAAAAAAAATTGGGACTTGTTCCAAAAAAGTTGACATCAATGTTAAGGAATATTCAAAGATGGATAAGCTACGGACGTATCAGAACTTCTTAGAGGAGCATGGGCTAATGTTTCATTGTTTCATGTCTACATTTTGTAAGCTTTTTACACTCTAATCTGTATATGTAATTGAAAGATATTTACATATCCCATCCTATATGTAAGTAAGCAAATTTTGTTTTGTAGGTACCTAATTCATATGCACATTTCCATTTTAAATATAACCGAGATCACATTCCTTTGTCCTCAAAGGATTTCGGTAGCTGAAATCGAGCACGATTATCTCCAAGTTCATGGGTATGTTAAAAAGGCTTTTATGTCCGAACTTGAAAAGGAGGATAAGCATTGCAAATTCATGTTAGCACCATATCTTCAAAGGTATGTCCCATGACACATGCTTAATTATTTACTAATGTATATGTTTGTGAATTACAAGTTAATTAAAGTCTTATGGAATGTCATTTTTTGTATAGTAATCATTGGGTGCTTTTGGTAATAAACTTACAATTGGGCCTTGTGTTTGAGTTTGATCCTGCCACTTGTCCTAAAAAGACCCCAAGAAAATTGAGATTGGCTGATATACTAATGAAGTAAGTGGAGAAAGAACATCATTTTCACATAATAAATTCAAAGGTTATGAAATATGTTTCTGTTTCTATTTTTTTATTATAATTCTTTTTACAGGGCATACAAGGTGTATTTGTCTAAATTGAAGGGTAATGTTTTGAAAAGTAAAAGGAAAAATTTGCTATTTAAGCAAATGGAGGTACATAAGTGATATCATTTACGCTACACAAATTTATGTATGCATGCATTAGTATTTTAATGGGTATGTAATTGTGGGCTTTGATGTTAAATTGTAGTGTGCTCAACAAAAAGGAGGTACTGAGTGTGGATATTACGTCATGAGATACATGTATGAGGTTGTTGTTGGTCACAGTGAGTGCGAGGGTAATTTAGAGGAGGTAAGTGACGTGTGATTGTTTCTTTGAAATAATTAAAATTATCCTTTTGAATGTATTTGAATATATAATATTTGTTCGTATTTTCCCAGGTATATTCTGCAAGGAAAATGGCTTATGATGAAAGGGAATTGAATGAAACTCGAGAACAGTGGGCAAATTTCTTCAGAAGAAAATATTTGTTGAATGAACCAATTTAGTTATATTTTCATCGAACACATAACTAATTTGATTGCATATGTAGTTATGGAAGAGCTTGTTTGTTGATTAGTGAATGGTTTTATTGATGTGTGACATTTAATTTGTTATGTGGAATGATGACTTCATTTATTGGTGAATGCTAATGATGTTAGAGTAGTGTTGTGAGTACAGGTTTATGTACAGGTTTTTATTAATCAATATCAACTTGAAGTTGGGATGTTCGTAAAACAGGGGAGATGCTGTCCGTTTTCCTGTAGGAAAATGAGATTGTATATGTGTCTATGTACTTAAACCTGCAGGAACCATCTCCGTATATAAATTATTTGGATGAAAAAAAACAATTATTCGTAACCAACAAATTAATCGTTAGTTACAAATAGCATGAAGTATTATCATAAATTGAAGGTATAATTGTAACTGACAATAAATATGGTAGTTACGATCAAACCTATAATCGTAACGGACAATTAACATGATAGTTACAAATAATAGCTTAATTGTAACTAACATTTAAACAGTTAGTTACAATCAACTGTCAAATTAGGCTATAAAATGGGGTATTTGTAACTAACATTTAGAAAGTCCGTTACAATTGCCTAACTTTTTGTAACTAACAGTGTTGGTTACAAAAGCCTAATAGTATTTGTAACTCCTGTATACGTAACGGACAAAGTTGGTTACGAAAAGCTCATTTTTGTCAGTTACAAATGTCGTTTTTTCTACTAGTG


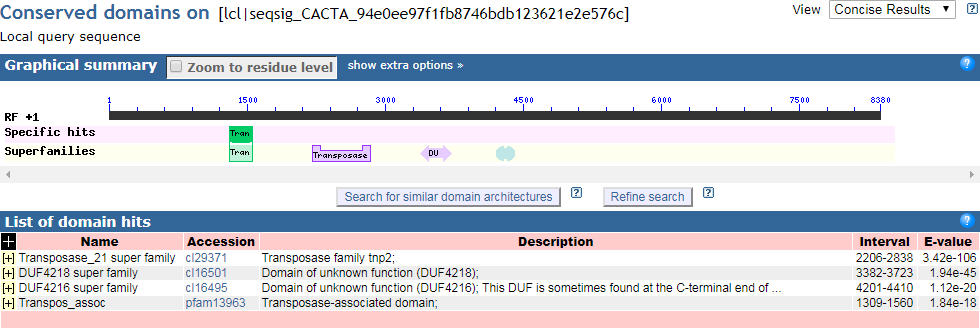


ORF:

MNREWMYDAIRTSPAYLEGVNEFCKIAVQHQWSVVVEQRLTRARPIFCPCCHCKNVKRWDDIKKIEEHLIIHGFMSDYTVWYWHGEKLTPMDSSSSINNDIELDRESGGSDNNDGFDHENNVDEDNINDMMEGLEDRVNENSRTIEEVSKAAETPLYPGCTKYSKLSGVLKLFNLKAKSGWTDTSFTLLLETLSDMFPEGNDIPKSTYYAKKLMCPMGLEYTKIHACPNDCVLFRKENENLDACPQCGESRYKREGLNLDRKKWPPAKVVWYLPIIPRFKRLFSIKQDAKNLVWHDNERKKDGLIRHPADSLQWKHIDETFPEFGNEPRNLRLALSTDGMNPYGTLSSKHSTWPVLLSIYNLPPWLCQKRKYIMLSLLISGPKQPGHDIDVYLEPLIDDLKLLWDEGVVTFDAHTGTDFRLRAMIFCTINDFPAYGNLSGYKNKGQKACPVCEDDMPVTYLKHWGKNVYLHTRRSLRRDHPYRKMKKQFNGFTEKGVCRKPLTGTQVYERIKHINTVYGKLHTPKPKKGVLWKKVSKLWELPYWKHLRVRHCLDVMHIEKNVCDSLIGTLLNIKGKTKDGATVRRDMKEANVRPKLWPEDNESKKKSFLPHACYTLSREEKRIFCECLKGIKVPTGYSSNVSRFVSVTDKKVTGMKCHDCHVMMQVFLPIAIRGLLPKHVRYAVVKLCDFFSDICSKAIDPKRLDEMEADVIETLCKFEMYFPPSFFDIMVHVVIHLPREIKECGPVFMHYMYPYERHMGSLQDKVKNRASPEGSIIQATVAEEAGNFCAVFLARAKEIGVPISRHEGRLQGKGTVGRTLVRPPIDRFQKAHRYVLQQLSEVHPYIEKHQHELQRQNPRITAYSLMQEHNRRFVEWFQVQVKLQLSQNENVPEMIKWLARGPQPLVYTYESYDINGYSFSTYLKDKKSVQQNSGVVVVASSTEYSSAKDTRPTNATQAYYGIIQEIWELDYVDFTIPLFRCKWADNRRGHKRDVLFGYTLVDSSRYIEGEEPFILAKQAKQIFYIKDNMDPMWRVVVQGKRRIVGVEDVVDEEEYDRLDDTPPLSMGVQPLQDGEDIVDDEDYDRDEGVEVDLVTTPL

Conserved domains:

Transposase associated:

REWMYDAIRTSPAYLEGVNEFckiaVQHqwsvvVEQRLTRARPIFCPCCHCKNVKrWDDIKKIEEHLIIHGFMSDYTVWY

Tnp2:

DGLIRHPADSLQWKHIDETFPEFGNEPRNLRLALSTDGMNPYGTLSSKHSTWPVLLSIYNLPPWLCQKRKYIMLSLLISGPKQPGHDIDVYLEPLIDDLKLLWDEGVVTFDAHTGTDFRLRAMIFCTINDFPAYGNLSGYKNKGQKACPVCEDDMPVTYLKHWGKNVYLHTRRSLRRDHPYRKMKKQFNGFTEKGVCRKPLTGTQVYERIK

Domain of unknown function (DUF4218):

ICSKAIDPKRLDEMEADVIETLCKFEMYFPPSFFDIMVHVVIHLPREIKECGPVFMHYMYPYERHMGSLQDKVKNRASPEGSIIQATVAEEAGNFCAVFLARAKEIGVPISRHE

Domain of unknown function (DUF4216):

WELDYVDFTIPLFRCKWADNRRGHKRDVlFGYTLVDSSRYIEGEEPFILAKQAKQIFYIKDNMDPMWRVV

Self-to-self comparisons of the CACTA putative element from genome of *C. pamiricum* displayed as dot plots (YASS program output).


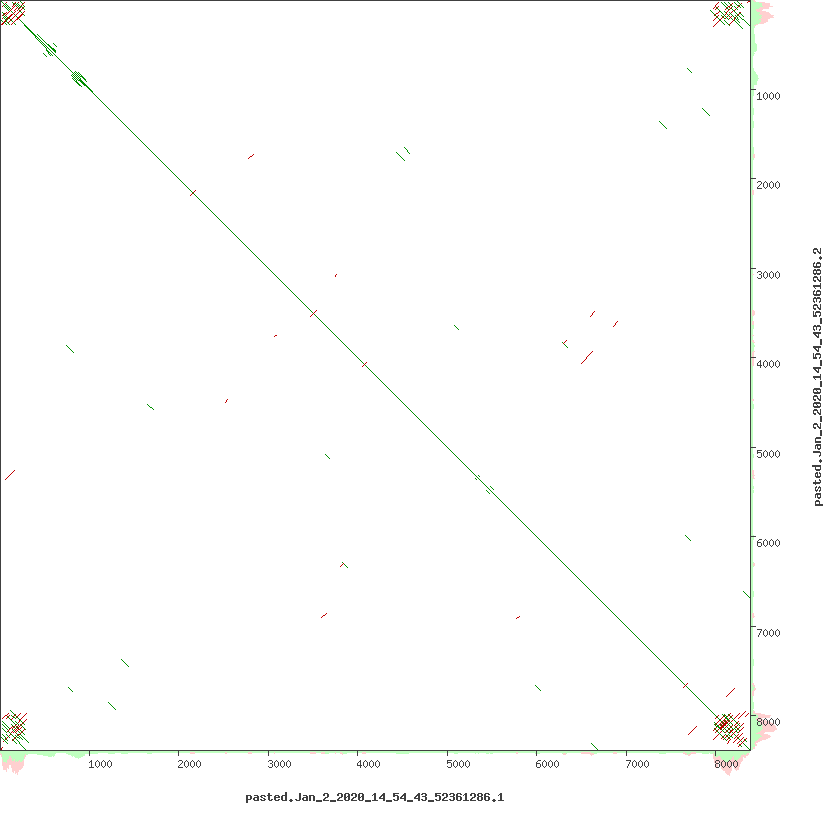


Characteristics of the putative CACTA element *Jozin* from the genome of *C. sosnowskyi*

**Characteristics:**

Length: 8258 bp

TIRs: 28 bp CACTAGTGGAAAAAAGTTCATTTGCAAC (highlighted yellow)

Sub TIRs: 5’ 276 bp, 3’ 258 bp

ORF: position 1620-4928; 3308 bp | 1102 aa

Conserved domains (highlighted red):

Transposase associated: position 1626-1874 bp, 248 bp;

Transposase tnp2: position 2517-3149 bp, 632 bp;

DUF 4218: 3693-4013 bp, 320 bp;

DUF 4216: 4512-4712 bp, 200 bp

Sequence:

CACTAGTGGAAAAAAGTTCATTTGCAACGGGCTATTTGTAACGGGCATGTTGGTGACCGTTACAATTACCTCATTTTTAATGGGAAAAATCAATATCCGTTACAAAAAAGTTGTGAATTGCGAAGTAAAAGTAGTATTTGTAACGGGCAAATAATTGTCCGTTACAAATAACGCCATATTTGTAACGGGCGTTTTACTGCCCGTTACAAATAATAAGTTTTTTGTAACGTGCAGCTTTTGCCCGATACAAATAGTATAGTCAAAAAAAATTTGACCAAGGTATCCGTAACCAACATCACTTGCCCGTTACAACTATGGTTTTAAAATAAACCCTAACATTTCATTTCCCCTTTCTCGCTCTCTCTGCTCAACACCTTTCCCCGCGTTTCTCACTCTAGCTGCCTTCAATTCTCCCTCCATTGAAACTGCACCTTTCGTCGTCGTTCCTCCCTTTCGCCGTCGTTCCTCTCTTTCGCCGTCGGTTGTCCCTTTCGACCTCTCAAGCGTCGTCGATCGTCCTCCCTCGCAAGCTCCTGTGCACCGCCCCTTTCTCCCTCGCAAGCTCCCTCGTTCCTCCGTCCCTTTCTCCCTTGCAACTCCGGTGGAAGTTTCTCGCAGGTTTGGGGTTATTTTCGGCGGCCAAGTGAATAAACGTTCTTGTTTAATTTTTAATTTTAATTGTTGTTCTTTAATTTTCAGTTTTAATTAATTTTAAATAATTCCTTTTTTTTTTGCAGCCGTTGTTTCTTCTACACTAGAAATAGTGCCTCTGCTAATGAGAATTGAAGAAGGGTTGCAAGGCTGAAAGATTTCGCGTTCCCGTTCAGAGCAAGAGAAGCGTAGTTCTAGGGCTGAGAGGTTCTTTCTTTTGATTTTTACTTGTTTCTGATTTTTGTTCAAGTCATGAAATTTGCATTTAATTATTTATTTTTCTGCCATTTTTATAACATTATGATTGATTATTCAGCCATTTTTAATTTTCTGCCATTTTCTTTATTTATATTTATACTGCTCTGATTTTATGCTCTGTTATAATTTCCTGTTTCATTGAAATTATTACTCTATTTTTATTGTTTTTTTTATTTTATGGAAATTTGTTGTATTTTGCATTTCTGTTTAGTTTAATTCAATTTAACTGTTGTTTTGATTTTGCTTGAACATATCTCTAATTTGGTGATTGATCTTGAATGTGTTAATATTTATTATGCATTATATCTTCATATGCCTAGATACCCGTTTAAGATTTACTCATTAGGAGTAAATCTTGAATAGTCCCCGTTTGGGACTGTTCTTGGAATTGTATTCTCACTTAGGATGTAAATGGTTGCTTGTTTTTCGGTAATAAAGGTAATTTCGTGTTAGTATTCCCTTATGTGTTCTCTGGGTACATATGTAATTAAGAAATTAATTACATCGTGTACTTGGAGAACCATAGGGAAATGCTGCCAAAATTTCCTTAGAACCGAAAAGTGGGCAACCATTTGCTAGTGGGAATACACTTCCAAGGATGGGTTAGGTTGGAGCGATAAGGACCTTTGGAAGCATGCATTTGGAATGTGTTGATATGTGATGTTGTTGAATATAATTTGTTTCTTGGGTTGTAATCATACATTGTAGAAATGGATCGTCGTTGGATGTATGGTTCTCGAACCACACCAGAATATTTAGCTGGGGTCAACGAGTTCTGTAGAATCGCAGTGCAACACCAATGGAGTACTCTCGTTGAACAAAGGCTAACCAAAGCAAGACCTATTTTCTGCCCTTGTCGTGATTGTAAGAATGTGAAGAGGTGGGAAGACATTAAGAAAATAGAGGAGCATTTGATTATTCGTGGGTTTATGCCAGACTACAACATTTGGTATTGGCATGGTGAAAAATTAACTCCCATGGATAATTCATCTTCTATTAACGGCAATGAAGCTGATGAGAGAGAGCCGAATGGTTTTGATGATAATGAAGTGGGTGATAATGATGACATGGAGGAAGACAAGATAGATGAGATGATGGACGGGTTAGGTGATCATGTTAATGAAGATTCTCATATGTATGAGAATGTGTCGAAAGCAGCTGAGACACCGTTGTATCCTGGTTGTTCTAAGTATTCCAAGCTTTCTGGGATGTTAACATTATTCAACTTGAAAGCGAAGAGTGGGTGGACCGACACTAGTTTCACTTTGTTGTTGGAAACCCTCTCTGATATGTTTCCAGAAGGAAATGACATTCCTAAGTCGACTTACTATGCGAAGAAGTTGATGTGTCCATTGGGTTTAGAGTACACCAAGATCCATGCATGTCCTAATGATTGTGTGCTATATCGAAAGGAGAATGAAAACTTGGATGCTTGCCCAAAGTGTGGTGTGTCACGATACAAGCGAGAGGGCTTAAATAAGGATCGCTTGAAGTGGCCGCCAGCTAAGGTAGTATGGTATCTTCCCATAATACCAAGGTTGAAGCGCCTGTTCTCAATTAAGGAGGAAGCGAAAAAATTGGTGTGGCATGAAAATGAGAGGAAGAAAGATGGGTTGATTAGACACCCGGCTGATTCGTTGCAATGGAAAAACATTGACAAGACATTTCCAGAATTCGGGAAGGAGCCGAGGAACATAAGACTGGCATTAAGTACGGATGGGATGAATCCATTTAGCACTCTTAGTAGTCAACATAGTACATGGCCTGTCCTTCTGTCAATATACAACCTACCTCCTTGGTTGTGCATGAAGCGCAAGTACATCATGTTATCACTTCTAATCCCAGGTCCTAAACAACCTGGCCATGAGATAGATGTTTATTTGGAGCCCCTTATTGACGATTTGAAACTGTTGTGGGATGAAGGTGTTGTGACATTTGATGCTCTTTCAGGTACAAATTTTAGACTAAGAGCCATGGTGTTTTGTACCATAAATGATTTTCCTGCATACGGAAATTTGTCAGGGTACAAAGTCAAAGGAGAAAAACCATGCCCTATTTGTGAGGATGACATGCAAGTTACACGCTTGAAGCATTGTAACAAGAATGTGTATATGCATACCAGACGATCACTTCGTCGAAATCACCCATACCGGAAGATGAGACAACAATTCAACGGGCATGCTGAGAGAGGAGTGAATCGTGAGCCATTGACAGGAATGGAAGTGTATGAGAGAATTAAAAATGTTAAGACAATCTTCGGCAAGAAGCACAGAGAAAAATCGAAGAAGGGTGAGTTGTGGAAAAAAGTGTCTAAGTTGTGGGATCTTCCTTATTGGAAGCACTTGGCGGTTCGACATTGTCTTGATGTTATGCATATTGAAAAAAATGTTTGTGAGGCACTAATTGGGACTTTGTTGAATATCAAAGGGAAGACAAAGGATGGTGACAATGTGCGTAGAGACATGAAGCGTGCCAAAATTCGACCTAAGTTATGGCCTGAGGATAATCATGCAACGAAAAAAGCATTTTTGCCCCATGCGTGTTATACATTGTCTAGAGAAGAAAAAAGGATCTTTTGTGAGTGTTTGAAAGGAATCAAGGTGCCGACAGGGTATTCGTCGAACATAGGGCGTTTTGTTTCTACTACTGATAAAAAAGTAGTTGGTATGAAGTCCCATGATTGTCATGTGATGATGCAAGTTTTCCTGCCAATTGCACTTCGAGGGCTTTTGCCAAAACATGTTAGGCATGCTGTTGTGAAGATTTGCTTGTTTTTCAATGCCATTTGCAGTAAGGTGATTGATCCAAAAACACTCGACGATATGGAATATGATATTGTTGAAACATTATGCAAATTCGAGATGTATTTTCCTCCCTCTTTTTTTGACATAATGGTTCACCTTGTTATGCACTTGCCTCGTGAGATAAAGGAGTGTGGTCCTGTATTTTTGCGTTGGATGTACCCTTTTGAGAGGCAAATGGCTACTTTGGGAGATAAGGCCAAAAACAGAGCAAATCCTGAAGGGAGCATCATTCAAGGAACCATGGCAGAGGAAGCAGGGAACTTTTGTGCAGTGTTTTTAGCTAAGGCTAAAGAAATTGGGATTCCGACTTCTTGTCACGAGGGAAGACTTCAAGGGAAGGGGATAATAGGTCGGAAATTGGTTAAACCTCCACTTGATCGATTTAAGAAAGCTCACCGATTTGTGCTACAAAACCTATCTGTAGTTCATCCATACATTGAAAAACATCTCCATGAGTTGAAAATTCACAATCCCAGGATCAGTTCATATGCTTTGATGCAGGAGCATAATCGCCACTTTGTTGAGTGGTTTGAAGGCCAAGTGAAGCTTGAATTGAGAAGAAATGAGAACGTTTCAGAAACAATCAAGTGGCTTTCACGAGGTCCACAACCACTTGTTTATACTTATGAGGGTTACGATATCAATGGATTTACTTTCGCCACTTGTCGTCAGGATGAAAAAAGTGTGCAACAAAATAGTGGTGTTGTCGTGGTTGCATCTTCTACTGAATACTCAAGTGCTAGGGATACAAGGCCAATTGAAGCTACACAGGCATATTACGGTGTCATTCAAGAAATTTGGGAGTTGGATTATGTTGATTTCACAGTTCCTTTATTTCGTTGCACATGGGCTGATAACCGTCGTGGTCTGAAAACCGATGAGTTATTTGGATTCACCTTGGTTGACTCCAGTCGATACATTGATGGTGAAGAGCCTTTTGTATTAGCGTCACAAGCGAAACAAATATTCTACATCAAGGATAATATAGATCCGCAATGGCGTGTTGTAGTTGAAGGCAAGAGAAAAATTGTGGGTGTGGAAGATGTTGTGGACGAAGAAGAGTATGACCAATTTGATGACATCCCGCCTTTAACAATGGGAGTGCAACCTCTACAAGAAGGGGAAGATATTGCTGATGATGAAGAGTATGATCGTGAGGAAGGGGTAGAGGTTCCTATAACTACAGCCGAGGTTTGTGTTATTGTATTCCTTTAACAATATATGTGTATTTTTTGGCTATAAATATTGAAATTATTAAATTGGTATATTTAAATCATTATTTGCTTTATTGTATAGTGTTTACATTAATATGATTTTATTTATATGACTTTGATTCTTATTAATTTAATTCCTTGTGTAGGTGTAGGCATACAACAATGGAAGAAAACATAAATGATCATGCTCCACAGGAACAATCCGGTGACATGGATGCCTCCACTTCCCAAGGAAAATCATCTACAAGAAAGGGACGAGGCCCATCTAAAGCAGTCCAAACAACTACACCCATGTTCCTCGAATTTGATGAGTTTGACATGCCTACCGGAAAGTGGGAACTTGCTTATGGGAAGCAAATTGGTACTTGTGCTCAAAGAATTGACATTAATGTGAAGGGATATCCGAAGTATGATAAAGTTCAAAAGCAAAACTTATGGGAGGAGACTAAGGTAATTGGAGTATAAATATGCTTAGTGCTCAAAATTAAATGATACTTATAATTAGTTCTGAATATTAACATCCTCTAATTTTGTAGCGGAAGTTCCACATTGATGATCCCAAGGGTGTTAAAGAGAAGAAGTTCCACGAAGCTGTGGGTGCTAGATTTAGGAAGCACAAGTCTTGGTTAATTTCACGCTTCATTACTAAGGAAAGTGCACCTCCGCCTGACTCACCTAATGCAAACATTAAGCCCTGGGAGCTTTATGAAGGTTACATCACACAAGAGCAATGGAAAGACTTTGAGACATATTGCAATACAGATGAATTGTTGAGACATTTGGCGAGGGGTGTGAGCAATGGATGATAGTTGTGGCATTAAATCAAGGCAATCTGCCATCAAAGCTACAAAATTTCAACCACTTAAAGATAAAATTGTTGAGACTTTTGGCGAGGGGTGTGAGTTGTTGTATTGGTTGATGAGTTCTGATGATGAAAAGTATGATACAACATCGGTACTTTTAAAGGCCTCAATGTTTAATTTTGACAGCGACAAACAGATTTGGGTAACCTCTACCGATGTACTTGAATTTTTAAGAGGAGCGTGGGCTAATGTTTCATTGATCCATGTTTATATCATGTAGGTTACCTAATTATCCTTTAATTATGTTGTTACATTTACTTTAGTAGTATCTGAATTTCTAAGAGGAGCATGAGCTGATTATACTTTGTTCTATGTCTTTATCATGTAGGTACTTAGTTGAAAACGTCATCGACTTATTCAACAAAAATGAGATCACATTCTTTTGTCCTCAAAGTATTTCTGAAGCTGCAATTGACAATGATTACCTTGAAGTTTATTCATATGTTAAGAAAACATTTTTGCATGAAATTAGAAAGACGGGTAACCATTGCAAATTCATAGTGGCCCCATACGTACAAAGGTTCGAACTATGTTTATTATACGTGCACTAGTTTAACTATATAATTACTTGCCTATTTATGTGACTGATATGTTATTCACCAATTTCTTGATTCTCTAACTTCACTTTACCTTTGAAATGATGTAGTGGTCATTGGGTTGTGGTAGATCTACAATTAGGTTTTGCGTACGAGTTTGACTCTATCATGCAACCTAAAGAAAAACCACGACAAATGAGAATTGCTGAAATCATGATGACGTGAGCCACAAACTTGAAATTGTGCCCAAGTTTCTATTTTCTTATTATAAGTGGTGTTGTAGATTATTTTGTTCTAAGTGTTGGCATATCATTTTTGTAGGGCTTATAAGGTATATCGGGCGAATCTCACAGGGAATGAATTGAAATCACAAAGGCAAAAATTGAAATTCATACAAATGGAGGTAAATACACTACTCACCCCATATTGTTATACATTGTTTACTTTGAATACAAAATGTTTAACTATTTGTTATATATGCTAGTGTGCTCAACAACAAGGAGGCGTTGAGTGTGGCTACTACATTATGAGATATATGTATGAGATTGTTACTTCTCACTTGGAGTGCAAAGGTGTCTTAGAGGAGGTTAGTATCTTTTTAATTGAAATGGTTTTCAAGTTGATGTATTGACATGTATGCGATTGTTTACGTTTTGAACCAAAACATGAAAGATGGGAAAGATAGAAGTCCCTTAGGCAATTTTGAACCCTTTTTGGCTCTAAATGCTCCCAAAGGTGAGTAAATGGTCATATGTCATTGATTTTAATTCATAGAAACCAAAATATAAGTTAATTAAGGATGTTATGAGTTTTATTTCTATAATTTGGATCAAAACATGAAAGATGGGAATGATAGAAGTCCCTTAGGCAATTTTGAACCCTTTTTGGCTATAAATGCTCCCAAAAGTGAGTAAATGGTCATATTTTATTGATTTCAATTCATAGAAACCAAAATGCAAGTTAATTAAGGATGTTATGACTTTTATTTCTTTTATTTGGATTAAAACTTGAAAGATGGGAAATATAGAAGTCCCTTAGAACCATTTTGAACCTTTTTTGATTTAAAATGCTCCTAAACTTAAGAAAACATTATATTTAATTGATTTCAACTCATAGAAACAAAAATATAAGTATGTTAAGGATGTTACGAGTGGTAATTCTTTAATTTTGATTACAACATGAAGAATGGGAAAGATAGAAATCCCTTACGCTAAATTTGAACCATTTTTGGCTCAAAATGCTCCAAAAACTTAGTAAATTAATGGTAATTGTCGATTTATTCCAATGATTGTTTTTATTTTGTAACCTCATTTATCCCTTTTTTTTGAAGGATTTTTGCCCAAGAAAAGCCCCTTATGATGCTAGCGAGTTGAATGTGGTTCGAGAACAATGGGCGCAATACTTTAGATCTAAATATTTATTGAATGCATAACTATTAGGTAGAATAATGTAATATTATTAGTGTTGTATGTTTGATAAACTTATACAAGTTTTATTTGTAATGTATTATTACTTATATTTTTATATAATGTCCTATTAATTTCTAGTAATGTTTTGTTCGTTATAATAGTTTATGTACAGGGTTGCAGAGGCACAGGTTTATAATAACAGGTTCTAATTAATATGTATTAATTATAAATATAAATAAAAGCTGCCAAAATTTATTCGGAAAAAAAAAATAATTTACAAACTATTTGTAACGGGCGAAAGAAATGTTGGATACAAATAACCACTTATTTGTAACGGGCATTTATGTTGCCCGTTACAAATAAGTACTTATTTAAATACAAATAAGTCTTTATTCGTAACAGGCGGCTGGAATGCCCGTTACAAATAACTTTCCCTATCCATAACGGCCGCAAGCGTAATGCCCGCTGCCCGTTACAGATAGGCAGTTTTTGCCCGTTACAGATGAGCTTTTTTCCACTAGTG


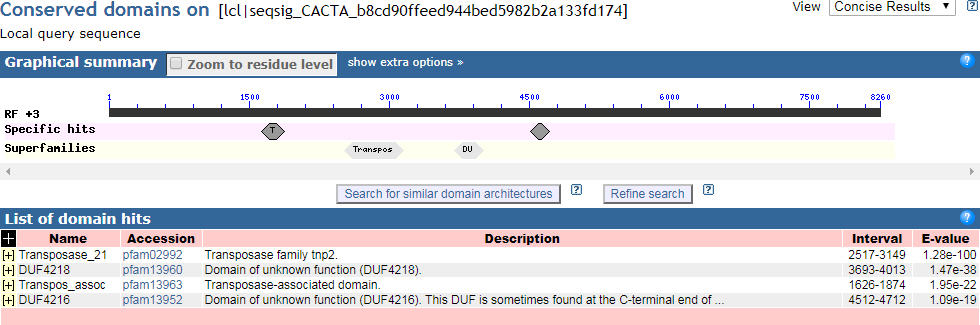


ORF:

MDRRWMYGSRTTPEYLAGVNEFCRIAVQHQWSTLVEQRLTKARPIFCPCRDCKNVKRWEDIKKIEEHLIIRGFMPDYNIWYWHGEKLTPMDNSSSINGNEADEREPNGFDDNEVGDNDDMEEDKIDEMMDGLGDHVNEDSHMYENVSKAAETPLYPGCSKYSKLSGMLTLFNLKAKSGWTDTSFTLLLETLSDMFPEGNDIPKSTYYAKKLMCPLGLEYTKIHACPNDCVLYRKENENLDACPKCGVSRYKREGLNKDRLKWPPAKVVWYLPIIPRLKRLFSIKEEAKKLVWHENERKKDGLIRHPADSLQWKNIDKTFPEFGKEPRNIRLALSTDGMNPFSTLSSQHSTWPVLLSIYNLPPWLCMKRKYIMLSLLIPGPKQPGHEIDVYLEPLIDDLKLLWDEGVVTFDALSGTNFRLRAMVFCTINDFPAYGNLSGYKVKGEKPCPICEDDMQVTRLKHCNKNVYMHTRRSLRRNHPYRKMRQQFNGHAERGVNREPLTGMEVYERIKNVKTIFGKKHREKSKKGELWKKVSKLWDLPYWKHLAVRHCLDVMHIEKNVCEALIGTLLNIKGKTKDGDNVRRDMKRAKIRPKLWPEDNHATKKAFLPHACYTLSREEKRIFCECLKGIKVPTGYSSNIGRFVSTTDKKVVGMKSHDCHVMMQVFLPIALRGLLPKHVRHAVVKICLFFNAICSKVIDPKTLDDMEYDIVETLCKFEMYFPPSFFDIMVHLVMHLPREIKECGPVFLRWMYPFERQMATLGDKAKNRANPEGSIIQGTMAEEAGNFCAVFLAKAKEIGIPTSCHEGRLQGKGIIGRKLVKPPLDRFKKAHRFVLQNLSVVHPYIEKHLHELKIHNPRISSYALMQEHNRHFVEWFEGQVKLELRRNENVSETIKWLSRGPQPLVYTYEGYDINGFTFATCRQDEKSVQQNSGVVVVASSTEYSSARDTRPIEATQAYYGVIQEIWELDYVDFTVPLFRCTWADNRRGLKTDELFGFTLVDSSRYIDGEEPFVLASQAKQIFYIKDNIDPQWRVVVEGKRKIVGVEDVVDEEEYDQFDDIPPLTMGVQPLQEGEDIADDEEYDREEGVEVPITTAEVCVIVFL

Conserved domains:

Transposase associated:

RRWMYGSRTTPEYLAGVNEFcriaVQHqwstlVEQRLTKARPIFCPCRDCKNvKRWEDIKKIEEHLIIRGFMPDYNIWY

TNP2:

DGLIRHPADSLQWKNIDKTFPEFGKEPRNIRLALSTDGMNPFSTLSSQHSTWPVLLSIYNLPPWLCMKRKYIMLSLLIPGPKQPGHEIDVYLEPLIDDLKLLWDEGVVTFDALSGTNFRLRAMVFCTINDFPAYGNLSGYKVKGEKPCPICEDDMQVTRLKHCNKNVYMHTRRSLRRNHPYRKMRQQFNGHAERGVNREPLTGMEVYERIK

Domain of unknown function (DUF4218):

ICSKVIDPKTLDDMEYDIVETLCKFEMYFPPSFFDIMVHLVMHLPREIKECGPVFLRWMYPFERQMATLGDKAKNRANPELCSKELRLDVLEKLEEEIPETLCKLEKIFPPAFFDVMVHLIVHLPDEAILGGPVHYRWMYPVERYLCRLKGYVRNKARPE

Domain of unknown function (DUF4216):

WELDYVDFTVPLFRCTWADNRRGLKTDElFGFTLVDSSRYIDGEEPFVLASQAKQIFYIKDNIDPQW


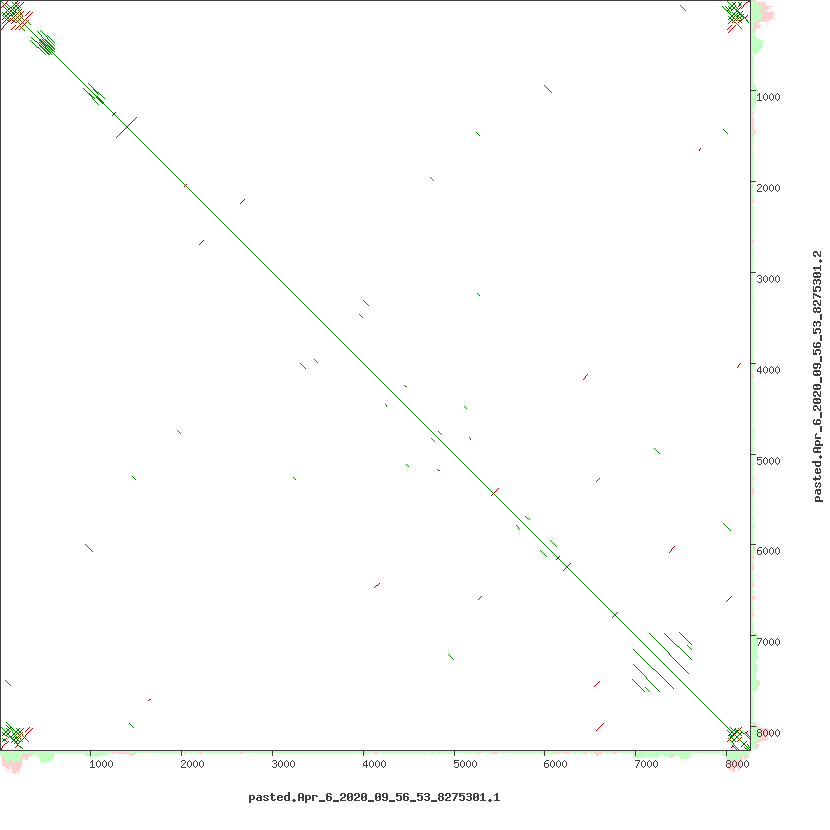

Supplement: Supplementary file 3 — Additional file 3: Characteristics of the putative CACTA element Jozin from the genomes of C. pamiricum and C. sosnowskyi. [file 13100_2020_219_MOESM3_ESM.docx]
