# Supplementary material for: Transposons and satellite DNA: on the origin of the major satellite DNA family in the Chenopodium genome
Source: Mob DNA. 2020 Jun 26;11:20. doi: 10.1186/s13100-020-00219-7 (PMC7320549; doi:10.1186/s13100-020-00219-7)
Supplement: Supplementary file 4 — Additional file 4: Three ON reads from the C. acuminatum genome with association of tnp2B and CficCl-61-40 satDNA family arrays. [file 13100_2020_219_MOESM4_ESM.docx]

**Additional file 4**

Sequences of three ON reads from *C. acuminatum* genome with association of *tnp2*B and CficCl-61-40 satDNA family array presented in Fig 1E.

Presence of *tnp2*B fragment was determined with the conserved GGCTGGGTTACC motif. Presence of CficCl-61-40 satDNA family array was determined with the TTTCATTTGA motif.

Conserved motifs of *tnp2*B highlighted blue. Beginning of parental fragment of *tnp2*B highlighted green. Conservative motifs of CficCl-61-40 satDNA family monomer highlighted yellow (beginning of arrays).

>read 17

CATTGCTACAATTAGTGGATGGCTGTCAAATTCTTGGGTTAAATATTACCCACGCCTACCACGATCGGATCGTAATGCCTTAGTGTCTTCCCAAGTTGATTTTGTACTTCATTCTGGAATTCTTTGAATTTTTCAAAAGGCTTCATGTGCTTCATTAAGTAAATATAACCATATCTGCTTAAATCATCGGTAGAGATTGTAGTATCTAAATCCACACAGCATTTAATTCGTTGGTCCACATACATGCATGAGTCCCGATCAGCAGCTCTTTCACCTATACGCGTAAAGGGTGACTTGAGTGCGTTTTCCCAAGCAAGCATGACTCACGATTACCGAATGATTAAGTAATCAAAGTAAATAGTCCATCCTTATGTAATTTTTGGGCCAGCTTTTGGTTAATATGTCCAGAAGCCTACAATGCCATAGGTAAGTTGGGTTTGAACCATGCATTTTTGCCTTTTTATGATCAATGTTGTGGAGCATGAGTTGTATATTCTAAAACATACAAACCATTAATTAATCTAGCATTAGCATATAACATGTTTTCTTTCGAATAAAACAACGCTTGTCTCTTATTTCAAGCGTAAAACCCGGTGATATCTAAAGATTTGGGCGGAAATTAATGTTTTTGGTAATGCTAGGAGCAAAAATAACAATTATTAAGTATCGAAATTAATCAGAAGGAAGTCTCAAATGATAACTCCCGATAGCAAGTGCGTGACTTTTTGCTCATTTCCTACTCGAAGTGTTACTTCCCTGGATCTAGCTTCCCTACTTCCTTCCAGTACACGCACGTCATTAACTGATGAAGCTTACAGTAGGTATCAAGTGGAAGATGATAAGATTTATTTCTACAGCAAAACATCAAGTAACTCCTTGCATACATTTTTCTTGGTGTATAATGGGGACAGCCTACTGGTGTATGGACTTGCCATCCACCATGATAGAAACTTGCAGCGGTCTATAACCGGGTATACTCGGCATGGTTAAGTAGGATGTTGATCAAATTCTTTGTTGATCCTCATCATTGTGAAAGTCTCGCAGTGTCCCAAAACCAGCATGGGCGAGGAAACGAAGATAATGTCACGTGCATAGTCATCATGTCGGTTTCAGAGACATCCATTTGGTTGATCGTATCAGCTTGATTACATGTTGTATCTTCCTTGCGACTCACCGGGCAAGAGTTTCTTTGCCATGATGTTACTATGAATCCGGTATGTCTCGTACCTGGCTGTCGGCCGAGGAATAGTAAATCGAGGGATCGGTGCGATCTCTCTTACATTTGGATCATTTGATCCTCCGCTCCCAAATGCACCCATGCCGCTCTAGATAGCACGAACATTGATATAAATTGGTCACTTCATACCTTACGAATTTATTATACCCCGACGGTTTGGTAGCAGTCGATTATGGGTTCTTGACCTTTATTGCATCAAAAACAGTTTTGCATGCAATGCGGGATCACGACCCGAGGAGCACACTTGTTCTCAGCGGGAACATACGAGACGAGCACTCGAAGCTTAAAGCTTGTGAAACCGCTTCTCGGCTTCGCCCACTCCCATTGCTGTAATTTCTGAGATTAGCCTTAGATTGAGTTTCATTAGGAGTTCTGCGTTGTCATATAAGTTATTCATCTGAAAACAAAACAAAACGAGCGAAAATTCAATAATTAGCATTTTAATAAATTTAAAGTACCATAATTTTAGTTATTGGTGACTTTAAAACATTTATTCAATTTTAAATTGATCCTATCGAATAAAATGATTCCAAGATCCTTTATTTGTGTAATAATTAACGGAGTGAATCCCATCAACCATTACATTTTGAGGTGAGTAAACTCTTTGATTACAACTCTTGTAATTCTTGGTTTGAATAATTAACCATCGCATGTTTAATTATTACCCTTAGCTTCAAGGTTCTAAACTATTTAGGTAACCTGTTGAATACTCTTGATTAAAAGTGTAGTTATGCGATTCTACCCCAACTTCCCCAAGATATATTATGATGCTCGGTGAGTCTTTGACCACGCCTAAGCGATCGAGAGTTTTCATCGGGTTTTTAATTGGAAAAGCGCATCAATATTTCTGGGATCTAGATTTATTATGTTGTGATTTTATAGTGAACTCTAGCATCATCAAGCAGCATAAATAAACCTAAACATGCATAATCTACTAATCTGATAAAACATGACATAGTGATATGGCCTTAATCCTAAGAAACTAATAAAGTAAATGCTCCCACTTGAACCTTCATGGATCTCATCCATCTTGCTCCATTAGGCCTTCCCTTCATTGGTTTTGATCAAGCTGCAACACATGACCTTTATTCTATTCTACCACTACTTAAAAATAAAATAAAATAAATTACCAAAAATATAAACAGTACATGAACCGTATTTAGATTACAAAAATACTTCACGATGCAGATCGTATTTTAGAATCTATGGAGCCATACTAGTGGTTATATATATAGCGATAAATGATATTATATATTATAAAGTCTTGAAATATAATGTCGGCTAAGTAAATGAGCTTCTGCTTCTGGCGTAATGTTCATAATAAAGGTGCATGGCTATAAATATCTAAATATTTTTAATTAATTACCGTAATTAGAAAATATACATATATTAATCACTGACCAATTATAAAATATCATATATTTTAAAATTTAGGTATGAGATAGAATCAAATCTTATTTGAAATTATGGAATATAAATCATATTTATTTTCCCATGATTATCTAACAATTTACTGCCGGTGAAAAGAATTTCTATCCTAATTGCCCACCCGATCACATATTTTAAGTCGATTATTATCCATAAATAATTATGAGATAATTTAATCAATTATTATCACTTATAGAATCTTTTGCTCCATAATTATCCAAACTTAACAAAATACTTGACTTTACTTTGTTAATTATCTTTTCCAAAATTTAAACTTATCTTTTAAAATTTTGAGCGATTTTGCAAAAATAAAACCAAATAAAAAAAAATTTTTTTTTTAAAGAAAAACGACCAGTGGTGTGGCCAGGCGGAAGCCGGCACGGTGTTTCTGCGCCCACTGCGATAGCGAGGAGCAGAACAGCAGCAGCTACTTGCCATTCTTTGCCACATTTGCTCCTTCTTCTTGGCTCCCGGCCCCGCCCTGCTCTGGACCACCGGCGGCGGCACCAAGTGGCGCGGCGCCGCTTGGCCATGATGGCTAGCTGTACATTTTTCTTTCTCTTTTCCTTTTTTTTTTTTTTTTATATATTCCAATTAATCAAAACATATTTATTTAAAATTAATTAAAACTTTATGTGGTCAATTAATTCCAAAATTAAATCATATTGATTTAGAAATTAGGGTTCTTGATGAGAAATTCAATCCCCGATTTAATTCCCACAACATTTAAATTAAATAATGTGATACATATGAAAAATTCATAATTTTAACATGTAATCAAATAGATTAAATTCCTTAAACATATTATTATCGAGATTAAATCAAAAATCAAACACAAATTTCACAAATTAGGGTTTAATATTTGAAAATTTCGAAATTAGGGTTAGGGTTAGAAATTTCGATTTTATTAGGGCAAATTCGGATCTAAATTATATGGTTAATATGGAGCAAATTAAACACCTTTAATGATTCTATATTGTTAAATCACATTTAATATAACAATTGACTCATAATTAAATATTCTCCTCTAATTGAATTAAAATTGTGGATTATCAAAAATTATTACTCATTATATGTAGATGCAAGAGGCTAAGCGTACCAGCAGGAAGTTATAGGTCTTAAATCACAGATTAACATGAATCATATTCATATAGCACCAAACCAAGGATCACCATGCATAGCATAAGAAGTAAGGTATACCTTTGAGCGTGATTCCCGATGATCGAACAAGAACAATTAGGGGAATAAAGAACCTAATAGTGATTCCTACATTAGGTCCACGGGCACGGAATTAAAGCCTCACTAATGCTAGTTAGTCACCGAGAGAACACTTCACTCGATCTTCTACGGAGATGGAAGAGCAATCAACTAGGCAAGGAGCAGTACAGCACCCGAGGGCCACTAGGGGTGGCGCGCCGTGAGGGAGGTGAGAGAGGTGGCGCGTGTGAGGAAGGGAGAGAGGGTCTTGGATGAGTGAGATTTGCTTACTCACGATTCACTGTAATGTTGAATGTGACTTGCCATCAACTAGGCAAGGCTATTATTTATAGATCACATCTCTAAATAGCCTTAAGTCTAATTCAAGCCCAGCCACATTCTGCCGAACTATACTTAGCCGGCTCCAATGCCTGACTAATTGATTGCTCATGAGGCCCATAAATGGGCCTCTAACCTATGACAATACCCTTGTGTGTGACCAACATAGGATCAACTACAAAGGGCTTTGGGCTATTTGATAACCCCGAAGATCATCTCTCATAGTCAGCAACAGATGATAGCGCCCCCGATAGAATGAACTTATTATCTCCATAATAGTGATCCTTGATATATCTAGCTAATTGCATATCGAAGACATAATTCCTTCATCACCTAGTTTAGCCTTAGTTCTACCCCAGGCCCGCTGCTTGACTTGAAATAGCTTGATTTAGGCGTAGTGCATTTGATCTCAGTCCACTTTGGGTTCGACCTTGACACCACTCACTAAATAGGGGTTTGTTAAGTTACAAATTTTGTTTAATAGGGTGGGGTAGGGTAGCCCCGACGGACTGGATTCTCCTTCCCATCGCTCCCGAGCATTTATAAGTTTATTAGAAAGAGCTTGTAGAAAATGTAGTCACGGCTTATACCATTTTCAGAAAATGCTTGATTTCAGTTCTATAAGACACTTAAAACCTTATAACATCCTTTTTTTTTTTTGTGATTCATTTTGTTATGAAGCTATTAATACATATATTTGACATGACTCGAGCCTTTTCATGTTCATTAGAAAGAGCATGTCGAAAATAGAATACTTAATTTCCCATGATGCTCGGGTGCTTGATTCGATGCTTTACAAGACCATAAAACCTTATAACTTGCTTTTTTGTTTCTATTCTTAGGCAGTTATAAATCATATATTTGACATGATTCAGCATTTATAAGTTCATTATTAGAAGAAATAGTATGTAGAAAATAAGAATGTTTCTTACTATTTTGCCGAATATGCTTGATTTTGAGTACAAGACCTTAAAACCATAACATCCTTTTTGTGATTTATTTTTAGGAAGTTATGAATACATATTTATCATAAATCAAGCCTTTTTCAAGTTCATTAAGAAGAGCATGTAAGAAAATAAGCGGCCAAACTTCTTCATTGCCCGAATTACGCTTGATTTCGGTTCCTCTGGACCTTAAAACCTTATAACTTGCTTTGTCTTAGTGAGTTTATGCATTGAACCCGTATGAACATGGTATTAGATGCCTTTGGACTAGAAGGTGGTTTAGAAATGTGAAGAAGAGCCACACATGCTAGTTACAAGAAGTTCTTTGACACTGTTGAAGCGCGGAGAGAGCCATTGTATGATGGTACGAGTTATGTATTGTACGCATTTACTTGAGGATGGCAAACATCAAGAGTCAGTATAATATTCACGCATAAAGCTATAGATGAATTATTGCTTCATAATGAAAGATAGTTTTACAAGACTAAGACATTGCTTAAGGGCAGGAACTTCCCCACGCACAGATTAAGTATGTCGAATGGTTATGCTATTTTGGAAGGAACAGATCTCGAAAGAATATACATATTGCAAAGGAAGTCGTTATAAGACCTTTAGAGAAAGTGGCAACAATTCTCCTCGTAGTGCTCTTATTTACTTTCACTAATAGGCTAGAGATTACAAAGATTATATACGTAAGATCTTTGCGGAGCCGAATGAGGTGGCATAAAGATGATCCTCGAGTTCATGGCTTATGTCTCATCCAGTGATGAAGGCGTGGAAACACCTAGATGAAGAGTACGCTTCTTTTGCTGCAGACTAAATGTCGACTAGATGCTTTTTGTACCCGACGGATTTTCGTTTGGAAAGAAAGGAAGAGCGATATTCTTGTTGGCCGGTCATTTTAACTACTATAATCTTCCACAGTTGTGCATGAAGATTCCTTTTATGTTCTAAGTTTGATAATTCCAGGTCAGAAGAATCCTAAAGAGAATCTCGATGTGTACTTGCAACCTGCTTATTAAGAGTTAAGCGATTATGGGAGGCTGGGTTACCGACTTGACATCTACATAAAAGCAAAATTTTCATTTATTTCGATTAGCTTTGTATAATGTGTTTGACTTTCATTTTGACTCCAATTAGCTTTGTGAATTACATTTAACTTTCATTTAATTGACAAGCTTTGTTAGATAATGTTTGACTTTTATTTGACTCAGTACCTTTGTTGAATGCATTTTACTCATTTGATTAGCTTCGTTTGAATGCGTTTGAATTTCATTTTATTTCAATTAAGCTTAGTTGAATGCATTTTGACTTTCATTTGATTAAAAAAGATTTGTATGAATATGTTTTACTTTCATTAGACTCAATTATCTTTCTTGAATGCATTTGTCTTTCATTTGATTAAAAAACCAATGTTTGAATGTGTTTGCCTTTCATTTGACTCAATTAGCTTTGTAGTGCATTTTACTTTCATTTGATACAAAAGCTTTCTTTCAATGTGTTTGACTTTCATTTTACTCGATTAGCTTTGTTGAATGCATTTGACTTTAGTTTGATTAGCATTGTTTGAATGTTTTTGACTTGCATTTGACTTCAATTAGCTTTGTAGACTGCATTTGACATTCGTTTGACTCGAATTAACTTTGTATGAATGTGTTTGACATTTGTTTGACTCAATCGAACTGTTAATTCGTTTGGCTTTACGTTTAATTGAAACTTTGTTTGAATGTATTTCACTTTCATTTGATTAAGAAAAAGCTTTGTTTGAATGTGTTTGACTTTCATTTGACTCAATTACCTTTGTTGAATCGTTTGACTTTCATTTGATTAGTATTGTTTGAATGTGTTTGACTTTCAATTGACTCAATTAAGCTTTGTTGAATCATTAAAACTTTCGTTTGATATAAAAGCAGCTTTTGTTTGAATGTGTTTAATTTACGTTTTGACTGGAATTATTTTGTTGAGCAGCATTTGAATTTCGTTTGTTTCAGCTTTGTATGTGTGTGTTTTGACTTTAATTTGACATAATTATCTTTATTGAATGCATTTGACTTTTATTTGATTAGCGCTTTGTTTGAAGGTACTTGACTTTCATTTGAGTGAAAATCATTGTTGAATGCATTTGACTTTTATTTGATTAGCTTTGATTGAATGTGTTGGACTTTCATTTGACTCAGTGCTTTGTTAGAATGTGTATGACTTTCATATTAATTAATTATGTTTGTTGAATGCATTTGACTTTCATTTGATTGAAACTTTGTTTGAAGAGTGTTAGCTTTCATTGAGTTATAAAAGCATTGTTGAATGCACTTGACTTTTACGTTTAATATAAGAAGCTTTGTTTGTATGTGTGTTTGACTATCATTTGTGTATTAGCTTTGTTGATGCATTTGACTTTCATTTGATTAGCTTTGTTTGAATGTGTTTGACTTTCATTTGGCTGATTATCCTTTTTGAATTGATTGACTTTCGATTGATTAGCTTTGTATGAATGTGTTTGACTTTTCATTTGACTCAAATTAAGCTTAGTTGAATACGTTTGACTTTCATTTAATTTAAAAACTTTTGTTTGATTGTATTTGACTTTTATTTGGTTCAAAAGCTTTGTTGAATGTGTTTTTATTTTCCATTTTACTCAATTAGCTTTGTTGAATGCATTTAACTTCTTGATTTTAAAACCTTTGTTTAAATGTGTTTGACTTTTTCATTTGGTTAAACAAGCTTTGTTGAATGAGTTTGACTTTATTTCTCAATTAGATTTGTTGAATGCATTTGACTTTCATTTGATTAAAAAAGCATTGTTTGAATGTGTTAGACTTTATTTATTTGAAAACTTTGTTGAATGCATTTGACTTTCATTTTGATTTAAAAAGCTTTATTTGAATGTGTTTGACTTTCATTTTGGTGAAAAGCTTTATTAAATGCATTTGAATTTAATTTGATTAGCTTTGTTTGAATGTGTTTGACCTTCATTTACTCGATTATCTTTGTTGAATGCGTTTGACTTTTATTTGATTAGCTTTGTTTGAATGTGTTTGACATTCATTTGATTAGCTTTGTATGAATGTGTTTGACTTTCATTTAGTCAATTATCTTTGTTGAATGCATTTGACTTTCGTTTGATTAAAAAAGCATTGTTTGAATGTGTTTGACTTTGTTTGACTTAATTAGCTTTGTTGAATGCGTTTGACTTTCATTTGATTAGCTTGTTTTGAATGTTTTGACTTTAATTTATTTCGATTATCTTCGTTGAATGCATTTGACTTTCATGATTTTAAAAGCTTTGTTTTGAATGTGTTTTGACTTTCATTTGACTCGATTACTTTGTTAGTGCGTTCACTTTCATTTAATTAGCTTTGTTTGAATGTGTTTTACTTTTATTTACATTTCAAATTAGCTTTTTTGAATGCATTTTACTTTCATTTGATTAAAAAAGCTTTGTATGAATGTGTTTGACTTTCATTTATTTCAATTATCTTAGTTGAATGCATTTGACTTTCACTTGATTTTTAAAAGCTACATTTGAATGTGTTTGACTTTAAATTTGGTTAGCTTTTGTTGAATGAGTTTGTCTTTCATTTACTCGAATTTAGCTTTGTTGAATGCATTTGACTTTCATTTGATTAAAAGCATTGTTTGAATGTGTTTGACTTTCATTTGACTTAATTAACTATGATGAATCGTTTATCTTTGATTTGATTCAAAAAGCTTTGTTTGAAAGTGTTTGACTTTCATTTTGACTAAATTACCTTTATTGAATGCATTTGACTTTCATTTGATTCCAAAAGCTTTGTATGAATGTGTTTGACTTTCATTTTATTTCGATTATCCACATTGAATACATTTGACTTACATTTGATTTAAAAGCTTTGTTGGAATGTGTTTGACTTTTATTTTGAGTTAAAAATCTTTGTTGAATCGTTGACTTTTATTTGATTCAAAGAGCCTTTGTTTGAATAAGGTTTGACTGATTTGACTTAATTATCTTTGTTGAATGCATTTGACTTTCCATTTGGTTGGCGTTGTTTAATGTGTTTGACTTTCATTTATTTCCAATTAAGAAAGCTTGGTTGAATGCATTTGAGTTTTATTTGATTCAAAAAGCTTTGTATGAATGTGTTTGACTTTTATTTGACGATTACTTTTGTTGAATGCATTTGTCTTCATTTGATTCAATATATCTACTATTTTTGTTGCTTTTGAATACAAGTTGTTTTACTAGTTGTTGCTAGATTTCTCAAATTTTTGTCTTGTTTTCATATGTGAACAAATACATCAACTAATTTTTATTTGTATCGATTTGAAACAAATTGAGTGACAAATTTGGAATTGTTCCTATTAGAAATTAGCGAGTCTTACTAGTTATTTAAATTTTACATTCATTTATTCAAAAATCTTTGTTGAAAGTATTTGACTTTCATTTGACTCAAATAAGCTTTGTTGAATGCATTTGACTTTCATTTGATTGAGCTTTGTTTGAATGTGTTTGACTTTCGGTTAAAATTGAAAAGCCGTTGAATGCATTTGACTTTCATCAGTTAGCTTTGTTTGAATGTGTTTGGCTTCTAGTTTAGTTAAAAAGATATATTTGAATACGTAAGACTTCCATTTCAAATTTAGCTTGGTTTAAATGTCGACTTTCATTTGGCTGATTATCTTTGTTAGATCATTTGACTTTCATTTGATTCAAAAGCTTTGTTTTAATGTGTATGACTTTCATTTGACTCGATTGTCTTTTTAGATAAATTTGACTTTCATTTATTTTGAAAACGTTGTTGAATGTATTTGACTATCATTTGACTCGATTAGCTTTCTTGAATGCATTTAGTTTTATTTGATTAAAAAAGCTTTGTGTATCAATGTATGACTTTATTTGACCCAATTACCTTTGTTGAATGCATTTTGACTTCATTAGATTAGCTTTGTTTGAATGTGTTTGACTTTCATTTGACTCGATTGTCTTTGTTGATGCATTTTGACTTTCGTTTGATTTAGAAAGCGTTGTTGAATGTGTTTGACTATCATTTGACTCGATTAACTGTAGAATGCATTAAGTTTTATTTGATTGGCTTTGTATCGATGTGTTTGACTCATTTGACCCAGTGCCTTTGTTGAATCGTTTGACTTTAATTTTGATTGAGAGCTTTGTTTGAATGTGTTTGACTTTCATTTGAGTCAAAAAGCCGTTGAATGCATTTGACTTTCATTAGATTCAAAAAGCTTTGTTTGAATGTGTTTGACTTTGATTGAGTTAAAAAGATATGTTGGATACATTAGACTTCATTTGATTAGAAAAGCTTGGTTTAAATGTGTCGACTTTCATTTGACTTCAATTATCTTTGTTGAATACATTTGACTTTCATTTGGTTCAAAAGCTTTGTTTGAATGTGTTGGCTTTCATTTGACTCAAATTGTCTTTGTTGAATGCATTAGAAAGCGTTGTTGAATGTGTTTGACTATCATTTGACTCAATTAAGCTTTGTTGAATGCATTTATGTTTTATTTGATTAGCTTTTGTATCCAATGTGTTTGACTTTCATTTGACCCGATTACCTTTGTTGAATGCATTTGACTTTCATTTGATTCAAAAAGCTTTTTGTTTGAATGTGTTTAACTTTGCTTTTGACTCGGTGTCTTTGTTTGAATGCATTTGACTCATTGATTTAGAAAGCGTTGTTGAATGTGTTTGACTATCATTTGACTCAATTAAGCTTTGTTGAATGCATTTAAGTTTTATTTGATTAAAAAAGCTTTGTATCAATGTGTTTGACTTTCATTTGACCAAATTACCTTTGTTAGATGCATTTTGAATTTCATTTGATTCAAAAAATCTTTGTTTGAATGTGTTTGAGCTTTCATTTGACTCGGTGTCTTTGTTGAATGCATTTTGACTTTCATTTGATTTAAAGCGTTGTTGAATGTGTTTGACTTTCATTTGACTCGATTAAGCTTTGTTGAATGCTATTTAAGTTTTATTTGATTCAAAAGCTTTATCCAATGTGTAGCTTCGTTTGACCTAATTACCTTTGTTAGCAGAATGCATTTGACTTTCATTTGATTCAATATATCTCTTTTTGTTGCTATTGAAAACAAGTTGTCTTACTAGTTGTTCTTAAATTTCATCAATTTTAATATCGTATTATATGTCAATAATTCGACAAGCTAATTTTTTGTTTGTAGCGATTTAGAATCAAATTGAGGCACTAAATTTTTAAGGGTTATTCTATTAGAAATTAGCCGTCTCGTTATTTGTTGCTAGATGACCTTGATTTTTATTTGTTGTCATTTAGCAATGCCATTTCCTAAAACGTTTGGCAACAAAATTTCTAGTTGTTCTTTGATAGGGACACTTGTTTTACTATTTCTTGCTAAAATGACATCACATTTAATTTATTTGCCATTAGAAGAAAGTTGTGTCATAAATTATTTGCTAATTTATTAAGAGTCATTGAAGTAATTTACATAATATGTTTTAATAAATAAAAGCGAAAACAAATCATCTTGTTCTATCTGAAAGAAATATTAGAAGGAATTAATAATAATCGTTCAATCACTTTTAACCTTAATAAGCAGAAGAGAGTAAAATAAATATAAATATTATTTTATAAGTTCATTAGAAAGAGCATGTATAAAATGGGAATGCTTCTTACTATTATGTGAATTTGCTTGATTTTGGTTCTCTGAAGACACTTAAAACCTTATAACATGCTATGTTTTATGATTTCTTTTCTTGCGAGAAGTTATAAATACATATGTTTGACATGAATCAAGCCTTTTCGAGTTCATTAGAAGAGCATGTAGAAAATGGGAACGCTTTTTTTCCCATTATGGCCAAATTAAACGATTTACGTTCTAATTGTTGGGGTTTGGTGAGTCAGGCTCACCCCGCGGAAATAAACCCCGAAACCAAGAGCACTTTTCATGATAAATAAAACCATGAACATAATTAAATTAATCACAAGATTAAGTTAAGGTAATTACCTTTGTGGCGTGCTTCGCTCGAATGAGCTCTTACTTGAAAGCTTCAAGTCCCGCTGCGCACGAGGAGACCTCCAGCAGAGATCCACCCGGCACCAACAAAAGCCAGAAGTAGGTCGATGTCTTAGGGTTAGCAGACCGGGCGATAAAACACAGGTAATAATATTGAGTTGAGTTTCACTAATTGTGATGCCTTTTCAACGCTCTTACTCGAGCATGATTCCCGATGCCCTTACATGGGTATTTTTAGGAAATGATGGGGTATCATTGCTCTCCACGTCAATGTAGGATACTTTGATTTTCCATTTATTTTAAAGCTAAAATAAAATTAATAATCAAGCTAATGTCATCGACAAATTAGCTTGACTCGTAGTGGTTAGGTGAGGACCATTTGGAGGAAGGGGGGGACCAAATGTCCCCACTCCACTCTCCATGCTCCCGCCCGACACACTCGGCACGCACATGCACCTCGAGCCACATGGCGCCGCCTTGGGCACCCAGAGGCAGCCAAAGATGGGTACGCCATGTGGTGCGGCATGGCTGTGCCATGACAAGCCAATGACATGCGGGGCACAACTGCTTTACTCTTGGCAACATGCCCCCGCATGCTTGTGCGGGCTGCGCTGCTGGCCTTGTGCCTCCGCCCAGACCTTTGCCCAGGCTGCTTTGGCCGGCGTCTTTGGCGTGCATGCTAGCATGGTGCGTAGCAGCGCGCCCAGCCGGCCCAGGCTCGGCAGTGCGGCGTCTTGCTCCACCATGTGCTAGGCCATACGCCCTTAGCACTCGGTTTGCTAGGGCATTAAAGTCTTGTGTTATTTGGTCTTTGTTGATTTATTATATATTTCTCGTCGTCTTTCACTATTTTTCGGCTTCGACTTAAATGGCTCGGTTTCGACCTTCTAGCTCGAATTTCGGCGTCTAAATAAATATTACTCGAGTTGACTCATTAATTCCTTAGGATTAATTCGTGAGTTAGATTGTTGAATGCTTAGGTGTGACTAGGAAGAGATTCAGGTCGAATTAGGCTGTAACATTAATTATCCATTAATATTGCGATGAGACGGCCTCCACCAGGGCATTAGATCACTTGACCATCTTGAATATATTAGCTTTCTAATAATTACGAACCGTAGCAAGGAATAATACACTTGTTCGAGAAGGACACCATTCCTTTCAATCTCCTTGTCGCCAGGACAGTGCTCGTTGTGTACGACCACTTTCCTGTAAGCAATATTAGCTACCATGAACTATAAGGTAAGATTTATCTTGTCATCCTTATTACGTCTAGAAGTGTTTCCCGGTCCCAGGAGTCAGAAATGTCAATAAGCGGATGATTTCTAAAAATCCATCAGAGCACGGCCATGTATTTTACAGATTACCGCTCTACGAGGCCTTGTATCTAATAAAGACATCTTTATTTTCTTGGGGAACATTCCCGATCTTGTAATGTAGCTCCTCTAGCGAGAGGACCTACTTGGCAGTGTTGCCGTTATAATCCTAATACAGCGGGCGTTTGACAACATCAAAGCGAACTAGTACTCGAAGCAGAGAACTCCCGCTACTCATAGTCAGAGGAAATAGGTTCAGATAGCCATGATAAAGTATACTTATGTAAAAAATTTTAAATTTCTTAAAAAAGTATTCTCATAGGTCGATCCGGCATAGTTTCTAAACCAATACATTACCAAATGATTTATGACAATCATGTCCTCGTAGCACATGAAACGGTGCTATAAATCGGACTACACACTAATCTCAATTAGTGCGTTAATCGTATCGTTTAAATGACCTTGATTAGGGATAATTTTCGATTATAATATTGATGTTCACTTAATAGGTGTTTCATACCAAGGTGGTCTTGATACCCTATTTTGAATATTCTCAGAATCGAATGGACTCATTCTTTAATCCTAAACCACAATTAAATAGCATAATGGAGTAATAAATGTTGCATGAAATTTTATATCGCTCCCGAGCATGAATGGTTTTCCTTAGTGTCTTACATTAACTTTCTCAAAATTAAGCGGAAACATAAACAAGAAATCAAAGCCGATCTAACATCTATTCCCATCAGTGCTGTGTTCACCAGTTTAATTGGGTGGATAAGCTGCATTGTGTGGTTCAGACTTTATAAATTCTGATTCACCTCTAGGACTTTATTCTCAGTCATAATCAATCGCAAATTGCATGCTGTTTAGTATTCTCTAGTTCTAGACTTTGTGATAGCTCCGTTGTTGTCCTGATAGAAATTTGTGGACCACTATAGAGGTGCACGACATCGAGTTACGACAGACTTACGAATCGAACGGCCTCCTTAGGCGCCGGCGGTGCCCAGTGTTCGGCTTCGGTTGGCAGAATGCACGGTTGTACTGCGCTTGCGCTCTTCCCAGCTAAGCGCCCGCAAGTTTAGGCAGAACTTACATATCCAGTTGCGATTTGAAATCGTGCGTAAATGCGGAAGCTTCAACGTGTCGGTATAACATTCCTGACCAAATCCTTTGTGCCACCATACACGAGAATTTATCCTTGTTCCTTTTGGTACTTTAGGACTTTTTGGCCCTTGATCGATCTCGCCTCTCACGAATCGATTAGTAACGGCTAAATGCTTTCAAGCATCGACACATCGTACAAATGCGTATCATGGCATACATGATAGATCGATAAGCGAAGCATATGGGATCCCCGATTCATTCTTTCAGCTCATCGGGTGTAATACGTGAACCTTTAGCTAACTACTTAGGGTTATGCCATGTGACATTGGCGAGTGACCTCTCTTGGCAGCCTCTCACAGTACCCGAATCGAAGCGAAAGTACCTTATCGATATAAGTACTTTGACTGATCGATCATCCTTTTTCGATCTATCCTATGGATCTTGATGCACTAGTATGTCTTGAGCCTCTTACAAGTCTTTCATTGAGAAGCACTTTCCAAGCCAGGGCCTTCTGAGCTACAACAACGGAATCTTGAGCACCCGATGAAGCGATATGTCGTGACATATAAGACTAGGAAGCGATGTTGCCCAGCGACCTTCTTGTAAACCTGAGATTCATCGGATTCTTGATAAAACCAAACTTACGAGATGGCTTCATGTCAGGCCTTGATTCCAACACGCTTGCTTGCTTTAATCCATGATGGACTTCTTAGAAACACATCTTTCCAGCGTGTTTTGGTCGACAAAACCCGAACATGTCATATACGGTTGCTTCCAAATAACCATTAAGGAAGGCGGTTTTAACGTCCATACGCCATATTTCATAGTCGTAATATGCGTGATTGCTAGGAGTATCCGAGCAGTTTAAAGCATTGCAGAAGCTGGTGAAAGGTTTCATCGTAATGTCCGTAGACATGCGTAACCTTTTGCCACCAATCTAGCTGTAAATGAATACATTTCCATCTTTGTCGGTTTTAAGAGCTTGAATCCACTTACATCAGATCGGTATATAACCTTCGGGTAAGTCATTGAGTCCCAGGACTTGATTTTGAAACATGGAATCCATTTCGGATCCTCGCTGGCCTCTTTGCCGTTTAAGCGAGTCGGTGCTTGCCATCGCTTGCCAGTGAGTTCTTGGGTTCATCTCCATCCATAATCGGGATGTCACGATTCTCGGTTAATACTAAACCAAAGGTCCTCTATCAGGTTGAGATCTCGGCCTCGAGACTACGTAGGAGGTAATCGTTGAGCATTTGCCATTGTCGGTTCGACTTTGCACGTTTGACCTTCGTCAGCCAGTCTTTATTTGAGCACTTTGGCAGTATCATTATGATTTTGTTGCTCATCCTCACGAACTTAGTCGAGTTCGATTTTCCTCCAGCTTGTCATTTTGGAAAGATGCTCATACTCCCGAAAAGACATAATACATGGCAACAAACACTTTGTTCTCGATTTGTTGTAAGTAATACCACTTTGGTTTCTTTTGGGTAGCCTGAAAGTAAACCTTATCAGATTTGGGATGAAGTTTGTCGAGAGGTCAATTTCTTGCATTGCCATGCTTGGCAATCCCCATATCTTTAGAAAAGATAACCTAGGAAGTTTCTTCGTCCCGTAATTCATATGGAGTCTTTTACTACGGCTTTTGATGGAGCACGGTTCGATGTTAGTGCCGCGGTTAGGGTCGTGTCCCCCAGGAGAGATAGGAAGTCACTAAGATTACATTGTTGATCGATACCATGTCTAACAAGGTCCGATTTACTCCTTCGGATACTCAGTTCCATTGTAGGTTCACAGGAGGTGTCGGTCCCGAAAGTATACCTTTTTCTTTCGGATGGTCGCCCGAATTCTTGGCTCAAGTATTCGCCACCTCGGAATCCGATCGAAGGGCTTTGATTTTCTTGCCAAGTTGATTTTCTTCAACTCTGAAATTCTTTGAACTTTTGAATGATTCGGATTTGTGCGACATAAAGGTAGACATAGCCATATCTACTATAGTCGTGCGTGAAAGTGATGAAATATAGATGTCCATTTCTTTATTGGTTGAGCTCATTGGTCCACATACATCGGTATGTATAAGACCCGATAAGTCACTTGCCCTTTCTCAGACTTGGAGAATGAACATTTAATCATCTTGCTAATAAACGTATCCTTGCGATTCTCAAGCGATTCTAAATCGAAGAGTCAAAGAACACCTTCTTGTAGAAGTTTCTTGATGCGTTTGATGTTAATGTGGCGAGCGACAATGCCACAAGTAAGTCGATTTGACTCCATAGTCTTGTTTCTTTTGGTATCATTATTCACATTATAAACTTGTTTGTCAATGAAAGCTCATAAAGACCATTTTCGAATGTGCAATACCATATAAAACATTATCGAGATAAAATGAACATTCTTTGTCCTTTACTCGAAATTCAAAACCTTTGGCGTCCAACGCCGAGTGAAATAATGTTTTTGGTAATGGTGAGACATAATAAACATTATCGAGTTCAAAACTGGCAGGTGGGCAAAGACAAGCGAGGAGTTCCTACGGGATACGGCACCAATTCGGCTCCATTCTTGGGCCGGAGTTGTCTGCTCGCCTGCAACCTTTAGCGTTCCTTAGTCCACGAGAGTGGAACAAGGTGAGCACATCCGATTATCTAATACCCATGATGTTAAGCTAGTGAAGTTAAGTCTATAACGAATATATACCACGATTCGAAGCAACGAGTTCCGTTCTTCTTATCCTCCTTGAACTTGAGGCAATCCTATAGCACAATGTCCGTAGCTAACTACGGTAAGAAACATTCGAATCGGTTAGAAAATGTTTACCTTCTTATCCTTGGTAGCTTTAGACTTAGGCGGAAAAGTTTCTTCCACCTTTCCCCTTAAAGCCTTTCTTCTTCTTGCTAGAAAATTTGAAGACGGCTTGCCGCTTTGTCATAGGCATGTCGTACTTTCCACACCGTCAGCAGGCGTTTGCTCGGCTTGCTTCCAGCATTACCATGAAGCTCGGTGAGGTTTTTCTCCACATCTTTGTGCATATTATAGTTCATCTTGAACTGATCATAGCCTTTGTGCAGCGAGTGGAGAATGATGTCAATAACCATCTCGGTTGAATACCGCATCGAAGCCTTCCCATTGTTTTTGAGCAATCCCAGTCATTTTGAGTACATGTGGGAATTTGTGCTCTCGCTTTCTTGAGCTTGCTCTCAGGATCTACTGTGGGTTTCAAACTAACTGATTCGAGACCTGCTCTTGGAACATATTATGATTAAATAATCTCAAAAGCGGAGTCACCAGCTTTTCAAAAAATGCTTTCAGACTCGGGAGACATTGGCCCGGTATAATACATTTGACATCTCGATTGGCACTTTGCCATCGATTTAGACGACTTGAGTCCTCCTCATTAGGAGTCTCGGGCATTTCCTCGTCAAGGACATATTCACTTTTGCTCTTGCATGAAGAACAATTCGCAAGTCATTTGCCAATCAAGAAAAATTTGGCGCTCCAATTTCCTTGTCGAGAATTGAGGCCAGTGTCCGAGTTATTTGAGAACATGATTCTACAATTGAAAGAATTTAAAATGAAAAACCATTAAATTATACTTAAATAAGATTGAAATCATGCACATTTTAATCAAACTTATTTAAAAGCGTCTTATTCACACATTAAAGATTGTCCCCAGTATTGGTGAGAATAAGTGGGTTAAGATCGAAAGAAACGAAAGTCGGTTAAGCATGCTTTGGCTTGTCTTGTACTCTGATCTCTAAGGTAGCCAACGCTTTGCTAATGGCTCAAGCCAGCTCTTGGTTGAATAAATCGTCGCGTCCCATGCTCTCTAAACAATTTATTCACTTTGCCACGACATGTTAGGACTCCATCACTCATATGCGTTGGTATAACCAAAACCACTTGCATACCTAGTATGCACCACGTTCCTTGGAAGTAGTGATGCTCGCTTTGCGTAGCCACCATACCTCGTTAAGGTTGGTAAGTGTGTTTTGGAATGGCAAGGATTTAACTCAATTTTAAGGGACCTAGGGTTTCTATGTTGGTTGATTTAAGTGAACTTAGTAATCATCCCGACATATGAAATCTAACCATACATCTAACATATAATTCATATATATAAACATAAATATATATCGAAAACATAAATATAGAGATGCATTTCTTCTTTGCTGGCAACTAGCATGACCCACTTGAGATCTTCAAAGTCTTCAAAGGTCTTCTTTTGCAATATAATGGCTTCGTCTTCTAAGAACCCCGTCTTAAGACTTGACCAAATAATATAATTGCTAGTGTCTACGATTCGGACTAATCCCAAATATTAAGGTACAAAAATATTTTCCAGCGGTTCGCGGACCGTATTTAGATTTACAAAAATGCATCACGGATCACGATTAGTATTTTCTATCCTGTCGGACCCCCACTAGTTTGCCATTTCATAAAACACAATGAGAGAGATTTACATTCCATTCATCCATTCACTTATCGTGAATGGCCCATGCATTTACAACGATCCCGAACAAGTAGCAATCCCGTAAAACCGCGCGAACTAGTAATTAATGCGGGTGGTTAATTCAATTGCTCACGCCCTAATTAAACTAGTCAAAGTTGTTTTTATCAGGTTAGGTAATGCCACAGATACGGCTCCCACTAGAATTCGCATCAGGCTTAATTAATCGATTAATTAAGTCGGTGAACTGAAATATTTGTCAAATTAATTTTGCAAATGTAAACTCATAAACCAGACCTAGCCTCCAAAATTTAATTAAATAAACAAAATAATTAAAATAAAGCTTGACAAGTCAAGCAAAATTAAGTATTCCATAATGTCTAAGTGCTATGCTTGAACGGAACAGAAATTACCACTTATTTACTTTTTAAGCAATCTTTAAAATTAACCTTAAAATTAGCATCGACTTAATAACTAGGGGTTATTATGCTAATTATTGAATCGTACATACCGTACAACAAGCTGCGTAGCATAACCATCGGCACCCATAACACCAAGCATGCCCATTGGGCTGCTTGTGCTTTCAACATACGCCGCCCAGCCAGCTGACGTGTAGCAAATACGGCGCCGCATTTCAGAGGCTGGCTAACTGCATCATCTTCTTGCTCGTAGGCTGGGCCGGTGCGGGTATTAGCTGGCAGCCAGCACGACAGCCACGCCAATCCTCGCACACACGGCCTCACGCACCAAGGCCACGAGGCACCCAGCAGCCTCGGCAGCCACATGGGTTGCGTGGGCAGCCGGTGGCCGAGGAAAGCTGGCTACTAAAGGGGCTCTTAGGCCTCTTGTTGCGTGCTAATGCTTAGAAAATTATCGAATTTTTGTGTGACATTATAAGTGAAAATTCTAAATTAATTTTAGAATCAATTATTTTGAGGAAAAATGAAAAATAACAAGTCAACTTAAATAATTCATCCAAAGCAATTAACGGAATTTATTAAAGAATGTTCTTTTAATAAAATTGGTGCAACTTGATGTAACAAAAATAATTAGGGCATTTCTCACTATTAATTTAATCACTAAATTTAATCTTGAGATATTGACAAAAATAAAGAGTTGGAAAAATGAACATAAGTTCAGCTAAAGACTCAACTTTAATACTTGTGACTTCATGAAAAATCATATCTTCCAATATGAAGATAAACTAATTTTTATCACAAGTTTTAGAGTAGAAAACCGATTTCTGAAATCGGTCTGAATCAGACGGGTAAATTGTGGATTTTAAGGCATATTGGATGGTTCTGCCAAACAATTCTGGACTCCGGAGGGTGATATTAAGGAACATTAGTAAGCATCTAGGCAATTTTTTTTTTATTTAGAATGAAAAAGGTCAGAATTCAAGTGAAGGCTATAATACACGCTTTTAAAATTTTGAGTTAGGGGCCGTGTGAAAAGTCTCGAACCATGGGATACATCTTAGAAGTCATATAATGGCGTTTGGAAGCATATATGAGCCTTATATGTTGTACAAAATGGTAGAGTGGAGGCTTGATTCTGAGATCGATTGCAAAATCGGAGCGGGTAAATTAGTAGTTTTAAGGCATATTGGATGGTTCTACCCACTCTGATTGGACTCCGGAGGGTGTGAGGACCATGAGTAGCATCTCGGCATTTTATTTTGAGTCAAAATAGAGTGGAAAATTGTCAAAATTGAAGGCTATAATACGATTTCGGATCAATTTTTGACACTTTTTCAAAAAATGATACGAATGTGTTGGATGAGTCATATAAGGAGGATTTGAAACCATAAGACACTTGGAAATAAAGATGTCAATAAAGTACGAGAATGAGTTTTGACTCTATTTTGAAATTAAAGTGATGTATAGTGATGTAATAATGTGATGTGACTTGACTATAATGATGTCGTATGTGTATATTGATGTCGTATGTGTATATTGATGATGTGACTTAGACTACAATGATGTTAGAAAGTATATATAAAGTGATATGACTTGACTATGATGATGTCGTATAAGTATATATGTTATTAAATTAATAAATAATATGACATGAGTTGAAAAAGAATGATGTCATATGGGTATAATTATGCTACATAAGTGATGTAATAATATGGGTAAAATAGTCATGTGATGTGATGTACCCATAAAATAGAGTGATGTGATTTGTGATAGTAATGGAAATAGACACATTATATCATATGATGATTGATTTGGCTTGATAGTGACTTTCTTAAATTTATTCTTATTGGCATGTGTTTGGTTGATATGATGTGTGATGTGATAATGTTGGGGATGATTGATATATTTGAACAAACTATTCATGTTTGTTTGTAATGTAAGTATCATATAAGCAGTCCCCAAAATTAGCACTGCTTATGTATTGAGGTAGGGTTGTATTAAGTGTCAGCGTATTCTGGTCGGTCTAAAACCTCGAAAAAAAACACAATATAGCAAATATAGGATGTAAAAGTGTTTAAAACCCGTCCCACTCTTATTCCCAAGCTTTCCCCGTCTTACGAATTTGTTTTCGACGGTTTTCATATTTTCCCAAATTCACAGCCTTTTTTTTCAGTATTTTCCCTTTTGAGGCCCACAAAATATTTTACGGAAAAATAAGGGTATGTAAAAATGTAGATTAATACCCATTCTTTGGATTCACGAAAAAACGAGGATGTTATTATCACATTTTCTGAAGTTTTGACCCGTTGTTATTATTCTTGTCATTTTGGGTGTTCAGGATTTTATAGTGCTTATATAAAGCTACATAGGGAAGGGTGCTTGACTCAATGTTGAATAGCTATAATATAGTTATTCAACATTGATTCTCTCAGGGCCAGGTAGGTTGTGTGCATTGCAGGGCGCTAGGAAGAATGCACACAAAAAGGCTCTCAGGGCGAGGCGGAAAATATGACGGTGTCATGGCTTCGAGGGCGGGAATGTTGACCGACCGCATGCTTGGAACGATGCGGGCCAGCGACCTCGAATCTATGTGGCCGATATCGTCAGAGTGTTGCCTTGACGGTGTCGCGTTGGCGTTGTACTTTATGTTAGTTTAGGAGTGTTTTCAACTATATTTTTCGTGGCGACTTTTTGTTGTTTGGGGAATCGTAGTTAGGATTCTTTCGTGACTTGATGGTACAAATGCGACAAAAATTCGTTGATGGAGAACACGTGTGATGCAGGGTTCGGCTTTTTTGTTCGCCGGGAGCACAGGGCGAACTAATGGAACAGTCATTGTCCCAATTCGGTCGTCTACGTAGGGTATCGCGACGGTGAGAAAGTGTTCGGGAGGTGCCTTGATGCGGACTATAATACGAAGCGAGGGCGAAAGTATTCTTGATTAGTCGATTCTTGACATTTGTGGCATGAAATCCATCGTATAGCGATCTAGCTGAGTTAACGATTGGGTTGTTGTTTTTCACGTTTACGATTGCTAGTTGAGGATTAGGCCAATTCTTGCGGTCATATTTGTGGTTTGTGGGTTGTACGGCTGGGATTTCTTGATATAGCGTTACGGAACACGTGTGATCGTGATCGGTTTTTGGTGGCGGAGCTCGTGCTTGTGCATCGAGCCGCATGCGATAAACCTCTTGATGTTGCTCAGTCACGGTAAGCCTTGGGCGAGGTATGGTTTTACGTGTTTATTCCTAATGGCCAGGTATTGATGTATGTAAATCTTTGTTGCTTTGACTTGCATCCTTTTGGGTGTCTGGTGGACGCAGCAAGTGGAGTCGAGTCCTCCCGGCCTCGGCCCGCGAAAATCAGCGTTGTTGGTCGGGTGTACGGTCTCCCCAGTTTGTGCCGGGATACGAGAATGTTGAGGGGAATGGCGTTCATTAAGTCGCGTACCTAATTCGGCGTTTTACGCTAAGCACGAGCGACGTCGGGTGTACGAGTTGACTCTTCCCAGATTAAAAGGGAGAGCTTCGTGCGTCTTAGGTGTCGAGCAGTCTTCCACGGTTGATCTGCCGAGTAGTCATATGCTTGTCTCAAGATTAAGCCATGCATGTGTAAGTATGAACTAGTCGGTTTGTGAGCTGCGAATGGCTGTTCATCGATTATAGTTTGTTTGATGAATTTTCTTGCTCGGATAGCAGTAGTAATTCTACCATTGTACGATGCCTTGAGCCCAGGCTACGGAAGGGATGCATTTATTAGATAAAGGTCAACGCGAGGGCTGCGCGCGTTGCTCACGATGATTCGCGATAACTCGGCGGATCGTAGCGGCGCTGCTTGTGCAGTGACGCATCATTCAAATTTAAGCCTATCGACTTTCGATGGTAGGATAGTGACTGTGGTGATGACGGGTGACGGAGGTAGGAGGTTCGATTGGGAGAGGGCACGAGAAACGGCTACCACGTCAAGGAAGGCGGCGGCGCGCAAATTACCCAGATCGACACGGGGAGGTAGTGTGATAAATAGCAATCAGGGCTCTTTGGAGTGCACGGTAATTGGAATGAGTCTGATCTAAATCATGCGAGGATCCATTGGAGGCAAATTACGGTGCCGGCGGCAACGGCCAATTCCGGCTCAATAGCGTATGTTAGGTTATTGCGGGGTTAAAAGCTCGTAGTTGGACCTTGGGGTGAGATGCAGCCAGGTCAGCCTTTTGGTGTGCCAGGCCAGTCTCGCTGCTTTTCGCCCGGCGATGCGCTCACAGCCTTAAGCCGAGCGGGTCGTGCCTCGACCTTTGTTACTGAAGAAATTAGAGTGCTCAAGCTGACTCGCTCATGTATACATTAGCATGGGGGATAACATTATAGGATTAGGTCCTATTGTGTTGGCCTTCGGGATCGGAGTAATGATTAACGGGACGGTCGGGGGCACGTGATATTGTCAGGTAGAAATTCTTGGATTTATGAAGACGAACAAGCTGTGAACATTTGCGGATGTTTTCATTAATCGAAACGAAAGTTGGGGGCTCGAGACGATCGGATACCGTCCTAGTCCTCGGCCGTAAGCGATCTTAGGCCGGGGATCGAGGCGGATGTTACTTTTAGGGCCGCGGCGCCTTATGAGAAATCAAAGTTTTGGGTTCGGGGGAGTATGGTGTGAGAATTTGGCTTAAAGGAGGTGACGGAAGGGCGCCACCGGGAGTGGAGCTGCGGCTTAATTTGACTCCCGAGCACGGAAACTTACCGAGGTCAGGGGCGTAGTAAGTTAATGATTTGAAACTCTTTCTTGATTCTATGGGTGGTGGTGCATGGCAGTTCTTAGTTGGTGGAGCGGTTTATGCAGTTAATTAGTTAGCAGACGAGACCTCGGCCTGGCCCAGCTAGCTATGCGGAGGTATACCTTGCCGGCTAGCTTCTTAGAGGGGCTATGGCCTTTCGGGCCACGGAAGTTTGAGCGATAACAGTCGTGATGCCCTTAGATGTTACGGGCGCACAGCGCGCTGCTTGATGTATTGGCAATTACATAGCCTTGGCCAGACGGAGTCAGGACCAATCTTTTGAAATTTCATCGTGATGGGGATAGATCATTGCAATTGTTGGTCTTGACGAGAATTCCTAGTGCGCGAGTGTCGGCTCGCGTTGACTACGTCCACGCCCTTTGTACACACACGCCGTCGCTCCCTCACAGGTTGAATGGTCCGGTGGGTGTTCGGATCGCGCGGCGTGGGCGGTTCGCCGCCGGCGGCGGCTCGCCCGAGAAGTTCGAACCTTATCATTTAGAGGAAGGAGAGTATGCTGGGTTTCAGTAGGTGAACTGCGGAAGGATCATTGTCGAAACACCCCCAGGCGACGAGAGAACATGTTTATCATGAGCGGGGATGTGGTGAAAGCCGGAATCCCTAATTTGAGATGATTTTCGCCTCGGTGGGGTATCTTCTCGGCCACGATATGGCTTGGCGCGATGCCGAGGGACAGTGGATCTGAGTGTGTGCCGCTTACTGCCGGTGGTTCGCGGATGTAGAAGGTGGCACGAGTCGTATATAACATTCGCGACTCTCGCGGCGGATATCTCGAAGCTGCCGCATCGATGAGAAGACGTGGCGAAATCACGATGCCAGGTGTGAATTGCGGGAATCCGGGTGAACCGCCCGAGGTCTTTGAGCATGATTGCGCAGAGCCTTTAGGTCGGCGCTTTGGGCGTCACGCATGGCGTCTCCCACCACCTTGAGTGGGAGGCGGAGGTGATGGCTCCCATGCCTCACTTGGGTGTGGATGGCTAAATACGGAGCCCAGGTTATGAAGTCTTGTGGTATAGGTGAGATCTGAGCTGCCTAGAAGTCGGCATATTCGCGCACTTTTTAGCTTTTGAGGACTCGCGGGATTAGGTTTGTTTGCCCTTTGGGGCATCAAAACGTTGCGACCCAGTCGGGGCTACCGTGGGTTAAGCATATCAGTAAAGCGGAGGAAAAGAAACTTGCAGTTCCCACAGCCAACGACGACGAGCAGGGAACGGCCGCGGCTTTGCGTCAGGCGGCTTCGTCGTCCGGGTATGGTACGGGCGTCCTCTGCGGCGGACCGGGCCAGTCCTGGAAGGGGCGCGAGGAAGGGTGAGCCGGGTCGTGCCGGGACCTGCAGTCGCGCCCACACGTGTCGCCCAGAAATCGGGTTGTTTGGGAATCACGGCCCAGAAGTGGGCGGTAGAATTCGAGTCGAGGCTAAATGCGAGAGGCCAGGTGGCGAATTAAAATGCGCGAGGAAAAGATGAAAAAGGACTTTGAAAGGGAGTCGAAAGTGCTTGAAATTGTCGGGAGGGAAGCGGATAGAGCCGTGATGTGCCGGTCGGATGTGGAACGGCCCAGCCGTCCATCGTCGGCTTCCGAGGAGGTGCGAGGCCCAGAAGCCGCGGATTGGGGCGGCTAAACCCGATTTCGGGCTGTCGTCCCTCGATGCGTGGTAAGCGGCCTTGGCCGTCGCGAGCGTCTTCGGCCTGCGCGCGCTCCGGGCGTCGGCTGCGGGCCCCAGATTCGGCAGATCTTGAAGCACGGGAGTCGACATGTGTGTGCGAGTCGGCCCGGGCGAGTAAGCCGTCTACGGCGTGAGGAAGCTAATTGGCGGGATCCCTAGCGGGTGCCAGTAGGCCCAGGCCTTGATCTTACGAGGAAGGGGTTCGGTGAGGCGTGCACAGTCGGGACCGAAAGATGGTGAACTATGCTGGCCGGGGCGAAGCCGAGCTGCGGTGGAGGCCGGCGGTATCTTGGCAGTGCAAATCGTTCGTCGGAGGCTTGGGTATAGGGCGAAAGACTAATCGACCGTCTAGTAGGCTGGTTCCTCAGGGTTTCCCTCGAGGATGAGCTGGAGCTCATTCGCGAGTTATCCCGGGTAAAGCCCCGATGATTAGAGGCTGCGGGGCGCGGCGCCTCGACCTATTCTCGAGCTTTAAATAGGTAGGGCGGCGCGGGTTTGCTTTGTTGAGCCGTGCCATGGAATCAGGCTCCAAGTGGGCCATTTTTGGTAAGCAGAACTGACGATGCAGATGAGCCCAGGGGCCAGGGTTCACGGTGCCCAACTGCGCGCTAACCTAAGATCCCAGCGAGGGTGTTGGTCGATTAAGGCCGGCGGGACCAGGTGGTCATGGAGTGAAATCCGCTAAGGAGTGTGTAACGGCTCGCTCTTGAATCGACTAGCCGAAAATGGATGGCGAGCGTGAGCCCCCAGCATACCGGCCAGTCGGGCGGTGCGAGCCGGAGTGAGTGGGAAGAGGGCGCGTGATGTTGCAAAACGTGGCGTGAGCCGAGGCGGGCGGCCGTTGAGTCACGGATCTTGGTGGTAGTAGCAAATATTCAAATGAGAACTTTGAAGGCCGAAGAGGAAAGGTTCCATGTGAAGCTGACACTTGCCGCAGTGGGTTGAGTCGTCTGAAGACCGGGGCTTGTCAGATAAGCGCGTTCGCGCGAGCTTGGGAATCGGGTTAAAATTCTGAGCCAGGGACGTGGCGGTTGGCGGCCAGCGTTAGGAGTCGGAGGCGTCGGCGGGGGCCTCGGGAAGAGTTATCTTTTACGTTACTGGCTTGCCCACCACTGGAATCGACTCGATCGGAGGTAGGGTCCGGCCAATTTGGAAACACTCTTACTTACGTTTTGTGTGGTGTCGGTCTTCGACCCTTAATCAGGAGGGCCAGAGTGCAGGCGCCGGTCGTGCTCATAGCACTTGTCAGTGCTCGAGGTGAGGCCTCTGGTCGATGGAGCAGTAGGCAAGTCGTGAAAATGGATGTCTAACCGGGGAAAAGATTGGCTACGAGTGGGCACGGGGTCTCCGATCGAAACCCGTCGGTGTCGGCGGGCTGCTCGAGGGAGGCTGCCAGTGGCGGGCGGAGTCGCCGCGTGCCCGGCCAGAGGGACGGGCCCAGGAACGATCCACCCGGGGGCCTTTCCGGGCGTCGAGCAGTCGGCTGGGCGAGTGCGAGACCGAGGGGAATCAGTGTTTAATTAAAAACGAAAGCATTGCGATGGGTACTGCGGATGTTGCTTGCGATGTGATTTACGCAGGTGCTGCGAGATGTCAAAGTGAAGAGATTGACAGCGGGGAGTAGACGGCGGGAGTAACTATGACTCTCTTAAGGTAGCGAATGCCTCGTCGTCCTAGTGGTGGCATGAGATGGATTAACGAGATTCCCCTTGTCACATCTACTTATCCGACGAAACCCACGGCCAGGGGAACGGGCTTGGCCCAGAATCAGCGGGAAAGAAGACCTGTTGAAGGCTTGACTCTAGTCCGACTTTGTGAAATGACTTGAGGTGTAGGATAAGTGGGGGCTTCGGCGCGAGTGAATACCCTTACTTTTAACGTTATTTTACTTACTCAGTGAGTCGGGAGGCGGGCGATGCCCTCACTGAACTAAGGCAAACTGCCCAGCCGTCCGGCGAAAGGACATTGTCCGAATTAGGGAGTTTGGGTGGGCGGCACATACGTTCAAAGATAACGAGGTGTCATAAGATGAGCTGGCGAGCAATCTCGTGTGGAGCAGGAAGGGTAGCTCGTTTGATTACGATTTTTCGGTGCAGTACGAGCCGTGAGCGTGGCTTTAACGATCCTTTATTAGACCTTTGGTTAAAGCTAGAGGTGTCGAGAAAAAGTTACCACGAGGGATAAGCGGCTTGTGGCGCCCAGCGTTCATAGCGGCGTTGCTGATCACTTCGATGTCATTTCTTTCCTATCATTGTGACGGAATTCACGAGTGTTGGATTGTTTCACCCACGATAGGGAACGTGGTTTGGGTTTAGGCCAGTAAGGCGTGAGACGGAGTTAGTTTTACCTGCGATGACGATTATCATGATGGTAATTCCAACTGGTACGAGAGGCAGTTGATTCACACGATTGGTCGTCGCGCTTGGTTGAAAAGCCGGTGGCGCCCAGCTGCGTGTCTTTGGATATGGCCCGAGAACGCCTCTAAGTCGAGTCAAGGCCGAGAAGCAGCAGCATGCGCCGCCCGCCCGTTGCGGAATCTACGGGTAGGGTTTTCGGCCCAGAGGCGCGTGTCGTAGGCTAAGCTCGCGCGGTGGATGCGCCGTGAGGCCGTGTAATGCAATTTCTCTGCAGCGGCGGGTTGAATCGCTGCGAGACGACCATCTGGCGGGGTATTGTAAGTGGCGAGTGGCCTTCTTTGCCACGATCGCATTTGGTCAGCCACTATCGTAAGCTTCGATTACCTCACACCGCCTATTCCGCTTAAACACCTATATTTGGAAAACGAGGTTAAGCGATATATCTCGTATAATAAGAGAATATGCTAAGTGCAGGATTAAGACATGTCACAGCCTAAGTGTGGGGATGTTGTACGGTACAGTAGTGTGGCATGTACGAACATGTGCCTGAGGCTGAGAGAGCATACCGGTGTGGGAATGTGAAGGAACATTACGAGATGCATCCAGGGTCGAGATAATGTGGCCCCGACCCATATATGACCATTATCCGCTGCATACGGGCGACGTGAAACATAAGCTATCTGAGAGGTGTGTGCAGCGGTGAAATTGACCATTTTAAGGCATATTGGATGGATTCCGCGCTCGATTGGACTCAGGAGGGTATTAGGGGCAGAGTGGTAAACATCTCGCGATTTTTATTCCGGGCTCAAAGGTCCAAAAATTCCCAGTGAAGGCTATAATACGCTTTTTAGGAATTTTGAGGGTTCGGGGGGCCGGTGGCGAAAAGTCTCGAGACCATGGGATACATCTTAGGGTCATATAATGACGTTTGAAGCATATATGAGCACATATGTTATGCAAAATGGTCGTAGTAGGCTTGAGTTCTGAAATCGGTCTGTCAGGCAGGTATTAAATTAGTAGTTTAAGGCATATTGGATGGTTACCCGCTCCGATTGGACTCACAGGAGATATTAGGAGACCATGGTAAACATCTAGGCAATTTTTATTTAGGAGCGAAAAAAGGTCAAAATTCCAAAATTGAAGGCTATAATACGCTTTTTAGGAATTTTTGGGGATTCCCGCGGGGCCGGTGAAAAGTCTCGAGACACATAGGATACATCTTAGGAGTCATATAATGACGTTTGGAAGCATATGAGCCTTGCTGTGTGTACAAAATGGCGAGTGGAGGCTTCGGTTTAAGAAAATCGGTGCAAAATCGGGCGGGTAGAGTGGCTAGTTTTAAGGCATATTGGATGGTTCTACCCACTCGATTGGACTCCGGAGGGTGTTAGGGACCATGGTAAGCATCTCGGCATTTTTATTTTGAGTCAAAATAAGTGAAGAATTGGCCAAAATTGAAGGCTATAATACGATTTCGGATCAATTTTTGACACTTTTCAAAATAAATACGAATGTGTTGGATGACTCACGGAGTCATATAAGGGAGTTTGAAACCATAAGACACCGATAAAGATGTCCATAAAGTACCAAGAATGAGTTTTGACTCTATTTTGAAATTCATGATGTATAGTGATGTAATAATGTGATGTGACTTGACTATAATGATGTCATATGTGTATATTGATGTCATATGTGTATATTGAAGTGATTAATGACTTGACTACGATGATGTTAGAAAGTATATATAAAGTGATATGACTTGTAGTATGATGATGTCATATAGTATATATGTTGTCGTGTATAATATGACATGAGTTAGAAAAGAATGATGTCATATATGGGTATATTATGCTACATAAGTGATGTAATATGGGTAAAATAGTCATGTGATGTGATGTACCCATAAAATAGAGTGATGTGATTTGGCCAGTAGTAATGGAAATGACACATTATATCATATGATGATTGATTTGTATTAGTGACTTTCTTAAAATTGGTACTATTCATTGTGTTTTTGGTTGATATAGTGTGTGATGTGATAATGTTGAGTTGATTGATATATTTGAACAAACTATTCATGTTGTTTGTAATGTAAGTATCATATAAGCAGTCCCCCAAAATTATGCTACAGCATGTGAGTCGATCACCACAGCATTAGGAGTCGTCGGCCATATTCTCGATCCGGTCTAAAACCTCGAAAAAAACACAATATAGCAAATATGGGGATGTAAAAGTGTTTAAAAAACCCGTCCCTCTTATTCCCAAACTTTTTCCCCATCTTGAATTTGTTCGTGATTTTCTATTTTCCAATTCACAGCCTTTTTCAATGTTTTTCCTTTTGGATACAAAATATTTTCTGGAAAAATAAAAGTGTAAAAATGTAGATTAATACCCATTCTTTGGATTCCACAAAAAAAACGGTGTTATTACATCACATTTTCACAAGTTTTTGACCGGGTATTGTGTTCTTGTCATTTTGGGTGTTCACTAGGATTTTTAACGAGTACTATATAAAGCTCTATAGAGGGAAGAATTGGCAACTCGATGTTGAATAGCTATAATATAGTTATTGACATTGATTCCGGGCCGGAGGAGTAGGTTGTAATGCATTGCCGAGGCCTTTAGGAAGAATACACAAAAAATCACTTGGGCGAGGCGGAATATGGCGGTGTCATAGCCGAGGCGGGAATATTGACCGGCCATATGCTGGAACGATGCGAGGACGCGACCTGCGAATCTATAAAAGCCGATATCGTAGTGTTGCCTTCGACCAGTGTCCCACTTAGATTGGCGTTGTGCTATGTTAGTTTAGGGTGTTTTCAGTATATTTTCTCGTGGCGACTTTTTGTTGTTTCGGGAATCGTAATTTCGAGGGGATTCCTTTCCGGCGCGGCTTGATGGTACAATGCGACAAAATTCGTTGATGGAGAACACAGTAGTCACAGGGATTCGGCTTTTGTTACCATCGGGCTGGGCGGGCAATGGGCTGCCGTCACACATTTGTCTAATTTCGTAGATACACATAGGGTATCGGGGTGAGAAAAGTGTTGGGGGTGCCTTGATGCGGACTATGTCAATCTGGCGAGGGCGAAAGTATTCTTGATTAGTCCGATTCTTGACATTTTGTGGCATGAATCATCGTAGTAGCGAGTCCCCCAGCTGAGTTAACGATTGGAGTTGTTGTTTCCGCGTTTTGGTGCTAGTTGAGGGATTAGACGAATTCTTGAGTCGTATTTGTGGTTGTGGGTTATGCGCTGGGATTTCTTGTTGATATAGCGTTACGGAACAGTGTAGTAGTGTCGGTTTTTGGGCTGGCGGGCTCGCGTACTTGTGCACATCAGCACTTTATCTTCGATAAACCTCTTCGATGTTTGCTCAATGCAGTAAGCCTTGGGCGAGGTATGGTTTTCTGTGTTTTATTCCCAATGACGGTATTGATGTCCCGTGCAAATCTTTTGTTTTCCTTTTGACATCCTTTTGGGTGTCTGGTGGACCTTATGGCCAGGTCGAGTCCTCCTGACCTCAACGGCGAAAATCAGCGTTGTTGGTCGGGTGTGCAGTCTCCCAGTTGTGCCGGGATGCGGGAATGTTGAGGAATGGGCGTTCATTCGTCGCATTACCTAATTCGGCGTTTCTGGCCCAAGCGAGCGGCGTCAGTGTGCAGGTTGACTCTTCCGTTAAAGGGAGCTCATGCGTCTTGGTGTCGAGCGAGGAGTGCTACAGTTGATCTGCCCGGTAGTCATATGCTTGTCACCGGAATTAAACCATGCATGTAAGTATGAACTAATTGGTTGTGAAGCTGCGAATGGCTCCGTTAAATCGATTATAGTTTGTTTGATGGTACACACGCTACTCGGATAGCAGTAGTATTCTAAGTTGTCGTGCAGCCAAACCCTGGAAGCTTACGGAAGGGATGCATTTATTAGATAAAAAGGTCAGCCTTGGGCTACGCGTTGCTCCCGATGATTCGCGATAGCTCGACGGATCGCGCGGCTGCTCGGCGGCGTCATTCAAATTTACGCCCTATCGACTTTTCGATGGTAGGATAGTGGCTACCATGAGGTGGTGACGGTGGCCAGGAGAATTAGGGTTCGATTCGGGAGGGGAGCACGGCTTGGTTACCACGTCGGAGAGAGGCGGCGGGCGCGCAGGTACCCCGATCGACACGGGGAGGTAGTGACAATAAATAAGCAATACGGGCTCTTTAGTGCAGTAGTGGAATAGTACAATCTAAATCCTTAACGAGGATCCATTGGAGGGCAAGTACGGTGCCCGGCGTGCTTTGACAATTCCGGCTCGATAGCGTATATTTAAGGATTGTTGCGGTTAAAAAGCTCGTAGTTGGACCTTGGGGTGAGTGCGTAGGTGTGCTGGTGTGCGTGGGCGAGTCTAGCCTCTTTCGCCAGGGCGTGCGCTCGGCCTTAACTGGCGAGGTCGTGCCTCACGACCTTCATTGCACTGAAGAAATTAGAGTGCTCAAGAAACGGCTACGCTCTGTATACATTAGCATGGGATAACATTATAGGATTAGGTCTTTATTGTGTTGGCCTTCGGGATCGGGTAATGATTACTTGAGGGGACGGTCGGGGGCATTCGTATTTCATAGTCGAGGTGAAATTCTTGGATTTATGAAAGACGAAACAGCTGAAAAGCATTTGCCGAGATGTTTTCATTAATCGAAACGAAAGTTGGGGGAGCTCGAAGGCTGATCGGATACGTCTAGTCTCGGCAAAAGCAAGCGATCTCAGGCGAGGATCGGCGGATGTACTTTTAGGGCCGTGCGCCTTATGAGAAATGTCAGAAAGTTTTTGGGTTCGGGAGTATGGTCGCGAGGCTGAAGCAAGGGGTGACGGAAGGGCACCACCGAGGAGTGGAGCTGCGGCAATTTGACTGGCACGGGAAACTTACGAGGTCGGTTCATAGTAAGTTGACGGAGCGAGAGCTCTTTCTTGATTATGGAGTGGTGGTGCATCATTGGTTCTTAGTTGGTGGACGATTTATGCAGTTAATTAAGGTGCCAATGACGAGACCTCGGCTTTGCTAACTAGCTATGCGGAGGTATACCTTATGACTAGCTTCTTAGAGGGCCCATGGCCTTTCGGGCCGCGGAAGTTTGAGCGATAGCAGTGCAGCGATGCCCTTAGATGTTGCAGGCGCACGCGCTGCAGCGATGTATTCGGCAGTACATAGCCTTGGTAAGCCAGTCGGGTAATCTTGAAATTTCATCGTGATGGGGATAGATCATTGCAATTGTTGGTCTTCGAGGAATTCTAGTAGCGCGATCGTCGGCTCGCGTTGACCCCTACGTCCGCCCTTTGTACACACTTGCGTCAGGCTCCCCTCACAGATTGAATGGTCCGGTGAAGGGAGTGTTCGGATCGCGGCGGCGTGGGCGGTTCGCCGCCCGGCGGCGTCGCGAAGTTCAGACCTTATCATTTAGAGGAAGGAGAAGTCGTAGAGGTTTCGGAGTAGGTGAACTGCGAGGATCATTAGTCGAAACACGCCCGTGAGCGACCCGGGAGAACATGTTTATCATGAGACGGGGATGTGGTGAAAGCCCCATCCACTGAGCAAGGATGGTACTCAGCCTCGGTGAGATGTCTTTCCGGCCACGATAACAATTTCCAGCGCGGTCTGCGCGAGAACATGGATCTGATGTGCCGCGGTGGTTCGCGGATGTAAGAGGTAACACCAAGTCGTATATAACATTAAGCGACTCTCGGCCTTGTGGATATCTCGGCTCTCGCATCGATGAAGAACGTGAGAAATCACGATACTTGGTGTGGACTATGGAATCCGTGAACCGTCGAGGTCTTTGAGCCTTGAGTTGCGCCGAGCCCCAGGTCGAGGCACGCACTAGGCGTCACGCGTCGCGTCTCCCCCACCCACCTTAGTGGGAGGGGGGCGGAGGATGATGTACCTCCCATGCCTCGTGGGTGTGGATGCTTTAAATCACAAGAGCCCCCGGTTATAAATCTTGCGTTATAGGTGGGGGCTCTGAGAGCTAGCCTAGGAATCGGCATATTCGCTTTTTAGCTTTTGAGGACTCGCGAGGACCCTAAGTTTGTTGCCCTTTGGGGCATCAAAACCGTTGCGACCCAGTCGACGGGGCTACCGAGTTTGCATCTATCCCGATAAGCGGAGGAAAAACTTCTGAGGATTCCTAATAGCAGCGAGCGAGCAGGGAACGGCCCGACTTTCATCGAGGCGAGCCGTCGTCCGAGTGTAATACGGAGAAGCGTCCTCTGCGGCGAGGGCCGGGCCCAGGAGTCCCACAGAGAGCAGGAGAAGGGTGAGAGCCCGTCGTGCCGGACAATCGCACCACGGCGCGTCGCCGAGTCGGGTTGTTGAATACGGCCCCGAGTGGGCGTGGGGTAGATCCAAGGCTAAATGCGGCGAGGCCAGATGGCGAACAAGTGCGAGGAAAGATGAAAAGGACTTTGAGAGTCGAAGGGTGCTTGAAATTGTCGGGAGGGAAGCGGATGGGCCGGCCCGATGTGCCAGTCGGATGTGGAACGGCCCAACCGGTCATGCCGTCGGCTTCGAGGGTGCGGACCGGCCGCGGATTGGGAGCGTAAACCGGTTTCGGGAGCGTCGTCCTCGATCGTGGTAGGCGGCGCGCGGCCGTCGCGCGTGCCCGGCACTGCGCGCTCGGGCGTGGCTGCGGGCCCCCCGTCGACAGTCTTGAAACACACGGACCAAGGAGTCGACATGTAATGCCGAGAGTCGACGGGCGTAAGCCGTACGAGCGCAAGGAAGCTAATTAGGCGGGATCCCAGCCCAGTGCCTTCGTGCGGCCAGACCTTGATCTTCGAGGAAGGGTTCGGTGAGAGCATGCACAGCCGGGACTAGAAAGGGTGGTGTGAACTATGCACGAGGCGGGGCGAGCGGAGGAAACTACGGGTGGGGCCGACGATCTTGGCCGTGCAAATGCGTTCGTCGACTTGGGTATAGGGGCGAAAGACTGTCGAGCCGCGTCACAGTAAGCTGGTTCCCTCAGTTTCCTCGGGATATTTGGAGCTCATTCGCCGGGTTCTATCGAGGTAAAGCCCCGATGATTAGAGGCTGCGGGGCGCAGCGCCCGCTCGACCTATTCTCAAACTTTAAATAGGTAGGACCGGCGCCGAATTTGCTTTGTTAGTAGTGCCGTGGAATCGAGAGCTCCAGTGGGCCCATTTTTGGTAAGCAGGCTAGCGGCAGGGATGAGGCAGGAGCCAGGGTTACGGTGCCCAAGCTACTTGGCATGCCTAGATCCCACAAGGGTGTTGAGTCGATTAAGACGGGGCCGGGTAGTCATGGAAGTGTCAGCTAAGGAGTGTGTAACAGCTTCCGCTGCCGAATCGACTAGCCCCAGAAAATGGATGGCGGCGAGCTGGCGTGAGCTATGCCGGCCCAGTGGGGCAAGTGCCGGGCCAGATGAGTAGGAGGCGCGTGATCGCAAAACACGTGGCGTGAGCCGGGCGGGCTGGCCGTTGAGTGCGGATCTTGAGTGAGTAGTAGCAAATATTGAATGAGAACTTTGAAGGCCGAAGGGAAAGGTTCCATGTGAGCGGCACTTGCACGTGGGTTAGTCGATCTAGAGACGGGAAGCCTGTCAGATAAGCGCGTTTGCGCGAGCAGGAATCAGTTAGAATTACGAGCAGGGCCGGCGTGGCGGTTGGCCAGCGGCGCGTTAGAATCGGAGGCGTCGGCGGGAACCTCGGGAAGAGTTATCTTTGTTTAACGGCTGCCCACCTGGAATCGGCTCGATCGGAGGTAGGGTCAGTGGAAGAGCACCGCACGTTTTGTGTGGTGTCGGTGCGCCCAGTATGAAAATCAGGAGGGCCAAGTGCAGGCCACGCCGGGTCGTACTGCATGTCATGGCATCCCGGGTCTCGAGGTGAGCGACCTGCAGTGCATTCAGAGTGATATGTGAGCGGGGAAGTCGGCAAAATGGATCAGTAACCCGGGAAAAGGATTGGCTCGAGTGGGCACGGTCCCGATCCGGGCTGCGTGTTTGTCGGCGGGCTAACAGGCGCCCAAAGGTGGCGGGCGGAGTCGCTGGCACGTCTTGCCGAGACAGGCCCAGGAACGGTCCGCCCGGGGGCACTTCCGGGCGTCGAGCCGGTCGGCTCGGGCGGGTACGGACAAAGGGAATCCAGGCATTTAATTAAAAACGAAAACATTGCGATGGTCCTGCGGATATTGATGTGATTTGCACCCGGTGCTACGGATGTAAAGTGAAGAAATTGACAGATGGGCCAGCAGAGATGTATGACTCTCTTAAGGTAGCGAATGCCTCGTCATCTAGTGGTAGCGCATGGATGGATTAACGAGATTCCCAGCATCACATGCGCTATCCGAGGCGGGAAACCACGGCCCAGAGGGAGCCGGGCTTAGCGGGATCGGCGAGGAAAGAAAGACCTGGTGAGTGACTCACAGTCGGGCTTTATTAGGAGACGGCTTGAGAGGTGGCAGAGATAAATTGGGAGCTTGGGCGCAAGTGAAATACCACTACTTTGCGTTATTTACTTACTCCTGGTGAGTGGAGACGGAGCGATGCCCACTCCTTTTTGAACTTTGGAATTAAAACTGTAGTGTCAGCGGAGGACATTGTCGAGGTGGGGAGTTTGGCTGGGGCGGCACACATGCATTAAGATAGCAACGGTGTCCTAAGATGAGCTCGACGAGCAATCTCGTGTGGAACGAGGAAGGGTAAAAGCTCGTTTGGTCCCCGATTTTCGATACGAATACGAGCCGGGTGAGCGTGGCCTAACGATCCTTTAGACCTTTTTGGATTTAAAAATTTGAGGTGTCGGAAAAGTTACCACGGGGGATAAGCGGCTTGTGGCGACCAGCGTTCATAAGCGGCGTTGCTTTTTGATCCTTGATGTCGGCTGCTCTTTATCATTGTAGACGGGAATTCACCAAGTGTTGGATTGTTCACCCACAGTAGGGAGCGTGGTGGGTTTAGGCCAGGTCGTGAGACGGAGTTGAGTTTTACCTACGCGGTGGCCGGTCTTCATGTGGTAATTGACTGAGTGCGGAGGAATAGTTGATTCGCCTGATTGGTTAAATCGGCAGCCCCAGTTGAAAGCCCCGATGTGTGAAGCTACGTGTGGCGGATTGTGGTAGACGCCTCCCAGAAGTCGAGTCCCAGGGCGGGCGGCGCGTGCGCCGCGCCGTTAGCAGATCCTACAGGTACAGGGGTTACGACCCCCAAGGGCAGTATGCGTAGGAAGGTAGCTCGCGGTGGATGCGCGCGTGAGGCGCCTTGAAAGTACAATTTCTGCGGGGTTGAATCCTTTGCGGGCTGACTTAAATGCGCGGCGGGAGCGTGTAAGTGGCGGTGGCGCTGGCGCCCGCGATGTGAGATTCGACCATGTCGCCGATTAGATCCTCACACACCTTTATTCCACTTTTAAAACACCTATATTTTGGAAAACGAGGTTAGCGATATATCACAAGTATAATAAAATATGCTAAATGCAGGATTAAGACATGTCCACAAGCCTAAGTGTAGGGATGTTATGCGGTCTGGTAGGTGTGGCATGCCTTGAAGACATGTGCACCCAGAGACCAGCGTACCGGTGTAGAATGTGAAGGAACATTACGAGATATCCGGTCGAGATAATATTATTTCGACCCGTATATGACCATTATCGCTACATACGAGGCGGCGTGAAAGCATAGCTATCTGAGAGGTGTGCAGCGGTGAAATTGACCATTTTAAGGCATATTGGATGGTTCCGCCCCGCCCGATTGGACTCAGAGATTATTAGGAGCCAGTGGTAAACATCTCGGCAATTTTATTTAGGGCTCAAAAAGGTCAAAATTACGAAATTGAAGGTATAATCTCTGGCGGCTTTTTAGGAATTTTGAGGGTTCGGGCAGGTGGGAAAAAATCTCGAGGCCGCATGGGATAAATCTTAGAATTCGTATAATGACGTTGGAAACATATATGAGCCTTATATATTTGGCCTAAAATGGTCGTAGTAGGCTTGATTCTGAGAAATCGGTCTGTCGAAGACGGGTAAATTGGCTAGTTTAAAGGCATATTGGATGGTTCTACCAGGCTCGGTGGACTCCAGGAATATTAGGGACCGTGGTAACATCTAGGCAATTTTTATTTAGAGGCTGAAAAAGGTCAAAATTCCCGAAAATTGAAGGCTATAATACCACTTGTTTTTAGGAATTTTGAGGTTAGAGACCGTGTGTAAAATCTCGAGACCATGGGATACATCTTAGGGTCATATAATGACGTTTGGAGGCATATATGAGCCTTATATGTTATGCAAAATGGCGTAGGAAGCTTCGATTTCTGAGAATCGGTGCAAAATCGGAGCGGGTAAATTGACCGTTTTAAGGCATATTGGATAGTTCTGCCCACTCAGTTGGACTCGGGGAGGATATTAGGGACCATAGTAGCATCTCGGCATTTTATTTTGAGTCAAAATAAGTGGAAAATTGTCAAATTGAAATTTTATAATACGATTTCGGATCAATTTTGACACTTTTAGAAAAATGATACGAATGTGTTGGATGACTCCTGGAGTCATATAAAGGAGGGTTTGAAACCATAAGACAGATAAAGATGTCCGTAAAGTACGGGAATGAGTTTGACTACATTTTTGAAATTAAATTGATGTATAGTGATGTAATAATGCATGATGTGAGCAGTATAATGATGTCATATGTGTATATTGATGTCATATGTGTATATTGAAGTGATGTGACTTGACTACAATGATGTTAGAAAGTATATATATAAAGTGATATGACTTGACTATGATGATGTCATATAAGTATATATGTTGTCGTATCGTAATATGACATGAGTTGAAAAGAATGATGTCATATGGGTATAATTATGCTACATAAGTGATGTAATATAGGTAAAATAGTCATGTGATGTGATGGCACCCATAAAATAAAGTGATGTGATTTGACAATAGTAATGGGAAATGAACACATTATATCATATGATGATTGATTTAATGATAGTGACTTTCTTCATTACTATTCACATGTGTTTTGGTTGATGATGTGTGATGTGATAATGTTGAGTTGATTGATATATTTGAACAAACTATTCATGTTTGTTTGTAATGTAAGTATCATATAACGTCCCCCAAAATTATGCTACATGTATTGAGGAGTCGATCACCTTTTGTATTAGGGTGTCAGGCGTATTCTCGGTCCGGTCTAAAACCTCAGAAAAAAACACAATATGGCAAATATGGGATGTAAAAGTGTTTAAAACCCATCCCCCTCTTATTCCCAAGCTTTTTCCCCATCTTATACAGGTGTTTGGAGTTTTTACATATTTTCCCAGTCTAGCCTTTTTTTCCCAGTATTTTCCCTTTTTGGGGCCCACAAAAATATTTTACGGAAAAATAAAAGTGTAAAAATGTAGATTAATACCCATTCTTTGGAATTCCACCAAAAAAACAGGGTGTTATTATCACATTTTCTGGATTTTGACCCATTGGCCCAGTATTCTTGTCATTTTGGGTGTTCACTAGGATTTTAACGAGGTACTATACTCAAAGCTCTATAGGGAAGGGTAGCAGCTCGATGTTGAATAGCTATAATATAGTTATTCAGCATTCCTCTCGAGGGCCAGGAGGTAGGTTGTGTGCATTACCGACGCTAGAATGCACACAAAAATCTCACCGAGGGCGAGGCGGAAAATATGGCGGTGTCGTAGCTTCGAGGCGCAGGGAATGTTGACCGGCCATATGCTGGAACGATGCGAGGCGCGACCTCGAATCCTATGGGCCAGATATCGTCCGTGTTGCCTTCGACCAATTGTCCACAGATTGACGTTGTACTTTATGTTAGTTTAGGGTGTTTATTATATTTTCTCGTGACGACTTTTTTGTTGTTTCGAAGGGAATCGTAGGATTCGAGGGATTCACTCGGCGGCAGTGGTACAATGCGACAAAAATTCGTTGATGGAGAACACAGTGTAGTGCAGGATTAGGCTTTTGTTACCATCGGGAAACACAGGCGAACTATTGGAACACTTAGATGCATTTGTCTAATTTCAGTGATACACACTTAGGGTGTCACGCGAAGCGGTGAGAAGAAGTGTTCGGGGTGCCTTGATGCGGACTATGTCAAATACGACGGGACGAAGTATTCTTGATTAGTCGATTCTTGACATTTTTGTGGCATGAAATCATCTCGTGTGGCAGTACGCTGAGACACGATTGGAGTTGTTGTTTTCACGTTTTACGGTGCTAGTTGAGGGATTAGACCCGAATTCTTGCGATCCTGTTTGTAGTTGTGGGTTGTGCGCTCGGGATTTCTTGTTATAAACGTTCACGGAACACGTGTGAGTAGTGTCCCGGTTTTTGGGCGGGCTCGGTAGCATGCGTCGAGCAGCATGGCGTAAGCCTCTTCGGTGTTTGCTCCAATCTGCGGTAAGCCTTGGGCGAGTAGTTTTACGTGTTTTATTCTAATGGCCCGGTATTGATGTATGTAAATCTTTTGTTTTCCATGACATCCTTTTGGGTGTACGGAGGTGGACCTTCTCATTGGAGTCGAGTCCTCCCGACCTCGGCCCATCAGCGTTGTTGGTCGGGTGTACGGTCTCTGGTTGTGCCGCGGGATGCGGAATGTTGAGGGGGGGAATGGGCGTTCATTCGTCGCGTACCTAATTCGGCGTTTTACGCTAAGCGAGCAGCGTCGGGTGTGCGAGGTTGACTGCTTCCGTTAAAGGGAGGCTCGTGCGTCTCTTGCGGTGTCGAACGAGAGTGCTACAGTTGATCTGCCGGTGAGTCGTATGCTTGTCTGAAAGATTAAGCCCCGTCGTGTGTAAGTATGAACTAATTCGGTTGTGAGCTACTTGAATGGCTCATTAAATGGAGTTATAGTTTGTTTGATGAATTTTCTTTACTTTCGGATAGCAGAAGTGGCCAATTCTAGGCTAATACGTGCAGCAAACCCCAGAGCTTAAGGAAGGGATGCATTTATTAGATAAAGGTCGGCCCAACGGGCGGCTACGCCGGATTTGCTACGATGATTCGCTGATAACTCACCACCGACGGATGCTTTTGGACTAGCATGGCGGCGCATCATTCAAATTTCACGCCTATCCCAACTTTCGATGGTAGGATAGTGGCCTACCATGGTGGTGGCAGGTGGCCAATTAGGGTTCGATTGGGAGGGGCACGAGCGGCTTCTACAGCGTCAGGAAGGCGGCGGCGCGCGAGTGATCGACACGGGAGGTAGTGACGATAAATCGCAATATCTAGGGCTCTTTGAGTCGGTAATTGGAATAGTGCGATCTAAATCCCTTAACGAGGATCCATTGGAGGGCAAGTACGGTGCCGGCCCGGCGCGGTAATTCCCGACTCGATAGCGTATATTTAGTTGTTGCGATTAAAAAGCTCGTAGTTGGACCTTAGGGTGAATGCAGCCCAGGTCAGCCTTTAGTGTGCCGTGTCTCGCCTCTTTTCGCCAGACGATGCGCTCAGCCTTCGCGGCCGGAGTCGTGCCTTACTCCGACAACTGTTACTGAAAAGAAATTAGAGTGCTCAGCCTTGAAACTACGCTACATACATTAGCATGGGACCAACATTATGGGAATTGAGTCCCTATTGTGTTAGCCTTCGGAGATCGGAGTAATGATTAGCAGGGGCAGTCGGGGGCATTCGGTATTTCGTAGTCGAGAGGTGAAATTCTTGGATTTATGAAAGGCAAACAGCTGCGGCATTTGCGAGATGTTGCTGGCGTGTCAAGAGCGAGTTGGGAGCTCGAGACGATCGGATCTGAGTCTAGTCTACGACCATAAGCACGATGCCAGACCCGGGGATGTGGATGTTACTTAGGGCATGGCCAGGCTGCCTGAGAAATCAAAGTTTTTGGGTTCGGGAGTATGGTCGCGAGAATTTTAGACTTAAAGGGTGTGGAAGAGCGCCACGGGAGTGGAGCTGCGGCTTAATTTGACTGGCGGGGCTGCAGGGTCAGGGCGTAGTAAGGATTGTGGTGAGAGGCTGCTGGCGGTCCCATGGGTGGTGGTGCATGAAAAAGTTAACTTGGTTGGTGGGCGATTTAATGCAGTTGAATTAGTTGCGACGAGACCTCGACTGGTAACTAGCTATGCGAGGTATACCTTCCTTGTTGTAGCTTCTTAGGGACTATGGCCTTTCGGGCCACGAAGTTTGTGACGATAGCAGTGTGATGCCCTTAGATGTTACAGGCGCACGTTACGCGATGTGTTGGCGAGTGCATGGCCTTGGCCAGAGCGGTCGGATAATCTTTGGTATTTCATCGTGATGGGGATAGATGGGTGCAATTGTTGAGTGCTTGACGGGAATTCCTAGTAAGCGAGTCATCGGCTCGCGTTGACTACGTCCACGCCCTTTGGCAGCGCACCTGGCCGTCGCTCACTGGGTGAATGGTCGGGTGAAGTGTTCGGATCGGCGGCGTGGGGCGGTTCGCCGCCCAGCGTCGCGAAGTTCAAGCGAACCTTATCATTAGAGGAAGGAGAAGTAAATAACAAAGGTTTCGAGTAGGTGAACTGCGCGGAAGGATCATTGTCGAGAGCACATGCCGGCGGAAGCGACCCGGAGAACGTATTTATCATGAGCGAGGGGATGTGGTGAAAGCCCCGTCCACTGGCAGGATGGTACTCGCCTCGGTGGGAGGTATCTTCACCGGCACGATAACGAACCGGCGCGGTGCAGCGCGAGGAACATGGATACAAGTGTGTGCCGCCCTGGTGGTTCGCGGATGTAGAGGGGTGGTATAGTCGTATATAACGATGCAGCGACTGCGACGGCGGATATCTCGGCTACCGCTGTCGATGAAGAAGCGTGGCGAATGCGATACTTGGTGTGGTGCCGAATCAGTGAACCATCGAGTCTTTGAGTATGAGGTGCGCCGAAGCCTTTGGAGTCGAGGCACCGCTGCTTTGGGCGTCGCGCATCGCGTCTCCGCGCCCAGGTGGGAGGGGCGGAGGATGATGGCCTCCGTGCCTCGCGGTGTAGGATGGCCTAAATCACAGGCCAGTTGTAAGTGCCGCAGTATCGTGGATACAAGGCCTAGCCTAGGAGATCGGCATATTCGCGCACTTTTTGGCTTTTGAGGACTCGCGGGGACCACTAAGTTTGTTTGCCCTTTGGAGGCGTCAAAGCTGGGTGCGACCCAGTCGAGACGGGGCCCCCATTGAGTTTAAGCATATCGATAAGCGGAGGAAAAAAAACTTACAAGGATTCCCTAGTAGCAGCGGCCAGGAGCATTGACTTTAAATCGGGCGGCCGTCGTCCGGGTGATACGGAGCGTCCTCTCTCTGCGGCGGACCGGGCCCAGTCCCACTGGAAAGGGGCTGCGAGAGGAAGGGTGAGCCCGTCGTGCCGGACCACTGTCGCACCACGAGCGGCGTCGCCGAATCCCGGGTTGTTTAGAATGCGGCCCAGTGGGCGGTAAATTCGTCACGTAAATCTTTCGAGGCAGATGAGGCTGTCTGCTTGAGGAAAGATGAAAAAGGACTTTGAAAGAGTCGAGTGCTTGAAATTGTCGCTCGGGAGGGAGGGCGGATGGGGCGGCGGTGTGCCGGTCGGATAATGGAGCGGCAGCGGTCCGCGTCGGCTTCGAGTGCGGACAGACGCGGATGGGAGGGCGGCTAAACCCTGGTTTGGGGCGTCGTCCCCTCGATCGTGGTAGGCGGTAGCCCAGCGCGGAAATCACGGCGTGCTTCGGCTTTCTGCGCGCTCCAGGCACGTGAAGCCTGCGCGGGCCCAGATTCGGCAGATCTTGAAACACGGACGAGAGTCGACATGTGTGCGGTAATGGCGAGTAAACCGTCTGGCCTTGAGGAAGCTAATTGGCGGGATCCCAGCGGGTGCACGTGAAGCCCAGGCCTTGATCTTCGAGAAGGGTTGGTGAGCGTACATCGGACTACAGAAGATGGTGAACTATGCTGACGGGCGAAGCCGAGAGAAACTACGGTGGAGGCAGAAGCGATCTTGGCGTCTGGAAATCGTTAAGTCGACTTGGGTAGGGGCGAAAGACTAATCGAGCCGAGTCTAGTAGGTGAAGTTCCCTCAGAAGTTTCCTCGAGGATAGGCTGGAGCTCATTCGCGAGTTCATCGGGTAAAGCCTTGATGATTAGAGGCTGCGGGCGCAGCGCCCTAGGCTTTATTCTCGAGCTTTAAATGGGTGGGACCAGGCGCGGGCTGCTTTGTTGGCTGTGCCGTGGATCGAGAGCTCAGAGTGGGCCATTTTTGAGTGGCAGAGCGCGTGCGGGATGAGCCAAGGCCGGGTTACGATTCTTGACTACGCGTAACACAGATCCCATGAGTGTTGGTCGATTAAGGCTGACGGGACGGTGGTCATGGAAGTCGAATCAGCTAAGGAGTGTGTAGCTGACTCACTGCGAGATCCATTAGCCCAGAAAATGGATGGCGGCTTGCGTGACCTATACCGGCCGTCGGGGCAAGTGCGAGCCCCGATGAGTAGGAGGGCGCGTGATCGTGCCAAAACACAATGAGTGCAGGCCGAGGGCGGAGACGGCGGATTGGTGCGAGATCTTGGTGGTAGTAGCGAAATATTCCAAATGCAAAGGCTTTGAGTAGAAGAGGGAAAGGTTCCATGTGAGCGGCACTTGCATGGGTTAGTCGATCTAAGAGACGGGGAAGGCTTTGTCCCAGATGCGTTTCGCGCGAGCTTGAGGAATGGGTAAAATTCACGAGGCAAGGGACCGTGGCGGTTGGCAGCGTTAGGAAGTCGGAGGCGTCGGCGGGGGCCTCGGAAGGAGTTATCTTTTTCTGTTTAACGGCCTTGCCCACCACGGAATCGGCTTCGGTCCAGGTAAGTCGTGTTTGAAGAAACACGCACGTTTTGTGGTGTCAGAGTGCGCCCGTGACCCTTAATCAGGAGGGCCAGAGGTCACCAGGCCACGCCGGTCGTACTCATAGCATCCCGAGGTCTCAAGGTGAACGACCTGCAGTCGATGGAGACAATGAGTAAGGGAAGTCGGCAAAATGGATCAGTAACCCCGAAGGAAGTTGGCTGCGGGTAGGCACGGGGTCAGTCGAACGTCAGTGTGGCGGTTTCGCTCGGCTGCCCGGGTGGCGGGCGGGTCGCCCGCGTCTTGGCCGAGAGAACAGGTAGGAAACCGGATATCAGGGGGCCTTCCGGGCGTCGGCCCGATTGACTCAGGCCAGTACAGGACGAGGAATCCGTACATTTAATTAAAAGCAAGCATTGCGTGGTCCTGCGGATGTTAGCAGCAATCATGAGATTTCACCCGATCTACGAATGTCAAAAGTGAAGAAATTCAGCAAAAAGCGCGGAGTAGCAGCGGGAGTAACTATGACTCTGTTAAGGTAGCCCAAAATGCCTGGAAATCGTCTAATTGGTGGCGCATGAATGGATGAGATTCCCCTTGTCACATGCACTATCCCGGCGAAACCACGACGAGGAACGGGCTTAGCGAAGTGGGTGAGGAAAGACCACACTGGGCTTGACTGGTCAGGCTTTGTGAAATGACTTGAGAGGTGTAGGATAGAGTGGGAGCTTCGGCGCAGTGAAATACCACTACTTTTAACGTTATTTTGCCTCGGGTGAGTCGGAAGGCTGATGCCCCTCCTTTTGAGACTAGGTCGCTTTGCCCGGTGCCCGTCCGAGGCGGAGGATTGTCGGAGTGGGGGAGTTTGCGTTTGGGGCGGCACATGCATTAAAAGATACTTGCAGTGTCTAAGATGAGCTCGAGCAGACGAATCTAGGTGTGGAACGAAGGGTAAAAGCTCGTTTGATTACGATTTTGATGCCGAATACGAGCTTGAGTGAAGCGTGGCCTAACGATCCTTTAGACCTTTTGGATTTAAAGCTAGAGGTGCCAGGAGAAAAGTTACCACGGGGATAGCGGCTTGCTGGCAGCAGCGTTCATAAGCAGCGTTGCTTTTTGATCCTTCGATGTCGGCTCTTGCTATCATTGTAGGCGGGAATTCACCAAGTGTTGGATTGTTCACCCACGATAGGAGCGTGGTGGGTTTTAGGCGGAGTCGTGAGACAGGGTTAGTTTTACCTGCGATGACGGTGTCGCGATGGTAATTGACCTAGTACGAGAGAGGCAGTTGATTCACACGATTGGTCGTCGGCTTGGTTGAAGCCGGTCGGCGCGAAGCTGCGGGTGTCTTTGGATTATGGTGACGCCTCTAGTCGAGTCCCAGGGCCGGAGCGGCACTTGTCGCCTTCGCCGTTGCGATCCTACGGTAGGGGTTTCGGCTGAGGGCACGTGTCGTGGAAGGCTAAGCTCGCGCGGTGGATCTTGTAGTGAGCCATGGCAATCTGTTCTCTACGGCCGTGGGTTGAATCCTTTGCGAGACTGACTTAAATACGCGACGGGGTATTTGTAAGTGAGCGAGTGACGCTGGCGCCCACGATCCTTTGAATTCGACCTATGTCGCGATTAGTCCACTCCCCCCCCCCTTTTATTCCACTTAAAAACACTATATTTTGGAAAACGAGGTTAGCGATATATACGTATAATAAAATATGCTAAATGCAAAGGATGGAGCATGTCCCGCAGCCTAAGTGTGGGGATGTTATGCAGTGCAGTAGGTGTGCGTATCTGTGAACATGTGCACCCAAGGGCCAGGCGTACCGGTGTGGGAATGTGAAGGAACATTACGAGATGCATCCAGGAATTGAATAATATTATTCGACCCATATATGACCATTATACGGCTACATACGGGCGACGTGAAAGCTGTAAGCTAATACGAGAGATTGTGTCAGCAGTGAAATTGACCGTTTTAAGGCGTATTGGATGGGTTCACCGCTCTCGGTGGACTCCAGAGGGTGTATTAGGGGCCGTGGTAAACATCTCGGCAATTTTTATTTCGGGCTCAAAGGTCAAAATTCAAAGTGAAGAGGCATAATACACGCTTTTTTAGGAATTTTGAGTTCGGGGCAGGTGGCGAAGAATCTCGAACCACATGGGATACATCTTAGGGTCATATAATGACGTTTGGAAGCTTATATATAGGCATATATGTTGTAAAATGGTAAATTAGTAGGCTTCGATTCTGAAAATCGGTCTGTCAGACGGGTAAATTGGCCGTTTTAAAGGCATATTGGATGGTTACCCATCAATTGGACTCCGGAGGTGTATTAGGGACCATGGTAAACATCCCCAGAGCCAATTTTATTTAGGGCGAAAAAGGTCAAAGTTCAAGGTGAAGGCTATAATACACCGCTTTTAGGAATTTTGAGGGATTCGGGGCCCAGGTGGCGAAAATCTCGAGACCACATGGGATGCATCTTGGGTCGCTATAATGACGTTGGAAGCATATATGAGCCTTATATGTTGTACAAAATGGCGTGGAGGCTTCGGTTCTGAATCGGTCTGTCGGGCAGGTAAATTGGCCGTTTTTAAGGCATATTGGATGGATTGCCCACTCGGTGGACCGGGAGGATTAGGGACCATGGTAGCATCTCGGCATTTTTTATTTTTCGAGTCGAAATAAGTGGAAAATTGTCGAAAATTGAAGGGTATATTACCAGATTGGATCAATTTTTGACACTTTCAAAAATGATACGAATGTGTTGGATGACTCACGGAGTCATATAAGGAGTTTGAAACCATAAGACAGATAAAGATGTCCATAAAGTACGAATGAGTTTTGACTCTATTTTAGAAATTAAAGCTGATGTATAGTGATGTAATAATGTAGTCTTGAAGGCTTGACTATAATGATGTCATATGTATATTGATGTCATATGTGTATATTGAAGTGATGTGACTTGACTTCTATTGATGTTAGAAAGTATATATAAAGTGATATGACTTGACTATGATGATGTCATATAAGTATATATGTTGTCGTATCATAATATGACATGAGTTAGAAAAGAATGATGTCATATGGGTATAATTATGCTACATAAGTGATGTAATATGGGTAAAATAGTCTCGTAGTGATGTGATGTACCATAAAATAGAAGTGATGTGATTTGTGATAGTAATGAAATGAACACATTATATCATATGATGATTGATTTGTAAATAGTGTGACTTTCTTCGTTATTTACTATTCCGCATGTGTTTTAGTTGATATGATGTGTGATGTGATAATGTTGAGTTGATTGATATATTTTGAGCAAACTATTCATGTTTTGTTTGTAATATGAAATGTCATGCTCTTGATCCCCAAATTATGCTCTGTTGTATTGAAAGTGTCCTTGCGTATTGGGGTGTCAGCATATTCGGTCCGGTCTAAAACCTCGAAAAAAACACAATATGGCAAATATGGGATGTAAAAAGTGTTTTAAAACCCGTCCCCCTCTTATTCCCAAGCTTTTTCCCACGTCTTAAAATTTGTTTCGGCGGTTTTCTATTTCCCGATTCACAGCCTTTTTTTCAGTATTTCCCTTTTTTGGGGGCCCTTGAAAAATATTACGGAAATAAAAGTGTAAAAATGTAGATTAATACCCATTCTTTAGTTCCACCAAAAAAAAACAGGGTGTTATTATCACATTTTCACGAGTTTTGACAGATTATTATTATTCTTGTCATTTGGGTGGTGTTCACTAGGATTTTACTTGGAGTACTTATATAAAGCTCTATAGGGAAGGGAGTGGCAACTCAATGTTGAATAGCTATAATATAGTTATTCAACATTGATTCCTTCTCCGGGCCCGGAGGTAAGGTTGTGTGCATTGCCGGGCTAGGAAGAATGCACCGCAAAAGAAATGCGCCGGGGCGAGGCGGAAAAATGACGGTGTCGTAGCCGAGGCGGAATGTTGACCGGCCATATGCTTGGAACGTGCGGTGCGACCTCGAATCTATGAAGCCGATATCGTCAGAGTGTTGCCATTGATTGTCCCTGCAGATTGACGTTGTACTTTGCTATGTTAGTTTAGGGTGCTGCCAGCTATATTTTCGTAGCTGACTTTGTTGTTTCGGGAATCGT

>read 131

ACTCGTGACCCCAGGTTTACTAAACTTGGACCTTCTCTCCTACTCTCCTGCTTATTTTCCCTCCTTACTCTAATTTTCCCTTTCTCTCTCTACAAACTCTCAAACCTAACCCCAATTCATCATTCCTTACTCCTTAATTCAAATTTCCATCAAATTCTTCCTAAAATCAACCTCATTTCTCCATTTTTCATCCTTCTACTCATATTTTGGCAATTTCGGTGGTGTGGGGTACTCTTTGATGATTTGAGTCCCCGATCTTGGTATACTTCTAACCTTTGTTTTAATTCTTGTCAATTTTACTTGATTTCTTTACATTGGTTGGTATTATCTTGATTGTGGTATGATTTTTGTATTTCATGACTATTGTTTGTGAAATTTGGAATTTTGAATGATCTTGTTTTCTTGAAAAAAAACTTGAATATATTTGATGGATGATTGTTGATATTGGTATTTGTTATTGGAATTTTGGTTGTGGTGTTGATTGACAGGTACCTTGTAGTTTTGGGGGTTTCGGTTGGATTTGAAGGAGGGTTGTGTGAGAAAGCTTGATAGTTGTTGTTTGATTTGGGGAGTTGGGCTATTTCTTGAGGATTTTTGTGTAGTTTGTTTCATATACAAAACGAAGAACCACCATGAATCTCTTGAGGCCGTTAGAAAATTACTTAAAAGCCGCTCCACCCCAAGTCTCAGCCAATCTTCTCAAGGTAATCCTCTAATCCTAACCCACAAACACACCCCTCTAACCAGATGGCGCCCCTCCTCCTCTACCTCCTCTGAGTGTGGAATGGTCTAAACAACTTCCCGTTTACAATAGATGAAAGATCGTGGGTTTAGACCCACTCGCCACTATTCCATGCTAATATATGGCCAAGCTAGGGATTGATAATGATCTTGAGGTTCTTTTAAACAACCTTAGCTTAGGTGAATTCACGAAGTACAACCCTCGAACTTACTTGGAGCCATGATCAATTCTTCTCTCTTTCGAGTTATTAGGGTTGGGGACAGGTCCATCATCTCCTTCCAGCTTTCAACATTAAACACACAATGACCCTCACTTGAGTTCAATGTGCCCTCGGTTTCAGCTTACGGATGGTGATATTTTCAGACCTCTTATCACCGCACTTGAGAGTGGGTACAACGAGGTTTCTTGGTGGAGAACCATAGCCTGGCTTGAGTTTGACTCCAAAACTTCCTTGCTGGTGAGATCATTCATCCGGCCCTTAGGATCCTCCATAGAATTCTTGGGTACTCGGTTTATAGCAAGCAAGACTTGGGTAAAGTTGTCTCGAGAGGAGATCATGGTTTTTGTGGATGGCTACAAAGCCGAGAATTGGGTTCTAGAATCAACACAGGTACATCTTTATGGAAGGGTGTAAGGAGATCATGCACGGTGGCAGGTGAGGTTCATTGTGGGGGCATGGTGACCCGGTTGGTAGGTACTTCCAACCAAGAGAGGTTCTTGGAGTCTCTTGCACTTTCCGTTGGAGAAGTGAAGACATTTGGATAAACTTGAGACAAGTTAACTTTCCATCATCACTAGCACTTTTGAGGGGCCGGAACCTACTCTTGGCTTGTTAGAACACCACCACTGCCTTCTCCCTAACCCGGCGCAACCTCCATCTTTCTTGAAGCTAGAAATGTGTGGATAGTCCCCAGGGACAATGGTAGGGTTGATGAGGATGAGATGGAGGAGGATGCTCACTTTGTTGAGGGTTGGGTTGGGAGATGTGAGCGAGGGAGGAGGAGGAAGATAGAGGAGATGATGATGAGGAACAACAACAACAACAACCATTTCAACAACAACAGCAACATGGAAGGAAGAAGGCAAGCGACAACAACAACAGAACAGCACAGCCACGAGTCATATGACATTGAGCAAGATATGGCGGCTATGTAGGCGCGAAATGGACCCATGAGACTCTCAAATGCATGGTATGTGTCGACCACCAAACTCTTTCTTATCAAGCGGGCTTTATATGATGCTGGTATGCGTGGGGAGATCACACCCCAGGTCGGCTATCATATGTGCCGCCCCAAACAAGGGCCGGTGCCTTCACGAGGTGGTGATGGCCTCCTCATTGCCCTTGTTAGCGAGGGTTGAGAACCCCCGATGTGCCCAAGCGCGACTTCCCGCCTTATGACTAGGGCCCCGGGGAAGTGATTATAAGGGTGACCACAGCCTATTATTATGTTGACCCCATGCTTTGAGCCCCTCATTCGATACCACCCTTTGACCCGATTGACCTATCCCGGCAAGACCTATTCAAGGGTTATTATGGTTTTCCGCCCGGTTTTCGCTGGGAGGGTTGGGACAGGGCGATGGTTTAGGGAGTGATGATGGTGCCGGTCAAGTGGCCATTAGTCCTTTATTTGCATTTGTTTCACCTTTTGCTCTCCCCTTTCCTATTTTGCATGGTTGATGGCATCGAGTCGATGCTTTCATTTGTGAGGGAGAATTCGAAAGGATGTGTTGCATGGGGCCAGCACCTTTGGCTCAGTGGGAGAGTGCTTTGATAAGGTTTTTTGGTGGATCTTGCTATTTGGTCTTGTTTTTGTTGTTGATTATTTCTTTAGATGATGTTTTGGGTTTCTTCTATACAAGTTTTGATGATGATGGGTTCTTTTAGGTTTTGTATATAGTTAGTGTTTTTCGTTTTTTTATTTTTGTTTGCTTTTGTGTGAGGAAGTTATAACAATTTCACTTTGTATATATATAGCTTTCTTTTCTTTTACTCTGTTCGTAGCTCATGCTTTGGATTACGGAGCCCTTTGGGAGAAGCCTCTGCGAATTCTTTGGGTACCTTAATCTGTACGGGCCCTTGGTGAGACCTTTCTTATTTTGCTTCTTTTGTCTTTCGCTCCGTTTTAATTTTCTTGGTTGCTTAGATTTAGATTAGACTTAGGGGAAGTTGAGCAGTTCGAGGATGTATGATTTAACACCTAGTGTATTTGGGGTCTTTGGCGATGTGGCCATTAGACCCTTGTATGTCGATTGGGATAAATTTGTTTTGATCGCATCTTTTGCCGTTTCTTCTTTCTCTTTTCTTATCACTTGTCTAGGTTTTCCGTCTTTTCTTTTCTTTTCTTTCTTTTTATTCTTGTTGGTGAATATCTTTTTGAGGCTTTCTTTCTTACCTTTTTCTTTTTTGAAAGTCGGACTTGTTTGCAATGGACTTTTGCTCTTGAATAATAGTCTTCCTAGGTAATTATGGTATGCACGGTTTGAGATTACGTTTGGTTGACTCTAGGATAAAGCATGCTGAGTAGATGTCTAGAACTAGAGGATCACGGCAGTTCTACTTGTTTGTGGCATGTTTGGTAGTTGCTCCTATCTTGATTTGACAACCTTAGTGAGTTTTGCTACAACCATGTGAGGTGTTTATTTGTTCAAGCTTTCATCACATTCTTGCATAAGCTGGCATGGATCCCTTGATTTTTCGCAAACCTAATCCTAGAATCCCAGGCATATGATTCCCAGAGACGTGACCCGAACTTCCTAGGAAAGACAGGCACCTTTTGAGGTGAGTATTTTCATTTTAGGATAGTTTGATTCAATTTGTTTTGGGTTTGTTTGCAGCCATTTGAGCCTTTACCCTTCTTTTTGACCACATCTTTAGATATTTGGCCTTTGTTTCATTTTCTTTCTTTCTTGGTTTGTGAAGCAAGCAGTCATTCTTGATGGAGTTTGTGGTGGAGAGTTGGATATTAGGTTTACATAAGTTTGCAAATTTATATCTTTGTGAATAATGGCAAATGTTGTGTAAAGCCGTCCCTGAAATCCATAAAGGCATGGACTAATTAATTGCATAAAAAAAAGCCGTGCGATTTTATCAAAGAAAGGCAAGGACATTACCAAGTTTAAATGAAAAAAAAAAAAAAAAAAAAAAAGAGGTTAGAAAAAAGTAAAATTCCAAAAAAATATTTGATAAATTCTTTCCTTCTAAGCATTGAGGACAATGTTTCCTTTAGCTTAGGGGGGAAGGCGTTTTCCCTTCACGCTTTTTGGTCTTTTTCTTGTAGAGTATAGTGTTGAATAAGCTTGGGGGTGTTATTTCATTTTTGTTTGGTGATTATTTAAAGTTTGGTGTTGAGATTTGGCTGAGTCTTTTGCTTCACATTTTAGGATAACCACCAAATTTTAAAGAATTTTTAGCCTACCTTAACCCTTCTTGCCCTACGTTACAACTAGCAGGAAAGACCTTTTGACTTAGGAGTTGGAGTCTTTCATCGGTGGAGGGAATCATAAGTTGTGGACTAAGGAAGGGGATGCGAAATCGAGATGTCGAGTAGCCTAAAACTTTGATCTTTTTCCGGTTCTTCCTTGGAATCTATGAGATGCTCACTAGGGGAATGTAAGGATTAGTAGAGACGGTAAGATTGTATGGGATGAAGGTGGAGTGTGATAACTAGTTCCTATGCTTAATATTTATGTTTCATTCAACATAGGTATCTTTAACGTAAATACCATAATTCGCTTTAGAGATTATTTATTCATTTGTGCTATAGTCTCTTTGTAAAGTCCATTTCCTTAAAGATTTTGTTTCTTTTTCTTCTTTGTGCTTTCCTTGGACCTCTTGGGGTTTGGTGGAGAGTTATGGGTAAAGCTCTTTGGAAACCCTTACGGGTGCTCAGATCCTCTTGAGCGATCTTGGGGTTTAAAATGGCTTGTTGTAGCTAAAAACAATAGTGATTCCTACGACAGGTGAGTCCTAGTGTATATTCTTACCTCCTTTTCTTTTTACTTGTCCTCTTATTCCTTCTTGGCTTTTGTTTGCTCGGACAATGAAGTGCTTAAGGGCTGAGTAGGTATCATTTATGGCACTCATAGGGTAGTGTTTTAAGGTCTTTTCTTTCCCTTTTATTTACTTTCTTCTTTAGTTTTGGTTCTTTTCTTTTAGTTTCTCCCTTTTCCTCTTTCTCTTTGTTTCTCGTATTTTTTAAGTGTTTTCCTTTATTTTGCACTTTTTTTCCCTTTTATTTCCTTCTTTTGTTTGCTTGTGTAGGTATTGAGAGTTTCACTGAAAGCGAAGGATACAATTCGGGACCTTAATGATCAAGCAAGGAAGAAGCATTGGGCTAGAGAGGGCTTGGTATGGAGGCCTCGAGGAAGTCTCAGTCACCGGGCGCGGGCGCCCAGCAGTCTTGGCCATGGGCGCGGGCTTATGATAAATCAAAAGCGCAAAAGTTGGCGGGAGACTTGCAGCGGTTGCTTGGTCTCCGGGCGCGGGCAAAGTGGAGGAGGCCTCTCCACTCGAGGAGAGACCGCATTTGATCGACCTTTGGGAAGGCGAGGATTTGGCGCGGGCGCGGGTCATCTACCCGGAGCGGGCCAGTGCCGCGGGCGCGGGTCTTGCCCCGAGCGGCCCAGCGGAGGGTTTCGGTACCCATTTTCCGCAGCGCGGGTGCATCGGGAGAGACCGCGGGGCTGCGGATTCTTCTTTGTTTATAAATAGAGTTGTATTTCCCTTTTTAAAAACTAACTTTTAGATAATTTTTGATTAGCTTAATTTTTAGAGAGAAATTAGAGAGAAGATAGAGAACTTTTTAGCTTGGTTGTAGCTCAACAATTGATGATCAAGTTTGGATTTCTTCATCAATTTTTAGTGAGATCTTCTTCTTTCCTTAATCTTTACTTACTTGTTATTTGTTCCTTTTGTTGATGTTTGATTTTGCTTGGGAATTAGATTGAGATATTATTGAATACTTTGATTTCTTTTGGATTTAATTTAGAATGTTGATCAATGCAATTTCCTAGTTATTTGTTCTTGAATTTCATCTTGATTAGTAGGTAATCTAGTTGCTAGGAAGGGAAGCATGTTTGATTGATGGAAATGCTAGTTGGAAACTCTTGATTGGTTGATTTGTTGCAATGAATTTGGTTGACATGGTTTTTGCATTAGATAGATTAGACTGGATTGATCACCCTATAAGATTAATCTAGATTGCATTGCAACTCTTAATGACATGACCAATTGAATTAAAAGTTGCTTTAATTGATTGAGTTTGTTTATGATTCTTCTTGGTTTGCCTTTCATAGTGACCACTAGATTGGCCTATAGGAATAGTAATTGAACTCTCTTGATTACTATTGTTTGTCCATTGCAATCATTTGAACTAATTGAATGAGTTTAATCTAGCATTATCATTGGTTTGCATGGAACCCATCTCCTAGTGTTTACACTTTTGATTTAGTTCATTTAGTACAAACTGATATCTCATTCCCGTCGAGGTAGCTTGACTTAGACTTAAGTTTTCATCATTGCATTCCCTTGGGATCGATCCTTAACTTACCTATATTCATATGGAGTTGGTTAGGTGTTTACAAATTTAGTTTGGCTAGGGAAAGGGAGTAGTAACGACATGCATTTTCCATATCGAGGAGTTGAATTGTTGAACAACTGAATTTAATCACTTAATACTTTTATTGGGTTTTGAATAAGGCCATAATGGTTCCAAGTTGTATGACTTAATTTGATTTCATTCGTTGGTTTGGAAACTTATTTATAACTAATTTAGTTTTCCCATTTGCTATTTTTACGATAAAGCCTTAACGTTTGAACAAGTGGCGGTACGCGGTAATTCTCTTGATTATTGTTCAGGGTCACGGAGGTTTAGAGAGAATTATTGGGGTGTTACAAGTGGTATCACGGGAGCTTTAGTTCTATTTAATTACCCTTTAAAGTTACTTTAAAAGTTTTGGGCGCCTAATGTTTTAAATCTTATGATTCTGGATTTTATGACGTGATTTAAGGTTAAACTTTAATTTTCAAAAATTATTTTTATTTTAATTAAATCATTGGCGGTTGAAATAAAATACATGTATTTTTATAAATTACTATGTTATTAATTATTTTTGAAAATTAGTTGAATCTGCGTACTTATTTTGTTCTTGGTCCAGCAGTATTAATTTATTTTATTTTAAGTGTGAGATGTGGGTAGTCTTAAGAAGGGCATATAGGACGGATGATTTCTTTTTCTGCATTATAATTTAATTTTGGTGCCATTTATTTTGAATGTAGTTTTATGCAAGTTATATTTAAGTGTATATGTTTTATATAATCCATGTAAAATTTGGGTGTTGTGATCACCTAGAATGTGTAAAGTTAAAGAATACAATATAGATTAATTTGTAGATGATTTGTGCAGTGTAATGCGAGTAATGTATGGATGATCATTATATGAAGCATGTAATTGTGACTATTTGGTGTCTTATTTAAGTGTTATTTATTATTTATTTGGTGCTTTAGCTTATTGGTAAGATCTATATTTTATTATTTAATATCTCCTTCAATTTTGCAGCTTGATGAGTGTTTATTTGAAACCTTATGATCAAATTAAGACGTGCTTGGAGCAGTGGTGAATATGTTGGTGATGATATTCCCGAGAGGAGTGTATGAATTTTGCTTTAGTCCAGCGCATACTTTAGGAAATTGGAGACCTTGCTTTTTTGTAGAGTATTTGGGCGTTGAAAGATGAGTTTTTGTTGTTTCCAGATTGCCATGAGAATTTGAGGAGCATAACTTTGGTGGAAAGAATAGGGATCTGATTCGGTAGACAATTGACTTCGTTAGGAGAATTTGAAAACCAAGTTGGAGAGAAGAAAATTTTGGGGAGTCCATGGTTATAAACATGGAAATATAAAGTAGACCTTCTTGGAGTTAATAATGTATTAACAATCGTATAGTTTAATGAATGAAGTATTTCCATAAATCTGATGATACGATGCCTGCCAAAAGCTGTGTGATATCTTGCTATACTTGAAGAGTAGGAGAATTATAATGAGCATTTTGAGGATTACGTGACATTTTAGGGACTGATTTGTAATATGCTCGGTTCAGAGTGTGAATTTTATTAATGAAAGTAAGTTTCATTTGGGACAAAAAAATTAGTGGTAAAGTAAGTGGTCTACACCCTAAATGTGACCCGATATAAGGAGTTTATTGAGATTAGATGTTTAAAGAAATTTGAAGGACTTTTGAGGATTGTTGTATATACCGCCAGTGGAAAGTTGGTTTAGAATGAGACTGAGAAGTGTAAACAAGCCTTTAAACTCTTAAAGAACATTTAATGGTGCACTTGATACTAGTCTTGCCTAATAGGAGTGGTGGCCTTGAAGTTTTACTTAATGGATGTGTGTTTGCAACAAAATGGAAAAAAATGATATTGCTTGGCCAAGACAAATCTTATGAGACCAATTACCACCCATGATCTTGAGATTGCATATATATTGTTTACATTGGAGTTGTTTGAAATACAATGTTACTGTGGAATTTTACAGCTCTTAATAGCCTTTGGTTGTTAGCTAAGGAGATGAAAGTTGTGAGTGCCTATGATTTGAGCTATTCAAATTTGAAATTATAGAATAACAAGTTATGGATAAATTATTAGGAAAGAAGAAATGAAAAGAAATGCCTAATGATATTATTGTGATGAGTTACGGGCGTAATAAACTTTTCTTCAGGGTAGAATGCGATGTAATTTCGCTTTTTACCTCAATTATGCTATTTATTGCTTGAGCTTGCATTATTTGCTTATTAATATACATATTGTTGTATTTACATGTAAGTATGAACTTTGTGGCCAAGTATGAGTCTATTAAGCACTTCAAAGTTGGGTTTGTTGAGTGTACATGTTGAGAATCAATCAATTACAACAATATAAAACACAAAAGTTTGAATGGTTTTGGAAAATTTATGCAATGGCTTTTGGGTTAAGTTATTGAAACTTCTTTTAAGAGGGTAGATTGGGTTAGAACTATTGAGGTGTTTACACGGTTATTTATTTCTTTAATTTGCTTGATTTTTAGTTCTACAAGACCTTAAAACCATATAACTTGCTTTGCTTTGTTATTTATTTGCTTAGGAATTTATCATCTGGCTTTTTCTACATACCATAGGTTTTTGTTTATTCACCATATACCCATGAAAATTTTCAAATTCTGTATTCCCTAAGTTGTAATAAAAATTCACGTATATCCTTAATGAAATAAGATGAAAGAATTTGGTTAAATATACATCAGGAAATGCCTTAGATATCTCCCTTCATGCACCCAAACAGCTCTTACTCAAAACTATACAGCCACAGAAACCACTCCAGAATCTCATAACCATAACCATATAAATAATTTTGAGAGTTAGAAAATGAAATTTAGGGTAAGTTTGGGGTGCAAAAAGGAATGTGGTGGTTTCGAGGGTGAGGAATATGTTAAAGAAATGGTTTCAGTTCTGTAATTATCCCAAATGATTGTAGATTTAACAATAATATGTTTATCTAAGGAATTTATGGTGTATTTTGTGAAAACTCATGGGTACTCATAGTGAATTTAAAAAGTTCATGGGTATATATTGAATAAACAAAAAACTCAATGAGTAATTGAAATATCAAATTATAAATATGTATATTTGACATGATTCGGGAATTTATAATTTCATTAGAAAGAGCATGTAGAAAATGGGAATGCTTCTTACTATTTGCTAAATTTGCTTGATTTGGTTCTACAAGACCTTAAAACCTTATGGTGCTTTGTTTTGCAATTTATTTTGTGAGAAGTTATGAATACATATATTTGACATGATTCAAGCCTTTTATAGTTCATTAGAAAGAGCATGTAAAAAAATGGGAATGCTTACGTCCATTTTTGGCTGAATTTACTTAATTTTGGCTCTACAAGACCTTAAAGCCTTATGAAATGCTTTATCTTTGTGATTTATTTTTCTTAGAAAGTTGTAAATACATATATTTTACATGAATCAAGCCTTTTCAAGTTCATTGGAAAGAGCATGTAGAAAATGGGGCAGCTTCTTTATTTTGCCCGAATTTGCTTGATTACGGTTCTACCGGACCTTAAAACAATTATAACTTGCTTGGACTTAGTAATTGCTATGTAGTCTATTACAGATACATATATTGACATGATTCAAGCCTTTCTCAGTTCATTAGAAGAGCATGTAGAAAAAGAGAACGCGTCTTCTACATTTAGGCCGAATTTATGATTTTGGTTCTACAAGACCTTAAAACCTTAAAAATTGCTTTGTTTTTATTATTTCTTTTCTTAGAATTTATAAATACATATATTTTACATGAATCAAGCTTTCAAGTTAATTAGAAAGAGCATGTAGAAATTGGGGCAGCTTCTTTATTATGCCCGAATTTGCTTGATTTTGGTTCTACTAGACTAAAAACATTATACCTTGCTTTGTCTTAGTAGTTCTATTCTTAGGCGGTTACTAATACATATATTTGACATCATTCAAACGTTATAATTTCATTGAAGGAGCATGTAGAAAGCAGAACGCTTCTTTACATTTTGGCGAATTTGCTTGATTTTGGTTCTCTGACACCTTTAAACCTATAACATGCTTTTTGTGATTCTTTTCTTAGGAAGTTATAAATACATATGTAGACATGAATCAAACCTTTTCAAGTTCATTAGAAGAGTGTATTTGAAAGTGGAGCCTTCTCATTTGTAATTTGCTTGATTTCGGTTCTACGGACAAAACCTTATAACTTGCTTTTTTTTTTTGTGATTTCTGCAAGGAATTTACAAAAATATATTTGACATGACTCAAGCATTTATAAGTTTTTAGAAAGAGCATGTAGAAAATGGAGTACGCTTCTTACCATTTTCCGAAAATGCTTGATTTTGGTTCTCTGAAGACCTTAATACCTTATAACATCCTTTTTTTTTGTGATTCATTTTTATTACGAAGTTATAAATACATATGTTTGACATGATTCAAGCCTTTCATGTTCATTAGAAAGAGCATGTCGATGGGAATGCTTCTTCCCATGTTTATCAAAGGTTGCTTGATTAGGTTCTAGAACCATAAAACATATAACTTGCTTTTTTTTTTATTTCTATTCTTAGGAAGTTATAAATCCTTATATTTAACATGATTCACGCATTTATATGTTCATTAGAAAGTGTATGTAGAAAATGGGAATGCTTCTTGCTTTTTACCCGAATATGCTTGATTTTGGTACTACAAGACCTTAAAACCTTATAACATCCTTTTTTGTGATTTCGTTTTTAGGAAGTTATAAATACATATATTTGACATAAATCAAGCCTTTTCCAAGTTCATTAGAAGAGCATGTAGAAAATGGGAACGCTTCTTCTCATTTTGCTCGAATTCGGTTGATTTCGGTTCTACGGACAAAAACTGATAACGCTGTCTTAGTGATTTCTATGCTTGATTATAATACATATATGGCTTAGTTCAAGCCTTTATAAGTTCATTGAAAGAGCATGTAGAAAATAGGAGGCAGCTTCTTCCCAATTGGGCCAAATTTGGTTAATTTTGGTTCTACAAGACCTTAAACCTTATAAAATCCATTGTTTTGAGATTTATTTTGTTAGGAAGTTATAAATACATATATTTGACATGACTCAAGCCTTTTCAAGTTCATTAGTAAGTGCATGTAGAAAGTAGAGAAACCTTCTTCTCATTTTGCCTTAATTTATGATTTCGGTTCTACGAGACCTTAAAAACTTATAACTTGCTTTGTTTTGTGATTTCTATGCTTGAATTTACAAAAAATATATTTGACATGACTCAAGCATTTATAAGTTTTTAGAAAAACATATAGAAAATGGTACGCTTCTTAGCATTTTGCTCGAAAATGCTTGATTTTAGTTCTACAAGACCTTAAAACCTTATAACATCCCTTTTTTGTGATTCATTTTGTTATGAAGCTATTAATGCATATATTTGACATGACTCATGTAACACCCCAATAATTCTCTCTTTAAACCTCTGAGGACCACTGAACAATATTCAGAGAATTGCTAGCGTGCTACCATGCGTGTTCAAGCGATTAAAACTGTCGAGAAAATAACGGCGGAAAAACTAAATCGATTCTCATCAGTTTCAAAACAACAGAATGAAACCAAAATTAAGTCATACAACTTGGAACCATTAAGGCCCTTATTCAAAATGATAAAAGTAGTAAGTTATTAAATTCGGTATTGAAGAATAAATTCAAACCCAAAGAAATAATTAATAGAATAAAATGAATTAAAAATAAATTGAGCTGCAAAAGAAATAAGAATTAAACAGCCAACTCTCTCAAGATCCCACAACCGATGAATCTTTTTGCATGTGTAAGAACACATTGGTAGTGCATCTGATATTACAAGTGTAAAATTGGATCGATCACGAAGGGGTCATTGAAAGTGAAGGCCATGACCAAACAAAGGCACAACACACAATCGATAAGCGAGTACATACAAAATTTGCAAAATAAATTAAGAATTCCATAACATGCTACTAATAATCAATCATCATGCATCATAAATTATAGACTCGACCTACGACTACGACTATTTATATTACTAAGACTCCATTAATTAATAATAGGTAAATCATTATTTTTATTCATTGGATTACGCATAATAGACAGAGTTGTCAACCCATGCTCCATTTGGAACATCAAACAAAAAAACCCCATACCTCCTTTCGGAAACATCAATAAACATCCAATAGCGTGGGCGCGACTCGTGACCGTGGTTAGTATGAGATCTCACCTAACTCCTAGTTTTGAAAAATATTTGAAATAAGGGCTCGTGATCAGAATGACCTTGAGAAAAGTACAGCTAATACCCCATGTACCCCAACCCCTGATTGTCATTACTCCAGAAGTGCACCAATCCGAGTTTATTGCTCATCCATGCTGTGCTTTCATTGATTTACAGGTTTAGACTTGGTGATGACTCATATGCATAACAATACATTGATCGATATCAATTGATCCAGTAAAAGCAACTTTCCAATTTCAATTCTTGATTTATTAGTGGCAAGCAGCATTTATTATTTATTCCTTTACTGCACCGGTGTATAAACACCTTGAGTGCATAAGAGCACCCCAAACTCTGTAACCTTGTCTTATAAGACATGAACCAGGTAACTTTATTCATCCTATCTCATTAAATCATGAATGGAATAATAATTAAGTCATTTCTCAATCACATACCTTTGCCTCTAAAAGCGCACCTTGCATAATTAGAACATAACCAATAATTACAATAAGGAATAGTGCACGTCAAAGGAAATTCACAATGTATAACCAAACAATTTAACCCAGTCATCCTAGACATGATACTAGATTCTCCATCCGTCCTTAAACATGCACCCTAAAAGCAAGGTTTTGCTTAACTTATGCGTACCTTGAGCAAACGAATTAGTATGCCACTTTACTAACTAGAAAATCACCTTTAATAAACTCCTCTCTAAGCCAAATAATAAAATCTCAATCAATACCCAATCAATTAAATTTACAGCAATACTAACACTTTAAAACATTCAAAATCCAAATCAAACAACACTTTAAAACCTTCCTTTTGTTAAAATTAGAAACATATATAGTTCTTTAGAATTTTCTTGATAAAAACAACAAATACACTCTTATACATGAAATTACAGCAAGTTTAAAAGTCTAATAAAAATCTAAAAATTATGTAAAACCCCTAAAATCTCTCTTTTTTGTGGGTGATTAAACCCTCTTTTATGAAACATCAAAAGTAGTACTTAGAAACTATTTCCAACACTTGAAACTTCAATAAAAACATATATTTATGCTTAATTAACTTTAAACATAATTAATTCTATTAATATAACTCAAGGATAATATTTTAAGTATGTAAAAACTTTGTTAAAATGAAAAACTCTTGAAAATCATATTTAGAAATTTGAATTTGGATGTCGAAAATGGGAATGCTTCTTCCCATATTTAACCAATTGCTTGATTCGGTACTACAAGACCATAAAACCTTATAACTTGCTTTTTTATTTCTATTCTTAGGCGATTTATAAATCCATATATTTGACATGATTCAAGCATTTATAAGTTCATTAGAAATAGTATGTAGAAGAATGGGAATGCTCCTTACTATTTTTGCAGAATATCTTGATTTTAATTCACTACTAAGACCTTAAAACCTTATAACATCCTTTTTGTGATTTATTTTTTTAGGAAGTTATGAATACATATATTTGACATAAATCAAGCCTTTTCAAGTTCATTAGAAGAGCAAGTAGAAAATGGGAACAACTTCTTCTCATTTACTTGGTCGGCTTGATTTCGGGATTCTAGGTCCTTAAAACCTTATAACTTGCTTTGTCTTAGTGATTTCTATGCTAAGGGAGTTACTAATACATATATTATAATTCAAGCCTTTATAAGTTCATTCGAAAGAGCATGTAGAAAATGGGAACGCTTCTTCCCATTTGGGCCGAATTTGATTTGATTTTGGTTCTACAAGACCTTTAAACCTTATAAAAATCTATTGTATGATCACTCAAGCATTTATAAGTTTATTGAGAAAGAGCTTGTAGAAAATGGAGTACGCTTCTTACCATTTTTCCGAAAATGCTTGATTTTGGTTCTACAAGACCTTAAAACCTTATAACATCCTTTTTTTGTGATCATTTTGTTATGAAGCTGTTAATCCATATATTTGAAATGGCTCTCAGGCCTTTTCATGTTCATTAGAAGAGCATGTCGAAAATGGAATGCTTCTTCCCATATTTTCAGAATTTGCTTGATTCGGTACTACAAGACCATAAAACCTTATAAATTCTTTGTTTTTATTTCTATTGTAGGCAGTTATAAATCCATATATTTGACATGATTCAAACATTTATAAGTTCATTAGAAATAGTATGTAGAAAATGGGAATGCTTCTTACTATTTTGCCCGAATATGTAATTTTGGTACTACAAGACCTTAAAACCTTATAACATCCTTTTTGTGATTTATTTTTTTAGGAAGTTATGAATACATATATTTGACATAAATCAAGGCCTTTTCCAGTTCATTAGAAGAGCATGTAGAAGTAGACAACTTCTTCTCATTTTTGCAGAATTAAACGTTTTGTTCTGGACCTTAAAACCTTATAACTTGCTTTGTCTTAGTGATTTCTATGCTAGAGGAGTTACTAATACATATATGTAACATGATTCAAGCCTTTATAAGTTCATTAGAAAAGAGCATGTAGAAAATGGGACAGCTTCTTCCCATTTATAGGCCATTCAATTGATTTTGGTTCTACAAGACCTTTAAACATTATAAAATCCATTGTTTTTGTGATTTCTTTTTATGAAGTTATAAATACCTTATAGTGACATGACTCAAGCCTTTTCAAGTTCATTAGACAATACATGTTGAAAATAGACGCTTCTTCTCATTTTGCACAATTCTCTTGATTTCGGTTCTATCGGACCTTAAAACCTTATTAATTACTTTCTTTATTTATTTATTTTCTTAGGAAAATACAAATACATATATTCGACATGATTCAAGCCTTTATAAGACCATTAGAAGGAGCATGTAGAAAATAGGGCAGCTTCTTCCTATTTTGTGAATTTGCTTGATTTTGGTTTCTACAAGACCTTGAAGCTTTTATAACCTTAATACAGTTTTGTGATTTCTTTCTTAGGAATTTATAAATACATATAGTAGACATGAATCAAGCCTTTTCAAGTTCATTAGAAGCATTAATGAAAATAGAACGCTTCTTCTCGTATGATGTTAATTGGTAACAATAATTTTCATTTGATGACCTTAAATAGTTAAATGACTAAAAGAATGTCATTCTTCTAAGTGCTTTACTTAGTTGTCTGTAATTTTTGTATCATTTTATTTTTAGTGAAAGCGCTATATAATGAGGAGGATCGGGCATGGATGTATACGCAGTTAGAAGGAAAAATTCCTTAGTGAACCTTCTTTGAAAAGGTCAATGAATTTATTACATTTGCTACCACACAAGAATGTCGTGATAGATGGTGTGATAGAAATGTCCATGTGCCTGATGTCAAATATTCCTTGCTCAGAGATCTTGATACCATTATGGAGCATCTCTATAGGCATGGTTTTTGCCTAACTATTTCCAATGGAGTTTCATAGTGAGCACTTCCAAAGAGAATCCCAAGTAGCAGTTCCATATCCTTAGAGGATGCATTGAACCCGTATAGAAAACATGGTATTAGATGCCTTTGGACTAGAAGGTAGTTTAGAAAATATTGAAGAAGAGCCATGCTAGTTACAAGAGTTCTTTGAAGCATGTTGAAGGCAGCGGAAGAGCCATTGTATGATGGCTGCAAGTTATCTGCTTATTGTCTGCCCAAGTGCGAGGATGTGATATCAAGTCAAGTATAATATTAGCATAAAGCTATAGATGGTGTTGCTTCCATAATGAAAGATAGTTTTTACAAGATTAAGACATTGCTTAAGGGCTAGAACTTCCGCACCAAAGATTCATATGTCAAATGGATTTATGCTCTTTTTGGGAAGGAACACGAAGATCTCAAAGAATGTCTATATTACGAAAGGAAGTGGATTATAAGACACTTAGAAAGTAGCAGCAATTCTCTCCATAGTGCTCTTATTTACTTTCCTGTAGGCCCGAATTACAAAGATTATATGCAGCAAGATCTACTGCAGACGGAGTGGTCAGCATAAATACTCATAATCCTCGAGTTCATGGCCTTATGTCTGTCCAGTGATGAAGGCGTGGAAACACCTAGATGAAGAGTACCCTTCTTTCTTCCCAGACTGATGTCCCGACTAGGTCTTTGTACCGACCAGTTTTCACCCATTTGGAAAGACAGGAAGGCAATATTCTTGTTGGCACATCATTTTAACTCCTTATAATCTTCCACAGTGTGCATGAAGAAACCTTATATGTTTCTAAGTTTGATAATTCCCAGTCCGGAGAATCCTAAGGAATCTCGTGTGTACTTGCGACCTCTTATTGAAGAGTTAAAGCAATTATGGGAGGCTGGGTTACCAGGTGATAGCTTTGTATAATGTATTTGACTTTCATTTGACTAAATTAGCTTTGTTAATTACATTTAACTTTCATTTAATTGTGACTTTGTTAAATGTGTTTGACTTTTATTTGACTCGATTACCTTTGTTGAATGCATTTACTTTCATTTGATTCAAAAGCTTCGTTTGAATGCGTTTGAATTTCATTTGACTTCAATTAGCTTAGTTGAATCGTTTGACTTTCGTTTGATTCAAAAGCTTTGTATGAATATGTTTACTTTCATTAGACTCAATTATCTTTCTTAGGCCATTTGTCTCTTTCATTTGATTCAAAACCAATGTTTGAATGTTTGACTTTCATTTGACTCAGTGCTTTGTTAGATCATTTTACTTTCATTTAATCTAGCTTTGCTGATGTGTTTGACTTTCATTTTACTTAGTTAGCTTTGTTGAATGTATTTTGACTTTAGTTTGATTCAAAAGCATTGTTTAGATGTGTTTGGCTTTCATTTACTCAATTAGCTTTGTGATGCATTTGACATTCATTAACTCGATTAGCTTTGTATGAATGTGTTTGACATTCATTTATTTCGATTAAGCTTTGTTAATTGCACTTCTTTCATTTAATTTAAAGCTTTGTTTGAATGTTTCCTTTCATTTGATTGAAGAAGCTTTGTTTGAATGTGATTTGACTTTCATTTGACTCGATTACCTTTGTTGAATGCATTTGACTTTCATTTGATTAAAAAGTATTGTTTGAATGTATTTGACTTTCAATTATTTCAATTAAGCTTTGTTGAATGGTAGACTTTCATTTGATATAAAACCTTTGTTTGAATGTGTTTAATTTCATTTTGACTCAATTATTTTTATTGAATGCATTTAACTCTCATTTGATTCAAAAAGCTTTGTTTAAATGTGTTTGACTTTTATTGAGTGAAATGGTTTGTTGAATGCATTTGGCTTTCGACTGATTCAAAAACTTTGTTTGAATGTGTTTAACTTTCATTTAACTCAATTATCTTTGTTGAATGTATTTGACTTTCTCTTGATTCAAAAAAGCTTTTGTAAAAATATGATAATTTAGTTTGACTCAGAAGTGCTTTGTTAATTGCATTTCATTTTCTTTTAATTGAAAATGATTTGTTTGTTTGTGTTCTGTTTGTTGACTCAATTAACTTTGTTGAATGCATTTTACTCATTGGATTCAAAAAGCTTTTTAGAATGTGTTTGACTTTCATTTGACAATTAAGCTTTGTTGAATGCATTTGACTTTCATTTGATTAGCTTTGTTAGAGTGTTTGACTTTCATTTTGACTCAATTATCTTTGTTGAATGCGTTTGACTTTCATTTAATTCAAAAAGCTTTGTATGAATGTGTTTGACTTTCATTAAGAGTCGATTATCTTCTATTGAATCTGGATTCGACTTTCATTTGATTGAAAGCTTTGTTTGAATACGTTTGACTTTCATTTGACTCAATTATCTTTATTTAAATGCATTTGACTTTCTTTTGATTTAAAAGCTTTGTTTGAATGTGTTTGACTTTAATTTGACTTAATTATCTTTGTTGAATGCATTTGATTTTCAAAAGCTTTGTTTAAAAGGTGTTTGACTTTCATTTAAACTCAATTATCTTTATTGTATGCATTTGACTTTCATTAGATCCTACAAACTTTGTATGAATGTGTTTGAGCTTTCCTTTGACTTAAATATCTTTGTTGAATGCATTTGACTTTCATTTGATTGAAAAGCTTTGTTTGAATGTGTTTGAATTTCTTTTCGTTGATTATCTTTGTTGATGTATTTGACTTTCATTAGATTCAAAAAGCTTTTGTTTGAATATGTTTAACTTTCATTTGAGTTAAAAGCTTTGTTGAATGGAAATTGACTTTAATTTGATTAGCTCACTAAAATGTTTGATTTTCATTTGAGTCAATTATCTTTATTGAATCATTTGACTTTCATTTAATTCCAAAAGCTTTGTTTGAATATGCTAATTTTCATTTGAATTAGCTTTGTTGAATGCATTTTAACTTTCATTTGACTCAACTTTGTTTTAATGTGTTTGACTTTCAGTTTTTCACTCGGCTATCTTTTTTGAATGCGAGTTGACTTTCATTTGATTCAAAGAATCTTTGTTTTGAGTGGGAAATGAAAGTCAAACACACAGTGGCACAAAGCTTTGAGTCAAATGAAAGAGGGTTAAATGCATTCAACAAAAGCTTGTTTCAGTGAATTAAACACCTCATTGGAAGCAAACTTTAATTAAATGAGGGGTGTTGTGTTATTATTGGGAGGAGAGCCAGATGTTGGCATTTATTGGACCTTCTAAGTGTTGTCAAATGAAATCGGCTAAGCTTTTGAAATCAGTAAAAGTCGATTACATTGACAAAGCACAGTTAAACCCATGTCGAGCAAGCTTTGAATCTAATGAAGTCGAGCCGACCAAACAGACAATTGAGCAGAAAGAATCAGAGCATTCAAACAAAGCTTTTAATCAAATGAAAGTCAATGCGTCAACAGACTATTTAAGAGATCGAAAGAAAGTGGCGCCACATTCATCACAGACTGTGAGATCTAATGAAAGTCAGCTCTCTGACTGTAATTGGCAGAAAAAAGTGGCACCTTTAAGCAAGCTTTTGAAAATCAAATGCATTAACAATAATGGTGAAAGTGATCAAACACATTCAAATAAGCTTTCAAATCGAAGAAAGTCGAATGCATTGAAAATGGTAGGTGGTAAGTGTGTGGCAAGCTTTGAATCAGTGAAGTCGGCTACCGACCGATAAAGATAATTACGACGTGAGAAAGTCGGCTACATTAAATCTGGCACGGGTGAAATGAGAAGTCAGCGCGTTGACAGTGGTGAGAGTGTGGCGACAGCTTTTGAGATCAAGTGAAGTGGTACGTGACAGAAGGCTGGTGAATCAGTAAAGTCGAAACACGATACAAAGCTTTAGTCAAATGAAGTAAATGCGTTCGGCAAGTGAGTAATGAAATAACGTTAAATCGATCCAGCAAATGGATACAGGTAAAAGAAAATGAAAATCACGATAGCAATGTGGTAGTTTATCATATTTTACAAGCTTTTGAATCGAAGAGAAAGTCCAAGCCCATTCAACAAGACAGAAGTTAAACACGACCAAACAAGCTTTGAATCGAGAAGTCGAGTCATTCGAAAGAACCATTTTGACTTTCAGTGAAGTCGAGTACGTTCAAACAGGCTTTTGAAGCCAGTGAGTTAGGCATTGATAAAGAAATGGTGAGTCAATGAGAGTGCGTAACAGCGTTGCAGTGAAGTCTGTGCGTCGACAGAAGCAATTGGTCGAGCACGACCAGCGATACTTTGTCAGTCAATCATTGACAAGTAGTGGTCGGTAGTCGACATTGATAAAGCTTCTTGATGGAAATGGTGAAACGACCAGCAAAACTTTTTGGTGGAAATAGTGGTGCAACAAAAACTGGTGAACCGGTGAGATCGAACACATTCATCTGGTAATTGGTAGATGATGTCGAGTGCGGTCTGCCCAGTGAGTAAGAATGAAGAGCGAGCACGGCCAGTGATCTTTTGAATGGTAAGTGTACATTCAGCAAACTGTAAGTAAATGAAGTCGAAACACGTGGCTTTGTCAGTAATTGGAAATGCGTTGAGGCTAGTGAAGTCGGTAGTCGGCATGTGCAGTTTTGAATCGGTGGTGCATTGAAAGACTGGTGAGTCTGTGGTAAGAACATGTTCATCAAAGCACGATGGTCGGCGCCCGACAAACTAGTGAGTCAGTGAGCCAGACGCGGTGATCAACGAAGCTTTGAAACATCGGAAATGGTAAATCATTGACAAGGCAGTGGTGGCAAAAGTCGGCACATTTAACAAAGCTTTGTTGAATTAAATGGTTAATGTAATTAACAAAGCTAATTAGTCAAATGAAGTCAAACACATTATAAAAACTATTAATCGGTGGCTTGACCTCCCATAATTGCAACTGCGATGGAGGTCGCCAGTACGTCAGTTCCCTTTAGATTCTCGGACTGTAGGCTTAGAAACATATAAAGGTTTCTTCATCTGCTGACCAGTGAAGATTATAAGGAGTTGGAAATGACGGTGTGGCTGAAATGTGCGCTCTGTCTTTCCAAATGGTGAAGAGCGTCGTGAACCTGGTGCAAAGAAAGGACCTCTGGAGTGGACTCACGCATCTTCACTAGATGGACATGGGCAGTGGTTTCCGGATATGTTGTGCCGCCTCATGCTCGCAGAAGATCTTGTTATAATCTTTTGTAATCTCGAACTCTGAGAAATAAATAGAGCACTATGGAAAATTGCCGCACTACAAGTGTCTTATAACGACTCTTTGCGATAGACATTCTTTGGATCTTTGTAATTCGCTCGAAATGTATAAATCCGATTTGAACATACATCAGTCTTTGGTCTTGATTCAACCTTAGCGATATTAATGTAAAGTATCTTTCATTATGGAAACGGCACCAATCTATAGCCCATGCGGGGTATACCGGACTTGATGTTGCCATCCTTGCGTGCGGAAACTGTACGGTGACTGGAAGCCGGAAGTCGCCACAATGGCTCTTCGGCGCCTTCGACATGTGCGAAAAAACTCTTGCTGTGCGTGTGGCTGTTTTCACAAACCACCTTCTGAGTCGAGCATCTGGCTCACCATTTCTATGTAGATTCGATGCATCCTGGGACCTGAAGGCGCTGCTAGGATACTGAATGCGGTTTAAACCATTGAATCCAGGCAAAACATCTTTCAGAATATGCATGAATATTTACCGACGCTGTGCGCATGAACATTTCATCGCACCATGCCGCGACATTCTGTGTGTGGCAAATGTAATAAATTCATTGACGCACCGGCGCGAAGAACCGACGAAACACCGCTACAGCTGTTGCTACATCACGTATCACTCCTGCATTATATAGCGATTTCACTAAAAATAAATGATACAAATGAAAATAGCGCAGAAAGAATGACATTCTTTAGATAAATTTAACGGTCATCAAAATATTATTGTTACGATGTTTATCGTATGAGAAAGAAGCGTTCGACCTACACTGCATTGTTTAATGAACGAAAGGCGGTGTCTAATATATGTGTTATAATTCCTAAGAAAGAATCACAAAGCGCGTGTGCAGATCTTAGCCAGAACAAAAAATCGGCTAAATCGACCAAATAGGAAGAAGCGTTTATTTTCTACGTAGCTCACTGCAATGAACATAAGGCAGATGTATTAGAAGACCTATGTATTTGTATTTTCTGAAATGAAATAACGAAACAGTAATAGTTTGATCTGATAAACGAAATCAACAGTCGGGGAAAATGAGAAGGCGTTCTCATTACCAGCATGTGCATTCTGAAGCGTGTCAATGTATTTATAACTTCATAGAAAAGAAATCACAAAAAACAATAGTTGCAAATGTTTAAGATCTTTGTAGGGCAAAATCAACGGTGGCCCCAGTGGAAAATTCCATTTTACGTAACTTTGTGAACATAAAGGCTTGAATCATGTTACATGTGTGCTCTCTTGGCATAGAAATCGCAAGACAAAGCAGTTATAATTTTAGGTGACAGAGGCAAAAATCAAGCGGTCGGCTGAAAATGAAGAAGAGCGTTCCGATTTTCTATGCTGATGAACTTGAAGACGTTATGTCGATATATATCAATTCGCCAACTTCTAAATGAAATCTGTGACCTAGCCAGACATGTACCAAGAGTAAGCATATTCAGAGGCGTAGTGAAACGATCCATTTTCTGCATGCCCATTTCTGTGAACATGAAATGCTTGAATCATCAATCGAAGCCTGTATGGTTGCTGGCGCTACAGAACAAGTAAAAGGCAATTTATAAAGGTTTTATGGTGTATGTGCCAGTCAAGCTGAAGTCGGCAACATCGGAGAAGCATTCCCATTTCGACATGCGTGAAAGACCTGGTCATTCCAGATAACTGTAGTTAACAGCACATACAAAAATGATGCAAAAAGAAAGGATGTTATAAGGATTTAGACTTTGTGAGACAATCAGCATTTGGAAAAGACGATAACCCCTTTCTGGCTCTTTGGCATAAACATAAATGCTTGGTCGTACAATAGATTTTATAGTTTAAAGATCTTGTAGGGCAAATCAACAGAATAGGCCAATGGAAGAGCGTTCGTAATAACCGGTGAGAACATAAAGGCAGATGTTACATATATGTATTAGTAACTCCTTTGTAAGTCACTAAGACGAAGCAGTTGGGGCTGAAGGTGGCCGAGGCCAGTGTTCCGACGCACGCTCTCTGTGAACTTGAAAGGCTTCGTTCTGTGTAAATATATCTTCTAAAAAATAAATCCTGGATGTTATAAGGTTTAGATGCTAATGTACCAAAAATCAGCATATTTCCGGCGGAAATGGCAAATTCCCATTTTCTACATGCTATTTCTAATGAACTTATAAATCTTGGAGAATCATGTCAGCTATGGTTATAACGCTAGAAATAAAAAGAAACGAAGTTATAGTTTTTATGTGTATGCCGGTGAAACCGGCTTAACATGAAACCCATTTTAAACACCGAATGACCTCCGGCGGTCATTTTGCTGAAATTTACATATAAATGTATCGCAGGACCCATATTAACAAGTAGTATGTTTAAAAGTTAATTAACATAAATATATGTTTTTATTGAAGTTCAGTATTGAAACTAAAAATACTTACTGATGTTTATAAAAAGAAGGTTTAATCACCCAGTTAAAGAATAGGAGGCATTTATATGGCCACGGAATTTTTATTGAACTGCTGCTGTATTTCATGTATAAATGTATTTGGAGTTTTTATCGAAGAAAGAACAGAGGCTTATATATGAAGCCTTTAACAAAGGAAGGTACAAGTGTTTGATTTGATTTTGAAGTTTAAGTAAGTTAATATTCTTCGTAAATTTGGTGATTAGCGACGATGAGACTGTATTGTGAAGAGTTTATGGTGATTTCTAGTTGGTAAAAGTGAGCATACCAGTGCGCAGCTCAGGTATTACCTGAGGCACTTGCTGGAAGGTGCGTGTTGAGGCGATGGCGACAACCATGTCCCAGGCTGGGCTGGTCATACATTGTAATTTCACAGTGCTGTCACTTATTGTAATTATTGGTATATTCCTGATGCAGTCGCTTAGAAAGTGAGTGATTGGTGAATGACAATATTATTCATTCATGAATCAGAGATGAGCAAAGAGATGGTTGTATCTTGCAAGGCGAAGGTTAAGGATTTGGGGTGCTTATATGCCTTCAGGTATTTATGCAGAACAATGAGATAAGTAATAAATGTTGCCAGTAAATCGAGGAGTAGTGGTGCTACTTGGCGGTGATGTTAGTTGACAATGACCCGTTATGCATATGAGCGGGGGTCTGGAAACACGCCGTCGCGTGAAGGCTGAGATGGTGGCGACAAAGCTGCAGTTGTTACGGAGGCAATGACTGTCGGGTTGAACACATGAGACATTAGCCGTGTACTTTGCCGGCGTCTGTCACCAGTCTTATTTGATATTTTTCAAGCAGGGTGGAGGTGGATCTCGTACCTTTGCGGTCGCGGTCGCTCCGTCGTTAGAGTGTTTATTGATGTTTAGAAAAGAGAAAGTTTTTCTTTTATTATTGTTCCAGAAAAGGCATGGTGGTTTTAACGCTGACTCACAACTAACGTATCAGTGGTGGCAGATTTACTATTATTGGTAATGGGTGCTAATATAAACAGATTGATCGTGGTACATTATGATGCATGATATTGATGACCCGAAGCAGCATAGGTGCTTAATTTATTTTGCGTACTGCTACCGACGGCGTGTATTTGGTCGTGACGCCGCCATGTTGTGGCGATCAGCCCGCTGTAATGTGAGGCGAAGTACCGCAGATTCTTGCATCTGTATTCATGAGGTTGTGAGATCGCGAGTAGAGGTTGGTTGTTTAATTTTGGCACTGCTACGGTTTATTCTTCCGACCGATTTTATTCGACACTATTTCTTTGGGTTTTGAGCTATTTAGCTGCGAATTTAATAACACTACTTTGTGGGTTTTGAATAAGGGCATAATGGTTAAGTTGACTTAATTTGGTTTCATTCGTTGGTTTGAAACTTATTTATAAGCCGATTTAATTTTATTTGCTGTTTTTACGATAAAGGCCTTAACGTTTGGGCTGCGTGGCGGTAGCACGCCAGTAATTCTCTTGGGTATTGTTCGAGGGCTCAGAGGATTTAAAGGAGAACTTAAGTGGGTCATGTCTAATATGCATTAATAACTCATAACAAAATGAATAAAGAATTATAAGGTTTAGTCTTTGTAGAACCAAAATCCACCAGCCTTAAAATGTAGAGAAGCGTACCCATTTTATATGCTGCTACAAAACTTATAAATGGCTGATCATGTCAACCTCTATTTTTTTTGTAAATTCCCTAAACATAAATCACAAAAACAAAAGCAAGTACTTTGGGTGGTAGAGGCCGAAATCAAGCTGAAACAGACGAAAATGAGGAAGAAGGTTCTCGACTACTACATGCACCTGTGAACTTACGAAGGCTTGGTCATGTCAGATATATGTGATGATACAACAAATAAATCTCAAAAGCGATGAACACCTGCTAGTGTTGTAGAACAAGGTGGCAAATTTAATGAAGGCGTTCCATGCTCTTTCAACGAACTTATAAAGGCGAGATCATGTTATATGTGTATTAGTAACTCTCACAAGCATGAAATCACTAAGACAAACAGTTATCGACCGGTAGAAAACCGAAATCAGAGAACCGACAAAATGAGAAGCGTTGCTCTCTGGCAGACGAAAGGCGATTTATGTCAGGCTCTCATTTCTTTCCTAGCGAAATACAAAAAGGATGTTATAAAGGACTTAGATCTTTGTGGCTGCAAATCAATGCATGTGAGTAAGAGAATGACGATCCCATTTTCCATGCACTCGCTGAACATACTAAACAAACCAGATCATGTTAAATATGGTTATAACTTCCACAGAATAGAAATAAAAAGAAAGAGTTATAAGGTTTTGTGGTGGCTGGCAGAAGACGGCTGAGCTTTCGACGACATGGTATTCCGTTTATGCTCTTTCACAATGGCATGAAAAGGAAAAAATCATCTCAAACATGTGTTATAACTGATGATAAAATAGTCACAAAAAAAAGGATGTTATAAGGTATTAGATCTTGTAGGAGCAAAATCAGCGACTAGGAAAAAGGCGATACCATTTTCTGCTAACAAAGGCACAAAATGCTTGATCATGTCAAATATATTTTTATCTAAGCATAAATCACAAAAAAAAGCAGTATAAGGTTGCAGGCTGCAGTGAGCAGAAAAATCAGCAGTAGGCAAAAGTGAAGCGTTGACCTCAACTCATCTTCTAACGGATGGGCTGATTCATGTCTAATATATGTATTTATAACTTCCTAGAAAAGAATCACAAAAGAATGATATCAGGTTTAAAGTGGTGTAGAACCAAAATCAGCAGTCGGCAGCTGGAAGAAGCTGGTTCTGGTTTCTGCATGCTCCCTTCTAATGGTATAGCGACTGAATGATGTCAGGCTGAGTGGCTTTGCTAAGAATGAAGTCACTGAACAAAAGCAGGCAATATTTTGGGTCTAGTAGGCAGAAATCAAGCAGGAACTGAGTTGACATGCTCTTCAACGAAAGGAAGCAGGTCCATGTAAAATATATGTATTTATAAATTTACAAGAAAAGAAGTAATAAAACTTGCAATTTTGGGTTTAGATGTGTAGAACTTGGAATCAGCTAAAGTCATTCTGCGCAGCGTTCCATACTACATTAGCAACAGCAATGAGCGGAGCGGTCATGTCAGATGGCTGTCATGACAGGCTAAGCGCAGAGAACCTTGGGTCAACAGTACGGTGCAGGCTGGCAGACCGTGTCCAGCAGAGTGACAAAATGAGGAAGAAGCGGTTCCCCATTTTCATTAGCTAACCAATGAACGAAGGCTCATGAAATATATGGCCATTTACAACTTTCTAGAAAATGAAATCGCAAAGATAAAAACGTTTCATGAAAGACTTTAGGTAGGCAGAGTGCTAGAGTCGACGAGAATGAGGATGAGCTGGCCCATTTTTACGTATCTTTCAGAGCTTGGTCATGTCAAATATGTGTTCATAACTCTCTAGTAAACCCAAAAACAAAGCAGTCGTAATTTAGATGCTAGTAAGCAAACCGATGGTAAGCTTCCCATTTTCTACATGCTTCTTTCTATTAATTGAAATTCTGAATCTGTCAAACTCTACATATTTATAACCGGATATTTCACCATATTCCGTGGTTTTGTTTATTCATCAGCTATGCCTTGAGCTTTTGGAAATTCATCATGCTATGAGTTTTCTGAAAATACACCATACTAACTGATAACATATTGTGTTAAGTCGTCGATCGACTGAGGATGAGCCGAAACCACTTTAAGCTCATTCGCACCGTACCACGTTCTTTTACGCAAACACCTAGAACTAAACACAATCTAAAAGTGATTTATGGTATGGGGCTGAGTATTTCCCAGGGCTAAACGAGGAAAATTGTTTAGACGTGGTAATATGTTCGATGTAACAAATTCTTTCATCTTATTTTCATTAACGACAATTTTATTACTGACAGAGGGAATATGGTAATTTGAGGTAGTATATGTTGGTCAAACAAAGCTTTGGTGTGTGATGGCGTCGACATAGTTCTGGAAATGGCTGAAAGCGAAAGCTTGAGTTATGAATGGCTGAACGAAATCAGTGGGCGAAATGGAAGAAGCGTCGTAACACCCGATAGTTGTGATACACCTGCAAAGAAGTTTGCGTACCGAAAACAACCCAGCGCCGACCTGCTTTTTCGAAACCATTATTTTGTGTTTATATTGTTGTGAAATTGGGGTGATTCTCACATGCACACTCGTGTACTATCATGGCAGACTCGTACTGGCGAAAGTTACATGCCCATTACGTAGCAAGCTGCAACGGTAGTGCATGTTAATAAGCGAGAAATAATATGCGGCGCTGAAGAATAAATGGCATGAATTGAATGGCATGAAGCTATCGCATTCTACCTGCAAAGAAGAGTTGCACCCGTAACCGTCGCGATAATATCATTAGGCATTCTTTCATTTCTTACTGCTGTGTTATCCGCTTGTTATTCTGTAATTTCACCAATTTGAATAGCTGAATCATGGGCGCTTCGATGTCTTTAAAACAAAGCGCTGGCAGCCAAAAACTGCAAAAGGCTGTAAAAAAATCTTGCGGGTAACGACGAATCTCGATAGCGAAGCAGCCATAATGTCTCAGATAAATGACGGGAACCGTAAGGCCCCGATACGACAGTAATATCATTTTTCCATTTGTTGCAGCCATCCATTAGTAAAAAACCTGACGCCTTCTAAACAGTCTTGACGATTAAATGTTCTTTAAGGATTTAAAGGCTTGTTGAAATAACTCTTTCATCGGCTATATCTGACGATCTCAAAGAAATCGCCACGACCTCTTACAAACATCTAATCTGATAAACTCGCTTATATCGGTGCATTGTTAGGTGTGAACCGCCCACTCACCATATTTTGATCTGAAATGAGCTTACTTTCGTGTAAAGGTCGCGCTGGCTGACCTGGAAGCGTCTAAAATGTCGCGATCACCAGTGATATTATAATTCTCCTCGCTCTTCAAAAGTATAACAAGACTATCGTTAACAAACCGTATATATCAGTGCTAACTACTATCAAAAATATTCGATTCGTAAATCCATGTGGTGTTAGTATTAACCGGTACGATTTTATGACCACCATGTTCGTGGACTCCACCGAAATTTTCTTCTCTCAGCAGTTTTTCATTCTCACCAGCAGTGTATCCGCATTCTCTTTCCGCAGTATGCTCTCAATTCTCGTGAAAACAACGAAACTCTTCAGCGCCCGGTCTCAAAAATAACTCTCGCAAAGTATCAGCGACGACAAAGCAGAATTAAATTACGCTCACCGTAGTTGAACTCTATATTCTCAATTCTCAGAATGCGGTCCTATTTGTCATGGAACCGAATAAACTCGTGGTGCAAGTGAAGGAGATATTAAATAAAAATATGAATCTTACGATAAGCTAAACACCAAATAAATGATAAATAACATCATTAAATAAGCATAACCAAGCTAGTCATATTATGCTATATAATGATCATCCATATCGCGATACATGCGCAATCACCAAGTAATCTGCTGTGTATTCTTTAATCTTGCATGATTCTAATTGATCTAACACCAAATTGTGATTATATACGCAAATACCAACTGCATAAAGCTACATTGAAGCAAATGGCTGCCAGTAAATTATGTGCACATGAAAGAAATCATAAATCTATGCCGAACTACCCAGCATCACACTTAAAAAACAAAATAATAATCTTGGGAATCAAGTAAAAACAAATGCCAGCAATTAATTTCAAAAATAGTGTATAAGTAAACTAAAGAAATACATGTATTTTATTTAGCTAGAACCCAGTGAAATAATTTTTGAGTAAAGTTAACCTTAAATCACGTCATAAATCACAGTCCATAGATTTAAAGCATTAGGCGCAAACTTTAAGCTGGCTTTAGTGACAATTAAACAGATACCCAAACCGGTACCGCTGGCACTGATAATTCTGCTCCTCGGTCCACAGCGGCTGTCGAAGTGCGGCGTTAACTTCACCGCCAGCGTGTTTCAGGCTTTGTCAGAAAATAGCGATGACGTAAAATAAGTTTCAAAAGCAACGAATCAGGCCAAGTCGCTGAACCGTGGCATATTGGAAACCCGATAAATAGTGATTAAAATGGAGTCTCAAATGAGGCGACGACGGGAAAGATAAGTATTACTTCTGGCAATAGAACGTGAAACCTTTGGCCAACCGCTGTATGAATAGAGGTGAGTTAGGATCGTTGAGAGTGCGGTAAGAGTCAGTCAGCTACTGAGGGCGTGAGATGGACTGATTGTCTTAAATGGTAAATCAGATGAGCACTTCAGGGGAGATGGCATGCTGCTTGTGTGTGCTGGGTAAAACTCGTTGTTGATGATTGCGATGAACAAGCAACGGTAGCGATCAGAAGGTTCGGTGCTATGCTCTGTAATTGTCTGGTGATCCTTGAAGGCTCTTGGAAGGTAAATGAACAGGCTTCCCGATCGATAAGCGACTTTGGTCGATGTCATGTCATTAAGGATTGCCGATGCGATCTGGGTGTCTTATGAGGTGATCAATCTGGTCTAATCTCTATCTGTGCAAAACCAGTGACAGCAAATCCGGTAATCGGAAGCCCGGCTTGGCATTCCGTGAATCAGCTGTCTTCCCGCTCTGTGACACTCACTTAATCGTGAGTTCAGGCAAGCAACTAAATTGCATTGATCGGCATTCACAAATTGTCAAAGAAATCGAGTGACCTGTATCTGTTGAGCTTCAGAAAATACGAGCATATCCAACAAAAGGCAGTAGTAGTAGTGAAGAAGAAGATCGCAAATCAAACTGATTGATATGTTGATGCAATCCCAAGCTAAAAAAAAGTATACTTATCTTCTCTCACAATTTCTCTCTCACAGAGCCAAGGCGGATAAGTATCTAAGATTAGTTTTTAAAAATGCATTGTTATAAACCAGAAGAAAATCCTCCGTACAGATCTGAGTCTCGCGGCTGATACGGCGCCAGCGGCTGTGTGAGTAGATGACCGCGCCCTTGGTAAATCCCACGCTCTGTGAGTGGAGAGGCCTCTCCACGGCAGCCGTGGCCCATGAGGGCCAACCGTGGTAGGCTGCGACTTTGGCAGTTTACAATCATCATAAGCAGCGCCGGCGGCCTGGCAGCCCCTGTGTCGCGGAGACTCTCAGAGGAACCTCCCGAAGGCTCTCGCCCATGCAGCTCACTGCTTGATCGTTATCTGGTATCCTTTACTGGGTGAAATACGGCCTTGAGCAAAGAAGGAAATAAAGGAAAGTGCAAAAATAAAGGAAAACATGAAAACTGCGTGAAAGCGAGAAGAAAGAAGAAGAATCAAAGGCTAAGAAGTAACGAAAAGGGAAAGAAAAGACAAACACCCTATGAGTGCCATAGTGACTTGCTCGTATTCCCCAGCTTTATAGTCCCCGTGCAAAACAAAAACGAAGAGAAAAGTGGGAGACGAGGAGGGCTAGAGGCTGGCACTAGGACGTGGGGTGCAGGTTGTTCCCAGCTCTGGCTTGGCCATTTAAACCCCGAATGGCTCGGGAAGGCAGTGATGAGGTCGAGGCGCCATGGCTTCTCCGCAGTAGAAGCAGGCTGAGAAAGGAAGATAAAATCTTTGTAATGGACTTCTGAAGGCCCCTTAGCACTGGAGTGAATGGCATCCTTTCGCGAATATGTCTGCGTTAATGGCTGTAAATATGCTAAGCATGAATTTAGTTATCACACTCACCTTCTGCCGCCGTTCCCCAATATCTGTGTGGAGCGTAGACAAGCGAAGAGCAGAAAAAATCGAAGGATACAGGCTACTAGGCTGTCTTGTTCGCATCCTTTACAGTCCACAACTATGATTCCTCCGGAATGAAAGACTCAACTCGGGTCTTTCCCCAATTATAACCTTAGGTGAAATTTAAAAATTACGGAATTTTGGTGGTTGTCTGCGTAATGGTAAAAGCGTCGAATCTACGACACGAGCAAGTAATCGCGGCAAATGAGAATAACGCCCCCAAGTATTGACTATGCTACACGAACCAGTGTCTGACTAGAAACGTCTCAGAGAAAATTTATCGTATTTTTTTCGGCCCTTTTTTTCATTTTAAACTAGATAATCGCTGCGCTTTGATAAAATCGACGACTTTTTGTCGCGGTGTCAGTCGTGCCATGGTGGGCGGCTTTGTAACATTTGCCATTGTTCACGCGAGATATGGGGCTTTGCGCTATCAGCGAAGCCCAGCCAATATACCACAAACCATCCGAGTGATCAGGTTATACTAAAGGCCAGAAAATGAGAAGCGAAAGACTGATATCTACGAAGATGATAGCTCAGCAAAGGCAATCAAGCCAGCTGGGTGAGCATCCAAAGTAGTACCGCCACGACAGCAGCTTAAAAATAAAAAAGGAGTTCGATGTGATCTCATAGACTACAGATTGGGTTCAGCGAAAATCGAAAGGATCCATCTTAAATCTGGAAACAGATGAAAACTGAATGAGCACTTCAGCGTGGTGAAAACTCGCTAAAGACCGTCAAATCGAGCAACTACGGTGGATGAACGTCCTGCTGGCGTAACATCGGCAAGCGTCAAACCCGTCTTGTATAATTACCTGGAAGACTATTGAAGCGAAAGAGCCTCAGATGTTCACTGGCAAGAATAAAGAAAGAAAGAAGAGAAAGGAAAACAGGCGAAGGATGAAAAGAGAAGACGAAGATCAAGCAATTTATCGATCGACACCAGTGATACAATGACCACATCTTTAGCAGGTGTTATCATAAATCTACGACGTTCGAAACTTTCACGGGAATCTAATCTAAATCTGGCAACAGAAAATAAAGCGGCGCAAGGCGAAAAGCCAGAAGATACTCTGGCGATAAGATCTTAGACAAAGGTACTTGAATGAGGCTCATATTTGGCATGTGCAGCAGATGACAAAGAGAAAGAGAAATATATGAAATTGTGACCAGCGGCACTTCCTGTGAAACAAAAATAAGCGAAAACGCAACTATATCTGAAGCCTAAAACATGCGTCATCAAACTGTATGAAAAGGCCAGTATCATCTAAGAAATAATCGACAACAAAAACGACTGGACCAGATAACGAGATCACGAAAACCTTATCAGACTTCCGCGAGTCAGAATCCCCGTGCTAACGTCCTTTGTCTCCCTCACAAAATGGCATGGGCCATGCCTCATGACCGGCTGCAAAATAGGGAAGAGGGAAGGCAAAGGAAACAAAGAATGCAAAATGCTGTAACCCACTGGACCGACACCATCATCAGCTCACTAGGCTCCATGGCCGTCCCGACCTGAGCCGTGGCGTATAACTACTTTGAATAGGTCTTGTTGGGACAAGTCAGTGGTGAAGGTGATGGTGTAATAATGTCATTACTCAATAATGACTGGGGAATCGCCCTGCCGCTCCCCACAGAGACATGAGTCGTAAGGCGGAAGTGGGTTATCAACCCCTTTGCCGATGAAGTGCGTGGGGAGGGCCATCGCACCCAGTGAAAGACATAGGGCGACTTTATGGAAATGGTTGAAGCCGGGTGTGATCTCCCCCTGCGTTAGCATGTATAAGCGATTATTGAAAGCGATCGCGGAGAGTCTCGTCTTGTCCATTGCCCGCCATATCTTGCCGATCGATGTTGTCGTGCCAACAACTCTCCTTCGTGTTGTTGTTGTTGTTGGTTGTTGTATTGTTGTTCCTGCGTCATCGTCTCTCCCATCTTCCTGTCGCCTGCATCTCCAGCCATTCAGCAGACCTCCTCCATCGTCACCGTCAACCACCAGGAACTGGCATCAGCGTTCCCAGCAAGATGGAGGTGCGAGAAGGCAATGATGGAGTGTTCACAAGCAGTAGGTCCGGCCCTCTTGATGCTGATGGTGGAGGGTGTTGCTTTGTGTAGGCCCATCGATCTTCGGCTCCCGATCGAGTCTTGAAGAACCTGGCGGATGTCTTGCCTGTACTCTGTGATGAACTGGCGATTTCACCCGTGCATGATGCTACGCCACTCGCGGCGCGAAGTTATTACAAGCGATTACGGCACCACGTATCCGCGAACCGTGATCTGCTGCCAGGGCGACTTGCAAGTCTTGCTGCAAGCCGCGGTGCCCAGGGGAATTACATGGAGGATAAAGCGTGATGAATGTCTCATAGCAGAGGAAGTTTTAGTCAAGCGTAGAACTATCACGGTACCTGCTCAACACGAACAGGGTGATAAGAAGGTTCGAGAAAATATCTCGCCATCGTTAAGCAGCTGGCTATTGAACACCGGCGTCGTGTTTAATGTTGAAAGTTGAAGAATGATGTAATCGTGTCCCCAGCCCTATTGAGCAGAAGATAGCTCAGAGACCGGTTGCCAATCACTGGCACTGGGTTGTTTAAAGAACCTCCTCGATCACAGCGCAGCTCGTGCAGAGGAAATAATTAATGGGGAGTGATCTGGAAGCCTACGATCTTTCATCTTGAAATGGAGATTGTTTAGGCGTCCCTGAACGGGTAGAGGAGGAGGCGTCATCTTGGTTAGAAAGGAAGTGTTGTGGGTTAAGGATGGCCTGGGCGAGGAAGACAGGTGGGCTGACTTTGCTACTCTTTTGAGTCTCAGTAGTGGTTGTTTTGAAAACAAACTTACGCCAAATCGAAAAAATATTGACCTGATCGGTAACAGTACTTCCAACTCACGCAATCTCGCTTCCGTCAACCAAAACGGAGTACTGCAATCACACCCGCGACAAATTCCAACGGCTAAAAATGATATCAACGATGTCCATGCTAATATTCAGTTTTCGAAAACAAGCATCACCGAAATTCAAATTTCACAACGATGATCATGAAATACAAAATCATACTCACAATCAGATATTACTAAGCGATGAAATCAAGTAAAGTATTAAGTAAACACGAAGGTTAAGTATGCCCTGGTACTCGAAAAACGCCCTGCAAAAGTGCGTATGGTAGAAATGATGAAAGGAAGAAATGATGGTGGTAATCAGAAAATTAATTAAGTAAGTGACAATTGAGGCAGAGTTTGAAGTTTGTGAAGAGAAAGTAAAGTGTGGAAAACAGGCGTGATTTAACTTGATTACCAGTAATACGTAAC

>read 313

CAAGCGTAATACTAGTTTGTCCTATGCTTTTGTATCATTTATTTTTTTTTTAATTAGAGCTTGCTATATAATGAGGGAGGATCGAGCATGGATGTATAAAAACGGTTAGAAGGGAAAATTCCTTAGTCCAACCTTTTCTTGAAAAGGTCGATGAATTTATTACATTTGCTACCACACAAGGGAATGTCGTGATGAATGGTGTGATGAAATGTCCATGTGCACGGTGTCAATATTCCCTTATCAAGATCTTGATACCATTAAGGAGCATCTCTAGGCATGTGGTTTTTTGCTAACTTGCTATTTCCAATGGGTTTTTCATGGTGTTGCACTTCAAAGAGAATCCCAAAGTAGCAGTTCCACTTATCCACCGAGGATGCATTGAACCCCGTATAGAAACATGGTATTAGATGCCTTTGGACTAGAAGGTGGTTTAGAAAATATTGAAGAGAGCCACATGCTAGTTACCGCAGTTCTTTCACATGTTGAAGGCAGCGGAAGAAGCCATTATGATGGGCTGAGATTATCTGTATTATGCTGCCAGTACACCAGGATGTAAACATCAGAATCAGAGTATAATATTCACAGTAAAGCTATAGATGGTGTTGCTTCCTTAATTAAAGATAGTTTTTTACAAGACTAAGAAATTGCCCAGAGGGCTGAACTTCCCGCACCAAAAGATTCACATCATATGTCCAAATGGATGTATGCTATTTTGGAAGGAACCACAAAGATCTCGAAGAATGTCTATATTGCGAAGGAAGTCGTTGCCAAGACACTTGAAATTGGCGGCAATTACTCCTCCTTAGTGCTCTTATTTACTTTCTGTAGGCCGGGATTACAAGATTATATGCAACAAGATCTACTGCGGAGAAGATGAGGTGGCATAAAAGATAATCCTCAGGTTCATGGCCTTATGTCTCGTCAAGTGATGAAGGCGTGGAAACACCCTATAGATGAAGAGTACCCTTCTTTGCTGCGGAGCCTAAATGTCAGGCCTAGGTCTAGTCCTTATGCAAACAGGATTTTCACCATTTAGTTAGACGGGGAAGGCAATATTCTTGTTGGCCCGTCATTTTTGACTCACTTATAATCTTCCACGGGTAATGCGTGAAGAAACATTTTTATGTTTTTAAGTTTGAGAAATAATTCAGGTCAAGTCCCTAAAGGGAATCTCGATGTGTGCCGCGACCTCTTATTGAAAGTTAAAGCAATTATGGGGAGAGGCTGGGTTCTGGAGCTTACAACATCTCGCAAAAACAATTTTCATTTGACTCAGTTAGCTTTGTATAATGTGTTTGACTTTCATTTGACTCGATTAGGTTTGTTAATTACATTTAACTTTCATTTAATTCTACAAGCTTTGTTGAATGTGTTGACTTTTATTTGACTCAATTACCTTTGTTGAATGCATTTTACTTTCATTTGATTCAAAAAGCTTCGTTTGAATGCGTTTGAATTTCATTTGACTTCCAGTCTTAGCTTAGTTGAATCACGTTTGACTTTCATTTGATTCAAAAGCTTTGTATGAATATGTTTACTTTCGTTAGACTCGATTATCTTTCTTAGATGCATTTTGTCTTTCATTTGATTCAAAAACCATTGTTTAGATGTGTTTGACTTTCATTTGACTCAATTAGCTTTGTTGAATACATTTACTTTCATTTGATACAAAAGCTTTCTTTGATATGTTTGACTTTCATTTACTCAATTAGCTTTGTTGAATGCATTTGACTTTAGTTTGATTCAAAAGCATTGTTTGAATGTGTTTGACCTTCATTTGACTCAATTAGCTTTGTAGACTGCATTTGACTTTCATTTAACTCAATTAACTTTGTATGAATGTGTTTGACATTCATTGGCGTGGGGAAAACTAAGGTCGTTGGGCTAATTCTCCCTAAGCTTGAGGCTCAGGGTATAAACACTCGACAAACCTAAATACAAATATAGAGGAATAAGTCAAGGGTCGGAGCCCAAAGGAACGGTAATGTCTAAGTAATGCTTTCAATTATATTGCCTAAGCTAGGTCGAAAACAAAGGGGAGTAAAAGGTGACTACAACAATTACCTAAACAGAAGAAACCAAACAAATATAAGAGCTAGGGATTAGGATCCGCAAATAATGGCAATGAACGATGCAAATTGGAATGGAAAATCGAATAAGTCAAGCAATGTGACGGAGAAAATCAAATCATGCTTAACCAAAGAACGATTACCTTTGTTGAATGCATTTGAATTTCATTTGATTCAAAAGTATTGTTTGAATGTGTTTGACTTTCCAATTGACTCAATTAGCTTTGTTGAATGCATTAGACTTTAGTTTGATGTAAAACCGCTTTGTTTGAATGTGTTTAATTTCATTTGACTTCAGTGGTATTTTGTTGAGCAGCATTTGACTTTCATTAGTTTCAAAAAGCTTTGTATGTGTGTGTTTGACTTTAATTTGAGCACAATTATCTTTATTGAATGCATTTGACTTTATTTGATTCAAAGCATTGTTTGAAGGTGCTTGACTTTGTTTAGTCGAAAATCATTATTGAATGCATTTGGCTTTTATTTGATTCAAATGAGCTTTGTTAAGTGTGATGACATTCATATTATACTTAATTATGTTTGTTGAATGCATTTGACTTTCATTTGATTCAAAAAGCTTTGTTTGAAGGTGTTGACTTTCATTTGAGTTATAAAGCATTGTTGAATGCCTTGACTTTCGTTTAATATAAAGCTTTGTTTGTATGTGTTTTTGACTTTCATTTGAGTCAATTAGCTTTGTTCAATGCATTTGACTTTCATTTGATTCAAAAGCTTTGTTGAATGTGTTTGACTTTCATTTGACTCAATTATCCTTTTTGAATGCAATTGACTTTCAATTGATTGAAAGCTGCATGAATGTGTTTGACTTTCATTTGACTCGATTAAGCTTCGTTGAATGCATTTGAGTTTCATTTGATTAAAAAGCTTTGTTTGATTGTGTTCCGAAGCTTTCATTTGGTTCAAAAAGCTTTGTTGAATGTGTTTGATTTCATTTTACTCAATTAGCTTTGTTGAATGCATTTGACTTTCATTTGATTATAAGACTTTATTTAAATGTGTTTGACTTTCATTTAGTTCAACAAGCTTTGTTGAATGAGTTTGACTTTCTGTTTCTCAATTAGATTTTTTGAATACATTTGACTTTCATTTGATTAAAAAGCATTGTTTGAATGTGTTAGACTTTATTTAACTCGATAAACTTTGTTGAATGCATTTGACTTTCATTTGATTTAAAAGCTTTATTTGAATGTGTTTGACTTTCATTTGAATGAAAAGCTTTATTAAATACATTTGAATTTAATTTGATTCAAAAGCTTTGTTTGAATGTGTTTGACTTTCATTTGACTCAATTATCTTTGTTGTTTAATGCGTTTGACTTTCATTTGATTCAAAATGCTTTGTTTGAATGTGTTTGACATTCATTTGATTAAAAGCTTTGTATGAATGTGTTTGACTTTCATTTGAGTCAATTATCTTTGTTGAATGCATTTGACTTACGTTTGATTCAAAAACATTGTTTGAATGTGTTTAACTTTCATTTGACAATTAGCTTTGTTGAATCTCGTTTGTTTCATTTGATTAAAGTCAACGCTGTGAATGTGTTTGACTTTCATTTGACTCGATTATCTTCGTTGAATGCATTTGACTTTCTACTTGATTTTAAAGCTACTCTGTTTGAATGTGTTTGACTTTCATTTTGGTTCAAAGCTTTTTGTGAATGAGTTTGTCTTTCATTTTACTTTCGGTTAGCTTTGTTGAATGCATTTGACTTTCGTTTGATTAAAAAGCATTGTTTGAATGTGTTTGACTTTCATTTGACAATTAACTATGATGAATGCATTTATCTTGATTTGATTCAAAAACTGTTTGAAAGTGTTTGACTTTCATTTGACTTCGATTACCTTTATTAGTGCATTTGACTTTCATTTGATTCAGCTTTGTATGAATGTGTTTGACTTTCATTTGACTCAGTATCCTTGTTGAATACATTTGACTTTTTTTCATTTGATTTCAAAAGCTTTGTTTGAACGTGTTTGACTTTTATGCGAGTTAAAAATCTTTGTTGAATGCATTTGACTTTCATTTGATTCAATATATCTGTTTTATTGCTTTTGAATACAAGTTGTTTTACTAGTTGTTGCTAGATTTCCTCAAATTTTGTCTTGTTTTCATATGTTAACAAATTCTATCAACTAATTTTTTTTATTTGTATCGATTTAGAAACAAATTGAAACTGACAAATTTTGAATTGTTCCAATTAGAAATTAGCCGTCGTACTAGTTGTTGCATTTGCATTCGTTTGATTAGCTTTGTTGAAAGTATTTGACTTTCATTTGACTGATAAGCTTTGTTGAATGCATTTTGACTCATTTGATTCGAAGAGCTTTGTTTTGAATGTATTTGACTTTCATTTGAGTCAAAAAGCTTTGTTGAATGCATTTGACTTTCATTAGATTAGCTTTGTTTGAATGTGTTTGACTTTCATTTGAGGTTAAAAGATATGTTGAATGCATTAGACTTCCATTTGATTAGCTTGATTTAAATGTGTTTGACTTTCATTTGACTTCGATTATCTTTGTTAGATACATTTGACTTTCATTTGATTGAAAAGCTTTGTTTGAATGTGTTTGACTTTCATTTGACTCGGTGTCTTTGTTGAATGCATTTGACTTTCATTTGATTTAGAAAGCGTTGTTGAATGTGTTTGACTATCATTTGACTCAAATTAGCTTTGTTGAGATGCATTTAAGTTTTATTTGATTAGCTTTTGTATCCAATGTGTTTGAGACTTTCATTTGACTTGATTACCTTTGTTGAATCATTTGACTTTCATTTGATTGAGCTTTGTTTGAATGTGTTTGACTTTCATTTGACTCGGTGTCTTTGTTGAATGCATTTGACTTTCATTTGATTTAGCGTTGTTGAATGTGTTTGACTTTCATTTGACTCGATTAGCTTTGTTGAATGTATTTAAGTTTTATTTAATTCAAAAGCTTTGTATCAATGTGTTTGACTTTCATTTGACCCAATTACCTTTGTTGAATGCATTTTGACTTTCATTTGATTCAATATATCTCTTTTTATTTGCTATTGAAACAAGTTGTCTTACTAGTTGTTGCTAAATTTCATCCAATTTTTGTCGTTTCCATATATCAACAAATTCGACAACTAATTTTTATTTGTAGCGATTTAGAATCAAATTGAGGCACTAAATTTATGAGTTGTTCCTGTAGAAATTAGCCGTCTCGTTCTTGTTGCTAGATGACAACGGATTTTTATTTCTTTGTCATTTAAGCGATTGCCATTTTACAAAGCGTTTGGCAACAAAATTTCCTAGTTGTTGCTATTAGGGACACTTGTTTACTATTTCTTGCTAGTAATAATTTAATTTGTTGCCATTAGAAAGAAAATTGTGTTATAAATTGTTGCTAATTTATTAAGAGTCATTATGTCAATTTACATAATATGTTTTAATAAATAAAAGCAGAAAACAAATCATCTTGTTCTATACAAAGAAACATTAAGAAAGAATTAATAATCGTTCAATCAATTTTAGCAATAAGAAAGCAGAAGGCGATAAAAATAATATATATTATTTTATAAGTTCATTAGAAGAGCATGTATAAAATGGGAATGCTTCTTACTAATATGGCCAATTGCTTGATTTTGGTTCTACAAGACCTTAAAACCTTATAACATGCTATGTTTTTGTGATTTCTTTCTTAGGAAGTTATAAATACATATGTTTGACATGAATCAAGCCTTTTTAAAGTTCATTAAGAAAGAGCATGTAAAAAATGGGAACGCTTCTTCCCATTATGGCGGAATAAACGATTTCTGTTCTAATAGACCTTAAAACCTTATATCTTGCTTTTTCTTAGTGATTTCTATGCCCAGCCAGTTACTAATACATATATTTGACATCATTCAAGCCTTTATAATTTCATTAAAAGATGAAGCCGAGAAAAATGGGAACGGCTTTTATCATTTAGGCCTTGAATTTGAAGGATTTGATTTTGGTTCTACAAGACCTTAAAACCTTATAACATCCTTTGTTTTTGTGATTTCTTTTTTAGGAGATTATAAATATATATTTGACATGAATTCAGAAAATCCCTTTTCAATCTCATTAGAAAAGCATGTAAAATATGGGTGCTTAACACCTATTTTGCTCGAATTTGTTTGATTTAGATTCTACCAGTCCTTAAAATCTTATAACTTGCTTTGTTTTTGTTATTTCTATGATTAGGAATTATAAATACATATATTTAACATTATTCAAGTTTTTGTAGTTCATTAGAAAGAGCATGTAGAAAATGAAATACTTAACTTTCTATTTTACTTTGAATTGCTTGATTTTGGTTCTAAAACTTTTAAAACCTTATAATTGCTTTGGTTATTGTCATTTCTTTGGTTAGGAAGTTATAAATACATATATTTGACATATGAATCAAGCCTTTTCAAATGCATTAGAAAGAGCATGTGGAAAATAAGTACACTTTTTCCCGGCTTTGCCCAAATTTTAGCGATTTAGGCTCTACTGACACCTTAAAACCTTATAACTGCTTTTTTTGTGATTTGTTGCTTAGAAATTATAAATACATATATTTATATGATTCAAAATTTATAAATTCATTAAGAAAGAGCATGTAGAAAATGGAAAGGCTTCTTACTATTTTGCCCGAATTTGCATGATTTTTGTTCTACTAGACCTTAAAACCCTAATATCTTGCTTTGTTTTGTGATTTTATATTGTTAGGAAGTTATAAATATATATATTTGACATGAATCAAGTTTTCAAGTTTATTAAAAGAGCTTGTAAAGGAATGCTTCTTACTATTTAGCCCAGAATTTGATTGATTTCGGTTCTGAGACCTTAAAACCTTATAAATTTGCTTAGTTTTGCGATTTCTATGCTTATGCATTTATAAATACATATATTTCACATGATTCAAAACAAATTATAAAAGTTCATGAAAACATGTAGAATTTGGGAATGCTTCTTACAATTTTAAAATTTGATTGATTTTGAATCTGAGACCTTAAAAACCTCATAACTTGATTTGTATTTGTTATTTATTTTATAATGGAGTTATAAATACATATATTTGACATCATCCAAACATTTATAAGCTCATTAGAAGAGCATGCGAGGAAAATAGGAACGTTTCTTTCCATTTTGCCCGAATTTGCTTGATTTTGGTTCCTGAGACCTTAAAACCATATAACTTTTGCTTTTCTTTGTTATTTATTTGCTTAGGCAGTTATAAATATGTATATTTGACATGATTCAAGAATTTATAATTTCATTAGAAGAGCATGTAGAAAATGGGAATGTTCTTACTATTTTGCCGCAATTTGCCCGATTTTGGTTCTACGAGACCTTATAACCTTATGAATTGATTTCTTTTTGTGATTATTTTTTGTGAGGCAGTTATAACTACATATATTTGACATTACTCAAGCCTTTTCAAATTCATTAGAAGAGCATGTAAAACGAGTCACTTAGCCTTCCCGTTGGCCGTATTTGGCAATTTTTGGCTCTACAAGACCTTTAAACCTTATGACATGCGTTGTTTTTGTCATTTATTTTCTAAAGGAAGTTGTAAATATATATTTTGTGATCAAGCCACACACTCAAGTTCATTGGAAAACATGTAGAAAATGGGAATAATTTGCTTCTCATTTTTCCGAATTGCTTGATTTCGGTTCTACCGGACCTTAAAACCTTATAACTTGCTTGGTCTTAGTGATTTCTATGCTTAGTCAGTTACAAATACATATATTGACATTATTCAAGCCTTTATCAGTTCAATAGAAAGAGCATGTAGAAAATGGGAACTCTTAACTTCCCGTTTGGACCCAGTGCTTAGTTTTAGTTTAAAGACTGTAAAACCTTATAAAATGATTTGTTTTGTGACTTCTTTTCTTAGGAAATTATAAATACATATATTTGACATGAATCAATCCTTTTCAAGTTTATTAGAAGAGCATGTAGAAATTAGAGCCGCTTCTTCCCATTATGCCCGAATTTTGCTTGATTTCGGTTCTACTAGACCTTTAAACCTTATACCTTGCTTTGTCTTAGTGATTTCTATGCTTAGGCAGTAACTAATACATATGTTGACATAATTCAAGCCTTTATAAGTTCATTAGAAAGAGAATATAGAAAATGGGAGCAGCTTTACCATTTGGGCCAATTTGGTTGATTTGGTTCTAAGACCTTAAAACCTTATAACATCTTTTGTTTTGTGATTTCTTTTGTTAGGAAATTATAAATGCACATATATTTGACATGAATAAAGCCTTTTCAAGTTCATTAGAAAGAGCATGTAAAATATAAGATTTTTTTCCTATTTTGCTCGAATTTAGCCCAGTTTAGTTCTGAGTCCTTAAAAGCATAACTTGCTTTATTTTGTTATATCGTGATTTGGAATTATAAATACATATATTTAACATTATTCAAGTTTTTGTAGTTCATTAGAGAAGAGCATGTAGAAAATGAGAATGTTTCTTTTTATTTTGCAGATTTTCTTGATTTTGGTTCTACAAAACCTTAAAACCTTATAACTTGCTTTGGTTTTTGTGATTTCTTTGGTTAGGCAGTTAGCAGATACATATCTCTCGATATAGATTAAGCTTTTAAAGTTCATTGATAGAAGAGCATGTGGAAAATAGATACGTTTTTTTCCCCATTTTGCCCAAATTTCCCTTGATTTAGGGTCCTGACACTGCAAAACCTTACTCAACTTGCTTTTTTTTTTGTGATTGTTGCTTAGAAAATTATAAATACATATATTTTATATGGTTAAAGAATTTATAAATTCATTAGAAAGAGCATGTAGAAAATGGAAAGCTTCTTACTATTTTGCCCGGTTGCATGATTTTGTTCTACTAGACCTTAAAACCTTATATCTTGCTTTATTTTTGTGATTTATATTGTTAGGAAGTTATCAAGTTTATTAAAAAGAGCATGAAAAAAGGGAATGCTTCTTACTATTTGCTAAATTTGATTGATTTTAGTTACACTGAGACCTTAAAACCTTATAAATTGCTTTGTTTTTGTGATTTCTACGCTTATGCATTTATAAATACATATATTTCACTTGATTCAAACAATTATAAGGTCGTAGAAAGAGCATGTAGAATTTGGGAATGCTTCTTACAATTTTACCCAAATTTGATTGATTTTGGTTCTACAAGACCTTAAAACCTCATAACTTGATTTGTATTTGTTATTTATTTTTAATGGAGTTATAAATACATATATTTGACATAAATCCGAGGAAACATTTATAAGCTCATTAGAAAAACGCTGAAAATGCGGACGTTGCTTTCCATTTTGCCCAGTAACAAATTTTGGTTCTACAAGACCTTAAAACCATATAACTTGCTTTTGCTTTGTCATTTATTTGCTTAGGAATTTATAAATACGGCTTTTCACCACATACCCTTGGGTTTTTGTTTATTCACCATATACTTACTGAAAATTTTCAAATTCACCCATGTGTGCCCCTAAGTTGTAATAAAAATTAAGCCGTATATCCTTAATGAATATAAGATGAAAAGAATTTGGTTAATTATATCAGAAATCACCTTAGATATCACCACTTCATCCCCAAAACAACTCTTACTCATTTCACAGCCCACAAACCACTCCAAATCTCCCATAACCATAACCATATAAATAATTTTGAGGATTAGAAAATGAAATTTAGAGTAAGTTTGGGGTGCAAAAAAAAGGAATGTGGGTGGTTTCGGGGTAGAGGAATGAATTTAGAGTGGTTTTAGGTTCATAGTATCCCCGAATGATTGACCAATTTAACAATAATATGTTATCAAAAAGGTATATGGTGTATTTTGTGAAAACTCATGAATTTATATGATGAAGTTAAAAGTTAATGGGTATATGATGAATAAACAAAAACTCAATGGCATATGGTGAAATATCCGTTATAAATATGTATATTTGACATGATTCGGGACTTTTATAATTTCATTAGAAGAGCATGTAGAAAATGGGAGATGCTTCTTACTATTTTGCCAGAATTTGCTTGATGTTGGTTCCTGAGACCTTAAAACTTATGGTGCTTTGTTTTGTAATTTATTTTGTGAGGAAGTTATGAATACATATATTTGACATGACTCAAGCCTTTTATAGTTCATTAAGAAGAGCATGTAAAAAATGGGAATGCTTAACTGCCCATTTTGGTAGAATTTGTAATTTGGCACAAGACCTTAAAGAGCCTTATGACATGCTTTATTTTTGTGATTTTATTTTCTACAAGAAAGTTGTAAATACACATATATTTTACATGAATCAAGAAAGCCTTTTCAAGTTCATTAGAAGAGCATGTAGAAAATGGGAGCCAACACCTTCTCGTTTGCCCGAATTTGCTTGATTACGGTTCCTGCGGATAGCAAAACGTTATAACTTGATTGGTAGTGAATTGCATGCTTAGTCTGTTTACAGACATATATTGACATGATTCAAGCCTTTATCAGTTCACTAGAAGAGCATGTAGAAAAGCGGAGCAGCGTCTTCCCATTTAGGCCGAATTTGCTTGATTTTGAATACAAGACCTTAAAACCTTAAAAATTGCTTTGTTTTTGTTATTTCTTTTCTTAGGAATTTATAAATACATATATTTTACACGAATCATGCCTTTTCAGAGTTCATTAGAAAGAGCATGTAGAAATTGGGAGGCAGCTTCTTCCATTATGCCCGGGTAGCGATTTCGGTTCTACTAGACCTTAAAACCCTTATACCTTGCTTTGTCATAGTGATTTCTATTCTTAGGCAGTTACTAATAAATATATTTGACATCATTCAAGCCTTTATAATTTCATTAGAAGGAGCATGTAAGCAGAACGCTTAACACATTTGTGAATTTGCTTGATTTTGGTTACAACGCTAAACCCTATAACATACTTTTTTGTGATTCTTTTCTTAGGAAGTTATAAATACATATATTAGACATGAATCAAACCTTTTCAAGTTCCATTAGAAAGGTGTATTTGAAAAATAGGAAACGGCTTCTCATTTTTTCCTTAATTTGCTTGATTTAGGTTCTGCGGACCTTAAAACCTTATAACACTTTGTTTTTGTGATTTCTATGCTTAGAATTTACAAAAAATATATTTGACATGACTCAAGCATTTATAAGTTTTTAGAAGAAAACGTATGAGAAAATGGGTGCTTCTTGCTATAATTTTTAGAAAATCTTGATTTTGATTTACTGAGACCTTAATACCTTATAACATCCTTTTTTTTGTGATTCATTTTTGTTATGAAGTTATTAATACATATATTTGACATGATTCAAGCCTTTTCATGTTCATTAGAAAAGAGCATGTCGAAAATGGGAATGCTTCTTCCCAGTATTTGCTTTCCGGAAGTTGCTTGGTCGGTTCTGAGACCATAAAACCTTATAACTTGCTTTTTTTTATTTCTATTTTTAGGCAGTTATAAATCCATATATTTAACATGATTCAAGCATTTATATGTTCTAGAAAGTGTATGTGTAGAAATGGGAGTGCTTCTTACTATTTGCCCGAATATGTGATTTTGGTACCACAAGACCTTAAAACCTTATAACATCCTTTTTTGTGATTTCTTTTTTAGGAAGTTATGAATACATATATTTGACATAAATCAAGACTTTCAAGTTCATTAGAAGAGCATGTAGAAAATGGGAGGCAGCTTCTTCTCATTTTGCTAGAATTCGGTTGATTTCCGGTTCTACCGGACCTTAAAAAACCTTATAACTTGCTTTGTCTTAGTGATTTCTATTCTTAGGGAGTTACTAATACATATATGTAACATGATTCAAGCTTTTATAAGTTCATTAGAAGAGCATGTAGAAAATAGGAGCAGCTTCTTCCCATTTGGGCCAAATTTGGTTGATTTGGTTCTGAGACCTTTAAACCTTATAAAATCCATTGTTTTTGAGATATCTTTTTGTTAGGGAAGTTATAAATACATATCTTGACATATTTCAACCTTTCAAGTTCATTAGTAAGTGCATGTAGAAATGAAACTGACTATACCGTTTGCCTTAATTTGCTTGATTTCGTTTCTACAGGACCTTAAAAACTTATAACTTGCTTTGTTTTGTGATTTCTATGCTTAGGAATTTACAAAAATATATTTGACATGACTCCAAGCATTTATAAAGTTTTTAGAAAGAAACAGAGAGAGAGTAGAGGATAGATTGCAGCTGTAGCATTTTGCCCAGAAAATGCTTGATTTTGGTTACAAGACCTTAAAACCTTATAGCATCCTTTTTTTTGTGATTCATTTTGTTATGGAAGCTATTAATACATATATTTGACATGACTCAGGCCTTTTCATGTTAATTAGAAGAGCATGTAAAATGGGAATGCTTTTTCCCATGTTGCTCGGAGAATTTGCGATTCGGTACACTTCTGAGACCATAAAACCTTATAACTTGCTTTTTTATTTCTATTCTTAGGCAGTTATAAATCCATATATTTGACATGATTCAAACATTTATAAGTTCATTAGAAATAGTATGTAAGAAAATAGAATGCTTCTTACTATTTTGCCCAGAATATGCTTGATTTTGGTACTACAAGACCTTAAAACCTTATAACATCCTTTTTTTTGTGTTTATTTTTAGGAAGTTATGAATACATATATTTGACATAAATCAAGCCTTTTTCAAGTTCATTAGAAAGAGCATGTAGAAAATGGGAGCCGCTTCTTCTCATTTTGCCCAGTCAGCTTGATTTCGAGTTCTCTGGGACCTTAAAACCATATTACTTGCTTTGTATTAGTGATTTCTATGCTAAGGGAGTTACTAATACATATATGTAACATGATTCAAGCCTTTATAAGTTCGTTCGAAGAGCATGTAGAAAATGGGCAGCTTCTTCCCATTTGGGCCGAATTAGTTGATTTGGTTCTACAAGACCTTTAAGCCTTATCAAATCCATTGTATGATCCTCGGCGTTTTATAAGTTTATTAGAAGAGCTTGTAGAAAGTAAAATTAAGCTTCTTACCATTTTTCCCGAAAATGCTTGATTTTGGTTCCTGAGACCTTAAACCTTATAACATCCTTTTTTTTTTTGTGATTCATTTTGTTATGAAGCTATTAATACATATATTTGACATGACTCATGCCTTTTCATGTTCATTAGAAAGAGCATGTGAGAAAATGGGAGTCACTTAACCACATTTGCTTTCGAATTTGCTTGATTCGGTACTACAAGACCATAAAACCTTATAACTTGCTTTTTTTCATTTCTATTCTTAGGCAGTTATAAATCCATATATTTGACATGATTCAAGCATTTATAAGTTCATTAGAAATAGTATGTAAGAAAATGGAATGCTAACACTATTTTGCCCGAATATGCTTGATTTTGGTACTACAAGACCAAAACCTTATAACATCCTTTTTGTGATTTATTTTTAGGAAGTTATGAATACATATATTTGACATAAAAATCAATCCTTTTCAAGTTCATTAGAAAGAGCATGTAAAATGGGAACGCTTAGCTACCATTTTTGCCGGGTCGACGATTTCGGTTTTACGGACCTTAAGACCTTATCACACTTTTTTGTCTTAGTGATTTCTATGCTAAAGGGAGTTACTAATACATATATATGTAACATGATTCAAGCCTTTATAAGTTCATTAGAAAGAGCATGTAGAAAGTAGAACCCTTCTTCCCACATTTGGGCCAATTTGGTTGATATTGGTTCTACAAGACCTTTAAACCTTATAAAATCCATTGTTTGTGATTTTCGTTTTTAGGAAGTTATAAATACATATATTTGACATGACTCAAGCCTTTTCAAGTTCATTAGAGATGTACATGTTGAAAGTAGGGACGCTTCTTCTCTGCATTTGCTGGATTTTGCTTGATTCGGGTTCTATCGGACCTTAAAACCTTATTAATTGCTTTCTCTTTGTTATTTATTTTTTCTTAGGAAGTTCCCACAAATACATATATTTGATGATATGATTCAAGCCTTTATAAGTTCATTAGAAGGGAGCATATATAGAAAATAGGAGCAGCTTGCTTCCGTTTTGGCCGAATTTGCCTTGATTTTGGTTCTACAAGACCTTAAAACTTTATAACATGCATTTTTTGTGATTTCTTTTCTTAGGAATTTATAAATACATATATTAGACATGAATCAAGCCTTTTTCAAGTTCATTAGAAATAGCGTGTAGATAATGGGAGGCAGCTTCTTCTCGTAATATGTTAATTGAGTATGATAATTTTCATTTGATGACCTTAAATAGTTAAATGACCTAAAGAATGTCATTCTTCTAAGTGCTTTACTTAGTTTGTCTATGCTTTTTATCATTTATTTTTTAGTGAAGGCACCTTTGTATATAATGAGGAAGTCGAGCATGGATGTATAAGCGGTTAGAAGGAAAATTCTGAGTCCGACCTTTTCTTTGGAAATGGTCAATGAATTTATTACATTTGCTACCACGCAAAGAGAATGTCGTGATAGATGGTGTGATGAAATGTCCATGTGCACGATTATTAGAAATATTCCTTATCAAGATCTTGATACCATTATGGAGCATCTCTATAGGCATGGTTTGCTAACTATTTCAATAGGTTTTTCATAGTGGCTTGCTTCCAAGAACCAAAGTAGCAGTTCCACTTATCCATAGAAAGGATGCATTGAACCCAGCAGAAACATGGTATTAGATGCCTTTGGACTAGAAGGTGGTTTAGAAAATATTGAAGAAAGAGCCACATACTAGTTACGAGATAGCACTCGCATGTTGAAGGCGGCGGAAGAGCCATTGTATGATAGGCGCAAGTTACTCTGTATTGTGTCTGCCCGGTACCAAGGATGGCAAACATCAAGTCCAGTATAATATTCCACATAAAGCTATAGATGGTGTTGCTTCCCTTAATTAAGATAGTTTTTACAAGACTAAGAGGGTAGCGAAGGGCGAACTTCCGCCTGAAAGATTCGTATGTCCCGAATGGATTTATGCTATTTTGGAAGGGACACAGATCTCGAAGAATGTCTATATTACCCAAAGGAAGTCGTTATAAGACACTTAGAGAAAAGTGGCAGCAATTCTCCTCCCTTAGTGCTCTGTTACTTTCTATTAGGCAGAGATTACAAAGATTATATGTGACGAGATCTACTGGAGCGAATGAGGAGAGGCATAAGATAATCCTCGAGTTCATGGCCTTATGTCTCATCCAAGTGATGAAGAGAGGCGTGGAAACACCTAGATGAAAGAGTACCCTTCTTTTTCTTTGCCGGGCCCTAGAGGAAATGTCAGAGCTAGGTCTTTGTACCGACGGATTTTCACCATTTGGAAAAACGAGGAGAGCAATATTCTTGTTGGCCCGTCATTTTAACTCACTTATAATGTTCCACTGAATTATTGCATGAAGAAACCTTTTATGTTTCTAAGTTTGAGTAATTCAGTCAGAAGAATCCTAAAGGGAATCTCGATGTGTACTTGCGACCTCTTATTGAAGATTTGGAAGCAATTATGGGAGGCTGGGTTACCGACTTACAGCATCTCGCAAAAACAAAATTTTCATTTGACTCAATTAGCTTTGTATAATGTGTTTACTTTCATTTGACTCAATTAGCTTTGTTAATTAATTTAACTTTCTTTTAATGACAAGCTTTGTTGAATGTGTTTGGCTTTATTTGACTCGAATTACCTTTGTTGAATGCATTTTACTTTCATTTTGATTAGCTTCGTTTGAATGCGTTTGAATTTCATTTGACTCAATTAGCTTAGTTGAGATACCTTTGACTTTCATTTGATTCAAAAAGCTTTGTATGAATATGTTTTACTTTCAGTAGACTAAATTATCTTTCTTAGATGCATTTTGTTTTCATTTGATTAATCAATGTTTAGATGTGTTTGGCTTTCATTTATTTCAATTAAGCTTTGTTGAATGCTTTTACTTTCATTTGTCTGGCTTTCTGGCACACTGATGTGTTTGACTTTCATTTACTCCACAATTGAAAGCGCTTTATTGAATGCATTTGACTTTAGTTTGGTTCAAAAAGCATTGTTTGAATGTGTTTGACTTTCATTTATTTCAATTAGCTTTGTATGAATGTGTTTGACATTCATTTGACTCAATTAGCTTTGTTAATTGCATTTCACTTTCATTTAATTAGCTTTGTTTGAATGTGTTTCGCTCATTTGATTGAAAAAGCTTTGTTTGAATGTGTTTTGACTTTCATTTGACTCAATTACCTTTGTTGAATGCATTTGACTTTCATTTGATTCAAAAAGTATTGTTTGAATGTGTTGACTTTCAATTGACTCAAATTAGCTTTGTTGAATGCATTAGACATTCGATTGATTAACTTTGTTTAGATGTGTTTAATTTCATTGACTTCGATTATTTGTATTGAATGCATTTGACTCTCATTTTGATTAATCTTTGTTTAAATGTGTTTGACTTTTATTTGAGTGGTTTGTTGAATGCATTTGACTTTCTTTTGATTAGCTTTGTTTGAAGGTGTTTAATTTTCATTTTAACTACGACTATCTTTACTGTTGAATGTAATTGACTTTCTTTGATTGAAAGCTTTGTAAAAATATGATAAATTTACCGTTTATTTCAGTAGCTTTGTTAATTAAATTTCATTTTCATTTAATTCAAACGATTTGTTTGAATGTATTTGGCGTTTTATTTATTTCGATTAACTTTGTTGAATGCATTTTACTTTCGTTTGATTAGCTTTTTAAGAATGTGTATATGACTTTCATTTGACTTAATTAGCTTTGTTGAATGCATTTGACTTTCATTTGATTCAAAAGCTTTGTTAGAATTGTGTTTGACTTTTCATTTGACTCGATTATCTTTGTTGAATACGTTTGACTTTCATTTAATTGAATTTGTATAGATGTGTTTGACTTTCATGAAAGTCGATTATCTTTATTGAATACGATTGTCTTTCATTTGATTAGCTTTTGTTTGAATACGTTTGACTTTCATTTGACTCAATTATCTTTGTTAAATGCATTTGACTTTCTTTTTTGATTAGCTTTGTTTGAATGTGTTTGACTTTAATTTGACTGATTATATTTGTTGAATGGATTTGATTTTAGCTTTGTTTAAAGGTGTTTGACTTTCATTTAAACCGATTATCTTTTTGAATGCATTTGATTCAAAACCAATGTTTGAATGTGTTTGACTTTCGTTTGTTTCAGTGCTTTGTTGAATGCATTTTACTTTCGTTTGATTAGCTTTCTTTGATGTGTTTGACTTTCATTTCTTTTCGATTAGCTTTGTTGAATGCATTTGACTTTAGTTTGATTAAAAGCATTATTTGAATGTGTTTAACTTTCATTTATTTCGATTAGCTTTGTAGGCGCATTTTGACTTTCATTTAACTTCAATTAGCTTTGAATCTGTTTGACATTCATTTATTTCGATTAGCTTTGTTAATTGCATTTCACTTTCATTTAATTGAGAAGCTTTGTTTGAATGTGTTTCACTTTCATTTGATTGAAAAAGCTTTGTTTGAATTTGTTTGACTCATTTGAATCAATTATCTTTGTTGAATCGTTTGACTTTCATTAAGATCTGAGCTTTGTATGAATGTGTTTGACTTTCCTTTGACTTAAATATCTTTGTTGAATGCATTTGACTTCATTTGATTGGCTTTGTTTGAATGTGTTTGACTTTCATTTCGTTAAATTATCTTTATTGAATACATTTGACTTTCATTAGATTGGCTTTGTTTGAATATGTTTAACTCATTTAGGTTAAAAGCTTTGTTGAATGCATTTGACTTTAATTTGATTGAAAAGCTTTTTTTTTAATGTGTTTGATTTTCATTTTGGTCAATTATCTTTATTAAGATACATTTGACTTTCATTTGATTCAAAATCTTTGTTTAGGAATATGTTTAACTTTCATTAAAATTGAAAAAGCTTTGTTAAATGCATTTGAATTTAAAATTTGACTCAAAAAGCTTTGTTTAATGTATTTGACTTTCATTTGACTCAATTATCTTTGTTTGAATATGTTTGACTTTCATTTTTCGCCTAAATGGTATTGATAGAATCGTTTTACTTTGATTTGATTGGCTTTGTTTAAATGTGTTTGACTTTCATTTCCAGAAAGCAATTATTTTTGTTGAATACGTTTGACTTTCTTTTGATTCAAAAAGCTTTGTATGAATGTGTTTGACTTTCATTTTGGCTCCAATTATCTTTATTGAATGCATTTGTTTCTTTTTTATTTCGATTAGCTTTTTATAAATGTGTTTATGCTTTCATTTTACTCAAATTAGATTTGTGAATTGCATTTCACTTTCATTAAATTTAAAGGTTTGTTGAATGTGTTTGATATTCATTTGATTAAAAAGCTTTGTATGAATGTGTTTGACTTTTCATTTGACTGAATATCTTTGTTAAATGCATGACTCCTTTGATTAGAGTTTATTTGAATGGGTTTGTCTTTCATTTTACTCAATTAGCTTTGTAGAATACATTTGACTTTCATTTAACTCGATTAGCTTTTTGTATGAATGTGTTTGATTTTCATTTATTTCAGTCTAGCTTTGTTAGTTGCATTTCACTTTCATTTAATTCGAAAAGCTTTGTTTGAATGTGTTTGACTTTCAACTTAATTGATTAGCTTTGTTAATTGCATTTCACTTTCATTTTATTCAAAAGTTTTGTTTAGTGTGTTTGACTTTCATTTGACTCAGTGAATGCATTTGACTTTCATTTAATTTAAAAAGCATTGTTGAATGTTTGACTTTCATTTGAATCCAAATTAGCTTTGTTAGTCAGTTTGACTTTCATTTAGTTGAAAAACTTTGTTTGAATGTGTTTGACTTTCATTTAGCTTAATTGGCTTTGTTAAATGCATTTGACTTTTCATTTGATTCAAAAGCTTTGTTTAGATGTGTTTTTGACTGCATATCCACTCGATTATCTTTGTTTAATCCATTCGACTTTTAATTGATTGAAAAGCTTTGTATTAATGTGTTTGACTTTCATTTTACTTAATTATCTTTATTGAATGCATTTGACTTTCATTTGATTTATAAAGCTTTGTTTTGAATTTATTGGACTTTCATTAGAGTCAAAGAGCTTTGTTGAGATGCATTTGACTAAAAAGCTTAGTATGAATGAGTTTGACTTTCATTTAACTCGAATATCTTTATTGAATACATTTGACTTTCATTTAATTTATATAAAGCTTTGTTTGAATGTGTTTGACTTCATTTGACCTGATTATCTTTGTTAAATACATTTTACTTTAATTTGATTTAAAAGCTTTGTTTGAATGTGTTTGACTTTGATTTAGTCTCAAAAGCTTTTGTTTGAATGTGTTTGCTCATTTTGACTTAAATATGTCATTGTTAAATGCATTTAAATTTTATTTAGTTCAAATGCTTTATTTAAATGTTTTTGACATTCATTTGATTCAAAAAGCTTCGTATGAATGTGTTTAGCTTTCATTTGACTAAATTATCTTTGTTGAATACATTTTACTTTCATTGATTAAAAAGGATTTTTGATTGTGTTTGACTTTCATTTATTTCGATTATCTTTGTTGAATGTGTTTGACTTTCATTTGATTAGCTTTGTATGAATGTGTTTGATTATCATTTGAGTCAATTATGTTTATTGAATGTATTTCACTTTCATTTGATTAGCTTTGTTTAGAATATGTTTGACTTTCATTTTGGGTCAAAAGCTTTGTTGAATGCATTTTGAGCTTCTAATTTGAGAGAGTTGCGCTAAAGAGATAGAGAGTGGTGCTAAGGGAGGAATGAAATTGGAAGGGTTGAGTCCTGAGAAATCTATTGTGAGAGGTTGACCAAAAGAGAGAGAAGAGTGTGTTATTTAGAAAAGATGAGTGAGAATTTAGAGAGATTTTCCGATCTACCCTTATCTGATAATATTTGAGATTCCAAAATGGATGAGCAATTTGAGGAAATTCCTTGAAGTTCTTAAGGATATTCATGATTCTACACCCTCTACTGAGCCAATTCTCGACATGCCCTAAGATACTCTAAGTTCCTTAAGGAGATCTTAGAGGAGAAGGAAGATTTGATGAGGTAGTTCGGTGGAAGGAGGGAAGTGCTAGTATCTTATCCATGGGTAATTCGAAGAAGATGGAAAGATCGAGAATTTTCAATCCCTTGTCAAATTAAGAAAATGGTTTTTTGAAAATGCTTTGTGTGATCTAGAGAGACTCTTTTGTAGTATTATGCCACTTTTCGGTCTTAAGGCTTGATCTAGATGACTTGACAATCTAATATAACACTTCAATTGTGGCCGATCGATCTATTAAGCTTCCTAAGGAAGAATTGATGATGTTCCCTTTAAGAGATTGGAGAGTTTGTCTTTCCCGTGGATTTTGTTGTTTTAGAGATTGAGGAGGATGAACATATTGATCATTTAGGGAGGCCTTTCTTAGCTACCTCGAGGAGCTTTAATAGATGTCAAAGGAGGTCAAATCACTTTGAGGTGTGGAAAATTAGAAGAGAGTTTTAAGTTGAAACCAATGCATGAATCCCTATCTTGTGTGAGATATCGCATGTGCTAGTCTTTCTCGTTCCATTGATGGTATGTGCATGATTGGTTCTTCTACTAACAATGTTTTTGAGATTGTTGATGATGTTTGTGGAATCTTAATGATGTTAGTGAAGAGGAATTGTGATTCAAAAGCCAGGGATGAGAAAGAAATTGAGTTTCAAGTGGTTCAAGCAATCTCCCGAGATGAAGAGAAAGCTTCCATGACTAAAAGATGTTGAAGTCGGCCCAGAAGAAAAAGGCCCGTGACTCGAAGAAAGCAAGGAAAAGGAAGAAGTTGAGAAATGAAAAGAAAAGGGTGAAAATTTTGTGAAAGTTTCATTAAGCAACGATGAGAAGTTGCTCCCGAGGGTTGAAAGAGAAGATTTTTGCTCTATCGACCACCTTGAAGAAGGTGGTGGCACCTTTGTCCTCGATTAGGAGGAAGGAGTCGAGGAAACTTAGGACCTTGTAACAGCGCTTCTGGGGAGCAACCCGGAGTTTCTAACCTTTCTCTTTGATTTTTTGAATTTTCTCAATTTTGTTTCTTTTATTACTTTGTCTTTTCTTTTACTTTTCTACTTATTTGGATTTTTAGGATGTCAACTGCGGGATTGGAACCCGGCAAGTATGCATGATGAATAGAAGGTGTTCATGGCACAAATCATATTTTGTTCGATTCTAGTCAGAATTTGAGGTAAAGGGTAAGAATTTTAGAAGGTAAGATAAATTCATGTTTAAATGTTGGAGGAAATTGATACTTAGTGAATTTTGTGGTGTGCACATGAATTTTCAACTTTGAGAGTTTGAAAATGATTTAGCTACTGCCTATGTTGTTTCCTCATCACCATCATGATTCATGTAGCACCAAAATAGGCGAGTAAGTATCAATTTTCGAGAAAAAAGAAGGAAGGGAGACGCACGGGGTGTCTTGGCCCAGCGCCACAGTGAGTGCGGGCGCCACGACTGGCTGGTGTGGCCTTTGACCTTCGCGTGTGCGGGCGCACACCGGTTACGTGCGGCCGCCGCGGCTACTGGGCGTGCACTATATCCTTCTTCCCTCCTTTTTCCTCCCCTTCATTTCAACATTTCCCTTCGTCTTCAACTCCACGAGCACCGCTCAAAACCTGCTTTATAGCAACTTCTTCCTCCGCATTCACCATCAAAAATCACACAAATCCTCGCTAAAATCACTCCATTTCACTCAAAATCACTTACAAACCTTATTACACTACAAATTCATACAAGGATTGACCCCAAATTTCACTCGCTCTACTCCTGCTCTTGGTGGCCGAAAATTGGGGCTTTTTGTTAAAGTTAATTGCTTGCTTTCAAGGTGGTTAATCTTGCTTCTTTTATGGTACATAATCACTAATCCTTGCTTTAATTTTGCCATTTATATCATAAATTTGCATGGATTACAAATTTGTATGAGCATGAATAATGTAGTATTGATTTTGTGATTTTGATGCTTGATCTTGATTTTAGTTGTGATTTGGTGATGTTTGTTGATTGATTTTGCATAACATTGTGTTGTTTGTTGATTTTTGTAGTGATTCTCATCATTTTGGTTGAAATCTTGTGGAGTTGAATTTTTGGAACATTGGTAGTATATGTTGATTTTGGTATTGGTCTTGTAGTTTGCTCTTTGGATTGTTGAAAGGAGTTTTCTTGGATCATTGGTATTTATTGTTGATTGGTTTGGAATTGGTTTGATTGAAAAAATTGTGAATTTGGGTTGGTGATTTTGGTATTAAGTATGGTGAATTTGGGTGTGGTGTTCTTGGTTCATTGGTTTTTGCTACTTGTTGGTTGTTGGTGATCTTCTTAGCCTCTCAAAATGTCTAAATACCTCGAGAAACTCACCCAGCGGAAAACCCGATTTTAGCATCGATGAACCTCCTCGCCCAAGTACTTCCTCTGAGCCTAATTACCTCTTCTAGGCCTATTTTAACCCTTCCTCTCCCACTCAAACCATTTGACCTCTCTTCCCAAAGAAAAACTTCACTTAGATAAACAAAAGACCTTTGCCAAAACACGAAAGATGAAAGAATCCTAAAAACAAACCATACGCTACTTACCCGGCCGATATCTTTGAGATTGGGTATTGATAATGATGCGGTCGTTGCTTTTAAATCTCAGTTAAGGTTCTTTCATCACCGTGCCTACCGCACCTATAAGGAGCCTACTTTGGAGTTCTTAGCTACCTTAGAGCTTGTACCCAGGGGGTATATGGGAATGCGTTTCCCGATTGTTCAACCACATCTTTGACCAATCTTTGGAAGAGTTCAACGCCGCTTTAGTTTTGATCCCCATGGGGATATTGTCAACGTTGAACACTTCATCTTCCCAGTATGATCAAAACTCATGGTGGGGAGAGATCTCCGGTGGTAATGAGTTTATCACCAACGGGGCTAAGATCTCGAGGAAGGTGTACTGACCTTGCCTTAGGGTTGTACGCGACTTTTGGGCTATGTTTTGTTCCCAAGCAAAAATGGGAAGTTGAGTAAATTAGACTCAAGGTCCTTTGGTTGTCTACTACTTGGGAGTATGTGCGTCGGTTGAACTTAGGCTATATCTTTGCTTGATATTGCTTGGAACCGCCAAGGAGGGACGGGGAGAGATCCCGTGGGGGTATGGTAACTAGGCTATAGACTCGCCTTTGCCTCAATGATTATCTCCCATTTTGTAAGGAGGATCGGGGATGCGGCTTCTATCTACCTCGTGTATGCCAACAATTGGGTCAAATCCAATTTATCTCCAAGGGCTTTAGTGAAGGGAGATATGAGTGAAGCCGGGCGACGAGCATTGGTGCACTTGCCTAACCTGGCTTGACTTCTTTGGCTAGGATTGCCAACAATGTGGCATGTTCCCAAGTCCTTAGGCATCCCAAATTTGACTCAGGGAGGATGAAGAAGATGATGAAGAAGAACAACGAGTCGAGAACTGACAACATCGGCAACAACGACATTATTCCCCTTGACAACAAAGCTCCTTGAAGCGAGAGCAAGAGCAAGGAGCAACATGCTTTCGCCAATGCAGATTGAGGGGCTCTTAGTGCGAGCAAATGTGTGGATTGAAAATCCGTGGATGGCATGTACGATCAAGACTTTGGGGCCATCGCTGACCAAATGAAGTATTCGAGATGCAATGAGGTATGTTTGCTCCCAATGACAACAAATTTATGAGGCGGAAGTTAGCAGGTGGGAACAGTACTCCCACAGTTAACTATCGTGCCTTCAAGCAACAAGGTCCTCTATCTTCGTTTGGGCATCCTTGTCGGGACAGCCAACCCCAGGCAGAGGCGTCCCAGTGCAGGACCGAGCATGGTAACTATGATTACAAGGCTCAGGGTGGTAGTGACTATAGGGGGACCCTAACTACCCCATGTCCATCAGCCACCCTTTGAACTCACTTTGCCCGATAACTTCACCCCTCTAGCTCCTTGGGAACAAGACCTTTTCAAGGGGCATTGTAGATGTGGCCCAGGGGGGCCTGCCCAGGAGGATGGGACCTTAACCTCATACCTTCGATGACCCAGAGCGCAAGTTCCAGTGGGAGCCAGCCGATGATGAAAGTGATGGTAGTAGTTATTAGTTCCCTTTAGGCACTCATCTTTACGCCGTCACATATTTTTTGATCTTTTGCTTTGTTGTTTTGATCGGGATGTTGAGATGTATTTTCCTATCTAGCTTGGGAGGCCAATAACTTGCTCGGTGACCATTTTGTTGTTGTATACATATCTTAGTTGCTTGCATGTTTATTTTCATTCCTCTTTTTGGTTTCCCTCATTTTCTTGATCTCTAGCCTCTTTCCTTGTGGTAGGCTGTTTCTTGATTTGCTTGGATGTTTGTTGTTTGTGGCGATGTTTTGCTTGGCATGTTGTTGTTGATGATCCCTTATGTCCAAATCCCTTAATCCAAAATCCCTTAATTCCAAATGTTGTAGTTTAGAGTAGTTAACAGTTAGTCTAGACTTTATTTCTTTTATTCTTTCTATTCTTTAGTTAGTTTGTTTTCTTTCCTTTGGTTGTATCTGCTTCTTGTAGTTTTATTTTCCCTTTTATTTATTGTTTCTTTTATTTGTTTGAGATTACTAACCATTTTCTTTGTTCATAGTTTCTTTTATTTCTTTCTCTAATAGTTCCTTCAAGACTTATGGATATGGCATGCTACGAGGTGGTTTATAATCGCTTCACTTGTACACCTAGCTGGAATCCAGGTGGGTCTTCTTATTTTCTCTTTATTTTCCTTGGTTGCATAGGATAGGATTAGTTTAGGAGAAGTTGAATGGTTGAGGATGGATGCTTGTTAACTTACGTAATGTGTTTGGGATCGGTGACTTTGTTGTCCTACTCGATCACTTGTTTGTCGATTAAGAGGTTATGTGTTTAACATGCTTTCTAGTCCTTTGCATGCTTTTCTCCTATGTTTCCTTTTCTATGTCTCCGTTCACTTTTCTTTTACCTCTCTTTTATTTTACCTTTTCTCTTACTTTCCTTTAATTTTCTTCTTATTTTTGATGAGATTTGAGGCATTGTTTTTCATTTCTTTCTTATGGGATGAACCTTGTACAGTGGACTTGTCTTTGCGTGACTTTCTTCCTAGGTTGTAAAGGTATGCGGTTTGAGATTATGTTCGGTTGACTCTAGGATAGTGTATGCTAGGGTAGTTGGGTTTGAACTAGGAGGATCATGAAGCTTGATTCTACTTGTTTGTGGTATGTTGGTAGTTGCTTCCCTATAGTTGATTTGGCACCACTAGGTTTTTGCCACATCCTTGACTATGCAAACCCTAATCTAATAAAGCTTGATGTATGATTCTCCAAACCACGAGACCCGAACTATCTAGGAAGGACAAAGGCACATTTTGGGATAATTATTTTCATTTTTTTCTATATTTTGTTATTTTCTTTATTTTTGGGTTTACATTTTGCCTTCTTGCAACCATTTGAGCCTTATCCCTTCTTTTCAAGCCACATCTTTAGTTATTTGACCTTTTGTTTCATTTTCTACTTTTCTTGATTTTTGTGTAGCAATATGATCATTTGAGATGAAGTTTGTGGTGGTTAAGTTGGAATTTTGGTTTTATGAGAGAAACAAGTTTGTGATTTCATCCCTTTTTGTGAATAATGGCAAATATATTTGGAAGAGTCGTCCCTACTTCTCAAAGGCATGGACTAATAATTTGCATTTAGGAAAAACTTCGTCAATTTAGCAAAAGGCAAGGACTTTGCAATTACAATATAAAAACACAAAAAAAAATAAGAAAAGTGAAATATTCAAAAAATATTTTGATTTATTCTTTTCTCCTACTAAGCGTGAGGACAATGCTCCATTTTAACAGAGGAGAGCGTGTACGCTTTTGTATATATTTTGTAAGTATACCTTCAAGTAAGCTTGGGGGTGTTGTTTTGTTTGGTAATTTCTTGATTGTGGTGTTAAGGATTGATCACCCTTTTGCTTCACTTTTAGAAAAATCAAAATTTTAGCCTACCTTCACCCTTTATGCCTTACGTTACAACCTTGGGGAAAGACCTTTTCGACCTAGATAGTTGAGTCACTATTCGGGGGGAGGGCATGGCAGGTCATTGCTAAGGAAGGGGATGCATAGTCAGAAGTTGTCGAGTGAGCCTTAAAATAATTCTTTTCGGGTTCCTTGAGGGAATCATTGGGACACTCACTAAAGGGAGATTTGAGACTAGTGGGAGAGCAGCGGTAAGATTGTTTTGGGATGAAAGTGAATTAGCGGCAACTGGTTCCTATGCCTGGCATACTATGTTTCAATCAACCTTGGATATCTTGAAGCGTGCATACCTTAACTACTTGAGAAAGATTGTCATTCACTTGCATTATAGTCTTTATGTTGATTCATTTCATAAAGGTTTGTTTATTTTCATTTTCTTTCTTTTAATTTTATGGTTTGCCTCATTGATGTTTGGTGGAGTTATGGGTCAAACTCTTAGGATACCATACGAGACCTCGCTCGTCCTCGTAGCGACTACGGGGTTTAAAGGGGCTTGTTGTAGCTAAATACAACCGTGATTCCCTACGACAATGAGTTTAGTGTATATTCTTATCTTGAGTCTTTCTTTTTCGTTTTCAATAAAATTTTTCATCTTCTCTTTTTCTTGCTTTGTTTTGCTTTCTAGGGGCGAGCAAAAGTTTGCTTGAAAATTTGATGAGTGACATTTTGGCACTCATTTAGTTCCTTTTTGGATGATTTTTATTGTCTCTCTTTTCTTTAGTTTCCTTAGTTTTATTCTTGTTTTCTTCATTTTATGCTTTTTGTAGTTAGTTTCTCTATTTCATGCTTTTTTCTAGTTTACTTGTTATTTTCTCTTTGCCTTTTTATCTTTTGTTTGTAGGTGGTAATTGATTGTCGAATTCCCGAGCTTCCTCTCTACGAGGAATTTTCATTGTTTTTGTAACACAAAGAGAGAGTGAGAAGCTCCAAGTCAAAGAGTGGATCAAGGGGCAGAAGAAGCACTTCAAGGCTTTGTTATGCCTCCCACTCATGGCTTATACTCTTGAAATTCAAGAAAATTCACCGCCTACATTGTCCTCCATCATCGTCATCACGGATGTGTGCACCACTTGAGCTAGGACAAAGAGAAGAAGGGGAAAGACCCGGCCAGTGCGGGCGCACCAAAGGGAGTGCGGCCCTTTGGTGGTCGATGCGGCCGCACACCCGGTGCGTCTTGGGCGCACAGGTGTCCCGATGCGGCGCCATTCTCTGCTCTCAGGCGCCGCTACGAGGTCTATTTTCGGGCGTCATTACCCGACCTCCTCTTTGTATATATAGTTATTTCTACTATTTTTAGGAAGCTAGTTTTAGATTGAATTTGATAGTTAGTTTTGGGAGAATTTTAGAGAGAAACTCAAGAACTTTAGAGAGAGAAACCCATTAATTTTGGTGATTGCTCGAGGATTCCTCGTTGTATTTGATCTTTAGATCAAAGTTTTGGAAGGATCTTCTTCTTGATTTCAATACAAGTTTGATTTTAGTTGATTTCGTAGTTTTTCCATGTTATTGATGATTGATTTGGTTATTGATTGAATTTTGATTGATTTTGATTGATTGTTGGTTTAATCTTTGATTCCCCGATTATATTGATGATGAAATTGATGATTGTTGTAATTTGATCTTAATTCCTATGGCTAGTAGGTAGTTTCCTAGCTAGAGGAAATGAGTAGAAGCATGTATGAATGATGGATTAGTGTAATTTAGGGATAATTGATAGAGTTGATGATATATGATTAAATTTGGTTGATATTTCGTGGCCATGTGTGTGATTAATTGAGGTTTGACCATCTTTGATTAATACACTAGATTACATTGTAAACTTGCTGTGATACGGACCATTGATTAGTAAAAACTTTGCAATTTCACGATTGGGGTGCTTTGTGATTCCCTGGGTAGTGTTTAGCGTAGTTGACGCTAGATAGACCTCTAGGTGAACCATCTTAGTATGCTCTTTTGGATACATGATGATCTTCCTTCATATCACTTGACTTAGTGGTTTAACCTCCCAAAGTACCCGTTTCCATTGCGATTGCATGTGGTCCATTGCCTAGTGTTTTAGATTGATTTCATATAAGCTTTTGCATCACCCTAGTTTGTACTTAGTTGCCTCAAACCCCTTCTTGACTTCCGAAATAGCTTGGTTAGGCATCGTGCATTTGTAAAAATGTGTTTGACTTTCATTTAGTGCTTTGTTGAATGCATTACCTTTCCATTTGATTAGCTTTGTTTGAATGTGTTTGAATTTAATTTGATCAGCTATGTTGAATGCATTTGACTTTCATTTTGATTGAAAAGCTTTGTTTGAATGAGTTTGACTTTCATTTGACTTCGATTATATTAGTTGAATGCATTTGACTTTCATTTGATTCAAAAAGCTTTGTATGAATGTGTTTGACTTACGTTTGACTCGAATTTGTTTAGGGTAATTTTACCGCTATGTGGCCAGTCAGGAGGCGGCACGGTGTCGGCCGGATGGTCAACGGACTTATCCCTGTTTTACATTAATAGATCCCCGGATATTTGGGGAAAAGTCGACTGACGAATCCAGAAGCCGTAATGCGGGGCGCGGTATCAGCGGTGAGGTTACATCACTGTCGAGGCACAAATTCCCGAGACCACATAAATAGGGATTCCTGCGGACCACGTTTTTCATTTAATGTTGTCGCTGATAGAACTACTTAGCTAAGCACAGCGGTCCGCTTTAAATTTCACTTGCACCTTTTTCTCTCACTTTCAAGTTACTAGCGATGGGTCGGAGTGGAGCACCAGCCAGCAGTGTCTCGTTTGTCTTGCGATGCAGGCGCTCGGACCCGATCTTGAGTGTAACTACTCGGGGACAAAAAACACCCAAAACCTGATTATCTTTATTGAATGCATTTGACTTTCATTTTTATTTAAAGCTTTGTTTGAATGTGTTTGACTTTCATTTGGCTCGATTAACTTTGTTAAATGCATTTGACTTTCATTTGATTGATAAGCTTTGTTTAATGTGTTTGACTTTCATTTAGTCAGCTTTGTTGAATGCATTTGACTTTCAATTGATTGAAAAACTTTGTTTAGATCATGTTTGACTTTTATTTGAGTGAATGGCTTTTGTTAAATGCATTTGACTTCCAGATTGATTCAAAAAGCTTTGTTTGAATGTGTTTGATTTTTCATTTGACTCAATTTCTTTGTTGAATGCATTTGACTTTTAATTGATTCAAAATGCTTTTTGGTTTCTAAAGTGGTAAACATTCTTAAATTTTATTATCAATGGTAAACCTCGATTTGTGATTACCATTGAAAGAATATTAATTTAATAAAATCCTTAACTATGGTTAGTTTTTATTTTTTAATTATTAAATTATTATAAAATATTGATAAAAAAAAAACCTTCCCCATTGTTCATTTTTATAATCTGAAGAAAATGACAAATCCAGGAATGTCAAGTATTGAAAACAATCTTCTGCTCTCTAAATATTGAAAATGAGCCTATTCAAGAAAAATGTAAAGACATTCCGGCAACTAGTGAGATTATGAAGGAAACGAGGAGAGTGGACAAACAGGGGTAAAAACAAAAACCCACGTATTGTATTAGTAGTGATGCTTTATGTATTAGTACTAATAATGTAGTTTATTTTGTTCAATCAGAAAACAATTAAGGTTTATGTAATATTTGAAGAATGTTTTAGTAATCGAGAACAATTAAGTTTATGTAATTTAAAATTATGATGTGTTATTGTAATATTAATTAAATTTTGTGTAATATGAGTAATTTTTATTGATATTTCAATGCTTTAAAATTATAGCATGACATTATGTTTACTGACTTGATTTGATTTTCAAAAATAAAACAAAAATCCCTAGCGGAGGAGATAACCAGAAAATTACGACAATGAAGCTCAAGAAGAAGATTTTATCTTCCCTCAAAAAGCTACGACCAAAGGTAAATATATCAAATATATTCCCTCAATAAATAAAGAACATTTGTGTGAATCAATTTGTTCATATAAATATTTCTTTTGTTCAGGGAAGAAAATATGGAAAAAAAGGAGTAAAAAGAGTGAATGAAAAACAACCCAGGAAAAGCAAAAAATTTTCCAAGAACAAAGATGAGAGGTATAAATTACAATCCCTTTAACTTGTGATTCATTTACGATGTTCATTTGTATATGCATATATCACTAGTGGAAAAACATCATCCGTGCAGGAGCAAAAGGCCCATCCGTAGCAGGCGTGTCCGGGTGCATTGGCATCGCGGCTTTCCGTTACGGTGGCCAATATTCGTAACGAGCAATACGTAACT
